# Unified Gold(I/III)-Catalyzed Arylative, Vinylative, and Alkynylative Lactonization via Iminium-Directed Cyclization Enabled by Hemilabile Ligands

**DOI:** 10.1021/acs.orglett.6c01067

**Published:** 2026-04-27

**Authors:** Jorge C. Herrera-Luna, Riccardo Mobili, Cyril Ollivier, Virginie Mouriès-Mansuy, Louis Fensterbank

**Affiliations:** † 27063 Institut Parisien de Chimie Moléculaire Sorbonne Université, CNRS, 4 place Jussieu, 75005 Paris, France; ‡ Laboratoire d’Activation Moléculaire, Collège de France, Sorbonne Université, CNRS, 11 place Marcelin Berthelot, 75005 Paris, France

## Abstract

We report a unified strategy for the synthesis of functionalized lactones via hemilabile Au­(I)/Au­(III) redox catalysis. Oxidative addition of aryl, vinyl, or alkynyl iodides to Au­(I) is followed by iminium-directed π-activation and lactonization, using AgOTf as a halide scavenger. The method affords 49 lactones under mild, scalable conditions with broad functional group tolerance. Mechanistic studies support iminium and Au­(III) intermediates. Moreover, we present a new Au­(I)/(III) hemilabile complex that is particularly effective in alkynylation.

## Graphical abstract

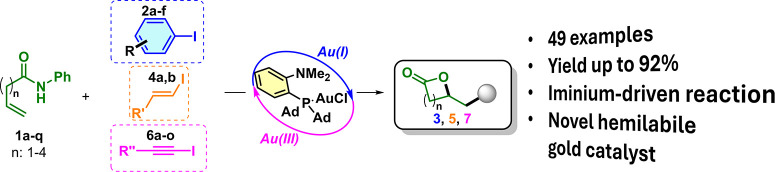

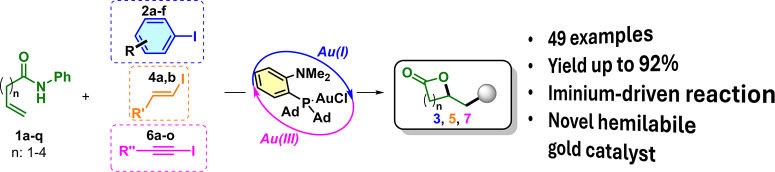

Recent advances in transition metal catalysis have enabled alternative approaches to heterocycle construction from olefin functionalization.^1–3^ Among these, gold catalysis has attracted attention for its capacity to activate π-systems.^4–9^ However, redox catalysis involving Au­(I)/Au­(III) cycles is less developed, mainly due to the reluctance of linear Au­(I) complexes to undergo oxidative addition.^10–24^ Rational ligand design has mitigated this issue by facilitating the conversion to square-planar Au­(III) species, thereby expanding the reactivity profile of gold.^21–24^

In particular, the MeDalphos ligand has proven to be critical for accessing Au­(I)/Au­(III) cycles.^25–28^ Bourissou’s group demonstrated its utility in oxidative addition of aryl iodides and π-activation, enabling oxy- and aminoarylation of alkenes (Scheme 1A).^26,27^ In 2021, they also described the oxovinylation of unsaturated alcohols; meanwhile, the alkynylation methodology proved to be ineffective.^28^ Notably, our group recently reported the first gold-catalyzed alkynylation of alkenols under mild conditions, mediated by silver-assisted oxidative addition (Scheme 1B).^29^ Furthermore, the Xie group described the alkynylation of norbornenes employing the gold­(I)/(III) strategy.^30^ Recently, Ribas and co-workers introduced MIC'^'N ligands that promoted the arylative lactonization of γ-alkenoic acids, although yields remained modest (Scheme 1C).^31^

**Scheme 1:**
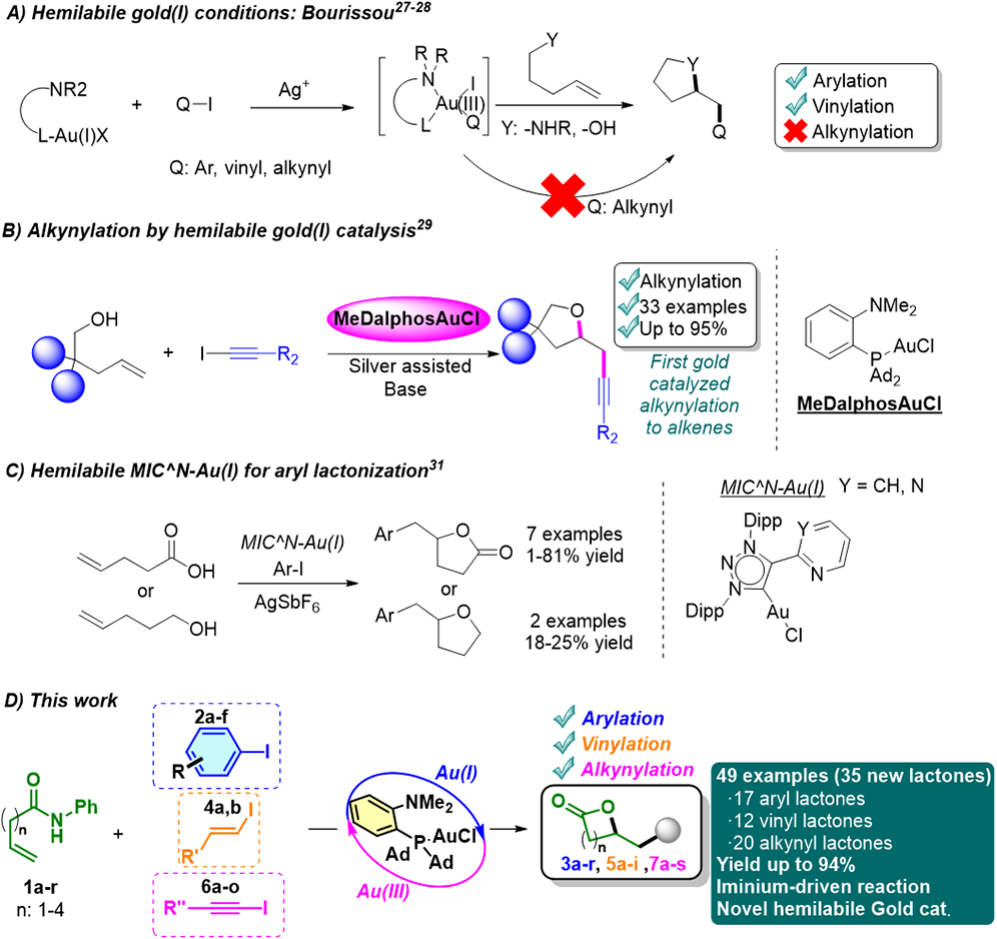
Strategies for Activating and Coupling π-Systems with a Gold­(I) Catalyst

Despite these advances, extending gold redox catalysis beyond aryl iodides, particularly to vinyl and alkynyl electrophiles, remains rare and synthetically valuable. In fact, alkynylation under Au­(I)/Au­(III) catalysis remains largely underexplored, especially in the context of heterocycle formation.

Lactones are privileged motifs in natural products and pharmaceutical agents.^32–34^ Developing efficient and versatile approaches to construct lactone-containing molecules remains a central pursuit in organic synthesis.^34^ Recently, Liu and Xu reported the synthesis of aryl lactones from the annulative addition of alkynes to *o*-iodo-arylamides.^35^

Building on our prior work on gold-catalyzed alkynylation of alkenols,^29^ we developed a general methodology for lactone synthesis through redox-active gold catalysis. Our approach relies on the in situ formation of an iminium intermediate from enamides, followed by gold­(III)-mediated π-activation and reductive elimination (Scheme 1D). This strategy enables arylation, vinylation, and alkynylation from a common platform using a suite of hemilabile ligands. Notably, we introduce one new ligand, MeCagephosAuCl (**Au2**), that demonstrates activity similar to that of MeDalphosAuCl. The resulting methodology exhibits a broad substrate scope, high efficiency, and excellent functional group tolerance, while mechanistic studies provide strong evidence for key iminium and Au­(III) intermediates.

During preliminary tests on the synthesis of lactams under Au­(I)/(III) catalysis and using our previous conditions for alkynylation of alkenols (Figure 1),^29^ we found that *N*-phenylhex-5-enamide (**1a**) afforded δ-valerolactone **3aa** in 28% yield. This unexpected result led us to reconsider the substrate class and to test 5-hexenoic acid (**1aa**) instead. Surprisingly, no reaction was observed. Moreover, the ester derivative (**1aa-OMe**) also did not work. Apparently, the amide group was essential for the formation of δ-valerolactone **3aa** under MeDalphosAuCl (**Au1**) catalysis in 1,2-dichloroethane (DCE) at 45 °C (Figure 1).

**Figure 1:**
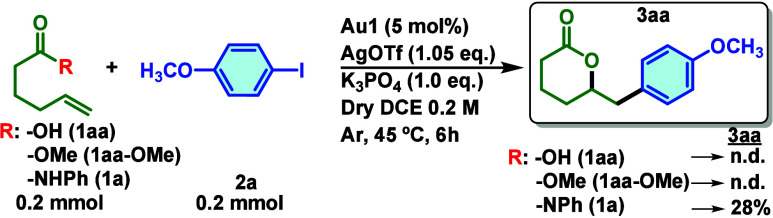
Preliminary test with alkene carboxylic acid (**1aa**), methyl ester (**1aa-OMe**), and enamide (**1a**). Conditions: 0.2 mmol of **1aa**, 0.2 mmol of **2a**, 0.01 mmol of **Au1** (MedalphosAuCl), 0.21 mmol of AgOTf, 0.2 mmol of K_3_PO_4_, and 1 mL of dry DCE (0.2 M) at 45 °C for 6 h. Yields were determined by ^1^H NMR with CH_2_Br_2_ as the internal standard.

Encouraged by this result, we optimized the reaction conditions. Under an argon atmosphere, in DCE, with 5 mol % MeDalphosAuCl (**Au1**), 1.05 equiv of AgOTf, and 1 equiv of NaHCO_3_, **3aa** was obtained in 92% yield (Table 1, entry 1). The reaction was sensitive to temperature and concentration (Table 1, entries 2 and 3, respectively), and control experiments confirmed the essential role of both gold and silver (Table 1, entry 4). The presence of a counteranion in the form of a silver salt has been demonstrated to have a significant impact on the reactivity of the system (Table 1, entry 5). In addition, other apolar solvents have exhibited results that are slightly less optimal in comparison to those of DCE (Table 1, entry 6). The reactivity observed in the air atmosphere (Table 1, entry 7) was consistent with the reactivity in the Ar atmosphere. Furthermore, the presence of water in the solvent did not exert an influence on the reactivity (Table 1, entry 8). Notably, under nonbasic conditions, an intermediate iminium species (**3aa-iminium** (Table 1, entry 9)) accumulated.

**Table 1:**
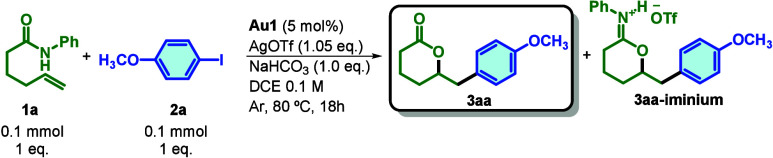
Optimization of the Conditions for Aryl Lactonization Catalyzed by Hemilabile Gold­(I)^a^

entry	variation	yield of **3aa** (%)^c^
1	–	92
2	rt instead of 80 °C	3
3	0.2 M/0.05 M instead of 0.1 M	71/71
4	without **Au1** and/or AgOTf	nd
5	AgBF_4_/AgNTf_2_/AgSbF_6_ instead of AgOTf	73/63/35
6	DCM/toluene instead of DCE	85/79
7	air instead of an Ar atmosphere	82
8	moist DCE instead dry DCE	92
9	without NaHCO_3_	9/83^b^

a With 0.1 mmol of **1a**, 0.1 mmol of **2a**, 0.005 mmol of **Au1**, 0.105 mmol of AgOTf, 0.1 mmol of NaHCO_3_, and 1 mL of dry DCE (0.1 M). Abbreviations: AgBF_4_, silver­(I) tetrafluoroborane; AgNTf_2_, silver­(I) bis­(trifluoromethanesulfonyl)­imide; AgSbF_6_, silver­(I) hexafluoroantimonate; DCM, dichloromethane.

b Yield of **3aa-iminium**.

c Yields were determined by ^1^H NMR with CH_2_Br_2_ as the internal standard.

The amide substituent was also evaluated (see Table S32), showing activity similar to that of the *tert*-butyl substituent and lower performance than that of the free amide.

In addition to MeDalphosAuCl (**Au1**), other gold catalysts were screened, including a new hemilabile gold­(I) chloride complex based on the sterically demanding but electron-poor cage phosphine 1,3,5,7-tetramethyl-6-phenyl-2,4,8-trioxa-6-phosphaadamantane (**Au2** (Figure 2 and Scheme S1)).^36^ MeCagephosAuCl (**Au2**) was synthesized in high yield, and the structure was confirmed by single-crystal X-ray diffraction (SCXRD) analysis (see section 7 of the Supporting Information). Although MeDalphosAuCl (**Au1**) remained the most effective catalyst, MeCagephosAuCl (**Au2**) proved to be a highly competitive ligand in Au­(I)/(III) redox chemistry, performing better than Karphos-based catalysts^37^ (**Au3** and **Au4**). No activity was observed with **Au5**.

**Figure 2:**
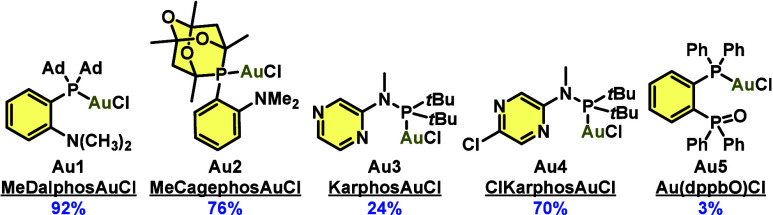
Hemilabile gold­(I) catalysts used in the screening for arylative, vinylative, and alkynylative lactonization. Yields of aryl lactone **3aa** obtained using the optimized conditions (Table 1, entry 1) are colored blue.

With the optimized conditions in hand, we next evaluated the generality of the transformation (Figure 3). Various lactone ring sizes were accessible, including β-lactones (**3da**, 32%), γ-lactones (**3ca**, 87%), δ-lactones (**3aa**, 92%), and ε-lactones (**3ba**, 73%), with good to excellent yields. Moreover, δ-valerolactone (**3aa**) showed similar reactivity when scaled up to 1 mmol (91%).

**Figure 3:**
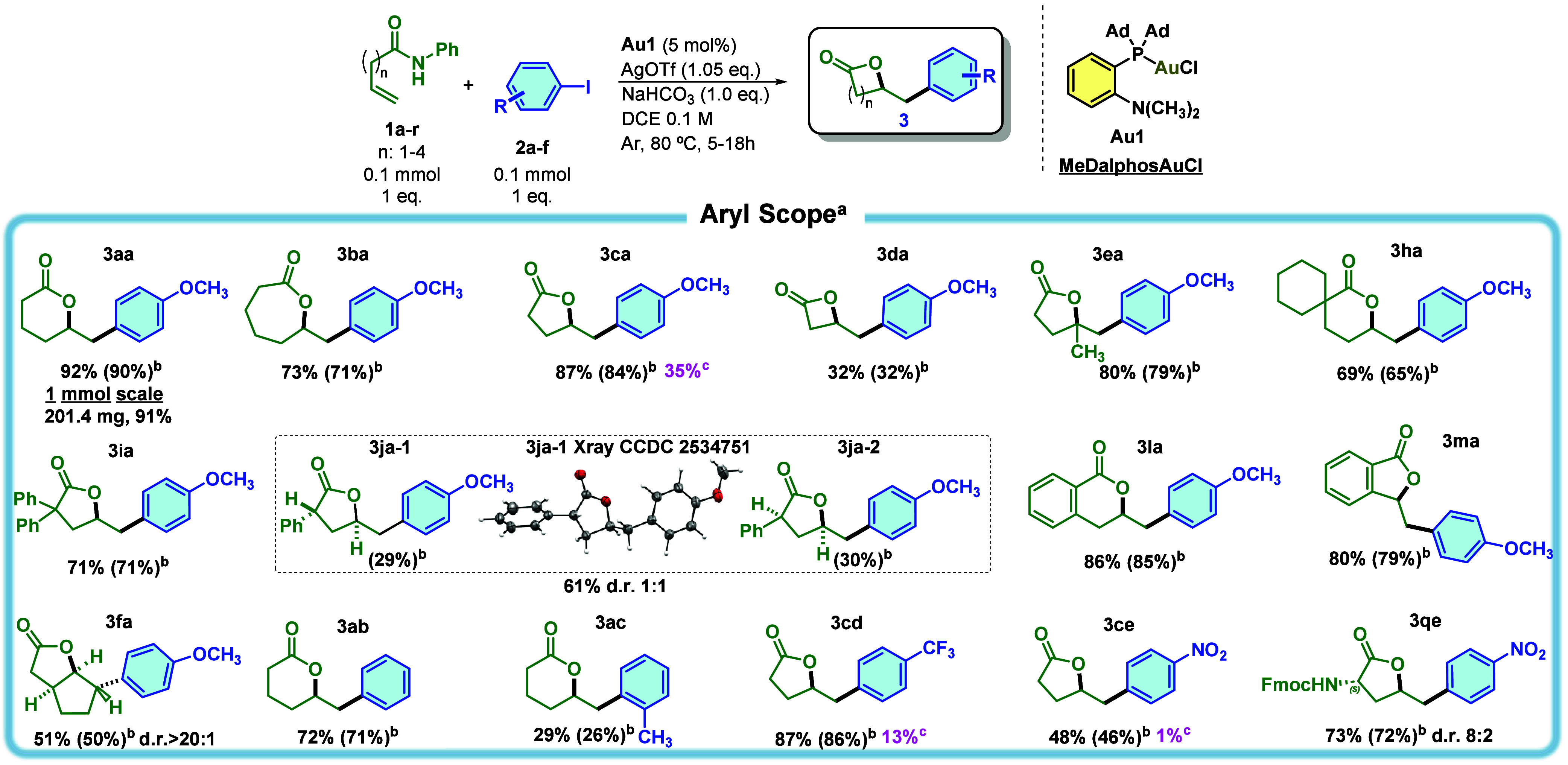
Aryl scope. ^a^With 0.1 mmol of **1**, 0.1 mmol of **2**, 0.005 mmol of **Au1**, 0.105 mmol of AgOTf, 0.1 mmol of NaHCO_3_, and 1 mL of dry DCE (0.1 M) at 80 °C for 18 h. ^b^Isolated yields in parentheses. ^c^Yield described in ref 31.

Trisubstituted alkenes and α-substituted enamides performed well, providing access to structurally complex lactones (**3ea** and **3ha–3ja**). The diastereoselectivity of **3ja-1** was assessed via X-ray crystallography, revealing *anti* disposition in key isomer. Moreover, challenging substrates like 1,2-disubstituted enamides and *Z*-alkenes were found to afford lactones (**3fa**, **3la**, and **3ma**) in high yields. The diastereoselectivity of **3fa** was confirmed (compared with **5fa**, resolved by SCXRD analysis), revealing two *syn* hydrogen disposition at positions 3 and 6, with an *anti* hydrogen at the aryl position.

Aryl iodides bearing electron-donating or -withdrawing substituents were compatible, and the method outperformed previous systems in direct comparisons. For instance, lactones **3ca**, **3cd**, and **3ce** were previously reported with yields of 35%, 13%, and 1%, respectively, using MIC'^'N-based gold catalysts,^31^ while our protocol delivered the same compounds in 87%, 87%, and 48% yields, respectively. Further validation came from the synthesis of γ-lactone **3qe**, an homologue scaffold of β-obafluorin, in 73% yield and an 8:2 diastereomeric ratio. This highlights the method’s potential in medicinal chemistry and natural product synthesis. However, nonterminal alkenes (**1g**) did not work for arylations (section 5 of the Supporting Information).

Next, we turned our attention to extending the methodology beyond aryl iodides. Vinylation (Figures S11–S20) and alkynylation (Figures S21–S30) were successfully achieved in good yields after optimization of the reaction conditions using **Au1**. Vinylation afforded lactone **5aa** in 84% yield at 90 °C (Figure 4 and Table S20), while alkynylation gave **7aa** in 62% yield at 45 °C (Figure 4 and Table S30). Also in these cases, the other gold catalyst was less active than MeDalphosAuCl, with only **Au2** being able to match its performance in alkynylation (62% vs 57% (Scheme S1)). As for aryl coupling (**3aa**), the desired product was obtained through iminium intermediate formation before hydrolysis (section 6 of the Supporting Information). Temperature was critical for alkynylation. Both higher and lower temperatures resulted in reduced yields or incomplete conversion. These are the first described examples of gold-catalyzed alkynylative lactonization with isolated products.

**Figure 4:**
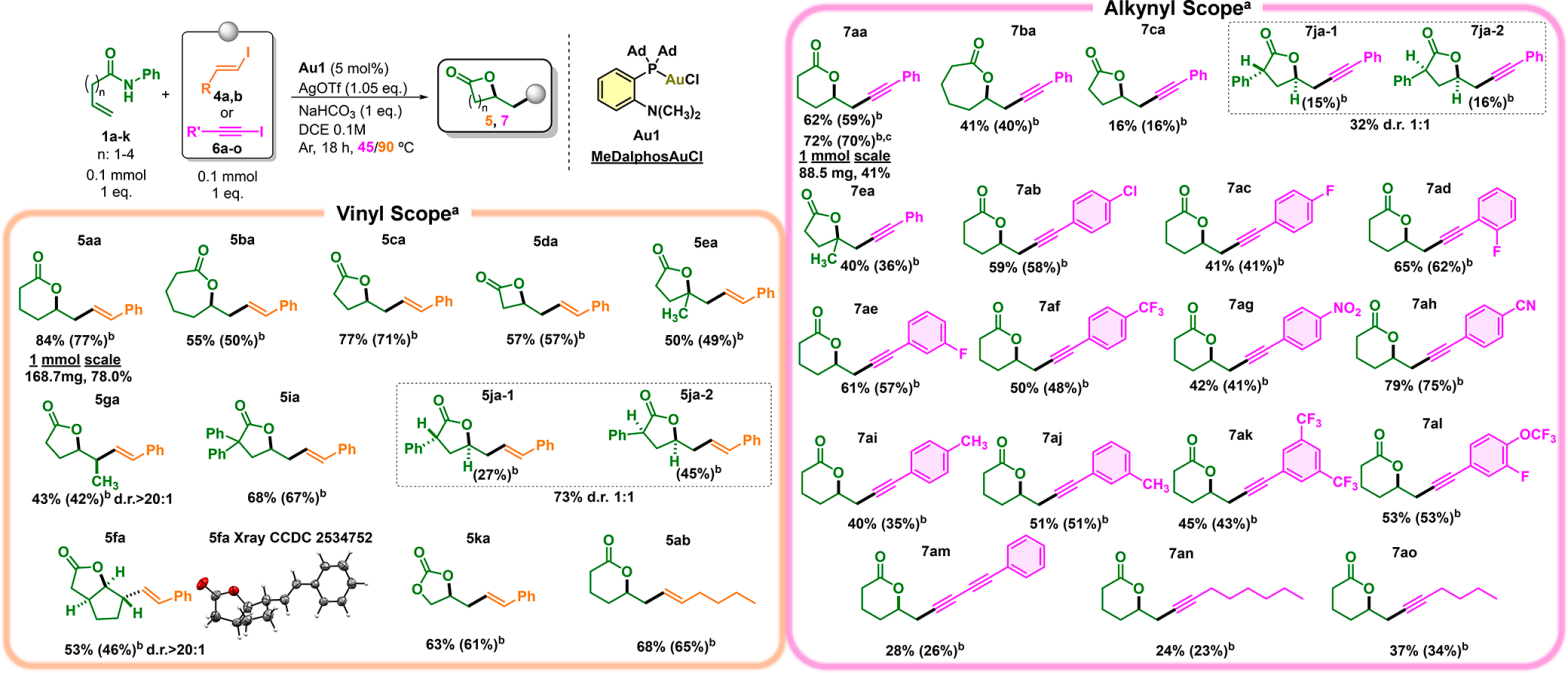
Vinyl and alkynyl scope. ^a^With 0.1 mmol of **1**, 0.1 mmol of **4** or **6**, 0.005 mmol of MedalphosAuCl, 0.105 mmol of AgOTf, 0.1 mmol of NaHCO_3_, and 1 mL of dry DCE (0.1 M) at 90 °C (with **4**) or 45 °C (with **6**) for 18 h. ^b^Isolated yields in parentheses. ^c^With 0.1 mmol of **1a**, 0.15 mmol of **6a**, 0.01 mmol of MedalphosAuCl, 0.21 mmol of AgOTf, 0.1 mmol of NaHCO_3_, and 1 mL of dry DCE (0.1 M) at 45 °C for 18 h.

The vinylation scope was evaluated using 12 substrates (Figure 4), including a scale-up to 1 mmol for δ-**5aa** (78% yield). β-Valerolactones (**5da**, 57%), γ-valerolactones (**5ca**, 77%), δ-valerolactones (**5aa**, 84%), and ε-valerolactones (**5ba**, 55%) were obtained with yields up to 84% from (*E*)-(2-iodovinyl)­benzene (**4a**).

Enamides bearing trisubstituted alkenes, diphenyl, or phenyl groups reacted efficiently, affording products **5ea**–**5ja** in 43–73% yields. In detail, only one diastereoisomer of **5ga** was detected in moderate yield (43% (Figure 4)). Vinylation of **1j** gave two diastereoisomers (1:1 ratio) of **5ja** (73%), and the geometry of **5ja-1** was determined by comparison with **3ja-1** (^1^H NMR). In the case of *Z*-enamide (**1f**), only one diastereoisomer (**5fa**, 53%) was produced and its geometry was determined by SCXRD analysis. Furthermore, oxolanone (**5ka**, 63%) was obtained from carbamate **1k**, and an *E*-aliphatic vinyl iodide (**4b**) was used to produce lactone **5ab** with a high yield of 68%.

For alkynylation (Figure 4), 20 lactones were synthesized, including electron-deficient and electron-rich alkynes, diynes, and aliphatic iodides. Electron-deficient aryl groups improved reactivity, while fluorinated positions (*ortho*, **7ad** > *meta*, **7ae** > *para*, **7ac**) influenced yields. In more depth, the ε (**7ba**, 41%), γ (**7ca**, 16%), and δ (**7aa**, 62%) forms were obtained, but no alkynyl β-lactone was obtained (Figure 4). δ-Valerolactone (**7aa**) was the optimal ring length for alkynylation, reaching 72% with larger quantities of reactants. When scaled up to 1 mmol, the yield decreased to 41%. Alkynylation of **1j** produced two diastereoisomers (1:1 ratio), with a low yield of 31% for **7ja**; the geometry of **7ja-1** was determined by comparison with that of aryl analogue **3ja-1** (^1^H NMR). Enamides **1k** and **1g** were ineffective for alkylation; meanwhile, **1l** and **1m**, which were effective for arylation, were not suitable for vinylation or alkylation.

Mechanistic analysis revealed key steps and provided evidence for the iminium-driven activation pathway. First, we monitored the treatment of enamide **1a** with AgOTf in deuterated dichloromethane at 40 °C by NMR (^1^H and ^13^C). A downfield shift of the alkene signals was observed, along with the appearance of new signals consistent with iminium formation (Figure 5 and Figures S2 and S3), as described previously.^38^ On the other hand, **Au1-I** was detected by ^31^P NMR (74 ppm), confirming the oxidative addition and Au­(III) formation upon addition of **2a** to MeDalphosAuCl in the presence of AgOTf (Figure 5 and Figure S5). Subsequent addition of **1a** (in equilibrium with its iminium form in the presence of AgOTf) to **Au1-I** gave new signals in the ^31^P NMR spectrum (84–95 ppm), assigned to Au­(III) intermediates (**Au1-1a**/**3aa** (Figure 5 and Figure S5), followed by the formation of **3aa-iminium**, which hydrolyzed to **3aa** in the presence of NaHCO_3_ and traces of water (Figure 5 and Figure S4).

**Figure 5:**
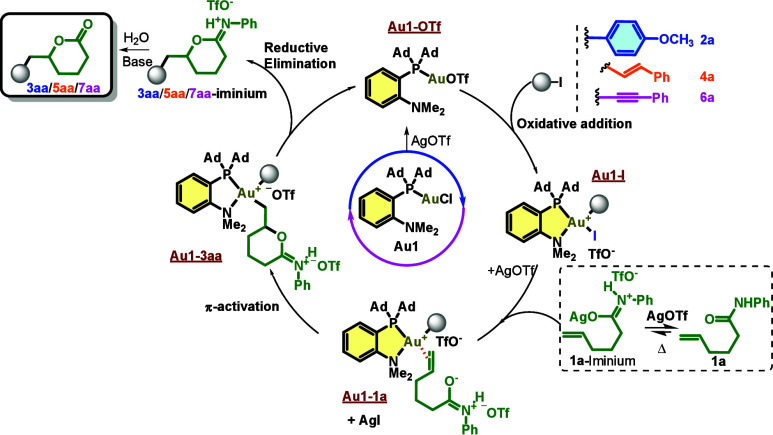
Proposed mechanism for hemilabile gold­(I)-catalyzed iminium-driven functionalized lactonization.

Despite the existence of alternative mechanisms, involving the possible formation of a triflate ester intermediate,^39^ our proposed rationale appears to be more plausible. This is evidenced by the observation that the reaction occurs in the presence of other silver counteranions (Table 1, entry 5, and Table S4).

These results underscore the critical role of transient iminium species and ligand-controlled gold redox chemistry. Compared with previous systems limited to aryl iodides and requiring oxidants or specialized substrates, our methodology unifies multiple electrophilic partners and nucleophilic substrates under mild, redox-neutral conditions. This generality and versatility represent a distinct advancement in gold redox catalysis and open new doors for late-stage functionalization and heterocycle synthesis.

We have developed a versatile Au­(I)/Au­(III)-catalyzed strategy for the synthesis of functionalized lactones via iminium-directed cyclization. The method tolerates aryl, vinyl, and alkynyl iodides and enables 49 examples under mild conditions. A new gold­(I) complex, MeCagephosAuCl (**Au2**), was introduced, expanding the scope of gold redox catalysts. Mechanistic studies provided evidence for key intermediates, including iminium and Au­(III) species, that play a critical role in enabling high reactivity and selectivity. This approach offers a unified platform for the efficient construction of lactones and sets the stage for further exploration of ligand design in redox-active gold catalysis.

## Supplementary Material



## Data Availability

The data underlying this study are available in the published article and its Supporting Information.

## Notes

Experimental procedures, optimization, mechanistic insights, XRD data, and NMR spectra (PDF)

## ∇

Universitat Politècnica de València, Chemistry Department, 46022 Valencia, Spain
