# Supplementary material for: Unified Gold(I/III)-Catalyzed Arylative, Vinylative, and Alkynylative Lactonization via Iminium-Directed Cyclization Enabled by Hemilabile Ligands
Source: Org Lett. 2026 Apr 27;28(18):5711–6. doi: 10.1021/acs.orglett.6c01067 (PMC13162315; doi:10.1021/acs.orglett.6c01067)

Supplementary information

Unified Gold(I/III)-Catalyzed Arylative, Vinylative, and Alkynylative Lactonization via Iminium-Directed Cyclization Enabled by Hemilabile Ligands

Jorge C. Herrera-Luna,[a†]* Riccardo Mobili,[a] Cyril Ollivier,[a] Virginie Mouriès-Mansuy,[a]* and Louis Fensterbank[a,b]*

[a]Sorbonne Université, CNRS, Institut Parisien de Chimie Moléculaire, 4 Place Jussieu, CC 229, 75252, Paris Cedex 05, France
[b]Collège de France, Chaire Activations en Chimie Moléculaire, Paris, France.
[†]Universitat Politècnica de València, Chemistry Department, 46022 Valencia, Spain.

TABLE OF CONTENTS

1. Materials and methods S4

2. Synthesis of substrates S5

2.1. General procedure 1 (GP1): Synthesis of enamides S5

*2.2. Aryl Iodides (**2**)* S10

*2.3. Synthesis of vinyl iodides (**4**)* S10

*2.4. General procedure 2 (GP2): Synthesis of alkynyl iodides (**6**)* S11

2.5. Alternative synthesis of alkynyl iodide S14

2.6. Synthesis of gold(I) catalysts S15

3. Optimization S17

*Table S1. Temperature optimization for **3aa*** S17

*Table S2. Concentration optimization for **3aa*** S17

*Table S3. Base screening for **3aa*** S18

*Table S4. Halide Scavenger screening for **3aa*** S18

*Table S5. Solvent screening for **3aa*** S19

*Table S6. Gold catalyst screening for **3aa*** S20

*Table S7. Control experiments for **3aa*** S20

*Table S8. Amount of base optimization for **3aa*** S21

*Table S9. Amount of silver optimization for **3aa*** S21

*Table S10. Amount of gold optimization for **3aa*** S21

*Table S11. Temperature optimization for **5aa*** S22

*Table S12. Concentration optimization for **5aa*** S22

*Table S13. Base screening for **5aa*** S23

*Table S14. Halide Scavenger screening for **5aa*** S23

*Table S15. Solvent screening for **5aa*** S24

*Table S16. Gold catalysts screening for **5aa*** S25

*Table S17. Amount of base optimization for **5aa*** S25

*Table S18. Amount of silver optimization for **5aa*** S26

*Table S19. Amount of gold optimization for **5aa*** S26

*Table S20. Control experiments for **5aa*** S26

*Table S21. Temperature optimization for **7aa*** S27

*Table S22. Concentration optimization for **7aa*** S27

*Table S23. Base screening for **7aa*** S28

*Table S24. Halide Scavenger screening for **7aa*** S28

*Table S25. Solvent screening for **7aa*** S29

*Table S26. Gold catalysts screening for **7aa*** S30

*Table S27. Amount of base optimization for **7aa*** S30

*Table S28. Amount of silver optimization for **7aa*** S31

*Table S29. Amount of gold optimization for **7aa*** S31

*Table S30. Control experiments for **7aa*** S31

*Table S31. Kinetic profile for **3aa**, **5aa**, and **7aa*** S32

*Figure S1. Kinetic plot of **3aa**, **5aa**, and **7aa*** S32

*Table S32. Amide substituent screening for **3aa*** S33

Scheme S1. Hemilabile gold(I) catalysts screening for arylative, vinylative, and alkynylative lactonization S33

4. Mechanistic insights S34

*Figure S2. Iminium formation assisted by silver triflate from **1a** by ^{1}H NMR* S34

*Figure S3. Iminium formation assisted by silver triflate from **1a** by ^{13}C NMR* S34

*Figure S4. In situ formation of iminium product of **3aa** by ^{1}H NMR* S35

*Figure S5. In situ formation of iminium product of **3aa** by ^{31}P NMR* S35

*Figure S6. ^{1}H NMR comparison between of **3aa** & **3aa**-iminium* S36

*Figure S7. ^{13}C NMR comparison between of **3a** & **3aa**-iminium* S36

5. Unsuccessful substrates S37

6. Compounds Characterization S38

6.1. General procedure 3 (GP3) S38

7. XRD Data S60

*Figure S8. ORTEP representation of **3ja-1** CCDC: 2534751* S60

*Figure S9. ORTEP representation of **5ka** CCDC: 2534752* S61

*Figure S10. ORTEP representation of **Au2** CCDC: 2534753* S61

8. References S62

9. NMR spectra S64

1. Materials and methods

All reactions involving air sensitive reagents or intermediates were carried out in pre-heated glassware under an argon atmosphere using standard Schlenk techniques. All solvents and chemicals were used as received from suppliers (Abcr, Alfa Aesar, Sigma Aldrich, TCI, BLDpharm, Doug, VWR). The solvents (Et_2O, CH_2Cl_2) were purified by distillation over calcium hydride, and THF over sodium/benzophenone under dry Argon atmosphere. 1,2-Dichloroethane was dried over activated 4 Å molecular sieves under Ar atmosphere for at least 3 days. Aryl iodides were commercially available and used directly without further purification. Chromatographic purifications of products were accomplished using flash column chromatography (FCC) on Silica 60 Å (40 – 63 µm) from Macherey-Nagel. Thin layer chromatography (TLC) was performed on Alugram Xtra SIL G UV254 aluminium sheets of silica gel 60 with fluorescence indicator from Macherey-Nagel. Filtrations through Celite® were performed using Hyflo Super Cel from Fluka. ^{1}H NMR spectra were recorded on a Brucker 400 AVANCE or 300 AVANCE (400 and 300 MHz respectively) and are calibrated with residual $CDCl_3$ protons signals at δ 7.26 ppm. ^{13}C NMR spectra were recorded on a Brucker 400 AVANCE or 300 AVANCE (100 and 75 MHz respectively) and are calibrated with $CDCl_3$ signal at δ 77.16 ppm. ^{31}P NMR spectra were recorded at 122 MHz or 162 MHz and were calibrated with H_3PO_4 (85% in water) as external standard at δ 0.0 ppm and ^{19}F spectra were recorded at 376.5 or 282.4 MHz and were calibrated with trifluorotoluene in $CDCl_3$ as external standard at δ -63.0 ppm. Data are reported as follows: chemical shift (δ ppm), multiplicity (s = singlet, d = doublet, t = triplet, q = quartet, qt = quintuplet, m = multiplet, bs = broad signal), coupling constant (Hz) and integration. High resolution mass spectrometry was performed on a microTOF (bruker) or an LTQ-Orbitrap (Thermo Fisher Scientific) by electrospray (ESI) or Atmospheric Pressure Chemical Ionization (APCI). Melting points were measured on Stuart Scientific Melting Point Apparatus SMP3.

2. Synthesis of substrates

2.1 General procedure 1 (GP1): Synthesis of enamides

N-phenylhex-5-enamide (**1a**), *N*-phenylhept-6-enamide (**1b**), *N*-phenylpent-4-enamide (**1c**), *N*-phenylbut-3-enamide (**1d**), 4-methyl-N-phenylpent-4-enamide (**1e**), 2-(cyclopent-2-en-1-yl)-N-phenylacetamide (**1f**), 1-(but-3-en-1-yl)-*N*-phenylcyclohexane-1-carboxamide (**1g**), *N*,2,2-triphenylpent-4-enamide (**1h**), N,2-diphenylpent-4-enamide (**1i**), 2-allyl-N-phenylbenzamide (**1j**), 2,2-diallyl-N^1,N^3-diphenylmalonamide (**1k**), hex-5-enamide (**1l**), and *N*-hydroxyhex-5-enamide (**1m**) were prepared according to reported procedure[40] from the corresponding carboxylic acid.

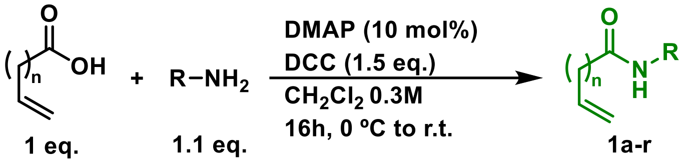

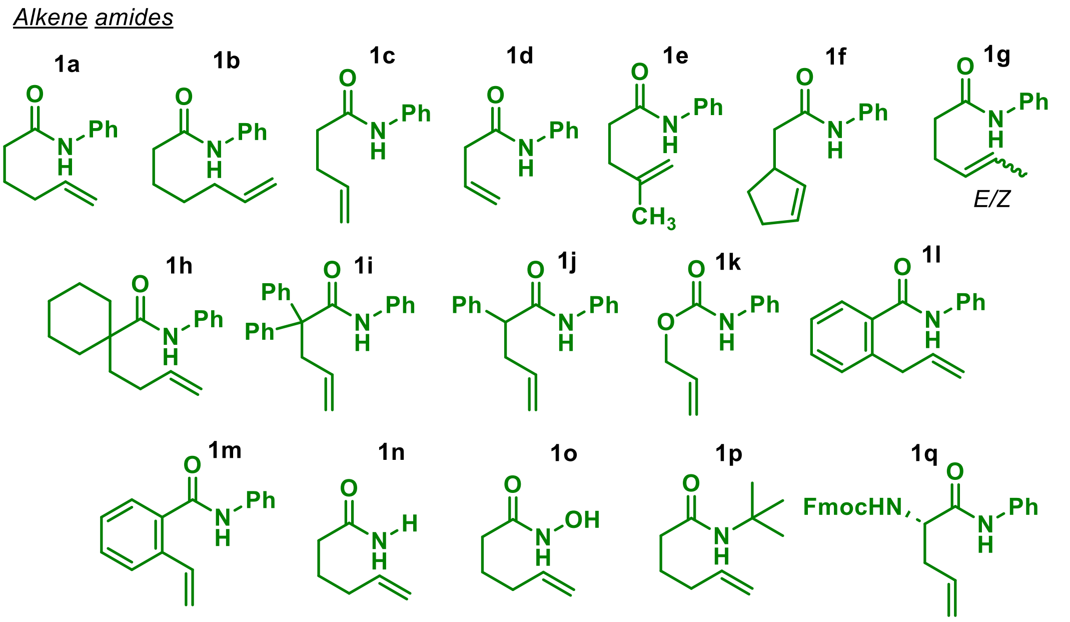

A solution of hex-5-enoic acid (1.56 mL, 13.14 mmol, 1 eq.), DMAP (4-dimethylaminopyridine, 159 mg, 10 mol%), aniline (1.2 mL, 1.1 eq.) in dry CH_2Cl_2 (45 mL) is cold down to 0 °C (ice-water bath). Then, DCC (*N,N'*-dicyclohexylcarbodiimide, 2.7 g, 2.5 eq.) is added in one portion to the mixture and the reaction is stirred overnight at r.t. (16-22 h). After that, 5 mL of HCl 2 M aq. sol. is injected, and the mixture is stirred vigorously for 10 min. The organic phase is separated and washed with 20 mL of $NaHCO_3$ aq. sol. 3 times and brine. Organic phase is dried over $MgSO_4$, filtered and concentrated. Purified by flash column chromatography with Hexane:EtOAc (100:0 to 90:10) affording **1a** as white solid (2.1 g, 85%)

1a. *N*-phenylhex-5-enamide

1a

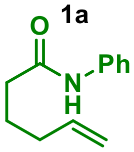

The compound was prepared according to the *GP1* purified by flash column chromatography with a mixture of hexane:EtOAc (100:0 to 90:10) yielding 2.1 g (85%) as white solid.

Spectrum

^{1}H NMR (300 MHz, $CDCl_3$) δ 7.51 (d, *J* = 7.9 Hz, 2H), 7.31 (t, *J* = 7.8 Hz, 3H), 7.24 (s, 1H), 7.10 (t, *J* = 7.4 Hz, 1H), 5.80 (tt, *J* = 13.3, 5.1 Hz, 1H), 5.11 – 4.97 (m, 2H), 2.36 (t, *J* = 7.5 Hz, 2H), 2.15 (q, *J* = 7.1 Hz, 2H), 1.85 (q, *J* = 7.4 Hz, 1H) ppm.
In agreement with the bibliography[Error! Bookmark not defined.].

1b. *N*-phenylhept-6-enamide

1b

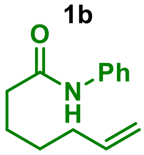

The compound was prepared according to the *GP1* from hept-6-enoic acid (3 mmol) purified by flash column chromatography with a mixture of hexane:EtOAc (100:0 to 90:10) yielding 439.6 mg (98%) as pale-yellow oil.

Spectrum

^{1}H NMR (300 MHz, $CDCl_3$) δ 7.51 (d, *J* = 7.9 Hz, 2H), 7.31 (t, *J* = 7.8 Hz, 2H), 7.19 (s, 1H), 7.10 (t, *J* = 7.4 Hz, 1H), 5.81 (ddt, *J* = 16.9, 10.2, 6.7 Hz, 1H), 5.07 – 4.92 (m, 2H), 2.36 (t, *J* = 7.5 Hz, 2H), 2.10 (d, *J* = 7.3 Hz, 2H), 1.83 – 1.67 (m, 2H), 1.56 – 1.40 (m, 2H) ppm.
In agreement with the bibliography[41].

1c. *N*-phenylpent-4-enamide

1c

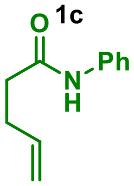

The compound was prepared according to the *GP1* from penten-4-oic acid (3 mmol) purified by flash column chromatography with a mixture of hexane:EtOAc (100:0 to 90:10) yielding 1.26 g (75%) as white solid.

Spectrum

^{1}H NMR (300 MHz, $CDCl_3$) δ 7.50 (d, *J* = 8.0 Hz, 2H), 7.31 (m, 3H), 7.10 (t, *J* = 7.4 Hz, 1H), 5.87 (ddd, *J* = 16.5, 10.6, 5.7 Hz, 1H), 5.14 – 5.01 (m, 2H), 2.48 (q, *J* = 3.9 Hz, 4H) ppm.
In agreement with the bibliography[Error! Bookmark not defined.].

1d. *N*-phenylbut-3-enamide

1d

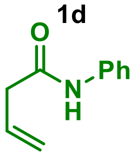

The compound was prepared according to the *GP1* from but-3-enoic acid (33 mmol) purified by flash column chromatography with a mixture of hexane:EtOAc (100:0 to 90:10) yielding 3.67 g (69%) as white solid.

Spectrum

^{1}H NMR (400 MHz, $CDCl_3$) δ 7.51 (d, *J* = 7.1 Hz, 1H), 7.43 (s, 1H), 7.31 (t, *J* = 7.9 Hz, 2H), 7.10 (t, *J* = 7.4 Hz, 1H), 6.03 (ddt, J = 17.3, 10.4, 7.2 Hz, 1H), 5.37 – 5.27 (m, 2H), 3.18 (dt, *J* = 7.2, 1.3 Hz, 2H) ppm.
In agreement with the bibliography[42].

1e. 4-methyl-*N*-phenylpent-4-enamide

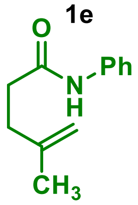
1e

The compound was prepared according to the *GP1* from 4-methylpent-4-enoic acid (3.5 mmol) purified by flash column chromatography with a mixture of hexane:EtOAc (100:0 to 90:10) yielding 417 mg (63%) as white solid.

Spectrum

^{1}H NMR (300 MHz, $CDCl_3$) δ 8.26 (s, 1H), 7.55 (d, *J* = 7.4 Hz, 2H), 7.29 (dd, *J* = 8.6, 7.2 Hz, 2H), 7.10 (t, *J* = 7.3 Hz, 1H), 4.77 (d, *J* = 13.0 Hz, 2H), 2.64 – 2.35 (m, 4H), 1.76 (s, 3H) ppm.
In agreement with the bibliography[Error! Bookmark not defined.].

1f. 2-(cyclopent-2-en-1-yl)-*N*-phenylacetamide

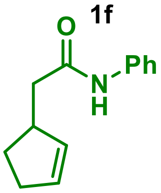
1f

The compound was prepared according to the *GP1* from 2-(cyclopent-2-en-1-yl)acetic acid (2 mmol) purified by flash column chromatography with a mixture of hexane:EtOAc (100:0 to 90:10) yielding 365.7 mg (78%) as colorless oil.

Spectrum

^{1}H NMR (400 MHz, $CDCl_3$) 8.71 (s, 1H), 7.54 (d, *J* = 7.7 Hz, 2H), 7.30 – 7.16 (m, 2H), 7.07 – 6.99 (m, 1H), 6.02 – 5.51 (m, 2H), 3.15 (ddt, *J* = 9.0, 3.9, 2.1 Hz, 1H), 2.49 – 2.18 (m, 4H), 2.07 (dtd, *J* = 13.5, 8.5, 5.1 Hz, 1H), 1.56 – 1.45 (m, 1H) ppm.
In agreement with the bibliography[43].

1g. *N*-phenylhex-4-enamide

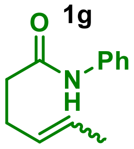
1g

E/Z

The compound was prepared according to the *GP1* from hex-4-enoic acid (4.38 mmol) purified by flash column chromatography with a mixture of hexane:EtOAc (100:0 to 90:10) yielding 510 mg (93%) as pale-yellow oil.

Spectrum

^{1}H NMR (400 MHz, $CDCl_3$) δ 8.73 (s, 1H), 7.59 (d, *J* = 8.0 Hz, 2H), 7.28 (t, *J* = 7.9 Hz, 2H), 7.09 (t, *J* = 7.4 Hz, 1H), 5.58 – 5.38 (m, 2H), 2.43 (ddt, *J* = 14.8, 11.4, 4.7 Hz, 4H), 1.65 (d, *J* = 5.5 Hz, 3H) ppm.
In agreement with the bibliography[Error! Bookmark not defined.].

1h. 1-(but-3-en-1-yl)-*N*-phenylcyclohexane-1-carboxamide

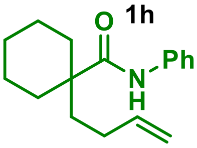
1h

The compound was prepared according to the *GP1* from 1-(but-3-en-1-yl)cyclohexane-1-carboxylic acid (1.65 mmol) purified by flash column chromatography with a mixture of hexane:EtOAc (100:0 to 90:10) yielding 339.7 mg (80%) as yellow oil.

Spectra

^{1}H NMR (400 MHz, $CDCl_3$) δ 7.52 (d, *J* = 7.6 Hz, 2H), 7.33 (t, *J* = 7.8 Hz, 3H), 7.11 (t, *J* = 7.4 Hz, 1H), 5.85 – 5.70 (m, 1H), 5.04 – 4.88 (m, 2H), 2.14 – 2.00 (m, 4H), 1.69 – 1.35 (m, 8H) ppm.
^{13}C NMR (101 MHz, $CDCl_3$) δ 174.6 (C), 138.5 (CH), 138.1 (C), 129.1 (CH), 124.4 (CH), 120.3 (CH), 114.8 (CH_2), 47.3 (C), 34.6 (CH_2), 28.6 (CH_2), 26.2 (CH_2), 23.1 (CH_2) ppm.
HRMS (ESI) m/z: [M+H]+ Calcd for $C_{17}H_{23}NOH$ 258.1852. Found 258.1853

1i. *N*,2,2-triphenylpent-4-enamide

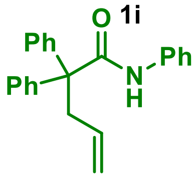

1i

The compound was prepared according to the *GP1* from 2,2-diphenylpent-4-enoic acid (3.61 mmol) purified by flash column chromatography with a mixture of hexane:EtOAc (100:0 to 90:10) yielding 528 mg (43%) as colorless oil.

Spectrum

^{1}H NMR (300 MHz, CD_3CN) δ 7.88 (s, 1H), 7.50 – 7.28 (m, 14H), 7.16 – 7.04 (m, 1H), 5.78 (ddt, *J* = 17.1, 10.2, 6.9 Hz, 1H), 5.12 – 4.90 (m, 2H), 3.36 (dt, *J* = 6.9, 1.4 Hz, 2H) ppm.
In agreement with the bibliography[44].

1j. *N*,2-diphenylpent-4-enamide

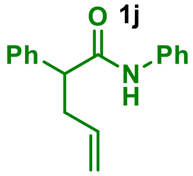

1j

The compound was prepared according to the *GP1* from 2-phenylpent-4-enoic acid (10.5 mmol) purified by flash column chromatography with a mixture of hexane:EtOAc (100:0 to 90:10) yielding 1.5 g (57%) as colorless oil.

Spectrum

^{1}H NMR (400 MHz, $CDCl_3$) δ 7.46 – 7.36 (m, 2H), 7.32 – 7.28 (m, 7H), 7.11 – 7.03 (m, 2H), 5.77 (ddt, *J* = 17.1, 10.2, 6.9 Hz, 1H), 5.09 (dd, *J* = 17.1, 1.6 Hz, 1H), 5.03 – 4.87 (m, 1H), 3.57 (t, J = 7.5 Hz, 1H), 3.07 – 2.95 (m, 1H), 2.60 (dt, J = 14.5, 7.4 Hz, 1H) ppm.
In agreement with the bibliography[Error! Bookmark not defined.].

1k. Allyl phenylcarbamate

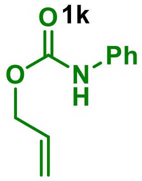

1k

The compound was prepared according to the described procedure[45] from phenyl isocyanate (10 mmol), allyl alcohol (10 mmol) and Et_3N (10 mmol) in dry THF (0.5M) yielding 1.97 g (quantitative) as white solid.

Spectrum

^{1}H NMR (300 MHz, $CDCl_3$) δ 7.48 (d, *J* = 8.0 Hz, 2H), 7.33 (t, *J* = 7.9 Hz, 3H), 7.16 – 7.05 (m, 1H), 6.10 – 5.91 (m, 1H), 5.46 – 5.21 (m, 2H), 4.72 (dd, *J* = 5.5, 1.5 Hz, 2H) ppm.
In agreement with the bibliography[45].

1l. 2-allyl-*N*-phenylbenzamide

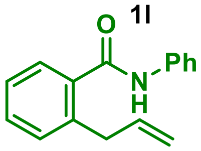

1l

The compound was prepared according to the described procedure[46] from 2-iodo-*N*-phenylbenzamide (1.55 mmol, 1 eq.), allyltributyltin (1.7 mmol), $Pd(PPh_3)_4$ (5 mol%), and dioxane (0.155M) purified by flash column chromatography with a mixture of hexane:EtOAc (100:0 to 80:20) yielding 221.2 mg (60%) as white solid.

Spectrum

^{1}H NMR (400 MHz, $CDCl_3$) δ 7.57 (dd, *J* = 23.0, 7.8 Hz, 4H), 7.47 – 7.25 (m, 5H), 7.19 – 7.08 (m, 1H), 6.08 (ddt, *J* = 16.5, 10.1, 6.2 Hz, 1H), 5.13 (dd, *J* = 10.2, 1.6 Hz, 1H), 5.02 (dd, *J* = 17.1, 1.7 Hz, 1H), 3.62 (dt, *J* = 6.3, 1.7 Hz, 2H) ppm.
In agreement with the bibliography[46].

1m. *N*-phenyl-2-vinylbenzamide

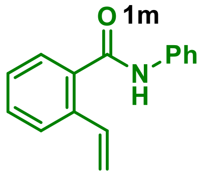

The compound was prepared according to the *GP1* from 2-vinylbenzoic acid (2 mmol) purified by flash column chromatography with a mixture of hexane:EtOAc (100:0 to 80:20) yielding 183.1 mg (41%) as white solid.

Spectrum

^{1}H NMR (300 MHz, $CDCl_3$) δ 7.69 (s, 1H), 7.52 (s, 4H), 7.50 – 7.23 (m, 4H), 7.21 – 7.01 (m, 2H), 5.75 (dd, *J* = 17.5, 1.1 Hz, 1H), 5.38 (dd, *J* = 11.0, 1.1 Hz, 1H) ppm.
In agreement with the bibliography[47].

1n. Hex-5-enamide

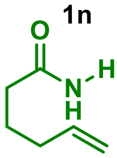

The compound was prepared according to the described procedure[48] from hex-5-enoyl chloride (2 mmol) and ammonia purified by flash column chromatography with a mixture of hexane:EtOAc (100:0 to 70:30) yielding 86 mg (38%) as white solid.

Spectrum

^{1}H NMR (400 MHz, $CDCl_3$) δ 6.04 (s, 1H), 5.76 (ddt, *J* = 16.9, 10.2, 6.7 Hz, 1H), 5.62 (s, 1H), 5.06 – 4.92 (m, 2H), 2.24 – 2.16 (m, 2H), 2.09 (q, *J* = 7.1 Hz, 2H), 1.72 (p, *J* = 7.5 Hz, 2H) ppm.
In agreement with the bibliography[48].

1o. *N*-hydroxyhex-5-enamide

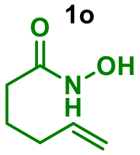

The compound was prepared according to described procedure[49] from hex-5-enoyl chloride (1.51 mmol, 1 eq.), hydroxylamine hydrochloride (1.81 mmol, 1.2 eq.), K_2CO_3 (2 eq.) in a mixture of EtOAc/H_2O 7.5 mL/7.5 mL purified by flash column chromatography with a mixture of hexane:EtOAc (100:0 to 80:20) yielding 132.6 mg (68%) as white solid.

Spectrum

^{1}H NMR (400 MHz, $CDCl_3$) δ 9.25 (bs, 2H), 5.71 (ddt, *J* = 16.9, 10.3, 6.5 Hz, 1H), 5.04 – 4.88 (m, 2H), 2.17 – 1.92 (m, 4H), 1.67 (q, *J* = 7.5 Hz, 2H) ppm.
In agreement with the bibliography[49].

1p. *N*-(tert-butyl)hex-5-enamide

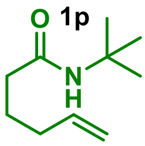

The compound was prepared according to the *GP1* from hex-5-enoic acid (3 mmol) purified by flash column chromatography with a mixture of hexane:EtOAc (100:0 to 90:10) yielding 470 mg (93%) as white solid.

Spectrum

^{1}H NMR (400 MHz, $CDCl_3$) δ 5.78 (ddt, *J* = 17.0, 10.2, 6.8 Hz, 1H), 5.23 (bs, 1H), 5.08 – 4.93 (m, 2H), 2.15 – 2.02 (m, 4H), 1.77 – 1.61 (m, 2H), 1.34 (s, 9H) ppm.
In agreement with the bibliography[50].

1q. (9*H*-fluoren-9-yl)methyl (*S*)-(1-oxo-1-(phenylamino)pent-4-en-2-yl)carbamate

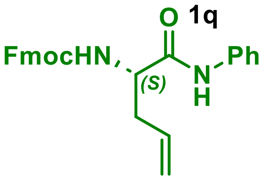

The compound was prepared according to the *GP1* from commercial Fmoc-allyl-Gly-OH (146549-21-5; 1.2 mmol, 1 eq.), purified by flash column chromatography with a mixture of hexane:EtOAc (100:0 to 70:30) yielding 234.4 mg (49%) as white solid.

Spectra

¹H NMR (400 MHz, $CDCl_3$) δ 8.12 (bs, 1H), 7.76 (d, *J* = 7.6 Hz, 2H), 7.56 (dd, *J* = 7.6, 3.5 Hz, 2H), 7.51 – 7.46 (m, 2H), 7.39 (t, *J* = 7.4 Hz, 2H), 7.34 – 7.27 (m, 4H), 7.12 (t, *J* = 7.4 Hz, 1H), 5.81 (bs, 1H), 5.44 (bs, 1H), 5.24 – 5.15 (m, 2H), 4.46 (d, *J* = 7.1 Hz, 2H), 4.38 (bs, 1H), 4.22 (t, *J* = 6.8 Hz, 1H), 2.61 (d, *J* = 8.0 Hz, 2H) ppm.
¹³C NMR (75 MHz, $CDCl_3$) δ 169.3 (C), 143.7 (C), 141.5 (C), 137.5 (C), 132.9 (CH), 129.1 (CH), 128.0 (CH), 127.3 (CH), 125.1 (CH), 124.8 (CH), 120.2 (CH), 120.2 (CH), 119.7 (CH_2), 67.5 (CH_2), 55.1 (CH), 47.3 (CH), 36.7 (CH_2), 34.1 (CH_2), 25.7 (CH_2), 25.1 (CH_2) ppm.
HRMS (ESI) m/z: $[M+Na]^+$ Calcd for $C_{26}H_{24}N_2O_3Na$ 435.1679. Found 435.1680.

*2.2 Aryl iodides (**2**)*

Aryl iodides employed were commercially available.

2a **2b** **2c** **2d** **2e**

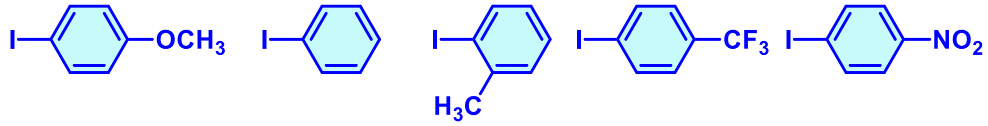

*2.3 Synthesis of vinyl iodides (**4**)*

4a. (*E*)-(2-iodovinyl)benzene

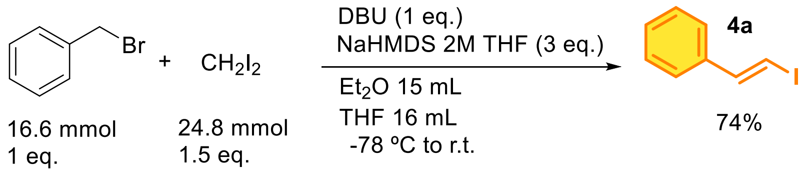

In a flask, a solution of CH_2I_2 (2 mL, 24.8 mmol, 1.5 eq.) in 6 mL THF is added in **dark** to a solution of NaHMDS (2 M THF, 25 mL, 3 eq.) in 15 mL of Et_2O at -78 °C (acetone bath) for 30 min, and then 20 min more stirring at -78 °C. After that, a solution of benzyl bromide (1.97 mL, 16.6 mmol, 1 eq.) in 10 mL THF is added to the mixture at -78 °C. The mixture is stirred for 90 min in **dark** at -78 °C, and then it is warmed up to r.t. for 30 min. DBU (2.5 mL, 1 eq.) is injected dropwise, and the reaction is stirred for 2h at r.t. After that, the mixture is diluted with 100 mL Et_2O and it is filtered through a plug of celite over a silica pad, and it is concentrated. The crude is purified by short FCC eluting with hexane affording **4a** as yellow oil (2.84 g, 74%).

The compound was prepared according to the procedure below[51] yielding 2.84 g (74%) as yellow oil.

4a

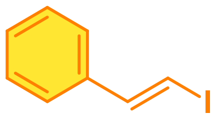

Spectrum

¹H NMR (300 MHz, $CDCl_3$) δ 7.44 (d, *J* = 14.9 Hz, 1H), 7.36 – 7.28 (m, 5H), 6.84 (d, *J* = 14.9 Hz, 1H) ppm.
In agreement with the bibliography[51].

4b. (*E*)-1-iodohex-1-ene

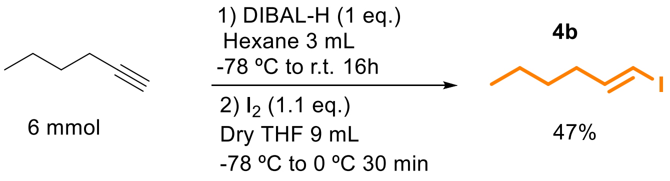

1) In a flask, DIBAL-H (1 M in THF, 6 mL, 1 eq.) is injected dropwise into a solution of heptyne (0.79 mL, 6 mmol, 1 eq.) in 3 mL of hexane at -78 °C (acetone bath). The mixture is stirred for 30 min at -78 °C, and then

16h at r.t. 2) The solvent is removed, and the residue is dissolved in 3 mL of THF and then cold down to -78 °C. After that, a solution of I_2 (1.68 g, 6.6 mmol, 1.1 eq.) in 6 mL THF is injected to the mixture at -78 °C stirring for 30 min. Then, the mixture is warmed up to 0 °C and it is quenched with H_2O and $Na_2S_2O_3$ sol. It is extracted with Et_2O 3 times, dried over $MgSO_4$, filtered and concentrated affording **4b** as yellow oil (633.6 mg, 47.1%).

4b

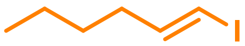

The compound was prepared according to the procedure[52] below yielding 633.6 mg (47.1%) as yellow oil.

Spectrum

^{1}H NMR (400 MHz, $CDCl_3$) δ 6.51 (dt, *J* = 14.3, 7.1 Hz, 1H), 5.97 (dt, *J* = 14.3, 1.5 Hz, 1H), 2.05 (qd, *J* = 7.2, 1.5 Hz, 2H), 1.47 – 1.19 (m, 7H), 0.97 – 0.83 (m, 4H) ppm.
In agreement with the bibliography[52].

*2.4 General procedure 2 (GP2): Synthesis of alkynyl iodides (**6**)*

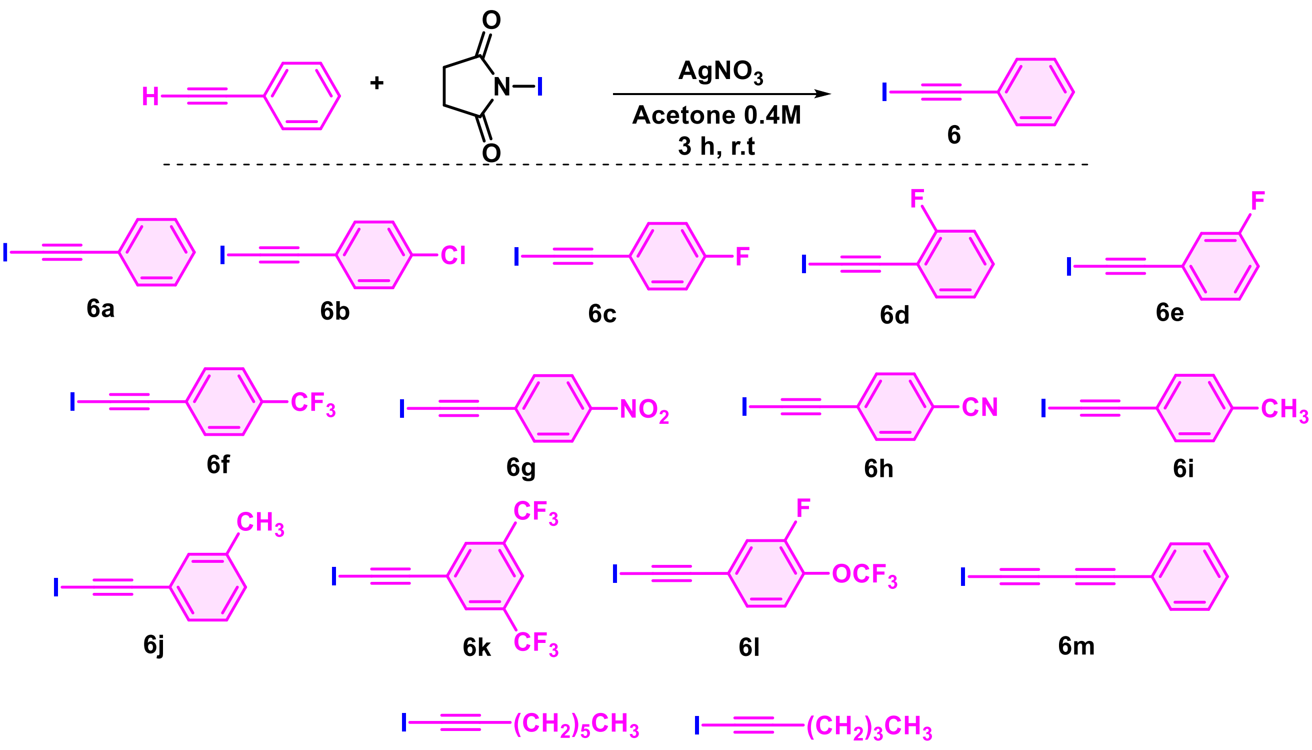

In a dry flask acetylene (2 mmol), iodosuccinimide (2.2 mmol), and silver nitrate (0.2 mmol) are introduced, and acetone (5 mL) is added. The mixture is stirred for 3 h at r.t. Then, the solvent is removed, and the residue is dissolved in hexane and is filtered through celite and purified by flash column chromatography if necessary.

6a. (Iodoethynyl)benzene

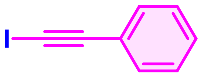

The compound was prepared according to the *GP2* using phenylacetylene (28.8 mmol), iodosuccinimide (31.68 mmol), and silver nitrate (2.9 mmol) in 70 mL of acetone yielding 5.78 g (88%) as yellow oil.

Spectrum

^{1}H NMR (300 MHz, $CDCl_3$) δ 7.47 – 7.41 (m, 2H), 7.31 (dd, *J* = 5.1, 1.9 Hz, 3H) ppm.

In agreement with the bibliography[53].

6b. 1-Chloro-4-(iodoethynyl)benzene

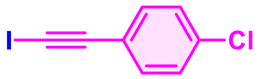

The compound was prepared according to the *GP2* using 1-chloro-4-ethynylbenzene yielding 409.9 mg (88%) as pale-yellow solid.

Spectrum
^{1}H NMR (300 MHz, $CDCl_3$) δ 7.40 – 7.33 (m, 2H), 7.31 – 7.27 (m, 2H) ppm.
In agreement with the bibliography[54].

6c. 1-Fluoro-4-(iodoethynyl)benzene

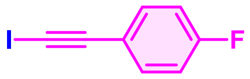

The compound was prepared according to the *GP2* using 1-ethynyl-4-fluorobenzene (16.65 mmol), iodosuccinimide (18.32 mmol), and silver nitrate (1.7 mmol) in 40 mL of acetone yielding 4.06 g (99%) as yellow oil.

Spectra
^{1}H NMR (300 MHz, $CDCl_3$) δ 7.49 – 7.36 (m, 2H), 7.00 (t, *J* = 8.7 Hz, 3H) ppm.
^{19}F NMR (282 MHz, $CDCl_3$) δ -109.71 ppm.
In agreement with the bibliography[53].

6d. 1-Fluoro-2-(iodoethynyl)benzene

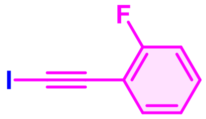

The compound was prepared according to the *GP2* using 1-ethynyl-2-fluorobenzene yielding 487.1 mg (99%) as yellow oil.

Spectra
^{1}H NMR (300 MHz, $CDCl_3$) δ 7.47 – 7.37 (m, 1H), 7.35 – 7.24 (m, 1H), 7.14 – 7.00 (m, 2H) ppm.
^{19}F NMR (282 MHz, $CDCl_3$) δ -110.05 ppm.
In agreement with the bibliography[55].

6e. 1-Fluoro-3-(iodoethynyl)benzene

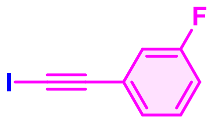

The compound was prepared according to the *GP2* using 1-ethynyl-3-fluorobenzene yielding 286.7 mg (58%) as yellow oil.

Spectra
^{1}H NMR (400 MHz, $CDCl_3$) δ 7.29 (dd, *J* = 7.9, 5.8 Hz, 1H), 7.21 (dt, *J* = 7.7, 1.3 Hz, 1H), 7.13 (ddd, *J* = 9.3, 2.6, 1.4 Hz, 1H), 7.04 (tdd, *J* = 8.4, 2.6, 1.2 Hz, 1H) ppm.
^{19}F NMR (376 MHz, $CDCl_3$) δ -113.55 ppm.
In agreement with the bibliography[53].

6f. 1-(Iodoethynyl)-4-(trifluoromethyl)benzene

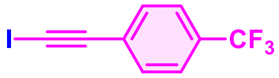

The compound was prepared according to the *GP2* using 1-ethynyl-4-(trifluoromethyl)benzene yielding 443.6 mg (75%) as pale-yellow solid.

Spectra
^{1}H NMR (300 MHz, $CDCl_3$) δ 7.72 – 7.47 (m, 4H) ppm.
^{19}F NMR (282 MHz, $CDCl_3$) δ -62.94 ppm.
In agreement with the bibliography[53].

6g. 1-(Iodoethynyl)-4-nitrobenzene

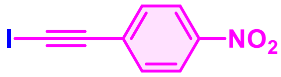

The compound was prepared according to the *GP2* using 1-ethynyl-4-nitrobenzene yielding 482.3 mg (88%) as yellow solid.

Spectrum

^{1}H NMR (300 MHz, $CDCl_3$) δ 8.19 (d, *J* = 8.9 Hz, 2H), 7.58 (d, *J* = 8.9 Hz, 2H) ppm.
In agreement with the bibliography[51].

6h. 4-(Iodoethynyl)benzonitrile

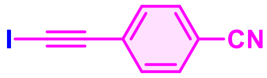

The compound was prepared according to the *GP2* using 4-ethynylbenzonitrile yielding 437.4 mg (86%) as white solid.

Spectrum

^{1}H NMR (300 MHz, $CDCl_3$) δ 7.60 (d, *J* = 8.4 Hz, 2H), 7.51 (d, *J* = 8.5 Hz, 2H) ppm.
In agreement with the bibliography[51].

6i. 1-(Iodoethynyl)-4-methylbenzene

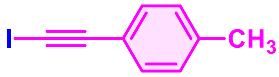

The compound was prepared according to the *GP2* using 1-ethynyl-4-methylbenzene yielding 440.5 mg (91%) as yellow oil.

Spectrum

^{1}H NMR (300 MHz, $CDCl_3$) δ 7.33 (d, *J* = 8.0 Hz, 2H), 7.12 (d, *J* = 8.0 Hz, 2H), 2.36 (s, 3H) ppm.
In agreement with the bibliography[51].

6j. 1-(Iodoethynyl)-3-methylbenzene

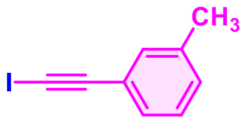

The compound was prepared according to the *GP2* using 1-ethynyl-3-methylbenzene yielding 527.6 mg (quantitative) as colorless oil.

Spectrum

^{1}H NMR (400 MHz, $CDCl_3$) δ 7.25 (d, *J* = 11.8 Hz, 2H), 7.20 (t, *J* = 7.5 Hz, 1H), 7.14 (d, *J* = 7.4 Hz, 1H), 2.32 (s, 3H) ppm.
In agreement with the bibliography[56].

6k. 1-(Iodoethynyl)-3,5-bis(trifluoromethyl)benzene

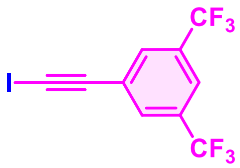

The compound was prepared according to the *GP2* using 1-ethynyl-3,5-bis(trifluoromethyl)benzene (1 mmol), iodosuccinimide (1.1 mmol), and silver nitrate (0.11 mmol) in 2.5 mL of acetone yielding 282.5 mg (78%) as yellow oil.

Spectra

^{1}H NMR (300 MHz, $CDCl_3$) δ 7.87 (m, 2H), 7.82 – 7.80 (m, 1H) ppm.
^{19}F NMR (282 MHz, $CDCl_3$) δ -63.21 ppm.
In agreement with the bibliography[57].

6m. (iodobuta-1,3-diyn-1-yl)benzene

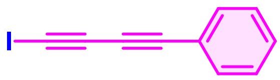

The compound was prepared according to the *GP2* using buta-1,3-diyn-1-ylbenzene (1 mmol), iodosuccinimide (1.1 mmol), and silver nitrate (0.11 mmol) in 2.5 mL of acetone yielding 189.0 mg (75%) as yellow oil.

Spectra

^{1}H NMR (300 MHz, $CDCl_3$) δ 7.87 (m, 2H), 7.82 – 7.80 (m, 1H) ppm.

^{19}F NMR (282 MHz, $CDCl_3$) δ -63.21 ppm.
In agreement with the bibliography[58].

6n. 1-Iodooct-1-yne

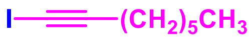

The compound was prepared according to the *GP2* using oct-1-yne (1 mmol), iodosuccinimide (1.1 mmol), and silver nitrate (0.11 mmol) in 2.5 mL of acetone yielding 302 mg (64%) as colourless oil.

Spectrum

^{1}H NMR (300 MHz, $CDCl_3$) δ 2.35 (t, *J* = 7.0 Hz, 2H), 1.57 – 1.45 (m, 2H), 1.44 – 1.22 (m, 6H), 0.89 (t, J = 6.8 Hz, 3H) ppm.
In agreement with the bibliography[59].

6o. 1-Iodohex-1-yne

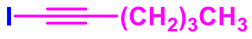

The compound was prepared according to the *GP2* using hex-1-yne, iodosuccinimide (1.1 mmol), and silver nitrate (0.11 mmol) in 2.5 mL of acetone yielding yielding 633.6 mg (47%) as colorless oil.

Spectrum

^{1}H NMR (400 MHz, $CDCl_3$) δ 2.36 (t, *J* = 7.0 Hz, 2H), 1.56 – 1.33 (m, 4H), 0.91 (t, *J* = 7.2 Hz, 3H) ppm.
In agreement with the bibliography[60].

2.5 Alternative synthesis of alkynyl iodides

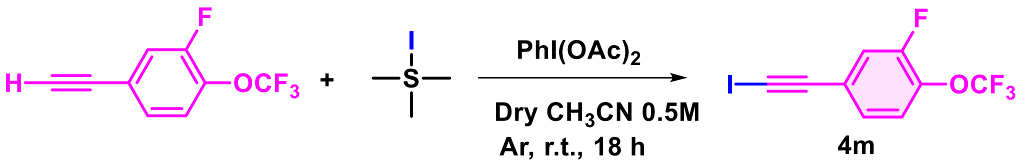

Acetylene (1 mmol) is added to a solution of Me_3SI (1.1 mmol) and $PhI(OAc)_2$ (1.1 mmol) in dry MeCN 0.5M (2 mL) at r.t. under Ar atm. Then, the mixture is stirred for 16 h at r.t. After that, the reaction is diluted with EtOAc (5 mL), quenched with sat. $NaHCO_3$ (2.5 mL), sat. $Na_2S_2O_3$ (11 mL) and extracted with EtOAc (3 x 15 mL). Afterward, it is washed with Brine, dried over anhydrous $MgSO_4$, filtered, and concentrated to reach the final compound.

6l. 2-Fluoro-4-(iodoethynyl)-1-(trifluoromethoxy)benzene

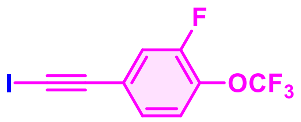

The compound was prepared as described above using 4-ethynyl-2-fluoro-1-(trifluoromethoxy)benzene (1 mmol) yielding 308 mg (93%) as yellow oil.

Spectra

^{1}H NMR (300 MHz, $CDCl_3$) δ 7.38 – 7.17 (m, 3H) ppm.
^{19}F NMR (282 MHz, $CDCl_3$) δ -58.68 (d, *J* = 4.8 Hz), -127.95 (q, *J* = 4.8 Hz) ppm.
In agreement with the bibliography.[29]

2.6 Synthesis of gold(I) catalysts

Au1, **Au4** & **Au5** were synthesized following the described bibliography.[61] In a dry flask the ligand (Ligand; Medalphos, 0.6 mmol) and $Me_2S{\cdot}AuCl$ (0.6 mmol) are dissolved in 2.5 mL of dry CH_2Cl_2 under Ar atm. The mixture is stirred in dark conditions for 2h at r.t. Then, it is filtered through a celite plug with CH_2Cl_2. It is concentrated under vacuum atmosphere without heating. After that, the white solid is recrystallized with the minimal amount of CH_2Cl_2 layered hexanes.

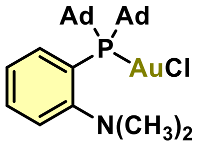

Au1
MeDalphosAuCl

MeDalphosAuCl (**Au1**) was synthesized following the procedure described above yielding 345.5 mg (89%). In agreement with the literature[61].

Spectra

^{1}H NMR (300 MHz, $CDCl_3$) δ 7.73 (t, J = 7.4 Hz, 1H), 7.53 (t, J = 5.8 Hz, 2H), 7.37 – 7.22 (m, 1H), 2.58 (s, 6H), 2.26 – 2.13 (m, 7H), 2.10 – 2.04 (m, 5H), 1.97 (s, 6H), 1.66 (s, 12H) ppm.
^{31}P NMR (122 MHz, $CDCl_3$) δ 56.55 ppm.

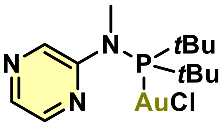

Au4
KarphosAuCl

KarphosAuCl (**Au4**) was synthesized following the procedure described above (on 0.1975 mmol) yielding 83.7 mg (87%). In agreement with the literature[37].

Spectrum

^{1}H NMR (400 MHz, $CDCl_3$) δ 8.48 (s, 1H), 8.23 (d, J = 18.4 Hz, 2H), 3.61 (d, J = 8.3 Hz, 3H), 1.49 (d, J = 16.9 Hz, 18H) ppm.

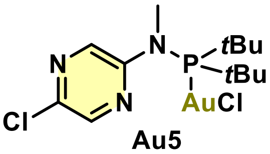

Au5
ClKarphosAuCl

ClKarphosAuCl (**Au5**)was synthesized following the procedure described above (on 0.521 mmol) yielding 255.8 mg (94%). In agreement with the literature[37].

Spectra

^{1}H NMR (300 MHz, $CDCl_3$) δ 8.22 (d, J = 9.0 Hz, 2H), 3.56 (d, J = 7.6 Hz, 3H), 1.48 (d, J = 17.0 Hz, 18H) ppm.
^{31}P NMR (122 MHz, $CDCl_3$) δ 119.46 ppm.

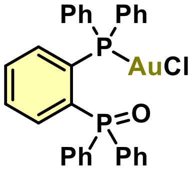

Au6
Au(dppbO)Cl

Au(dppbO)Cl (**Au6**) was synthesized following the reported procedure[62] in the literature. In a dry flask 1,2-bis(diphenylphosphino)benzene (0.2 mmol) and $NaAuCl_4{\cdot}2H_2O$ (0.194 mmol) are dissolved in 3.3 mL of $CH_3CN{:}H_2O$ (10:1) mixture. The reaction is refluxed for 8 hours. Then, the solvent is removed, and the residue is recrystallized in hot MeOH yielding a pale-yellow solid (60 mg, 43 %).

Spectra

^{1}H NMR (400 MHz, $CDCl_3$) δ 7.58 – 7.34 (m, 24H) ppm.
^{31}P NMR (162 MHz, $CDCl_3$) δ 34.05 (d, J = 5.9 Hz), 31.73 (d, J = 5.9 Hz) ppm.

MeCagephosAuCl (Au2)

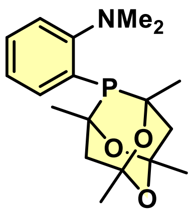

MeCagephos

MeCagephosAuCl (**Au2**) was synthesized firstly from the corresponding 2-Br-*N*,*N*,-dimethylaniline and PHcage (1,3,5,7-tetramethyl-2,4,8-trioxa-6-phospha-adamantane) using the described procedure[63], and then the insertion of gold by the procedure described above.[61] In a flask, 2-Br-*N*,*N*-dimethylaniline (2.0 g), PHcage (2.1 g), $Pd(PPh_3)_4$ (250 mg, 0.24 mmol) and potassium carbonate (2.2 g, 16.2 mmol) are dissolved with xylenes (20 mL) under an atmosphere of argon. The mixture is heated at 110 °C (oil bath) for 3 days. After that, the reaction is diluted with diethyl ether, filtered through a silica plug, concentrated under vacuum, and purified by FCC with Hexane:EtOAc (9:1).

Spectra

^{1}H NMR (400 MHz, $CDCl_3$) δ 8.11 (dt, *J* = 7.7, 1.6 Hz, 1H), 7.31 (td, *J* = 8.0, 1.5 Hz, 1H), 7.20 – 7.13 (m, 1H), 7.09 (td, *J* = 7.7, 1.1 Hz, 1H), 2.70 (s, 6H), 2.13 (dd, *J* = 13.2, 7.2 Hz, 1H), 1.92 (dd, *J* = 24.8, 13.2 Hz, 1H), 1.77 (d, *J* = 13.3 Hz, 1H), 1.51 (d, *J* = 12.2 Hz, 3H), 1.45 – 1.34 (m, 6H), 1.29 (d, *J* = 11.2 Hz, 3H) ppm.
^{31}P NMR (162 MHz, $CDCl_3$) δ -36.14 ppm.
In agreement with the literature.[63]

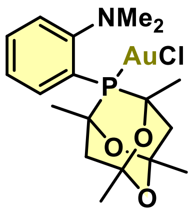

Au2
MeCagephosAuCl

MeCagephosAuCl (**Au2**) (CCDC number: 2534753) was synthesized following the procedure described above (on 0.5 mmol) yielding 252.7 mg (89%). It is crystallised using a minimum amount of CH_2Cl_2 and layered hexane.

Spectra
^{1}H NMR (400 MHz, $CDCl_3$) δ 8.21 (td, *J* = 8.0, 1.3 Hz, 1H), 7.56 (t, *J* = 7.6 Hz, 1H), 7.46 (dd, *J* = 7.1, 5.2 Hz, 1H), 7.33 – 7.24 (m, 1H), 2.80 (dd, *J* = 13.7, 5.5 Hz, 1H), 2.68 (s, 6H), 1.96 – 1.73 (m, 3H), 1.53 (d, *J* = 14.4 Hz, 2H), 1.41 (d, *J* = 2.3 Hz, 9H) ppm.
^{31}P NMR (162 MHz, $CDCl_3$) δ 8.66 ppm.
^{13}C NMR (101 MHz, $CDCl_3$) δ 161.5 (C, d, *J* = 8.6 Hz), 133.8 (C, t, *J* = 2.1 Hz), 126.5 (CH, d, *J* = 7.5 Hz), 125.4 (CH, d, *J* = 5.7 Hz), 122.0 (CH, d, *J* = 46.8 Hz), 97.0 (CH), 96.26 (C, d, *J* = 1.5 Hz), 75.19 – 72.57 (C, m), 47.83, 46.0 (CH_3, d, *J* = 11.5 Hz), 45.9 (CH_3), 37.1 (CH_2, d, *J* = 2.4 Hz), 27.6 (CH_3 ,d, *J* = 7.9 Hz), 27.5 (CH_2, d, *J* = 34.3 Hz), 25.60 (CH_3, d, *J* = 4.8 Hz) ppm.
HRMS (ESI) m/z: $[M+H]^+$ Calcd for $C_{18}H_{26}AuClNO_3PH$ 568.1077. Found 568.1061

3. Optimization

3.1 Optimization of δ-Valerolactone coupled (3aa) with 4-iodoanysole

Temperature

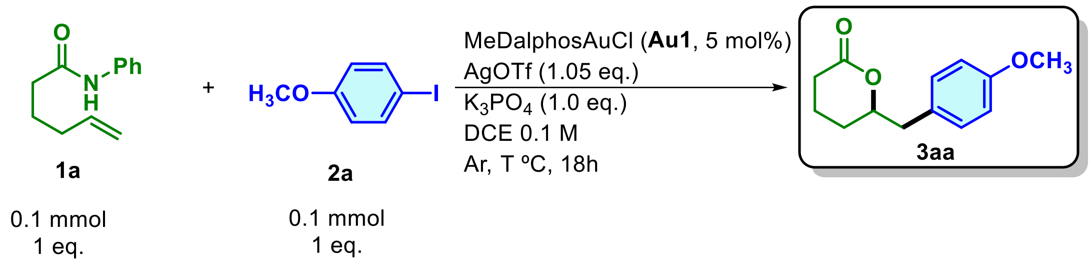

Table S1. Optimization of the reaction temperature.

Entry	T / °C	Conv. **1a** / %	Yield **3aa** / %
1	**r.t.**	48	3
2	**30**	73	12
3	**40**	71	45
4	**50**	99	42
5	**70**	84	80
**6**	**80**	**95**	**85**
7	**90**	87	80

n.d. not detected

Concentration

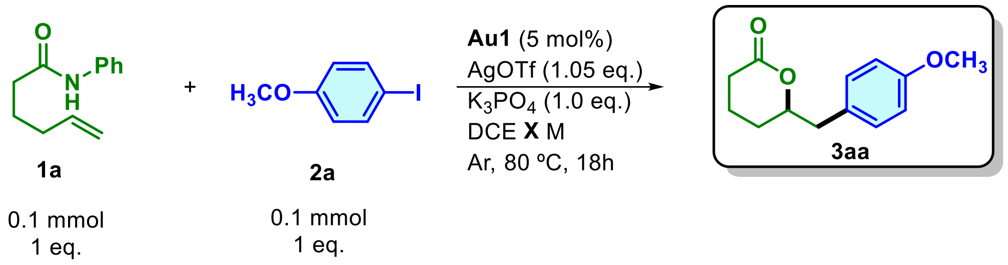

Table S2. Optimization of the reaction concentration.

Entry	[DCE] / M	Conv. **1a** / %	Yield **3aa** / %
8	**0.05**	83	71
**6**	**0.1**	**95**	**85**
9	**0.2**	80	71

n.d. not detected

Base screening

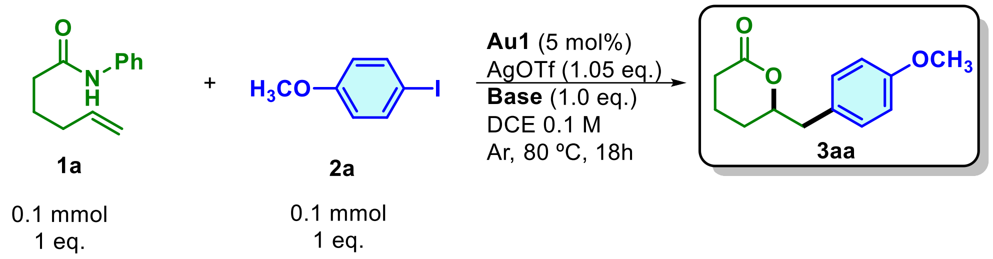

Table S3. Screening of the base.

Entry	**Base / 1 eq.**	Conv. **1a** / %	Yield **3aa** / %
6	**K_3PO_4**	95	85
10	**K_2HPO_4**	70	49
11	**KH_2PO_4**	100	87
12	$NaHCO_3$	95	92
13	**K_2CO_3**	100	75
14	**Et_3N**	41	40
15	**2,2'-Bipyridine**	73	39
16	**NaO*t*Bu**	33	20

K_3PO_4: potassium phosphate; K_2HPO_4: dipotassium hydrogen phosphate; KH_2PO_4: monopotassium dihydrogen phosphate; $NaHCO_3$: sodium hydrogen carbonate; K_2CO_3: potassium carbonate; Et_3N: triethylamine; NaO*t*Bu: sodium tert-butoxide. n.d. not detected

Halide Scavenger screening

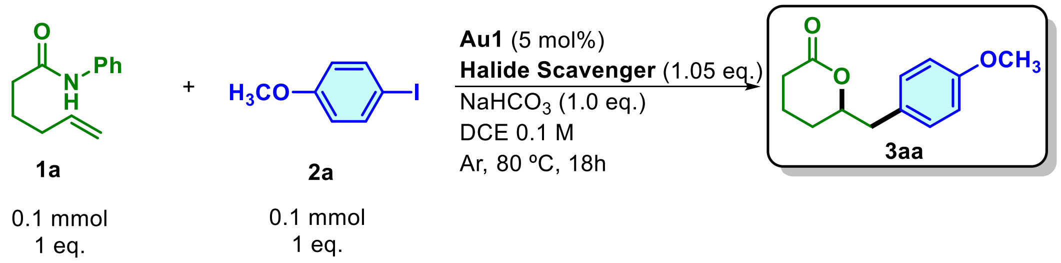

Table S4. Screening of the halide scavenger.

Entry	**Halide scavenger / 1.05 eq**	Conv. **1a** / %	Yield **3aa** / %
12	AgOTf	95	92
17	**$AgSbF_6$**	51	35
18	**$AgNTf_2$**	97	63
19	**$AgBF_4$**	100	73
20	**AgOTs**	19	19

AgOTf: silver (I) triflate; $AgSbF_6$: silver (I) hexafluoroantimonate; $AgNTf_2$: silver (I) bis(trifluoromethanesulfonyl)imide; $AgBF_4$: silver (I) tetrafluoroborate; AgOTs: silver (I) tosylate.

Solvent screening

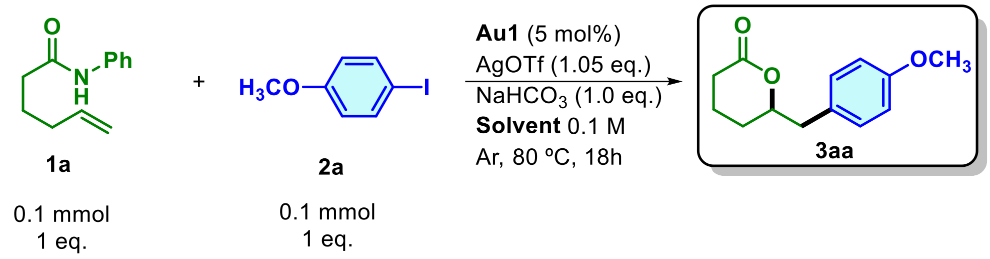

Table S5. Screening of the reaction solvent.

Entry	**Solvent / 0.1 M**	Conv. **1a** / %	Yield **3aa** / %
**12**	**DCE**	**95**	**92**
21	**DCM**	100	85
22	**MeCN**	0	n.d.
23	**DMF**	9	n.d.
24	**Acetone**	24	n.d.
25	**Toluene**	82	79
26	**Pyridine**	3	n.d.
27	**PhCl**	92	65
28	**o-Cl_2Ph**	96	66
29	**MeOH**	9	n.d.
30	**THF**	48	18

DCE: 1,2-dichloroethane; DCM: dichloromethane; CH_3CN: acetonitrile; DMF: dimethylformamide; PhCl: chlorobenzene; o-Cl_2Ph: 1,2-dichlorobenzene; MeOH: methanol; THF: tetrahydrofuran; n.d. not detected

Gold screening

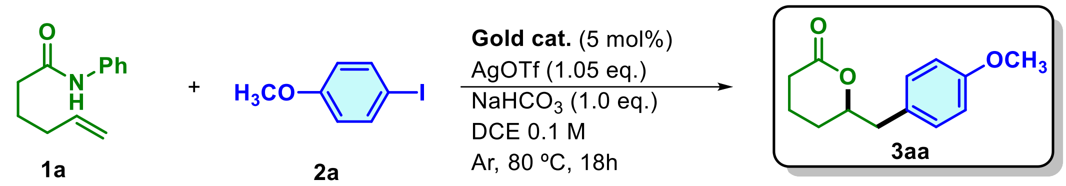

1a 0.1 mmol 1 eq. **2a** 0.1 mmol 1 eq.

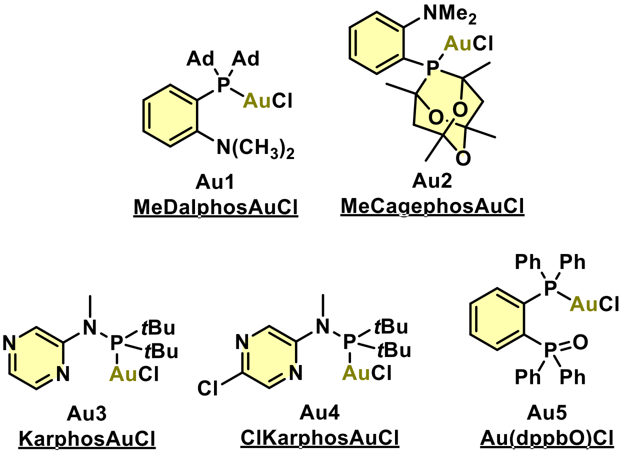

Table S6. Screening of gold catalyst.

Entry	**Au / 5 mol%**	Conv. **1a** / %	Yield **3aa** / %
**12**	**Au1**	**95**	**83**
31	**Au2**	89	76
32	**Au3**	39	24
33	**Au4**	70	70
34	**Au5**	32	3

Control experiments

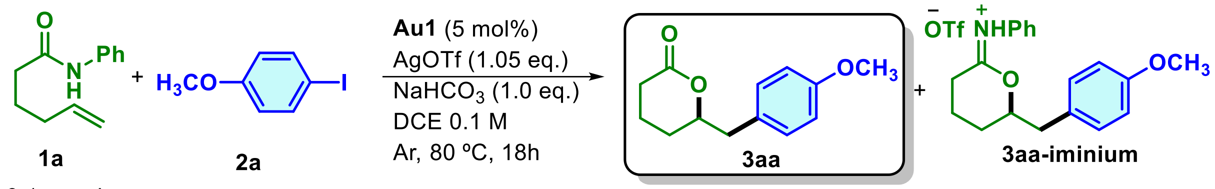

1a 0.1 mmol 1 eq. **2a** 0.1 mmol 1 eq.

Table S7. Control tests with optimal reaction conditions.

Entry	Atm	Au	Scavenger	Base	Conv. **1a** / %	Yield **3aa** / %	Yield **3aa-iminium** / %
**12**	**Ar**	**Au1**	**AgOTf**	**$NaHCO_3$**	**95**	**92**	**n.d.**
35	**Air**	Au1	AgOTf	$NaHCO_3$	98	82	n.d.
36[a]	**Ar**	Au1	AgOTf	$NaHCO_3$	95	92	n.d.
37	Ar	-	AgOTf	$NaHCO_3$	23	n.d.	n.d.
38	Ar	Au1	-	$NaHCO_3$	8	n.d.	n.d.
39	Ar	Au1	AgOTf	-	94	9	83

[a]*moist* DCE, directly from the bottle. n.d. not detected

Base amount

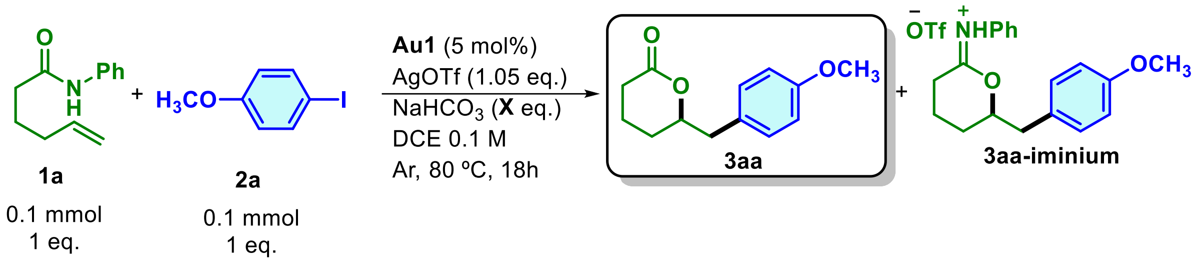

Table S8. Optimization of sodium hydrogenphosphate amount.

Entry	**$NaHCO_3$ / eq.**	Conv. **1a** / %	Yield **3aa** / %	Yield **3aa-iminium** / %
40	**0**	94	9	83
41	**0.5**	100	17	74
**12**	**1.0**	**95**	**92**	**n.d.**
42	**2.0**	100	92.7	n.d.

n.d. not detected

Silver amount

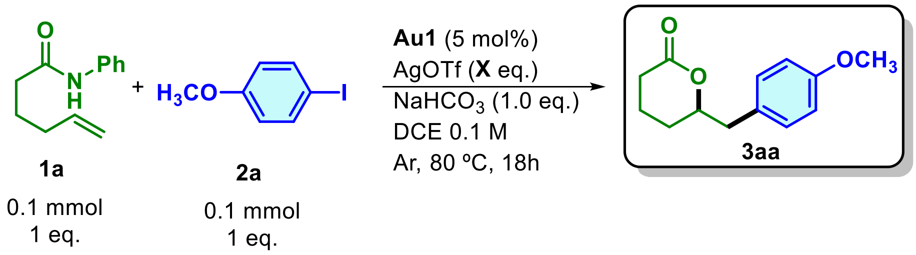

Table S9. Optimization of the silver triflate amount.

Entry	**AgOTf / eq.**	Conv. **1a** / %	Yield **3aa** / %
43	**0.55**	53	37
**12**	**1.05**	**95**	**92**
44	**2.05**	100	94

Gold amount

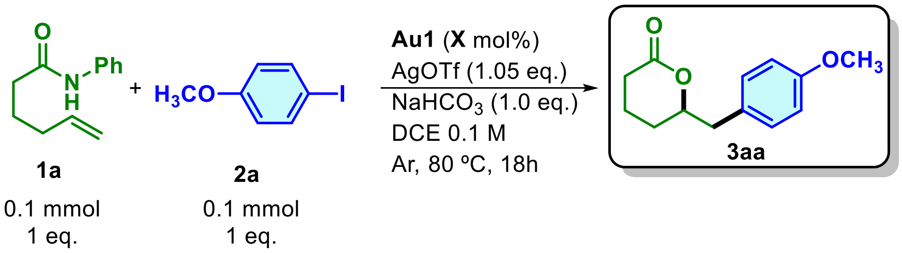

Table S10. Optimization of the gold amount.

Entry	**Au1 / mol%**	Conv. **1a** / %	Yield **3aa** / %
45	**2.5**	91	57
**12**	**5.0**	**95**	**92**
46	**10.0**	100	94

3.2 Optimization of δ-Valerolactone coupled (5aa) with (E)-(2-iodovinyl)benzene

Temperature

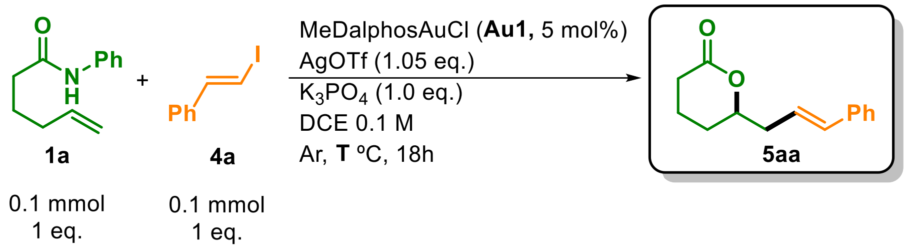

Table S11. Optimization of the reaction temperature.

Entry	**T / °C**	Conv. **1a** / %	Yield **5aa** / %
47	**r.t.**	32	29
48	**80**	66	50
49	**85**	57	47
**50**	**90**	**88**	**68**
51	**95**	85	55
52	**100**	85	49

Concentration

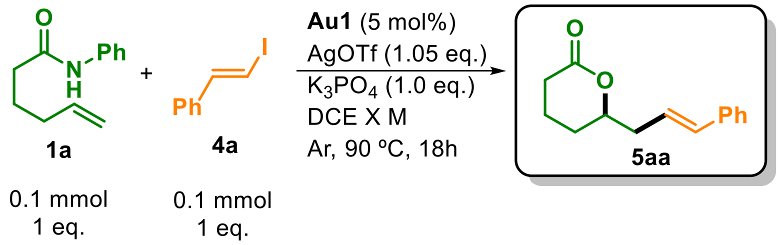

Table S12. Optimization of the reaction concentration.

Entry	**[DCE] / M**	Conv. **1a** / %	Yield **5aa** / %
53	**0.05**	78	67
**50**	**0.1**	**88**	**68**
54	**0.2**	66	49
55	**0.3**	58	41

Base screening

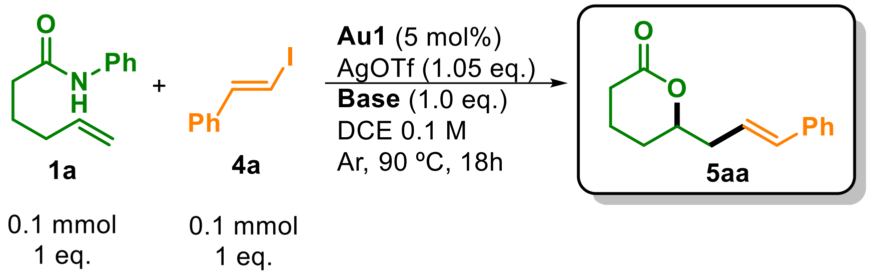

Table S13. Screening of the base.

Entry	**Base / 1 eq.**	Conv. **1a** / %	Yield **5aa** / %
50	$\mathbf{K_3PO_4}$	88	68
56	$\mathbf{K_2HPO_4}$	80	46
57	$\mathbf{KH_2PO_4}$	84	56
58	$\mathbf{NaHCO_3}$	98	84
59	$\mathbf{K_2CO_3}$	90	71
60	$\mathbf{Et_3N}$	78	47
61	**2,2'-Bipyridine**	52	45
62	**NaO*t*Bu**	36	14

K_3PO_4: potassium phosphate; K_2HPO_4: dipotassium hydrogen phosphate; KH_2PO_4: monopotassium dihydrogen phosphate; $NaHCO_3$: sodium hydrogen carbonate; K_2CO_3: potassium carbonate; Et_3N: triethylamine; NaO*t*Bu: sodium tert-butoxide. n.d. not detected

Halide Scavenger screening

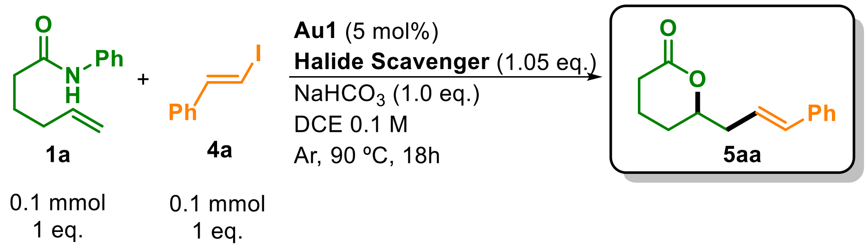

Table S14. Screening of the halide scavenger.

Entry	**Halide scavenger / 1.05 eq**	Conv. **1a** / %	Yield **5aa** / %
58	**AgOTf**	98	84
63	$\mathbf{AgSbF_6}$	84	57
64	$\mathbf{AgNTf_2}$	100	70
65	$\mathbf{AgBF_4}$	64	46
66	**AgOTs**	36	19

AgOTf: silver (I) triflate; $AgSbF_6$: silver (I) hexafluoroantimonate; $AgNTf_2$: silver (I) bis(trifluoromethanesulfonyl)imide; $AgBF_4$: silver (I) tetrafluoroborate; AgOTs: silver (I) tosylate.

Table of contents

Solvent screening

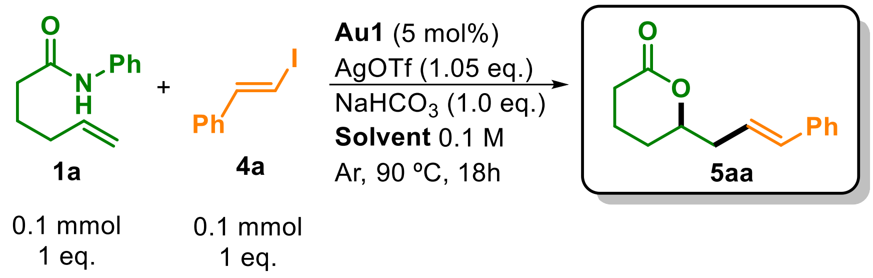

Table S15. Screening of the reaction solvent.

Entry	**Solvent / 0.1 M**	Conv. **1a** / %	Yield **5aa** / %
**58**	**DCE**	**98**	**84**
67	**DCM**	95	70
68	**MeCN**	25	6
69	**DMF**	11	n.d.
70	**Acetone**	44	29
71	**Toluene**	82	66
72	**Pyridine**	11	n.d.
73	**PhCl**	91	69
74	**o-Cl_2Ph**	73	69
75	**MeOH**	43	5
76	**THF**	36	22

DCE: 1,2-dichloroethane; DCM: dichloromethane; CH_3CN: acetonitrile; DMF: dimethylformamide; PhCl: chlorobenzene; o-Cl_2Ph: 1,2-dichlorobenzene; MeOH: methanol; THF: tetrahydrofuran; n.d. not detected

Gold screening

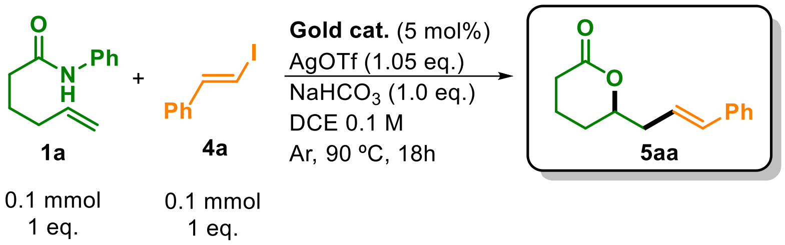

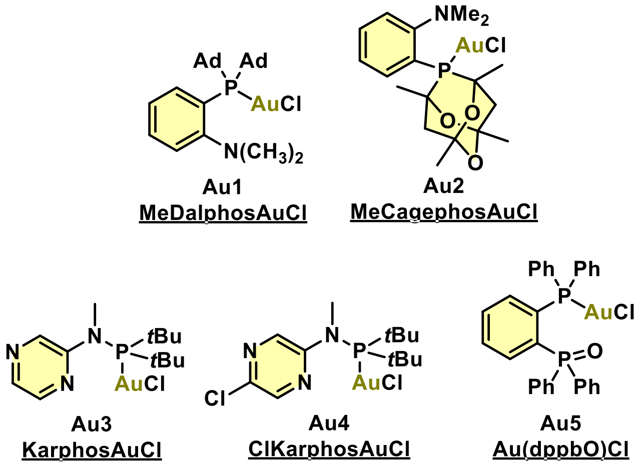

Table S16. Screening of gold catalyst.

Entry	**Au / 5 mol%**	Conv. **1a** / %	Yield **5aa** / %
**58**	**Au1**	**98**	**84**
77	**Au2**	68	47
78	**Au3**	87	64
79	**Au4**	100	66
80	**Au5**	39	n.d.

Base amount

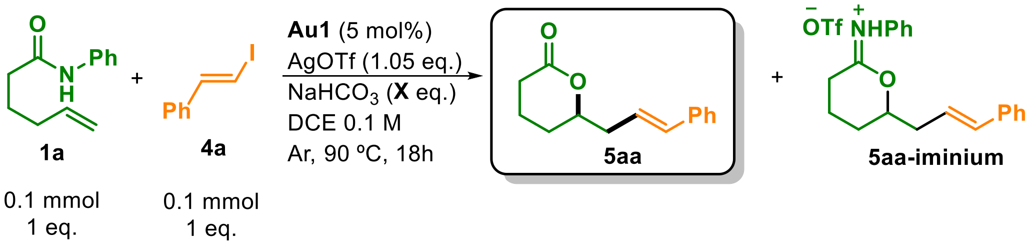

Table S17. Optimization of sodium hydrogenphosphate amount.

Entry	**$NaHCO_3$ / eq.**	Conv. **1a** / %	Yield **5aa** / %	Yield **5aa-iminium** / %
81	**0**	**100**	**n.d.**	**83.2**
82	**0.5**	82	57	n.d.
**58**	**1.0**	**98**	**84**	**n.d.**
83	**2.0**	83	75	n.d.

n.d. not detected

Silver amount

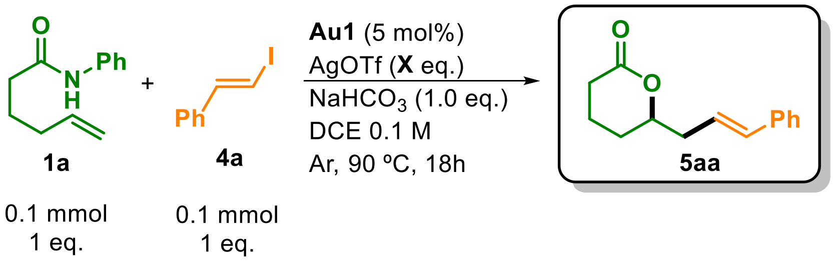

Table S18. Optimization of the silver triflate amount.

Entry	**AgOTf / eq.**	Conv. **1a** / %	Yield **5aa** / %
84	**0.55**	44	34
**58**	**1.05**	**98**	**84**
85	**2.05**	100	80

Gold amount

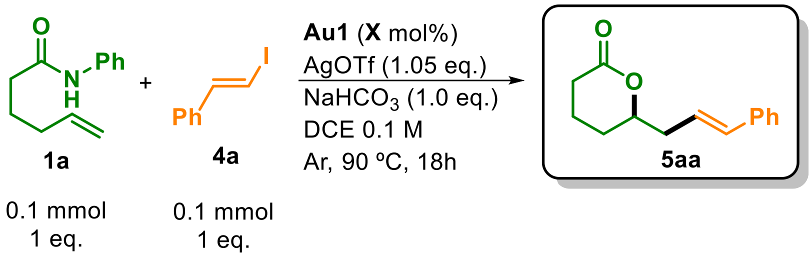

Table S19. Optimization of the gold amount.

Entry	**Au1 / mol%**	Conv. **1a** / %	Yield **5aa** / %
86	**2.5**	93	55
**58**	**5.0**	**98**	**84**
87	**10.0**	100	82

Control experiments

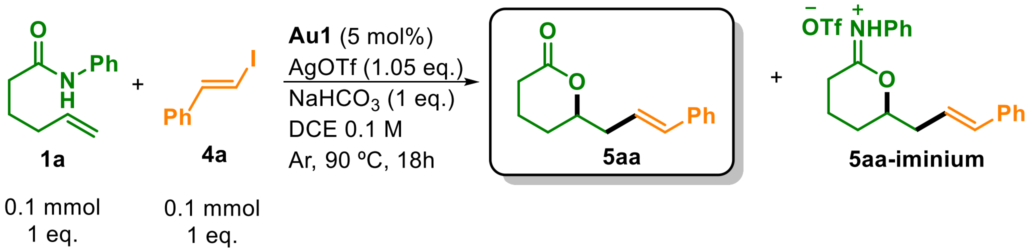

Table S20. Control tests with optimal reaction conditions.

Entry	Atm	Au	Scavenger	Base	Conv. **1a** / %	Yield **5aa** / %	Yield **5aa-iminium** / %
**58**	**Ar**	**Au1**	**AgOTf**	**$NaHCO_3$**	**98**	**84**	**n.d.**
88	**Air**	Au1	AgOTf	$NaHCO_3$	100	82	n.d.
89[a]	**Ar**	Au1	AgOTf	$NaHCO_3$	100	83	n.d.
90	Ar	-	AgOTf	$NaHCO_3$	23	n.d.	n.d.
91	Ar	Au1	-	$NaHCO_3$	8	n.d.	n.d.
92	Ar	Au1	AgOTf	-	100	n.d.	83

[a]*moist* DCE, directly from the bottle. n.d. not detected

Table of contents

3.3 Optimization of δ-Valerolactone coupled with (iodoethynyl)benzene (7aa)

Temperature

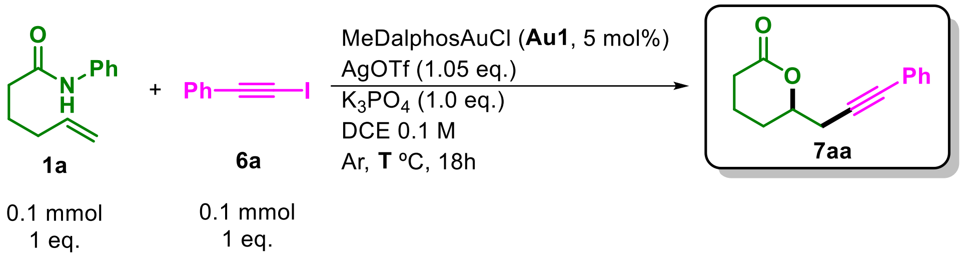

Table S21. Optimization of the reaction temperature and time.

Entry	T / °C	Conv. **1a** / %	Yield **7aa** / %
93	**r.t.**	62	4
94	**30**	32	11
95	**40**	69	31
**96**	**45**	**65**	**42**
97	**50**	61	18
98	**80**	72	20

n.d. not detected

Concentration

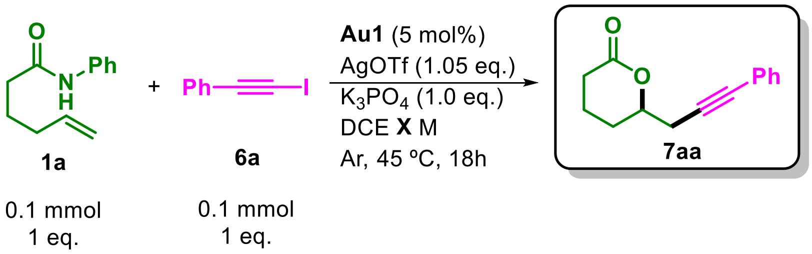

Table S22. Optimization of the reaction concentration.

Entry	[DCE] / M	Conv. **1a** / %	Yield **7aa** / %
99	**0.05**	71	19
**96**	**0.1**	**65**	**42**
100	**0.2**	71	30
101	**0.3**	62	20

Base screening

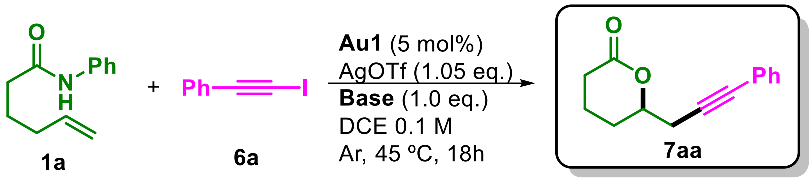

1a **6a**

0.1 mmol 1 eq. 0.1 mmol 1 eq.

Table S23. Screening of the base.

Entry	**Base / 1 eq.**	Conv. **1a** / %	Yield **7aa** / %
96	**K_3PO_4**	65	42
102	**K_2HPO_4**	38	15
103	**KH_2PO_4**	60	13
104	$NaHCO_3$	81	62
105	**K_2CO_3**	59	40
106	**Et_3N**	34	21
107	**2,2'-Bipyridine**	24	13
108	**NaO*t*Bu**	11	n.d.

K_3PO_4: potassium phosphate; K_2HPO_4: dipotassium hydrogen phosphate; KH_2PO_4: monopotassium dihydrogen phosphate; $NaHCO_3$: sodium hydrogen carbonate; K_2CO_3: potassium carbonate; Et_3N: triethylamine; NaO*t*Bu: sodium tert-butoxide. n.d. not detected

Halide Scavenger screening

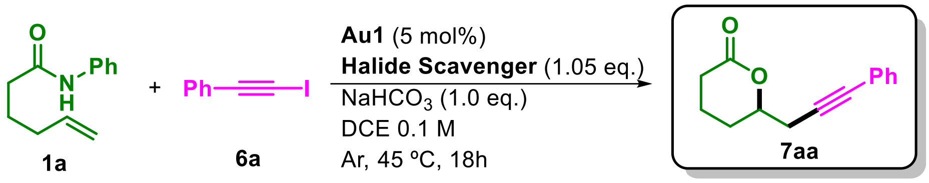

1a **6a**

0.1 mmol 1 eq. 0.1 mmol 1 eq.

Table S24. Screening of the halide scavenger.

Entry	**Halide scavenger / 1.05 eq**	Conv. **1a** / %	Yield **7aa** / %
104	AgOTf	81	62
109	**$AgSbF_6$**	32	9
110	**$AgNTf_2$**	43	16
111	**$AgBF_4$**	54	17
112	**AgOTs**	21	3

AgOTf: silver (I) triflate; $AgSbF_6$: silver (I) hexafluoroantimonate; $AgNTf_2$: silver (I) bis(trifluoromethanesulfonyl)imide; $AgBF_4$: silver (I) tetrafluoroborate; AgOTs: silver (I) tosylate.

Solvent screening

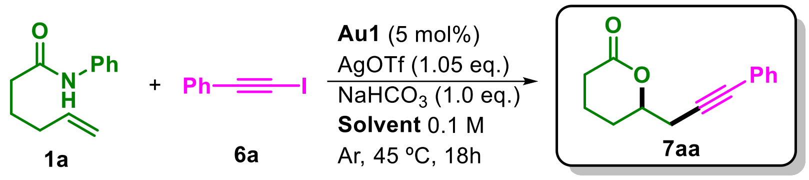

0.1 mmol
1 eq.

0.1 mmol
1 eq.

Table S25. Screening of the reaction solvent.

Entry	**Solvent / 0.1 M**	Conv. **1a** / %	Yield **7aa** / %
**104**	**DCE**	**81**	**62**
113	**DCM**	46	26
114	**MeCN**	0	n.d.
115	**DMF**	8	n.d.
116	**Acetone**	0	n.d.
117	**Toluene**	80	17
118	**Pyridine**	18	n.d.
119	**PhCl**	64	25
120	**o-Cl_2Ph**	75	22
121	**MeOH**	44	n.d.
122	**THF**	45	6

DCE: 1,2-dichloroethane; DCM: dichloromethane; CH_3CN: acetonitrile; DMF: dimethylformamide; PhCl: chlorobenzene; o-Cl_2Ph: 1,2-dichlorobenzene; MeOH: methanol; THF: tetrahydrofuran; n.d. not detected

Table of contents

Gold screening

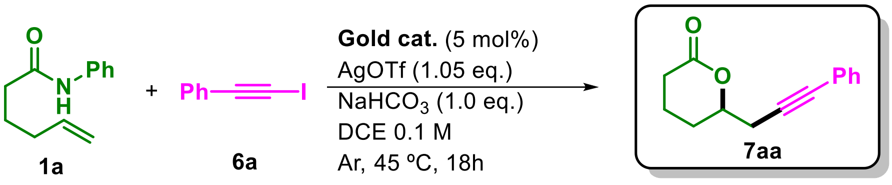

1a 0.1 mmol 1 eq. | **6a** 0.1 mmol 1 eq.

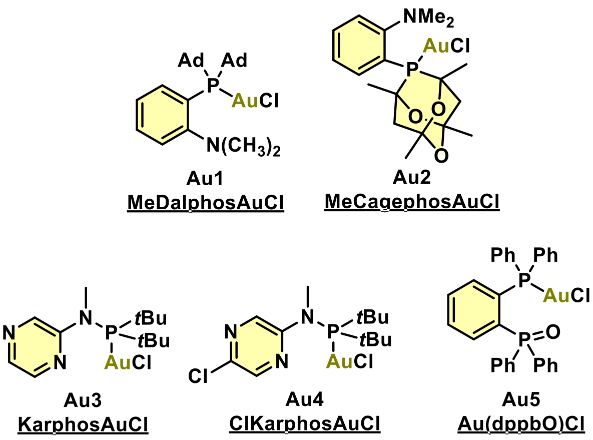

Table S26. Screening of gold catalyst.

Entry	**Au / 5 mol%**	Conv. **1a** / %	Yield **7aa** / %
104	Au1	81	62
123	**Au2**	68	57
124	**Au3**	40	6
125	**Au4**	56	12
126	**Au5**	38	n.d.

Base amount

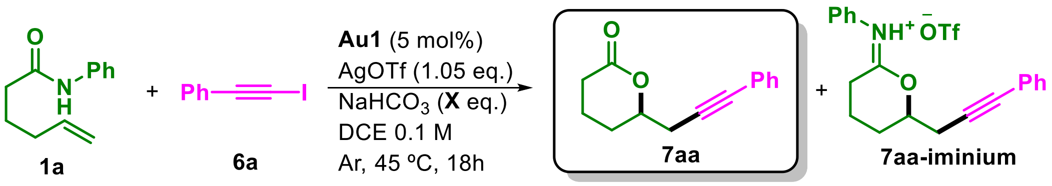

1a 0.1 mmol 1 eq. | **6a** 0.1 mmol 1 eq.

Table S27. Optimization of sodium hydrogenphosphate amount.

Entry	**$NaHCO_3$ / eq.**	Conv. **1a** / %	Yield **7aa** / %	Yield **7aa-iminium** / %
127	**0**	69	7	32
128	**0.5**	67	46	n.d.
104	1.0	81	62	n.d.
129	**2.0**	70	61	n.d.

n.d. not detected

Silver amount

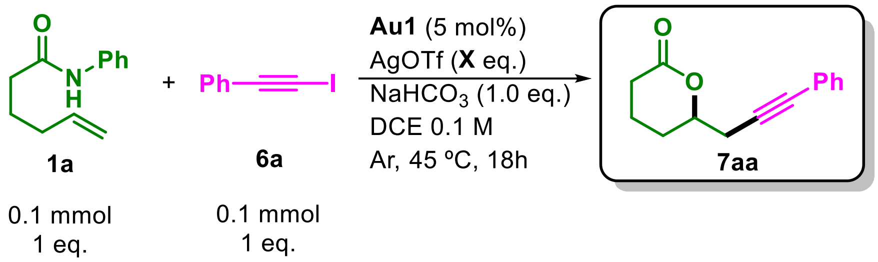

Table S28. Optimization of the silver triflate amount.

Entry	**AgOTf / eq.**	Conv. **1a** / %	Yield **7aa** / %
130	**0.55**	53	25
**104**	**1.05**	**81**	**62**
131	**2.05**	100	66

Gold amount

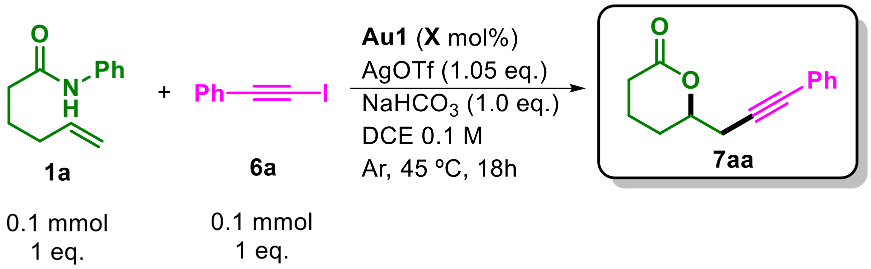

Table S29. Optimization of the gold amount.

Entry	**Au1 / mol%**	Conv. **1a** / %	Yield **7aa** / %
132	**2.5**	43	21
**104**	**5.0**	**81**	**62**
133	**10.0**	95	65

Control experiments

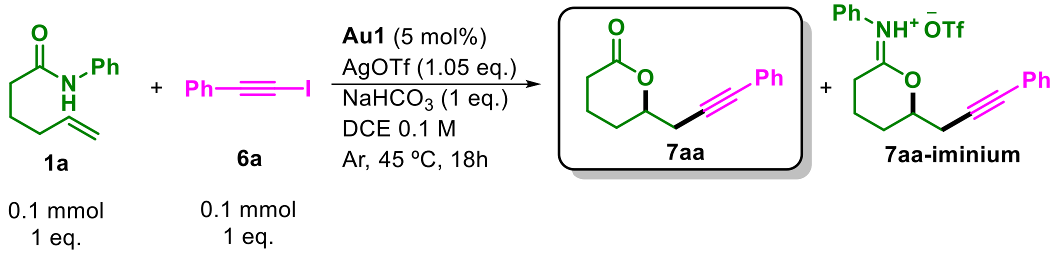

Table S30. Control tests with optimal reaction conditions.

Entry	Atm	**Au**	Scavenger	Base	Conv. **1a** / %	Yield **7aa** / %	Yield **7aa-iminium** / %
**104**	**Ar**	**Au1**	**AgOTf**	**$NaHCO_3$**	**81**	**62**	**n.d.**
134	**Air**	Au1	AgOTf	$NaHCO_3$	72	60	n.d.
135[a]	**Ar**	Au1	AgOTf	$NaHCO_3$	66	n.d.	60
136	Ar	-	AgOTf	$NaHCO_3$	n.d.	n.d.	n.d.
137	Ar	Au1	-	$NaHCO_3$	n.d.	n.d.	n.d.
127	Ar	Au1	AgOTf	-	69	7	32

[a]*moist* DCE, directly from the bottle. n.d. not detected

Table of contents

*Kinetics for **3a**, **5a** and **7a***

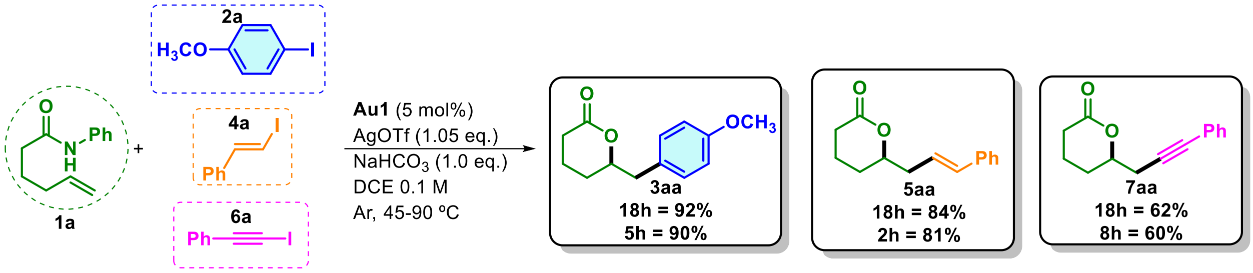

Table S31. Kinetic profile for **3aa**, **5aa**, and **7aa**.

Entry	**time / h**	Yield **3aa** / %	Yield **5aa** / %	Yield **7aa** / %
138	**0.25**	60	60	n.d.
139	**0.5**	72	66	n.d.
140	**1**	77	73	28
141	**2**	83	**81**	35
142	**5**	**90**	82	48
143	**8**	91	84	**60**
144	**18**	92	84	62

a

Figures S1. Kinetic plot of **3aa**, **5aa**, and **7aa**.

*Amide substituent screening for **3aa***

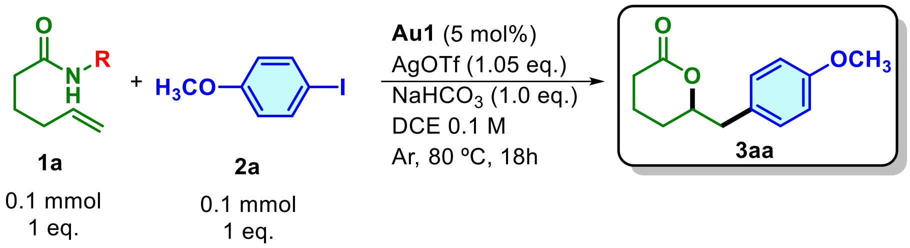

Table S32. Amide substituent for **3aa**.

Entry	R	Conv. **1a** / %	Yield **3aa** / %
145	**H**	51	46
146	**OH**	70	35
**12**	**Ph**	**95**	**92**
147	***t*Bu**	93	91

Hemilabile gold(I) catalysts screening for arylative, vinylative, and alkynylative lactonization

Scheme S1. Hemilabile gold(I) catalysts screening for aryl/vinyl/alkynyl lactonization. Above: general reaction scheme for aryl, vinyl, and alkynyl lactonization. Left: Hemilabile gold(I) catalysts employed and yields of aryl lactone 3aa (blue), vinyl lactone 5aa (orange), and alkynyl lactone 7aa (pink). Right: Radar plot of yield product with each gold catalyst.

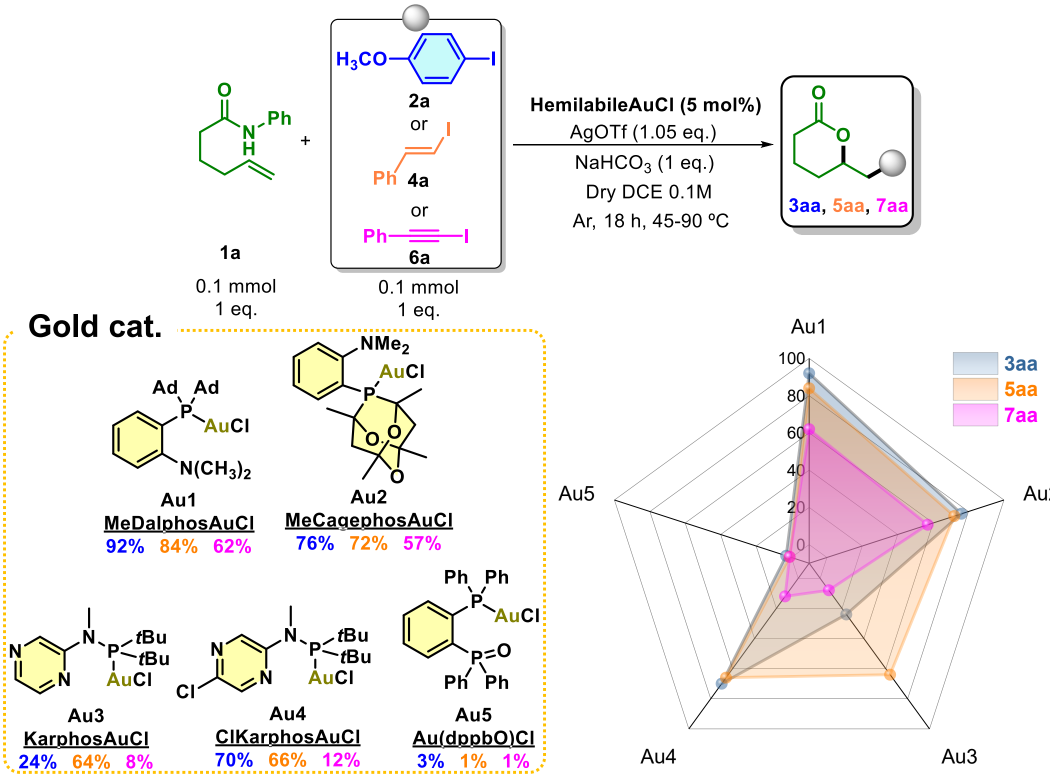

[a] 0.1 mmol of **1a**, 0.1 mmol of **2a**, 0.005 mmol of HemilabileAuCl, 0.105 mmol of AgOTf, 0.1 mmol of $NaHCO_3$, 1 mL of dry DCE (0.1M). 80 ºC for aryl (**3aa**), 90 ºC for vinyl (**5aa**), and 45 ºC for alkynyl (**7aa**) lactonization. Yields determined by ^1H NMR with CH_2Br_2 as internal standard.

Table of contents

4. MECHANISTIC INSIGHTS

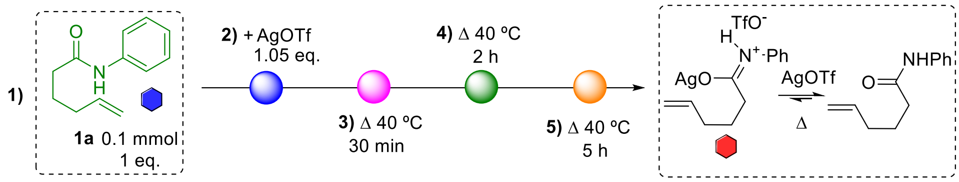

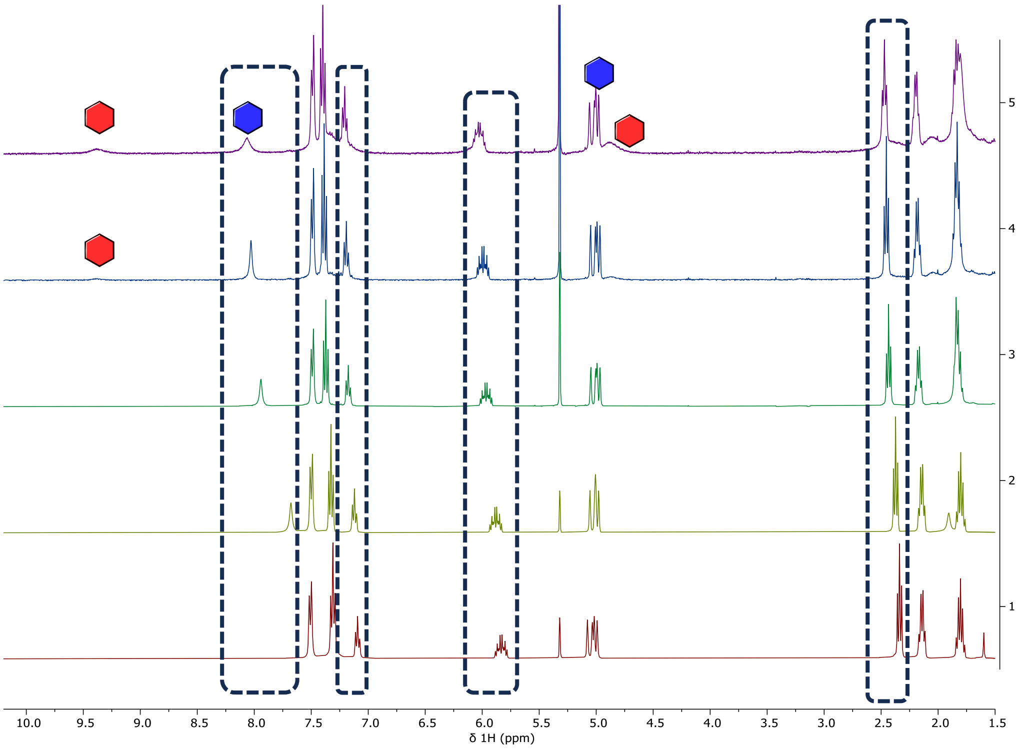

Figure S2. Iminium formation assisted by silver triflate from **1a** by ^{1}H NMR.

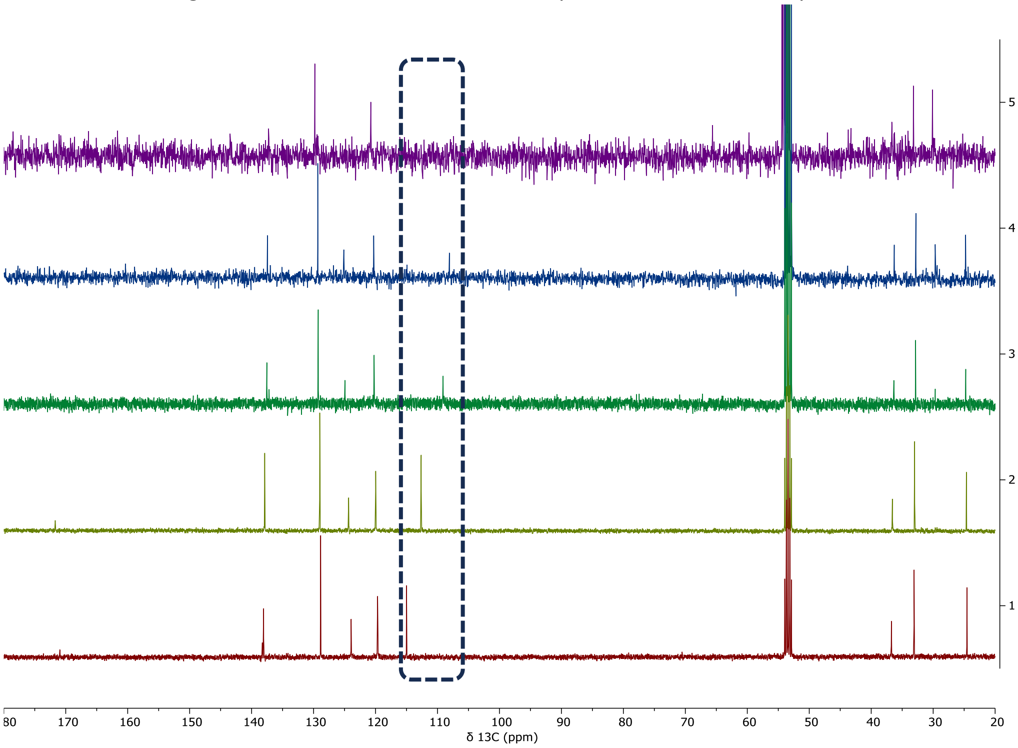

Figure S3. In situ formation of iminium assisted by silver triflate from **1a** by ^{13}C NMR. 1) Enamide **1a**. 2) Silver triflate addition. 3) Heat it to 40 °C for 30 min. 4) Heat it at 40 °C for 2h. 5) Heat it at 40 °C for 5h.

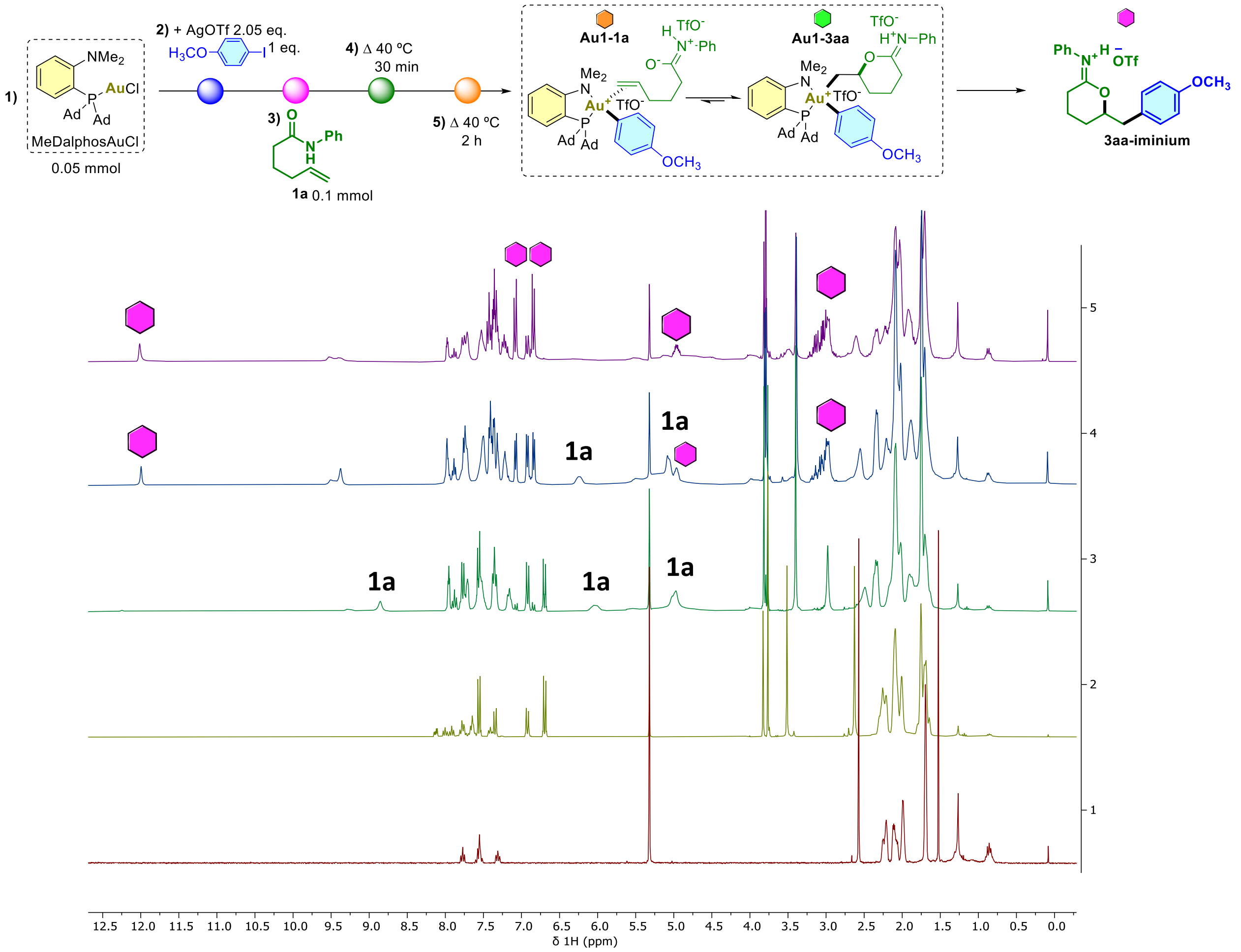

Figure S4. In situ formation of iminium product of **3aa** by ^{1}H NMR. 1) MedalphosAuCl. 2) MedalphosAuCl activated by silver triflate followed by oxidative addition with 4-iodoanysole. 3) Addition of enamide **1a**. 4) After heating to 40 °C for 30 min. 5) After heating to 40 °C for 2 h.

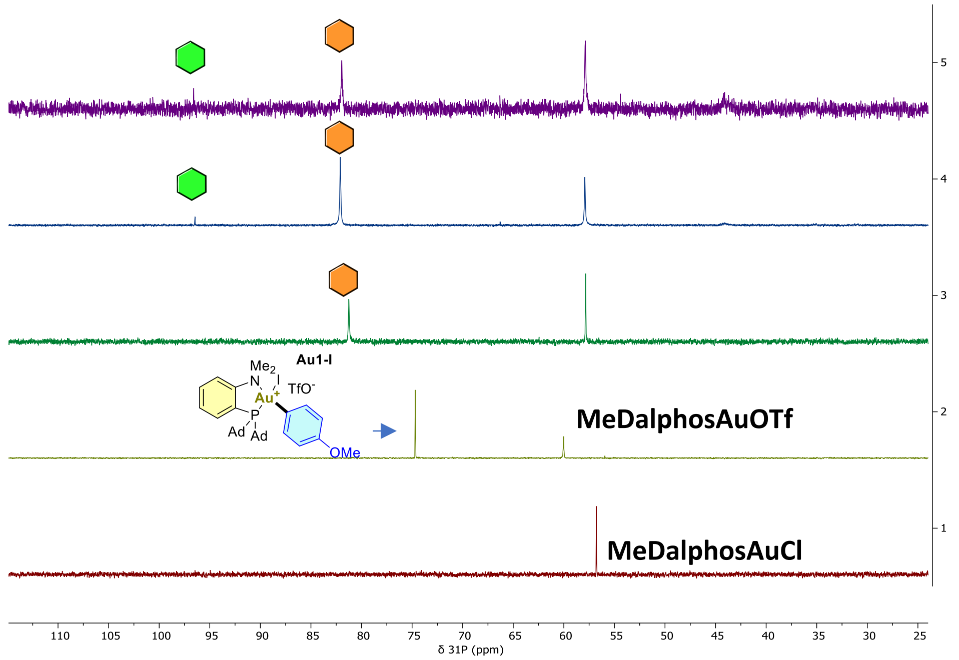

Figure S5. In situ formation of iminium product of **3aa** by ^{31}P NMR. 1) MedalphosAuCl. 2) MedalphosAuCl activated by silver triflate followed by oxidative addition to 4-iodoanysole. 3) Addition of enamide **1a**. 4) After heating to 40 °C for 30 min. 5) After heating to 40 °C for 2 h.

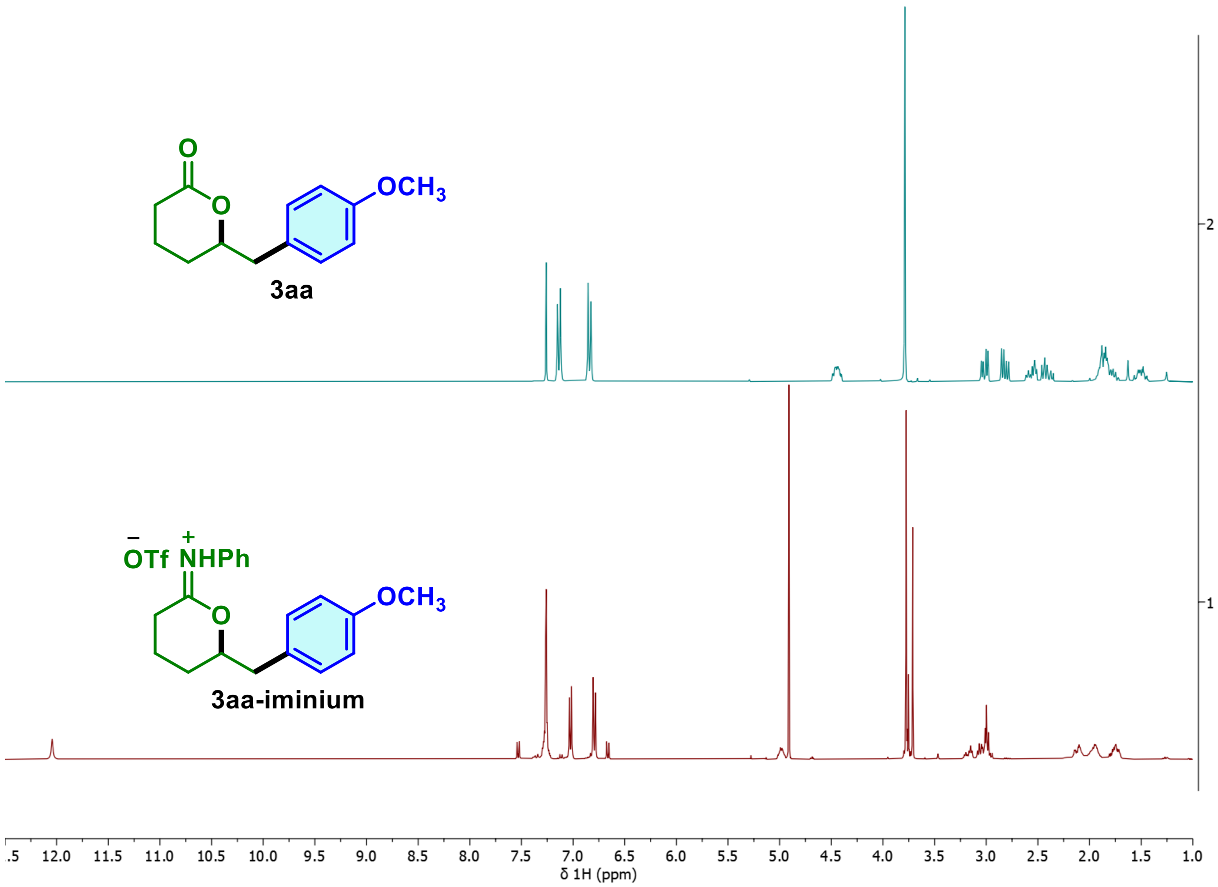

Figure S6. ^{1}H NMR comparison between of **3aa** & **3aa-iminium.**

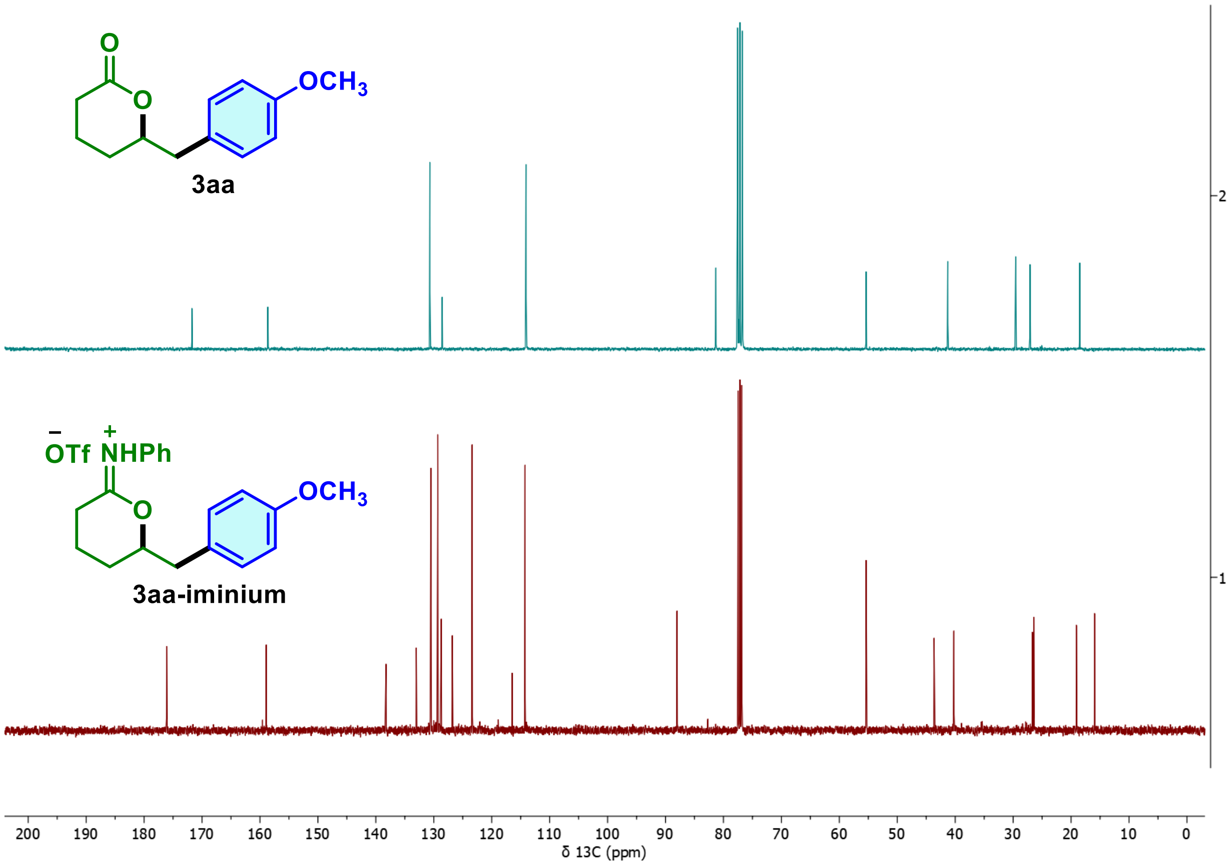

Figure S7. ^{13}C NMR comparison between of **3a** & **3aa-iminium.**

5. UNSUCCESSFUL SUBSTRATES

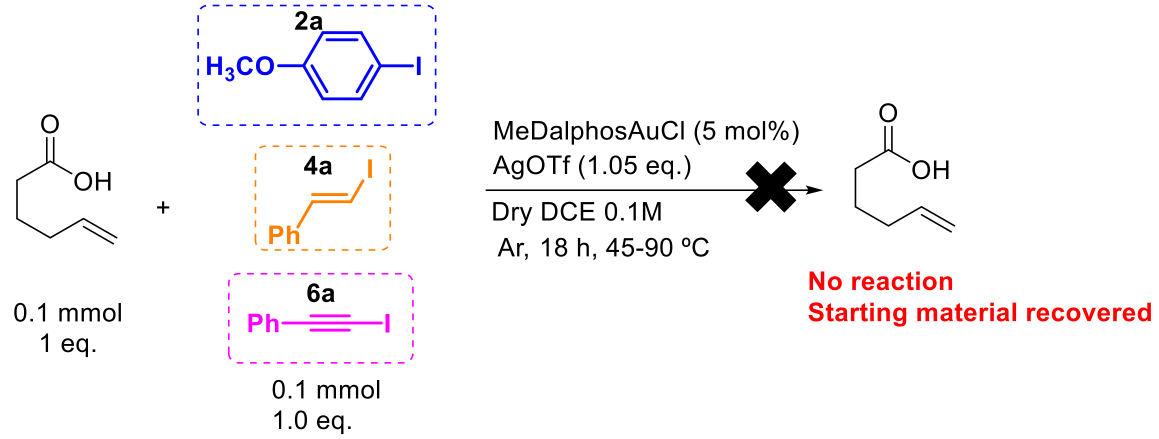

No reaction for 2a and 6a

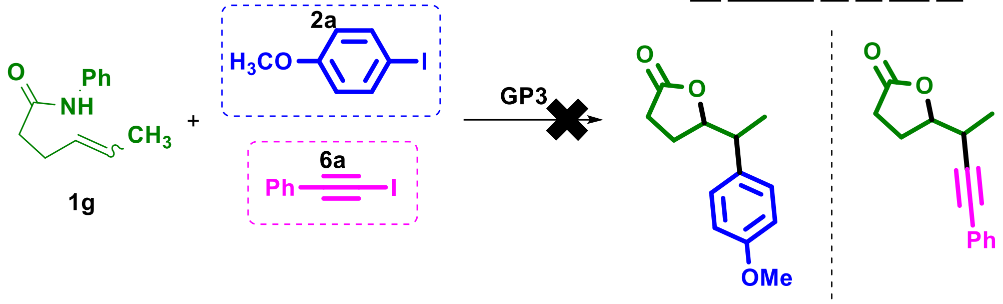

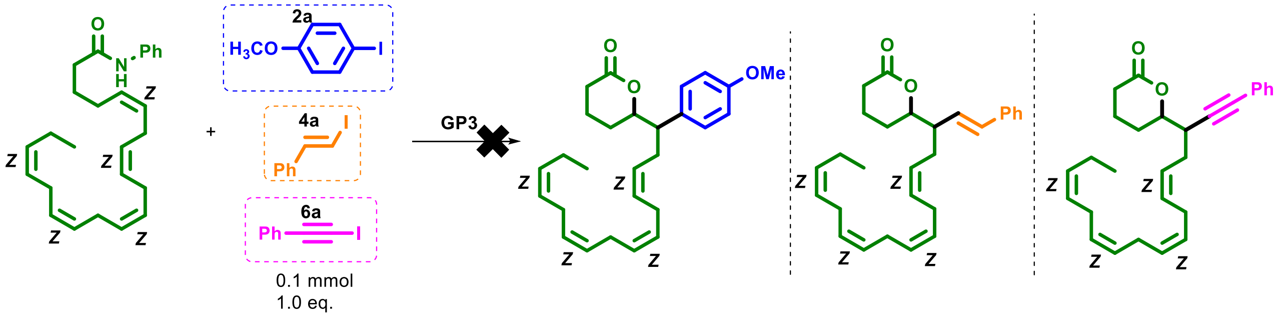

6. COMPOUNDS CHARACTERIZATION

*6.1 General procedure 3 (GP3): Cycloaddition of enamides (**1**) with aryl (**2**), vinyl (**4**) and alkynyl (**6**) iodides catalysed by an hemilabile gold(I) catalyst.*

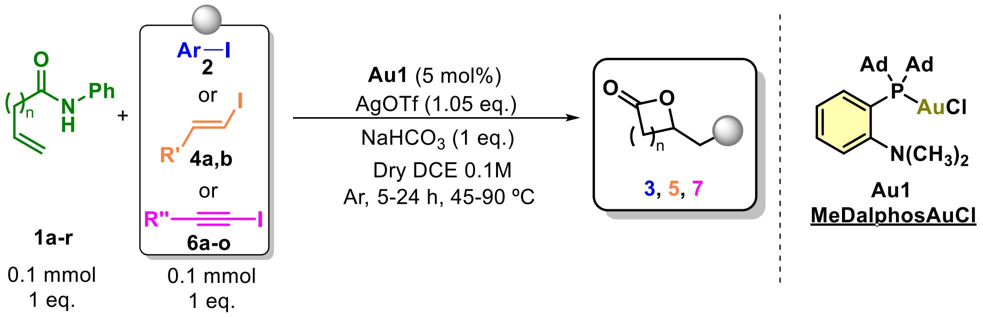

For **3**

In a dry vial (10 mL), with a stirring bar, AgOTf (0.105 mmol, 1.05 eq.), $NaHCO_3$ (0.1 mmol, 1 eq.), MeDalphosAuCl (0.005 mmol, 5 mol%), the corresponding enamide (**1**, 0.1 mmol, 1 eq.) and **2** (0.1 mmol, 1 eq.) are dissolved in 1 mL DCE under Ar atm. The mixture is stirred at 80 °C (oil bath) for 18 h (or the indicated time). Then, the mixture is filtered through a short celite plug and it is concentrated under vacuum. For NMR yield determination CH_2Br_2 is employed as standard (^{1}H NMR: 4.9 ppm, 2H). For purification flash column chromatography is performed with a mixture of hexane:EtOAc (100:0 to 80:20).

For **5**&**7**

In a dry vial (10 mL), with a stirring bar, AgOTf (0.105 mmol, 1.05 eq.), $NaHCO_3$ (0.1 mmol, 1 eq.), MeDalphosAuCl (0.005 mmol, 5 mol%), the corresponding enamide (**1**, 0.1 mmol, 1 eq.) are dissolved in 0.5 mL DCE under Ar atm. The mixture is stirred for 5 min. at r.t. and then, a solution of vinyl (**4**) or alkynyl (**6**) iodide (**5** or **7**, 0.1 mmol, 1 eq.) in 0.5 mL of DCE is injected into the reaction mixture. The reaction is stirred at 90 °C (for **5**) or 45 °C (for **7**)(or the indicated temperature in oil bath) for 18 h (or the indicated time). Then, the mixture is filtered through a short celite plug and it is concentrated under vacuum. For NMR yield determination CH_2Br_2 is employed as standard (^{1}H NMR: 4.9 ppm, 2H). For purification flash column chromatography is performed with a mixture of hexane:EtOAc (100:0 to 70:30).

3aa. 6-(4-methoxybenzyl)tetrahydro-2*H*-pyran-2-one

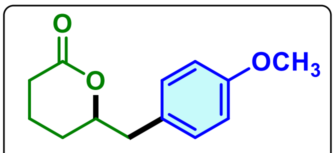

3aa

The compound (CAS: 336884-10-7) was prepared according to the *GP3* using **1a** *N*-phenylhex-5-enamide (18.9 mg, 0.1 mmol, 1 eq.), **2a** 4-iodoanylsole (23.4 mg, 0.1 mmol, 1 eq.), $NaHCO_3$ (8.4 mg, 0.1 mmol, 1 eq.), AgOTf (27.0 mg, 0.105 mmol, 1.05 eq.), MeDalphosAuCl (3.2 mg, 0.005 mmol, 0.05 equiv.) in DCE (1 mL, 0.1 M). The mixture was heated at 80 °C (oil bath) for 18 hours, giving 92% product yield according to NMR analysis with CH_2Br_2 as standard. Purification by flash column chromatography with a mixture of hexane:EtOAc (90:10 to 70:30) giving 90% (19.8 mg) isolated yield as brownish-yellow solid.

1 mmol scale

In a dry flask (100 mL), with a stirring bar, AgOTf (1.05 mmol, 1.05 eq.), $NaHCO_3$ (1 mmol, 1 eq.), MeDalphosAuCl (0.05 mmol, 5 mol%), the corresponding *N*-phenylhex-5-enamide (**1**, 1 mmol, 1 eq.) and **2** (1 mmol, 1 eq.) are dissolved in 10 mL DCE under Ar atm. The mixture is stirred at 80 °C (oil bath) for 18 h. Then, the reaction is quenched with 20 mL of NH_4Cl sat. sol extracted with CH_2Cl_2 20 mL x 3 times. The organic

phase is dried over dry $MgSO_4$, filtered and concentrated. Then, it is purified by flash column chromatography with a mixture of hexane:EtOAc (90:10 to 70:30) yielding 91% (201.4 mg) of **3aa**.

Spectra

^{1}H NMR (300 MHz, $CDCl_3$) δ 7.14 (d, *J* = 8.5 Hz, 2H), 6.84 (d, *J* = 8.5 Hz, 2H), 4.52 – 4.37 (m, 1H), 3.79 (s, 3H), 3.02 (dd, *J* = 13.9, 5.7 Hz, 1H), 2.82 (dd, *J* = 13.9, 6.9 Hz, 1H), 2.64 – 2.31 (m, 2H), 1.96 – 1.67 (m, 3H), 1.60 – 1.40 (m, 1H) ppm.

^{13}C{^{1}H} NMR (75 MHz, $CDCl_3$) δ 171.7 (s), 158.6 (s), 130.7 (s), 128.6 (s), 114.1 (s), 81.3 (s), 55.4 (s), 41.3 (s), 29.6 (s), 27.1 (s), 18.5 (s) ppm.

HRMS (ESI) m/z: $[M+Na]^+$ Calcd for $C_{13}H_{16}O_3Na$ 243.0992. Found 243.0993.

3aa-iminium. *N*-(6-(4-methoxybenzyl)tetrahydro-2*H*-pyran-2-ylidene)benzenaminium trifluoromethanesulfonate

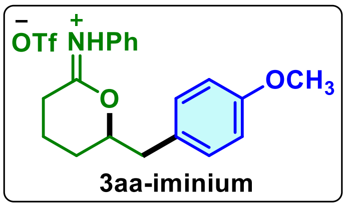

The compound was prepared according to the *GP3* using **1a** *N*-phenylhex-5-enamide (18.9 mg, 0.1 mmol, 1 eq.), **2a** 4-iodoanylsole (23.4 mg, 0.1 mmol, 1 eq.), AgOTf (27.0 mg, 0.105 mmol, 1.05 eq.), MeDalphosAuCl (3.2 mg, 0.005 mmol, 0.065 equiv.) in DCE (1 mL, 0.1 M). The mixture was heated at 80 °C (oil bath) for 5 hours, giving 83% product yield according to NMR analysis with CH_2Br_2 as standard (hydrolyse during the isolation in silica gel giving **3a**).

Spectra

^{1}H NMR (400 MHz, $CDCl_3$) δ 12.06 (s, 1H), 7.36 – 7.20 (m, 5H), 7.03 (d, *J* = 8.6 Hz, 2H), 6.79 (d, *J* = 8.6 Hz, 2H), 5.08 – 4.97 (m, 1H), 3.78 (s, 3H), 3.25 – 2.93 (m, 4H), 2.19 – 1.87 (m, 4H), 1.83 – 1.70 (m, 2H) ppm.

^{13}C{^{1}H} NMR (101 MHz, $CDCl_3$) δ 176.1 (s), 158.9 (s), 138.2 (s), 133.0 (s), 130.5 (s), 129.3 (s), 128.7 (s), 126.8 (s), 123.4 (s), 116.5 (s), 114.2 (s), 88.0 (s), 55.4 (s), 43.7 (s), 40.2 (s), 26.7 (s), 26.4 (s), 19.1 (s), 16.0 (s) ppm.

^{19}F NMR (376 MHz, $CDCl_3$) δ -79.28 ppm.

HRMS (ESI) m/z: $[M]^+$ Calcd for $C_{19}H_{22}NO_2$ 296.1645, Found 296.1645.

3ba. 7-(4-methoxybenzyl)oxepan-2-one

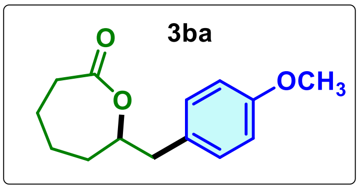

The compound (CAS: 199797-12-1) was prepared according to the *GP3* using **1b** *N*-phenylhept-6-enamide (20.3 mg, 0.1 mmol, 1 eq.), **2a** 4-iodoanylsole (23.4 mg, 0.1 mmol, 1 eq.), $NaHCO_3$ (8.4 mg, 0.1 mmol, 1 eq.), AgOTf (27.0 mg, 0.105 mmol, 1.05 eq.), MeDalphosAuCl (3.2 mg, 0.005 mmol, 0.05 equiv.) in DCE (1 mL, 0.1 M). The mixture was heated at 80 °C (oil bath) for 18 hours, giving 73% product yield according to NMR analysis with CH_2Br_2 as standard. Purification by flash column chromatography with a mixture of hexane:EtOAc (100:0 to 80:20) giving 71% (16.7 mg) isolated yield as pale-yellow solid.

Spectra

^{1}H NMR (300 MHz, $CDCl_3$) δ 7.43 – 7.37 (m, 2H), 7.30 – 7.26 (m, 3H), 4.13 (qd, *J* = 6.8, 5.2 Hz, 1H), 3.95 (dd, *J* = 14.8, 6.7 Hz, 1H), 3.80 (td, *J* = 7.8, 6.2 Hz, 1H), 2.74 – 2.57 (m, 2H), 2.15 – 2.08 (m, 1H), 2.04 – 1.86 (m, 1H), 1.86 – 1.76 (m, 1H) ppm.

^{13}C{^{1}H} NMR (75 MHz, $CDCl_3$) δ 131.8 (s), 128.3 (s), 127.8 (s), 123.9 (s), 86.9 (s), 81.9 (s), 77.5 (s), 68.7 (s), 30.9 (s), 26.4 (s), 25.9 (s) ppm.

HRMS (ESI) m/z: $[M+H]^+$ Calcd for $C_{13}H_{14}OH$ 187.1117. Found 187.1118.

3ca. 5-(4-methoxybenzyl)dihydrofuran-2(3*H*)-one

3ca

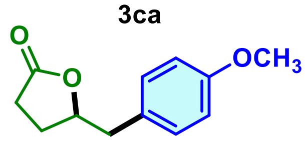

The compound (CAS: 24962-83-2) was prepared according to the *GP3* using **1c** *N*-phenylpent-4-enamide (17.5 mg, 0.1 mmol, 1 eq.), **2a** 4-iodoanylsole (23.4 mg, 0.1 mmol, 1 eq.), $NaHCO_3$ (8.4 mg, 0.1 mmol, 1 eq.), AgOTf (27.0 mg, 0.105 mmol, 1.05 eq.), MeDalphosAuCl (3.2 mg, 0.005 mmol, 0.05 equiv.) in DCE (1 mL, 0.1 M). The mixture was heated at 80 °C (oil bath) for 18 hours, giving 87% product yield according to NMR analysis with CH_2Br_2 as standard. Purification by flash column chromatography with a mixture of hexane:EtOAc (100:0 to 90:10) giving 84% (17.4 mg) isolated yield as pale-yellow oil.

Spectra

^{1}H NMR (400 MHz, $CDCl_3$) δ 7.14 (d, *J* = 8.6 Hz, 2H), 6.85 (d, *J* = 8.6 Hz, 2H), 4.70 (dq, *J* = 7.3, 6.2 Hz, 1H), 3.79 (s, 3H), 3.00 (dd, *J* = 14.1, 5.9 Hz, 1H), 2.88 (dd, *J* = 14.1, 6.2 Hz, 1H), 2.52 – 2.30 (m, 2H), 2.24 (dddd, *J* = 12.8, 9.6, 6.8, 4.8 Hz, 1H), 1.94 (dtd, *J* = 12.9, 9.2, 7.4 Hz, 1H) ppm.
^{13}C NMR (101 MHz, $CDCl_3$) δ 177.2 (s), 158.8 (s), 130.6 (s), 127.9 (s), 114.2 (s), 81.1 (s), 55.4 (s), 40.5 (s), 28.8 (s), 27.1 (s) ppm.
HRMS (ESI) m/z: $[M+Na]^+$ Calcd for $C_{12}H_{14}O_3Na$ 229.0835. Found 229.0835.

3da. 4-(4-methoxybenzyl)oxetan-2-one

3da

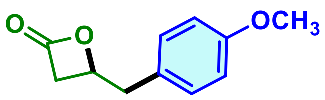

The compound (CAS: 949029-02-1) was prepared according to the *GP3* using **1d** *N*-phenylbut-3-enamid*e* (16.1 mg, 0.1 mmol, 1 eq.), **2a** 4-iodoanylsole (23.4 mg, 0.1 mmol, 1 eq.), $NaHCO_3$ (8.4 mg, 0.1 mmol, 1 eq.), AgOTf (27.0 mg, 0.105 mmol, 1.05 eq.), MeDalphosAuCl (3.2 mg, 0.005 mmol, 0.05 equiv.) in DCE (1 mL, 0.1 M). The mixture was heated at 80 °C (oil bath) for 18 hours, giving 32% product yield according to NMR analysis with CH_2Br_2 as standard. Purification by flash column chromatography with a mixture of hexane:EtOAc (100:0 to 80:10) giving 32% (6.1 mg) isolated yield as pale-yellow oil.

Spectra

^{1}H NMR (400 MHz, $CDCl_3$) δ 7.15 (d, *J* = 8.6 Hz, 2H), 6.90 (d, *J* = 8.7 Hz, 2H), 4.64 (dd, *J* = 9.0, 7.8 Hz, 1H), 4.22 (dd, *J* = 9.0, 8.0 Hz, 1H), 3.81 (s, 3H), 3.74 (p, *J* = 8.3 Hz, 1H), 2.90 (dd, *J* = 17.5, 8.7 Hz, 1H), 2.63 (dd, *J* = 17.5, 9.2 Hz, 1H) ppm.
^{13}C NMR (75 MHz, $CDCl_3$) δ 176.6 (s), 159.2 (s), 131.4 (s), 127.9 (s), 114.7 (s), 74.4 (s), 55.5 (s), 40.6 (s), 36.0 (s) ppm.
HRMS (ESI) m/z: $[M+Na]^+$ Calcd for $C_{11}H_{12}O_3Na$ 215.0679. Found 215.0679

3ea. 5-(4-methoxybenzyl)-5-methyldihydrofuran-2(3*H*)-one

3ea

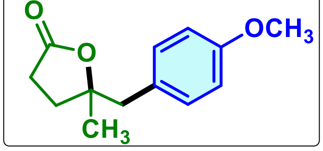

The compound (CAS: 1701988-98-8) was prepared according to the *GP3* using **1e** 4-methyl-*N*-phenylpent-4-enamide (18.9 mg, 0.1 mmol, 1 eq.), **2a** 4-iodoanysole (23.4 mg, 0.1 mmol, 1 eq.), $NaHCO_3$ (8.4 mg, 0.1 mmol, 1 eq.), AgOTf (27.0 mg, 0.105 mmol, 1.05 eq.), MeDalphosAuCl (3.2 mg, 0.005 mmol, 0.05 equiv.) in DCE (1 mL, 0.1 M). The mixture was heated at 80 °C (oil bath) for 18 hours, giving 80% product yield according to NMR analysis with CH_2Br_2 as standard. Purification by flash column chromatography with a mixture of hexane:EtOAc (100:0 to 90:10) giving 79% (17.4 mg) isolated yield as yellow oil.

Spectra

^{1}H NMR (300 MHz, $CDCl_3$) δ 7.14 (d, *J* = 8.6 Hz, 2H), 6.84 (d, *J* = 8.6 Hz, 2H), 3.79 (s, 3H), 2.96 (d, *J* = 14.1 Hz, 1H), 2.80 (d, *J* = 14.1 Hz, 1H), 2.51 – 2.30 (m, 1H), 2.25 – 1.85 (m, 3H), 1.43 (s, 3H) ppm.

^{13}C NMR (101 MHz, $CDCl_3$) δ 177.0 (s), 158.9 (s), 131.5 (s), 127.8 (s), 114.1 (s), 86.6 (s), 55.4 (s), 46.1 (s), 32.2 (s), 29.4 (s), 27.2 (s) ppm.

HRMS (APCI) m/z: $[M+H]^+$ Calcd for $C_{13}H_{16}O_3H$ 221.1172. Found 221.1172.

3ha. 3-(4-methoxybenzyl)-2-oxaspiro[5.5]undecan-1-one

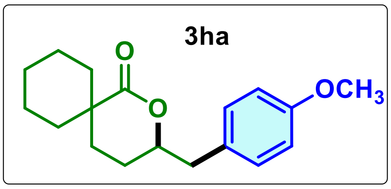

The compound was prepared according to the *GP3* using **1h** 1-(but-3-en-1-yl)-*N*-phenylcyclohexane-1-carboxamide (25.7 mg, 0.1 mmol, 1 eq.), **2a** 4-iodoanysole (23.4 mg, 0.1 mmol, 1 eq.), $NaHCO_3$ (8.4 mg, 0.1 mmol, 1 eq.), AgOTf (27.0 mg, 0.105 mmol, 1.05 eq.), MeDalphosAuCl (3.2 mg, 0.005 mmol, 0.05 equiv.) in DCE (1 mL, 0.1 M). The mixture was heated at 80 °C (oil bath) for 18 hours, giving 69% product yield according to NMR analysis with CH_2Br_2 as standard. Purification by flash column chromatography with a mixture of hexane:EtOAc (100:0 to 90:10) giving 65% (18.9 mg) isolated yield as pale-yellow oil.

Spectra

^{1}H NMR (400 MHz, $CDCl_3$) δ 7.13 (d, *J* = 8.6 Hz, 2H), 6.84 (d, *J* = 8.6 Hz, 2H), 4.42 (ddt, *J* = 9.0, 6.9, 2.4 Hz, 1H), 3.79 (s, 3H), 3.00 (dd, *J* = 13.9, 5.4 Hz, 1H), 2.79 (dd, *J* = 13.9, 7.2 Hz, 1H), 2.04 – 1.95 (m, 2H), 1.85 – 1.67 (m, 3H), 1.66 – 1.51 (m, 9H) ppm.

^{13}C NMR (101 MHz, $CDCl_3$) δ 177.5 (s), 158.6 (s), 130.7 (s), 128.7 (s), 114.1 (s), 81.2 (s), 55.4 (s), 41.7 (s), 35.9 (s), 33.5 (s), 27.7 (s), 25.6 (s), 24.9 (s), 22.3 (s), 21.1 (s), 21.0 (s) ppm.

HRMS (ESI) m/z: $[M+Na]^+$ Calcd for $C_{18}H_{24}O_3Na$ 311.1618. Found 311.1619

3ia. 5-(4-methoxybenzyl)-3,3-diphenyldihydrofuran-2(3*H*)-one

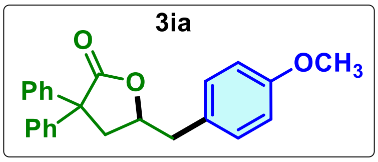

The compound was prepared according to the *GP3* using **1i** *N*,2,2-triphenylpent-4-enamide (32.7 mg, 0.1 mmol, 1 eq.), **2a** 4-iodoanysole (23.4 mg, 0.1 mmol, 1 eq.), $NaHCO_3$ (8.4 mg, 0.1 mmol, 1 eq.), AgOTf (27.0 mg, 0.105 mmol, 1.05 eq.), MeDalphosAuCl (3.2 mg, 0.005 mmol, 0.05 equiv.) in DCE (1 mL, 0.1 M). The mixture was heated at 80 °C (oil bath) for 18 hours, giving 71% product yield according to NMR analysis with CH_2Br_2 as standard. Purification by flash column chromatography with a mixture of hexane:EtOAc (100:0 to 90:10) giving 71% (25.3 mg) isolated yield as pale-yellow oil.

Spectra

^{1}H NMR (400 MHz, $CDCl_3$) δ 7.36 – 7.22 (m, 10H), 7.14 (d, *J* = 8.6 Hz, 2H), 6.85 (d, *J* = 8.6 Hz, 2H), 4.52 (dtd, *J* = 11.0, 6.3, 4.8 Hz, 1H), 3.79 (s, 3H), 3.11 (dd, *J* = 14.1, 6.4 Hz, 1H), 3.03 – 2.85 (m, 2H), 2.65 (dd, *J* = 13.0, 10.4 Hz, 1H) ppm.

^{13}C NMR (101 MHz, $CDCl_3$) δ 177.1 (s), 158.8 (s), 142.3 (s), 139.8 (s), 130.6 (s), 129.0 (s), 128.5 (s), 128.0 (s), 127.9 (s), 127.8 (s), 127.5 (s), 127.3 (s), 114.2 (s), 77.8 (s), 58.3 (s), 55.4 (s), 43.2 (s), 40.2 (s) ppm.

HRMS (ESI) m/z: $[M+Na]^+$ Calcd for $C_{24}H_{22}O_3Na$ 381.1461. Found 381.1459

3ja-1. (3R, 5R)/(3S, 5S)-5-(4-methoxybenzyl)-3-phenyldihydrofuran-2(3*H*)-one

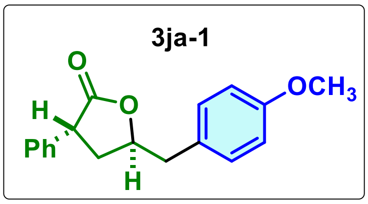

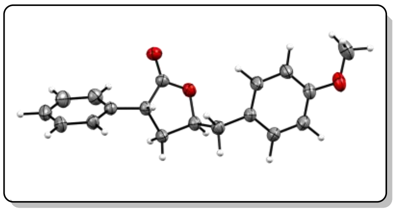

The compound was prepared according to the *GP3* using **1j** *N*,2-diphenylpent-4-enamide (25.1 mg, 0.1 mmol, 1 eq.), **2a** 4-iodoanysole (23.4 mg, 0.1 mmol, 1 eq.), $NaHCO_3$ (8.4 mg, 0.1 mmol, 1 eq.), AgOTf (27.0 mg, 0.105 mmol, 1.05 eq.), MeDalphosAuCl (3.2 mg, 0.005 mmol, 0.05 equiv.) in DCE (1 mL, 0.1 M). The mixture was heated at 80 °C (oil bath) for 18 hours, giving 61% product yield with 1:1 d.r. according to NMR analysis with CH_2Br_2 as standard. Purification by flash column chromatography with a mixture of hexane:EtOAc (100:0 to 80:10) giving 29% (8.1 mg) isolated yield of *anti*-isomer **3ja-1** as pale-yellow solid. It is crystallised using a minimum amount of CH_2Cl_2 and layered hexane.

Spectra

^{1}H NMR (300 MHz, $CDCl_3$) δ 7.32 (q, *J* = 6.8 Hz, 3H), 7.19 (t, *J* = 7.5 Hz, 4H), 6.87 (d, *J* = 8.4 Hz, 2H), 4.84 (p, *J* = 6.1 Hz, 1H), 3.80 (s, 3H), 3.61 (t, *J* = 8.4 Hz, 1H), 3.00 (qd, *J* = 14.1, 6.0 Hz, 2H), 2.56 – 2.34 (m, 2H) ppm.
^{13}C NMR (101 MHz, $CDCl_3$) δ 177.2 (s), 158.9 (s), 137.2 (s), 130.7 (s), 129.1 (s), 127.8 (s), 127.7 (s), 127.7 (s), 114.3 (s), 79.0 (s), 55.4 (s), 45.7 (s), 40.5 (s), 35.5 (s) ppm.
HRMS (ESI) m/z: $[M+Na]^+$ Calcd for C18H18O3Na 305.1148. Found 305.1147.
Melting point: 75-85 °C.

3ja-2. (3R, 5S)/(3S, 5R)-5-(4-methoxybenzyl)-3-phenyldihydrofuran-2(3*H*)-one

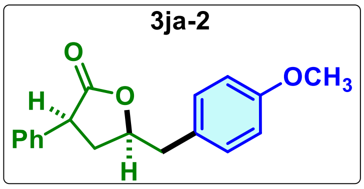

The compound was prepared according to the *GP3* using **1j** *N*,2-diphenylpent-4-enamide (25.1 mg, 0.1 mmol, 1 eq.), **2a** 4-iodoanysole (23.4 mg, 0.1 mmol, 1 eq.), $NaHCO_3$ (8.4 mg, 0.1 mmol, 1 eq.), AgOTf (27.0 mg, 0.105 mmol, 1.05 eq.), MeDalphosAuCl (3.2 mg, 0.005 mmol, 0.05 equiv.) in DCE (1 mL, 0.1 M). The mixture was heated at 80 °C (oil bath) for 18 hours, giving 61% product yield with 1:1 d.r. according to NMR analysis with CH_2Br_2 as standard. Purification by flash column chromatography with a mixture of hexane:EtOAc (100:0 to 90:10) giving 30% (8.4 mg) isolated yield of *syn*-isomer **3ja-2** as pale-yellow solid.

Spectra

^{1}H NMR (300 MHz, $CDCl_3$) δ 7.33 (q, *J* = 6.0 Hz, 3H), 7.18 (dd, *J* = 8.5, 3.4 Hz, 4H), 6.86 (d, *J* = 8.2 Hz, 2H), 4.67 (dt, *J* = 11.4, 5.8 Hz, 1H), 3.92 – 3.81 (m, 1H), 3.80 (s, 3H), 3.13 (dd, *J* = 14.1, 6.0 Hz, 1H), 2.96 (dd, *J* = 14.1, 6.3 Hz, 1H), 2.66 (ddd, *J* = 13.6, 8.7, 5.5 Hz, 1H), 2.11 (q, *J* = 12.5 Hz, 1H) ppm.
^{13}C NMR (101 MHz, $CDCl_3$) δ 177.8 (s), 158.9 (s), 137.2 (s), 130.7 (s), 129.0 (s), 128.2 (s), 127.9 (s), 127.8 (s), 114.2 (s), 78.9 (s), 55.4(s), 47.3 (s), 40.4 (s), 37.5 (s) ppm.
HRMS (ESI) m/z: $[M+Na]^+$ Calcd for $C_{18}H_{18}O_3Na$ 305.1148. Found 305.1148.
Melting point: 65-75 °C.

3la. 3-(4-methoxybenzyl)isochroman-1-one

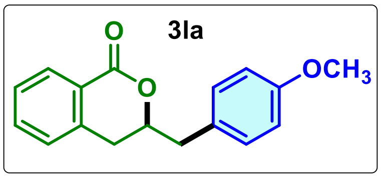

The compound was prepared according to the *GP3* using **1l** 2-allyl-*N*-phenylbenzamide (23.7 mg, 0.1 mmol, 1 eq.), **2a** 4-iodoanysole (23.4 mg, 0.1 mmol, 1 eq.), $NaHCO_3$ (8.4 mg, 0.1 mmol, 1 eq.), AgOTf (27.0 mg, 0.105 mmol, 1.05 eq.), MeDalphosAuCl (3.2 mg, 0.005 mmol, 0.05 equiv.) in DCE (1 mL, 0.1 M). The mixture was heated at 80 °C for 18 hours, giving 86%

product yield according to NMR analysis with CH_2Br_2 as standard. Purification by flash column chromatography with a mixture of hexane:EtOAc (100:0 to 90:10) giving 85% (22.9 mg) isolated yield as pale-yellow oil.

Spectra

^{1}H NMR (300 MHz, $CDCl_3$) δ 8.13 – 8.04 (m, 1H), 7.56 – 7.45 (m, 1H), 7.37 (t, *J* = 7.6 Hz, 1H), 7.18 (d, *J* = 8.6 Hz, 3H), 6.87 (d, *J* = 8.4 Hz, 2H), 4.83 – 4.60 (m, 1H), 3.80 (s, 3H), 3.19 (dd, *J* = 13.9, 5.6 Hz, 1H), 3.03 – 2.77 (m, 3H) ppm.

^{13}C NMR (75 MHz, $CDCl_3$) δ 165.6 (s), 158.8 (s), 139.1 (s), 133.8 (s), 130.7 (s), 130.4 (s), 128.2 (s), 127.8 (s), 127.6 (s), 125.4 (s), 114.2 (s), 79.4 (s), 55.4 (s), 40.4 (s), 32.4 (s) ppm.

HRMS (ESI) m/z: $[M+Na]^+$ Calcd for $C_{17}H_{16}O_3Na$ 291.0992. Found 291.0989.

3ma. 3-(4-methoxybenzyl)isobenzofuran-1(3*H*)-one

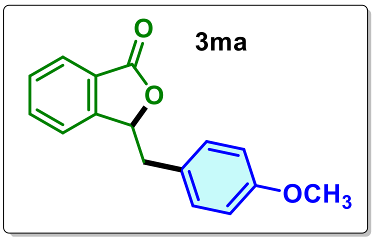

The compound (CAS: 66374-23-0) was prepared according to the *GP3* using **1m** *N*-phenyl-2-vinylbenzamide (22.3 mg, 0.1 mmol, 1 eq.), **2a** 4-iodoanysole (23.4 mg, 0.1 mmol, 1 eq.), $NaHCO_3$ (8.4 mg, 0.1 mmol, 1 eq.), AgOTf (27.0 mg, 0.105 mmol, 1.05 eq.), MedalphosAuCl (3.2 mg, 0.005 mmol, 0.05 equiv.) in DCE (1 mL, 0.1 M). The mixture was heated at 80 °C (oil bath) for 18 hours, giving 80% product yield according to NMR analysis with CH_2Br_2 as standard. Purification by flash column chromatography with a mixture of hexane:EtOAc (100:0 to 90:10) giving 79% (20.1 mg) isolated yield as white solid.

Spectra

^{1}H NMR (300 MHz, $CDCl_3$) δ 7.84 (dd, *J* = 7.6, 1.2 Hz, 1H), 7.59 (td, *J* = 7.5, 1.2 Hz, 1H), 7.48 (dd, *J* = 8.0, 7.0 Hz, 1H), 7.22 – 7.14 (m, 2H), 7.12 (d, *J* = 2.1 Hz, 2H), 6.82 (d, *J* = 8.6 Hz, 2H), 5.65 (t, *J* = 6.3 Hz, 1H), 3.79 (s, 3H), 3.24 (dd, *J* = 14.2, 6.3 Hz, 1H), 3.10 (dd, *J* = 14.2, 6.3 Hz, 1H) ppm.

In agreement with the bibliography.[64]

3fa. (3aS,6S,6aR)/(3aR,6R,6aS)-6-(4-methoxyphenyl)hexahydro-2*H*-cyclopenta[*b*]furan-2-one

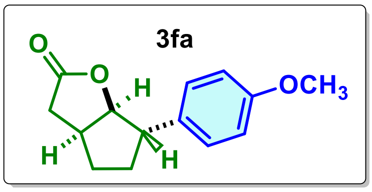

The compound was prepared according to the *GP3* using **1f** 2-(cyclopent-2-en-1-yl)-*N*-phenylacetamide (20.1 mg, 0.1 mmol, 1 eq.), **2a** 4-iodoanysole (23.4 mg, 0.1 mmol, 1 eq.), $NaHCO_3$ (8.4 mg, 0.1 mmol, 1 eq.), AgOTf (27.0 mg, 0.105 mmol, 1.05 eq.), MeDalphosAuCl (3.2 mg, 0.005 mmol, 0.05 equiv.) in DCE (1 mL, 0.1 M). The mixture was heated at 80 °C (oil bath) for 18 hours, giving 51% product yield with d.r.>20:1 according to NMR analysis with CH_2Br_2 as standard. Purification by flash column chromatography with a mixture of hexane:EtOAc (100:0 to 90:10) giving 50% (11.7 mg) isolated yield as pale-yellow oil. Diastereoisomer conformation determined by comparison with **5ia**.

Spectra

^{1}H NMR (400 MHz, $CDCl_3$) δ 7.15 (d, *J* = 8.5 Hz, 2H), 6.87 (d, *J* = 8.7 Hz, 2H), 4.86 (dd, *J* = 7.6, 4.1 Hz, 1H), 3.80 (s, 3H), 3.28 (ddd, *J* = 10.1, 6.6, 4.1 Hz, 1H), 2.95 (ddt, *J* = 12.8, 7.9, 3.9 Hz, 1H), 2.82 (dd, *J* = 18.2, 9.9 Hz, 1H), 2.41 (dd, *J* = 18.2, 3.0 Hz, 1H), 2.30 – 2.16 (m, 2H), 1.87 – 1.66 (m, 1H), 1.57 (dddd, *J* = 14.0, 11.1, 8.0, 4.3 Hz, 1H) ppm.

^{13}C NMR (75 MHz, $CDCl_3$) δ 177.4 (s), 158.5 (s), 133.7 (s), 128.2 (s), 114.2 (s), 92.0 (s), 55.4 (s), 51.3 (s), 38.6 (s), 35.9 (s), 32.8 (s), 32.1 (s) ppm.

HRMS (APCI) m/z: $[M+H]^+$ Calcd for $C_{14}H_{16}O_3H$ 233.1172. Found 233.1174

3ab. 6-benzyltetrahydro-2*H*-pyran-2-one

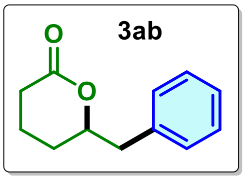

The compound (CAS: 40564-46-3) was prepared according to the *GP3* using **1a** *N*-phenylhex-5-enamide (18.9 mg, 0.1 mmol, 1 eq.), **2b** iodobencene (20.4 mg, 0.1 mmol, 1 eq.), $NaHCO_3$ (8.4 mg, 0.1 mmol, 1 eq.), AgOTf (27.0 mg, 0.105 mmol, 1.05 eq.), MeDalphosAuCl (3.2 mg, 0.005 mmol, 0.05 equiv.) in DCE (1 mL, 0.1 M). The mixture was heated at 80 °C (oil bath) for 18 hours, giving 72% product yield according to NMR analysis with CH_2Br_2 as standard. Purification by flash column chromatography with a mixture of hexane:EtOAc (100:0 to 90:10) giving 71% (13.6 mg) isolated yield as pale-yellow solid.

Spectra

^{1}H NMR (300 MHz, $CDCl_3$) δ 7.34 – 7.18 (m, 5H), 4.56 – 4.34 (m, 1H), 3.09 (dd, *J* = 13.8, 5.8 Hz, 1H), 2.88 (dd, *J* = 13.8, 7.0 Hz, 1H), 2.55 (t, *J* = 6.2 Hz, 1H), 2.50 – 2.36 (m, 1H), 1.87 (d, *J* = 11.5 Hz, 4H) ppm.
^{13}C NMR (101 MHz, CDCl3) δ 171.7 (s), 136.6 (s), 129.7 (s), 128.7 (s), 127.0 (s), 81.2 (s), 42.3 (s), 29.6 (s), 27.2 (s), 18.5 (s) ppm.
HRMS (APCI) m/z: $[M+K]^+$ Calcd for $C_{12}H_{14}O_2K$ 229.0625. Found 229.0624.

3ac. 6-(2-methylbenzyl)tetrahydro-2*H*-pyran-2-one

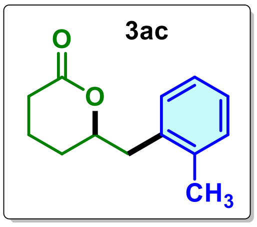

The compound was prepared according to the *GP3* using **1a** *N*-phenylhex-5-enamide (18.9 mg, 0.1 mmol, 1 eq.), **2c** 1-iodo-2-methylbenzene (21.8 mg, 0.1 mmol, 1 eq.), $NaHCO_3$ (8.4 mg, 0.1 mmol, 1 eq.), AgOTf (27.0 mg, 0.105 mmol, 1.05 eq.), MeDalphosAuCl (3.2 mg, 0.005 mmol, 0.05 equiv.) in DCE (1 mL, 0.1 M). The mixture was heated at 80 °C (oil bath) for 18 hours, giving 29% product yield according to NMR analysis with CH_2Br_2 as standard. Purification by flash column chromatography with a mixture of hexane:EtOAc (100:0 to 90:10) giving 26% (5.3 mg) isolated yield as pale-yellow oil.

Spectra

^{1}H NMR (300 MHz, $CDCl_3$) δ 7.16 (m, 4H), 4.55 – 4.44 (m, 1H), 3.16 (dd, *J* = 14.0, 5.7 Hz, 1H), 2.87 (dd, *J* = 13.8, 7.6 Hz, 1H), 2.52 (dt, *J* = 24.3, 7.2 Hz, 2H), 2.35 (s, 3H), 1.95 – 1.70 (m, 3H), 1.60 (m, 1H) ppm.
^{13}C NMR (75 MHz, $CDCl_3$) δ 171.7 (s), 136.7 (s), 134.9 (s), 130.7 (s), 130.5 (s), 127.1 (s), 126.2 (s), 80.6 (s), 39.5 (s), 29.6 (s), 27.5 (s), 19.8 (s), 18.6 (s) ppm.
HRMS (ESI) m/z: $[M+Na]^+$ Calcd for $C_{13}H_{16}O_2Na$ 227.1043. Found 227.1041.

3cd. 5-(4-(trifluoromethyl)benzyl)dihydrofuran-2(3*H*)-one

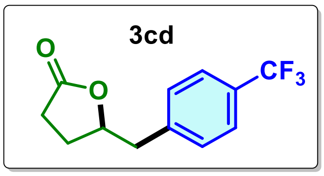

The compound (CAS: 2246354-77-6) was prepared according to the *GP3* using **1c** *N*-phenylpent-4-enamide (17.5 mg, 0.1 mmol, 1 eq.), **2d** 1-iodo-4-(trifluoromethyl)benzene (27.2 mg, 0.1 mmol, 1 eq.), $NaHCO_3$ (8.4 mg, 0.1 mmol, 1 eq.), AgOTf (27.0 mg, 0.105 mmol, 1.05 eq.), MeDalphosAuCl (3.2 mg, 0.005 mmol, 0.05 equiv.) in DCE (1 mL, 0.1 M). The mixture was heated at 80 °C (oil bath) for 18 hours, giving 87% product yield according to NMR analysis with CH_2Br_2 as standard. Purification by flash column chromatography with a mixture of hexane:EtOAc (100:0 to 90:10) giving 86% (21 mg) isolated yield as pale-yellow oil.

Spectra

1H NMR (400 MHz, $CDCl_3$) δ 7.58 (d, *J* = 8.0 Hz, 2H), 7.36 (d, *J* = 7.9 Hz, 2H), 4.73 (dtd, *J* = 7.9, 6.7, 5.5 Hz, 1H), 3.10 (dd, *J* = 14.2, 6.8 Hz, 1H), 3.01 (dd, *J* = 14.2, 5.6 Hz, 1H), 2.58 – 2.37 (m, 2H), 2.31 (dddd, *J* = 12.9, 9.4, 6.6, 4.5 Hz, 1H), 1.95 (dtd, *J* = 12.9, 9.5, 7.9 Hz, 1H) ppm.
13C NMR (101 MHz, $CDCl_3$) δ 176.7 (s), 140.3 (d, *J* = 1.6 Hz), 129.9 (s), 129.6 (q, *J* = 32.6 Hz), 125.72 (q, *J* = 3.7 Hz), 124.3 (q, *J* = 272.1 Hz), 80.3 (s), 41.3 (s), 28.8 (s), 27.5 (s) ppm.
19F NMR (376 MHz, $CDCl_3$) δ -63.51 ppm.
HRMS (ESI) m/z: $[M+Na]^+$ Calcd for $C_{12}H_{11}F_3O_2Na$ 267.0603. Found 267.0605.

3ce. 5-(4-nitrobenzyl)dihydrofuran-2(3*H*)-one

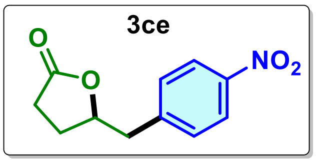

The compound (CAS: 2353020-95-6) was prepared according to the *GP3* using **1c** *N*-phenylpent-4-enamide (17.5 mg, 0.1 mmol, 1 eq.), **2e** 1-iodo-4-nitrobenzene (24.9 mg, 0.1 mmol, 1 eq.), $NaHCO_3$ (8.4 mg, 0.1 mmol, 1 eq.), AgOTf (27.0 mg, 0.105 mmol, 1.05 eq.), MeDalphosAuCl (3.2 mg, 0.005 mmol, 0.05 equiv.) in DCE (1 mL, 0.1 M). The mixture was heated at 80 °C (oil bath) for 18 hours, giving 48% product yield according to NMR analysis with CH_2Br_2 as standard. Purification by flash column chromatography with a mixture of hexane:EtOAc (100:0 to 90:10) giving 46% (10.5 mg) isolated yield as pale-yellow oil.

Spectra
1H NMR (400 MHz, $CDCl_3$) δ 8.18 (d, *J* = 8.7 Hz, 2H), 7.42 (d, *J* = 8.7 Hz, 2H), 4.74 (dtd, *J* = 8.1, 6.8, 5.3 Hz, 1H), 3.17 – 3.03 (m, 2H), 2.62 – 2.41 (m, 2H), 2.42 – 2.31 (m, 1H), 2.03 – 1.89 (m, 1H) ppm.
13C NMR (101 MHz, $CDCl_3$) δ 176.5 (s), 147.3 (s), 144.0 (s), 130.4 (s), 124.0 (s), 79.9 (s), 41.4 (s), 28.7 (s), 27.6 (s) ppm.
HRMS (ESI) m/z: $[M+Na]^+$ Calcd for $C_{11}H_{11}NO_4Na$ 244.0580. Found 244.058.

3qe. (9*H*-fluoren-9-yl)methyl ((3*S*)-5-(4-nitrobenzyl)-2-oxotetrahydrofuran-3-yl)carbamate

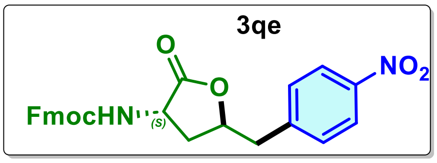

The compound was prepared according to the *GP3* using **1q** (9*H*-fluoren-9-yl)methyl (*S*)-(1-oxo-1-(phenylamino)pent-4-en-2-yl)carbamate (39.8 mg, 0.1 mmol, 1 eq.), **2e** 1-iodo-4-nitrobenzene (29.4 mg, 0.1 mmol, 1 eq.), $NaHCO_3$ (8.4 mg, 0.1 mmol, 1 eq.), AgOTf (27.0 mg, 0.105 mmol, 1.05 eq.), MeDalphosAuCl (3.2 mg, 0.005 mmol, 0.05 equiv.) in DCE (1 mL, 0.1 M). The mixture was heated at 80 °C (oil bath) for 18 hours, giving 73% product yield with 8:2 d.r. according to NMR analysis with CH_2Br_2 as standard. Purification by flash column chromatography with a mixture of hexane:EtOAc (100:0 to 70:30) giving 73% (33.2 mg) isolated yield as yellow oil.

Spectra
1H NMR (300 MHz, $CDCl_3$) δ 8.18 (d, *J* = 8.3 Hz, 2H), 7.76 (d, *J* = 7.5 Hz, 2H), 7.57 (d, *J* = 7.4 Hz, 2H), 7.40 (m, 4H), 7.31 (t, *J* = 7.4 Hz, 2H), 5.35 (bs, 1H), 4.65 (bs, 1H), 4.46 – 4.38 (m, 2H), 4.21 (t, *J* = 6.7 Hz, 1H), 3.20 – 3.04 (m, 1H), 2.84 (m, 1H), 1.92 (q, *J* = 11.8 Hz, 1H), 1.73 – 1.53 (m, 1H) ppm.
13C NMR (75 MHz, $CDCl_3$) δ 173.8 (s), 156.1 (s), 147.4 (s), 143.7 (s), 143.3 (s), 141.5 (s), 130.5 (s), 128.0 (s), 127.3 (s), 125.1 (s), 124.1 (s), 124.0 (s), 120.2 (s), 77.3 (s), 67.5 (s), 51.8 (s), 47.2 (s), 40.9 (s), 36.1 (s), 29.8 (s) ppm.
HRMS (ESI) m/z: $[M+K]^+$ Calcd for $C_{26}H_{22}N_2O_6K$ 497.1109. Found 497.1115.

5aa. (*E*)-6-cinnamyltetrahydro-2*H*-pyran-2-one

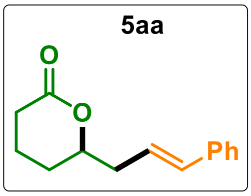

The compound (CAS: 1267664-38-9) was prepared according to the *GP3* using **1a** *N*-phenylhex-5-enamide (18.9 mg, 0.1 mmol, 1 eq.), **4a** (*E*)-(2-iodovinyl)benzene (23.0 mg, 0.1 mmol, 1 eq.), $NaHCO_3$ (8.4 mg, 0.1 mmol, 1 eq.), AgOTf (27.0 mg, 0.105 mmol, 1.05 eq.), MeDalphosAuCl (3.2 mg, 0.005 mmol, 0.05 equiv.) in DCE (1 mL, 0.1 M). The mixture was heated at 90 °C (oil bath) for 18 hours, giving 84% product yield according to NMR analysis with CH_2Br_2 as standard. Purification by flash column chromatography with a mixture of hexane:EtOAc (100:0 to 90:10) giving 77% (16.6 mg) isolated yield as yellow oil.

1 mmol scale

In a dry flask (100 mL), with a stirring bar, AgOTf (1.05 mmol, 1.05 eq.), $NaHCO_3$ (1 mmol, 1 eq.), MeDalphosAuCl (0.05 mmol, 5 mol%), *N*-phenylhex-5-enamide (**1a**, 1 mmol, 1 eq.) and **4a** (1 mmol, 1 eq.) are dissolved in 10 mL DCE under Ar atm. The mixture is stirred at 90 °C (oil bath) for 18 h. Then, the reaction is quenched with 20 mL of NH_4Cl sat. sol extracted with CH_2Cl_2 20 mL x 3 times. The organic phase is dried over dry $MgSO_4$, filtered and concentrated. Then, it is purified by flash column chromatography with a mixture of hexane:EtOAc (90:10 to 70:30) yielding 78% (168.7 mg) of **5aa**.

Spectra

^{1}H NMR (300 MHz, $CDCl_3$) δ 7.47 – 7.30 (m, 4H), 7.30 – 7.15 (m, 1H), 6.49 (d, *J* = 15.8 Hz, 1H), 6.23 (dt, *J* = 15.8, 7.2 Hz, 1H), 4.50 – 4.33 (m, 1H), 2.72 – 2.31 (m, 4H), 2.04 – 1.82 (m, 3H), 1.74 – 1.51 (m, 1H) ppm.

^{13}C NMR (75 MHz, $CDCl_3$) δ 171.8 (s), 137.2 (s), 133.7 (s), 128.7 (s), 127.6 (s), 126.3 (s), 124.2 (s), 80.2 (s), 39.4 (s), 29.6 (s), 27.5 (s), 18.6 (s) ppm.

HRMS (ESI) m/z: $[M+Na]^+$ Calcd for $C_{14}H_{16}O_2Na$ 239.1043. Found 239.1042.

5aa-iminium. (*E*)-*N*-(6-cinnamyltetrahydro-2*H*-pyran-2-ylidene)benzenaminium trifluoromethanesulfonate

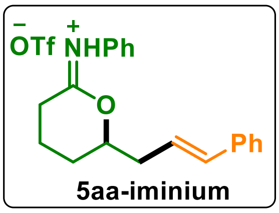

The compound was prepared according to the *GP3* using **1a** *N*-phenylhex-5-enamide (18.9 mg, 0.1 mmol, 1 eq.), **4a** (*E*)-(2-iodovinyl)benzene (23.0 mg, 0.1 mmol, 1 eq.), AgOTf (27.0 mg, 0.105 mmol, 1.05 eq.), MeDalphosAuCl (3.2 mg, 0.005 mmol, 0.05 equiv.) in DCE (1 mL, 0.1 M). The mixture was heated at 90 °C (oil bath) for 5 hours, giving 83% product yield according to NMR analysis with CH_2Br_2 as standard (hydrolyse during the isolation in silica gel).

Spectra

^{1}H NMR (400 MHz, CD_2Cl_2) δ 12.21 (s, 1H), 7.59 – 7.48 (m, 2H), 7.37 – 7.17 (m, 8H), 6.53 (dt, *J* = 15.9, 1.4 Hz, 1H), 6.18 (dt, *J* = 15.8, 7.2 Hz, 1H), 4.90 (dddd, *J* = 10.8, 7.2, 5.4, 3.4 Hz, 1H), 3.29 – 3.03 (m, 2H), 2.81 – 2.65 (m, 2H), 2.21 (dq, *J* = 13.9, 4.5 Hz, 1H), 2.11 – 2.00 (m, 3H), 1.84 (ddt, *J* = 14.4, 10.9, 8.2 Hz, 1H) ppm.

^{13}C NMR (101 MHz, CD_2Cl_2) δ 176.3 (s), 137.0 (s), 135.1 (s), 133.5 (s), 129.8 (s), 129.2 (s), 129.0 (s), 128.2 (CH), 126.6 (s), 123.6 (s), 122.9 (s), 87.6 (s), 38.8 (s), 27.2 (s), 26.9 (s), 16.4 (s) ppm.

^{19}F NMR (376 MHz, CD_2Cl_2) δ -79.66 ppm.

HRMS (ESI) m/z: $[M]^+$ Calcd for $C_{20}H_{22}NO$ 292.1696, found 292.1695.

5ba. (*E*)-7-cinnamyloxepan-2-one

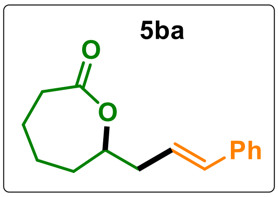

The compound (CAS: 2043954-55-6) was prepared according to the *GP3* using **1b** *N*-phenylhept-6-enamide (20.3 mg, 0.1 mmol, 1 eq.), **4a** (*E*)-(2-iodovinyl)benzene (23.0 mg, 0.1 mmol, 1 eq.), $NaHCO_3$ (8.4 mg, 0.1 mmol, 1 eq.), AgOTf (27.0 mg, 0.105 mmol, 1.05 eq.), MeDalphosAuCl (3.2 mg, 0.005 mmol, 0.05 equiv.) in DCE (1 mL, 0.1 M). The mixture was heated at 90 °C (oil bath) for 18 hours, giving 55% product yield according to NMR analysis with CH_2Br_2 as standard. Purification by flash column chromatography with a mixture of hexane:EtOAc (100:0 to 90:10) giving 50% (11.6 mg) isolated yield as pale-yellow oil.

Spectra

^{1}H NMR (400 MHz, $CDCl_3$) δ 7.44 – 7.15 (m, 5H), 6.48 (d, *J* = 16.1 Hz, 1H), 6.25 (ddd, *J* = 15.7, 7.6, 6.6 Hz, 1H), 4.36 (dt, *J* = 8.6, 6.2 Hz, 1H), 2.74 – 2.55 (m, 3H), 2.55 – 2.44 (m, 1H), 2.38 (t, *J* = 7.5 Hz, 1H), 2.21 (q, *J* = 6.9 Hz, 1H), 2.05 – 1.88 (m, 3H), 1.78 (p, *J* = 7.5 Hz, 1H) ppm.

^{13}C NMR (101 MHz, $CDCl_3$) δ 175.6 (s), 137.2 (s), 133.5 (s), 128.7 (s), 127.6 (s), 126.3 (s), 125.1 (s), 80.4 (s), 40.1 (s), 35.1 (s), 34.2 (s), 28.5 (s), 23.1 (s) ppm.

HRMS (ESI) m/z: $[M+Na]^+$ Calcd for $C_{15}H_{18}O_2Na$ 253.1199. Found 253.1199.

5ca. (*E*)-5-cinnamyldihydrofuran-2(3*H*)-one

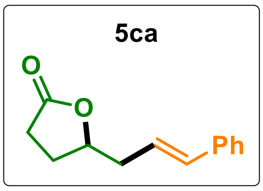

The compound (CAS: 1644290-97-0) was prepared according to the *GP3* using **1c** *N*-phenylpent-4-enamide (17.5 mg, 0.1 mmol, 1 eq.), **3a** (*E*)-(2-iodovinyl)benzene (23.0 mg, 0.1 mmol, 1 eq.), $NaHCO_3$ (8.4 mg, 0.1 mmol, 1 eq.), AgOTf (27.0 mg, 0.105 mmol, 1.05 eq.), MeDalphosAuCl (3.2 mg, 0.005 mmol, 0.05 equiv.) in DCE (1 mL, 0.1 M). The mixture was heated at 90 °C (oil bath) for 18 hours, giving 77% product yield according to NMR analysis with CH_2Br_2 as standard. Purification by flash column chromatography with a mixture of hexane:EtOAc (100:0 to 90:10) giving 71% (14.3 mg) isolated yield as pale-yellow solid.

Spectra

^{1}H NMR (400 MHz, $CDCl_3$) δ 7.42 – 7.28 (m, 4H), 7.28 – 7.20 (m, 1H), 6.52 (dt, *J* = 15.9, 1.5 Hz, 1H), 6.18 (dt, *J* = 15.8, 7.2 Hz, 1H), 4.63 (dq, *J* = 7.6, 6.2 Hz, 1H), 2.64 (ddd, *J* = 7.2, 6.2, 1.4 Hz, 1H), 2.60 – 2.49 (m, 3H), 2.41 – 2.28 (m, 1H), 2.06 – 1.91 (m, 1H) ppm.

^{13}C NMR (101 MHz, $CDCl_3$) δ 177.2 (s), 137.0 (s), 134.1 (s), 128.7 (s), 127.7 (s), 126.3 (s), 123.5 (s), 80.1 (s), 38.9 (s), 28.8 (s), 27.3 (s) ppm.

HRMS (ESI) m/z: $[M+Na]^+$ Calcd for $C_{13}H_{14}O_2Na$ 225.0886. Found 225.0885.

5da. (*E*)-4-cinnamyloxetan-2-one

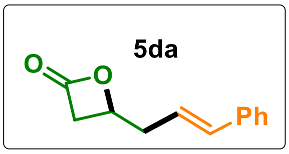

The compound was prepared according to the *GP3* using **1d** *N*-phenylbut-3-enamide (16.1 mg, 0.1 mmol, 1 eq.), **3a** (*E*)-(2-iodovinyl)benzene (23.0 mg, 0.1 mmol, 1 eq.), $NaHCO_3$ (8.4 mg, 0.1 mmol, 1 eq.), AgOTf (27.0 mg, 0.105 mmol, 1.05 eq.), MeDalphosAuCl (3.2 mg, 0.005 mmol, 0.05 equiv.) in DCE (1 mL, 0.1 M). The mixture was heated at 90 °C (oil bath) for 18 hours, giving 57% product yield according to NMR analysis with CH_2Br_2 as standard. Purification by flash column chromatography with a mixture of hexane:EtOAc (100:0 to 90:10) giving 57% (10.7 mg) isolated yield as pale-yellow oil.

Spectra

^{1}H NMR (300 MHz, $CDCl_3$) δ 7.39 – 7.28 (m, 5H), 6.53 (d, *J* = 15.8 Hz, 1H), 6.11 (dd, *J* = 15.8, 8.1 Hz, 1H), 4.51 (dd, *J* = 9.0, 7.7 Hz, 1H), 4.10 (t, *J* = 8.5 Hz, 1H), 3.40 (h, *J* = 8.3 Hz, 1H), 2.76 (dd, *J* = 17.4, 8.4 Hz, 1H), 2.47 (dd, *J* = 17.4, 9.2 Hz, 1H) ppm.
^{13}C NMR (101 MHz, $CDCl_3$) δ 176.5 (s), 136.2 (s), 132.9 (s), 128.9 (s), 128.2 (s), 127.0 (s), 126.4 (s), 72.6 (s), 39.7 (s), 34.8 (s) ppm.
HRMS (ESI) m/z: $[M+Na]^+$ Calcd for $C_{12}H_{12}O_2Na$ 211.0729. Found 211.0729.

5ea. (*E*)-5-cinnamyl-5-methyldihydrofuran-2(3*H*)-one

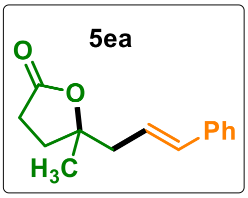

The compound (CAS: 1906891-33-5) was prepared according to the *GP3* using **1e** 4-methyl-*N*-phenylpent-4-enamide (18.9 mg, 0.1 mmol, 1 eq.), **3a** (*E*)-(2-iodovinyl)benzene (23.0 mg, 0.1 mmol, 1 eq.), $NaHCO_3$ (8.4 mg, 0.1 mmol, 1 eq.), AgOTf (27.0 mg, 0.105 mmol, 1.05 eq.), MeDalphosAuCl (3.2 mg, 0.005 mmol, 0.05 equiv.) in DCE (1 mL, 0.1 M). The mixture was heated at 90 °C (oil bath) for 18 hours, giving 50% product yield according to NMR analysis with CH_2Br_2 as standard. Purification by flash column chromatography with a mixture of hexane:EtOAc (100:0 to 90:10) giving 49% (10.5 mg) isolated yield as yellow oil.

Spectra
^{1}H NMR (300 MHz, $CDCl_3$) δ 7.40 – 7.20 (m, 5H), 6.50 (d, *J* = 15.7 Hz, 1H), 6.18 (dt, *J* = 15.9, 7.4 Hz, 1H), 2.73 – 2.48 (m, 4H), 2.29 – 2.13 (m, 1H), 2.07 – 1.92 (m, 1H), 1.45 (s, 3H) ppm.
^{13}C NMR (101 MHz, $CDCl_3$) δ 176.8 (s), 137.0 (s), 134.8 (s), 128.8 (s), 127.8 (s), 126.4 (s), 123.5 (s), 86.4 (s), 44.6 (s), 32.5 (s), 29.3 (s), 26.4 (s) ppm.
HRMS (APCI) m/z: $[M+H]^+$ Calcd for $C_{14}H_{16}O_2H$ 217.1223. Found 217.1224.

5ga. 5-((*E*)-4-phenylbut-3-en-2-yl)dihydrofuran-2(3*H*)-one

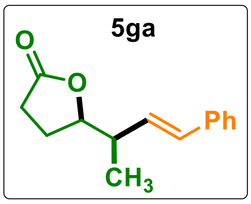

The compound was prepared according to the *GP3* using **1g** *N*-phenylhex-4-enamide (18.9 mg, 0.1 mmol, 1 eq.), **3a** (*E*)-(2-iodovinyl)benzene (23.0 mg, 0.1 mmol, 1 eq.), $NaHCO_3$ (8.4 mg, 0.1 mmol, 1 eq.), AgOTf (27.0 mg, 0.105 mmol, 1.05 eq.), MeDalphosAuCl (3.2 mg, 0.005 mmol, 0.05 equiv.) in DCE (1 mL, 0.1 M). The mixture was heated at 90 °C (oil bath) for 18 hours, giving 43% product yield according to NMR analysis with CH_2Br_2 as standard. Purification by flash column chromatography with a mixture of hexane:EtOAc (100:0 to 90:10) giving 42% (9.0 mg) isolated yield as yellow oil.

Spectra
^{1}H NMR (300 MHz, $CDCl_3$) δ 7.51 – 7.18 (m, 5H), 6.50 (d, *J* = 16.1 Hz, 1H), 6.06 (dd, *J* = 15.9, 8.0 Hz, 1H), 4.40 (q, *J* = 7.3 Hz, 1H), 2.62 (q, *J* = 7.0 Hz, 1H), 2.56 – 2.46 (m, 2H), 2.29 – 2.18 (m, 1H), 2.10 – 1.90 (m, 1H), 1.22 (s, 3H) ppm.
^{13}C NMR (101 MHz, $CDCl_3$) δ 177.3 (s), 137.1 (s), 132.2 (s), 129.2 (s), 128.8 (s), 127.8 (s), 126.4 (s), 84.0 (s), 42.3 (s), 29.0 (s), 25.6 (s), 16.6 (s) ppm.
HRMS (ESI) m/z: $[M+Na]^+$ Calcd for $C_{14}H_{16}O_2Na$ 239.1043. Found 239.1042.

5ia. (*E*)-5-cinnamyl-3,3-diphenyldihydrofuran-2(3*H*)-one

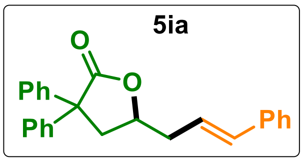

The compound was prepared according to the *GP3* using **1i** *N*,2,2-triphenylpent-4-enamide (32.7 mg, 0.1 mmol, 1 eq.), **3a** (*E*)-(2-iodovinyl)benzene (23.0 mg, 0.1 mmol, 1 eq.), $NaHCO_3$ (8.4 mg, 0.1 mmol, 1 eq.), AgOTf (27.0 mg, 0.105 mmol, 1.05 eq.), MeDalphosAuCl (3.2 mg, 0.005 mmol, 0.05 equiv.) in DCE (1 mL, 0.1

M). The mixture was heated at 90 °C (oil bath) for 18 hours, giving 68% product yield according to NMR analysis with CH_2Br_2 as standard. Purification by flash column chromatography with a mixture of hexane:EtOAc (100:0 to 90:10) giving 67% (23.7 mg) isolated yield as pale-yellow solid.

Spectra

^{1}H NMR (400 MHz, $CDCl_3$) δ 7.42 – 7.27 (m, 15H), 6.52 (d, *J* = 15.8 Hz, 1H), 6.20 (dt, *J* = 15.2, 7.2 Hz, 1H), 4.48 (dd, *J* = 10.8, 5.3 Hz, 1H), 3.07 (dd, *J* = 13.0, 4.9 Hz, 1H), 2.81 – 2.67 (m, 2H), 2.68 – 2.53 (m, 1H) ppm.
^{13}C NMR (101 MHz, $CDCl_3$) δ 177.2 (s), 142.1 (s), 140.0 (s), 137.0 (s), 134.1 (s), 129.1 (s), 128.7 (s), 128.5 (s), 127.8 (s), 127.7 (s), 127.5 (s), 127.4 (s), 126.3 (s), 123.4 (s), 77.6 (s), 58.3 (s), 43.3 (s), 38.4 (s) ppm.
HRMS (ESI) m/z: $[M+Na]^+$ Calcd for $C_{25}H_{22}O_2Na$ 377.1512. Found 377.1507
Melting point: 70-80 °C.

5ja-1. (*E*)- (3*R*,5*S*)/(3*S*,5*R*)-5-cinnamyl-3-phenyldihydrofuran-2(3*H*)-one

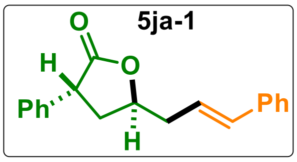

The compound was prepared according to the *GP3* using **1j** *N*,2-diphenylpent-4-enamide (25.1 mg, 0.1 mmol, 1 eq.), **3a** (*E*)-(2-iodovinyl)benzene (23.0 mg, 0.1 mmol, 1 eq.), $NaHCO_3$ (8.4 mg, 0.1 mmol, 1 eq.), AgOTf (27.0 mg, 0.105 mmol, 1.05 eq.), MeDalphosAuCl (3.2 mg, 0.005 mmol, 0.05 equiv.) in DCE (1 mL, 0.1 M). The mixture was heated at 90 °C (oil bath) for 18 hours, giving 73% product yield with 1:1 d.r. according to NMR analysis with CH_2Br_2 as standard. Purification by flash column chromatography with a mixture of hexane:EtOAc (100:0 to 80:20) giving 27% (7.4 mg) isolated yield for the *anti*-diasteresoisomer **5ja-1** as yellow oil. Diastereoisomer attributed by comparison with **3ja-1**.

Spectra

^{1}H NMR (300 MHz, $CDCl_3$) δ 7.54 – 7.09 (m, 10H), 6.56 (d, *J* = 15.8 Hz, 1H), 6.21 (dt, *J* = 15.3, 7.2 Hz, 1H), 4.78 (p, *J* = 6.4 Hz, 1H), 3.92 (t, *J* = 8.2 Hz, 1H), 2.68 (dt, *J* = 14.5, 7.0 Hz, 2H), 2.52 (t, *J* = 7.4 Hz, 2H) ppm.
^{13}C NMR (75 MHz, $CDCl_3$) δ 177.2 (s), 137.2 (s), 137.0 (s), 134.3 (s), 129.2 (s), 128.8 (s), 127.8 (s), 127.8 (s), 127.7 (s), 126.4 (s), 123.4 (s), 78.2 (s), 45.7 (s), 38.8 (s), 35.7 (s) ppm.
HRMS (ESI) m/z: $[M+Na]^+$ Calcd for $C_{19}H_{18}O_2Na$ 301.1199. Found 301.1201.

5ja-2. (*E*)-(3*S*,5*S*)/(3*R*,5*R*)-5-cinnamyl-3-phenyldihydrofuran-2(3*H*)-one

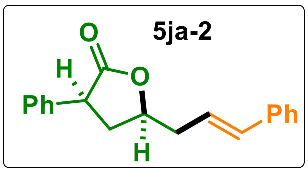

The compound was prepared according to the *GP3* using **1j** *N*,2-diphenylpent-4-enamide (25.1 mg, 0.1 mmol, 1 eq.), **3a** (*E*)-(2-iodovinyl)benzene (23.0 mg, 0.1 mmol, 1 eq.), $NaHCO_3$ (8.4 mg, 0.1 mmol, 1 eq.), AgOTf (27.0 mg, 0.105 mmol, 1.05 eq.), MeDalphosAuCl (3.2 mg, 0.005 mmol, 0.05 equiv.) in DCE (1 mL, 0.1 M). The mixture was heated at 90 °C (oil bath) for 18 hours, giving 73% product yield with 1:1 d.r. according to NMR analysis with CH_2Br_2 as standard. Purification by flash column chromatography with a mixture of hexane:EtOAc (100:0 to 80:20) giving 45% (12.4 mg) isolated yield for the *syn*-diasteresoisomer **5ja-2** as orange oil. Diastereoisomer attributed by comparison with **3ja-2**.

Spectra

^{1}H NMR (300 MHz, $CDCl_3$) δ 7.43 – 7.29 (m, 10H), 6.55 (d, *J* = 16.0 Hz, 1H), 6.23 (dt, *J* = 14.9, 7.1 Hz, 1H), 4.64 (dt, *J* = 10.6, 5.8 Hz, 1H), 3.98 – 3.84 (m, 1H), 2.73 (dtd, *J* = 27.6, 14.0, 7.7 Hz, 3H), 2.26 – 2.05 (m, 1H) ppm.
^{13}C NMR (101 MHz, $CDCl_3$) δ 176.8 (s), 137.0 (s), 136.6 (s), 134.2 (s), 129.0 (s), 128.8 (s), 128.2 (s), 127.8 (s), 127.7 (s), 126.4 (s), 123.4 (s), 77.9 (s), 47.2 (s), 38.7 (s), 37.5 (s) ppm.
HRMS (ESI) m/z: $[M+Na]^+$ Calcd for $C_{19}H_{18}O_2Na$ 301.1199. Found 301.1202.

5fa. (3aS,6S,6aR)/(3aR,6R,6aS)-6-((E)-styryl)hexahydro-2*H*-cyclopenta[*b*]furan-2-one

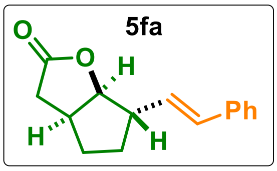

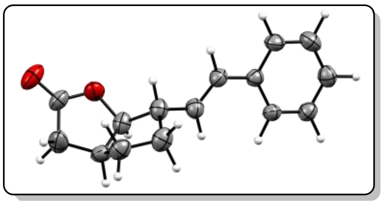

The compound was prepared according to the *GP3* using **1f** 2-(cyclopent-2-en-1-yl)-*N*-phenylacetamide (20.1 mg, 0.1 mmol, 1 eq.), **3a** (*E*)-(2-iodovinyl)benzene (23.0 mg, 0.1 mmol, 1 eq.), $NaHCO_3$ (8.4 mg, 0.1 mmol, 1 eq.), AgOTf (27.0 mg, 0.105 mmol, 1.05 eq.), MeDalphosAuCl (3.2 mg, 0.005 mmol, 0.05 equiv.) in DCE (1 mL, 0.1 M). The mixture was heated at 90 °C (oil bath) for 18 hours, giving 53% product yield with >20:1 d.r. according to NMR analysis with CH_2Br_2 as standard. Purification by flash column chromatography with a mixture of hexane:EtOAc (100:0 to 90:10) giving 46% (10.6 mg) isolated yield as pale-yellow solid. It is crystallised using a minimum amount of CH_2Cl_2 and layered hexane.

Spectra
^{1}H NMR (300 MHz, $CDCl_3$) δ 7.40 – 7.15 (m, 5H), 6.49 (d, *J* = 16.0 Hz, 1H), 6.15 (dd, *J* = 16.0, 7.2 Hz, 1H), 4.79 (dd, *J* = 7.2, 3.2 Hz, 1H), 3.05 – 2.89 (m, 2H), 2.82 (dd, *J* = 17.9, 10.0 Hz, 1H), 2.36 (dd, *J* = 17.9, 2.6 Hz, 1H), 2.18 (dd, *J* = 13.2, 7.1 Hz, 1H), 2.10 – 1.97 (m, 1H), 1.68 (dd, *J* = 13.0, 6.6 Hz, 1H), 1.54 (dd, *J* = 12.9, 6.5 Hz, 1H) ppm.
^{13}C NMR (75 MHz, $CDCl_3$) δ 177.4 (s), 137.1 (s), 131.2 (s), 129.1 (s), 128.7 (s), 127.6 (s), 126.3 (s), 90.4 (s), 49.4 (s), 37.8 (s), 36.0 (s), 32.4 (s), 30.4 (s) ppm.
HRMS (ESI) m/z: $[M+Na]^+$ Calcd for $C_{15}H_{16}O_2Na$ 251.1043. Found 251.1042.
Melting point: 120-130 °C.

5ka. (*E*)-4-cinnamyl-1,3-dioxolan-2-one

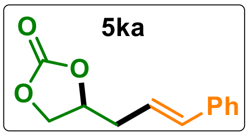

The compound was prepared according to the *GP3* using **1k** Allyl phenylcarbamate (17.7 mg, 0.1 mmol, 1 eq.), **3a** (*E*)-(2-iodovinyl)benzene (23.0 mg, 0.1 mmol, 1 eq.), $NaHCO_3$ (8.4 mg, 0.1 mmol, 1 eq.), AgOTf (27.0 mg, 0.105 mmol, 1.05 eq.), MeDalphosAuCl (3.2 mg, 0.005 mmol, 0.05 equiv.) in DCE (1 mL, 0.1 M). The mixture was heated at 90 °C (oil bath) for 18 hours, giving 63% product yield according to NMR analysis with CH_2Br_2 as standard. Purification by flash column chromatography with a mixture of hexane:EtOAc (100:0 to 90:10) giving 61% (12.5 mg) isolated yield as yellow oil.

Spectra
^{1}H NMR (300 MHz, $CDCl_3$) δ 7.41 – 7.20 (m, 5H), 6.57 (dt, *J* = 15.8, 1.4 Hz, 1H), 6.11 (dt, *J* = 15.8, 7.2 Hz, 1H), 4.84 (dq, *J* = 7.8, 6.3 Hz, 1H), 4.53 (dd, *J* = 8.6, 7.9 Hz, 1H), 4.20 (dd, *J* = 8.5, 6.7 Hz, 1H), 2.69 (dddd, *J* = 12.2, 7.5, 6.2, 1.4 Hz, 2H) ppm.
^{13}C NMR (101 MHz, $CDCl_3$) δ 155.0 (s), 136.5 (s), 135.5 (s), 128.8 (s), 128.1 (s), 126.4 (s), 121.0 (s), 76.0 (s), 68.7 (s), 37.2 (s) ppm.
HRMS (ESI) m/z: $[M+Na]^+$ Calcd for C12H12O_3Na 227.0679. Found 227.0679.

5ab. (*E*)-6-(hept-2-en-1-yl)tetrahydro-2H-pyran-2-one

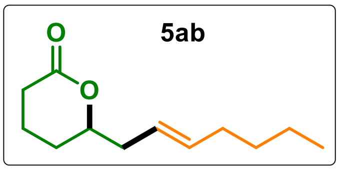

The compound (CAS: 34727-34-9) was prepared according to the *GP3* using **1a** *N*-phenylhex-5-enamide (18.9 mg, 0.1 mmol, 1 eq.), **3b** (*E*)-1-iodohex-1-ene (22.4 mg, 0.1 mmol, 1 eq.), $NaHCO_3$ (8.4 mg, 0.1 mmol, 1 eq.), AgOTf (27.0 mg, 0.105 mmol, 1.05 eq.), MeDalphosAuCl (3.2 mg, 0.005 mmol, 0.05 equiv.) in DCE (1 mL, 0.1 M). The mixture was heated at 90 °C (oil bath) for 18 hours, giving 68% product yield according to NMR analysis with CH_2Br_2 as standard. Purification by flash column

chromatography with a mixture of hexane:EtOAc (100:0 to 90:10) giving 65% (12.8 mg) isolated yield as yellow oil.

Spectra

^{1}H NMR (300 MHz, $CDCl_3$) δ 5.54 (dt, *J* = 15.3, 6.5 Hz, 1H), 5.40 (dt, *J* = 15.4, 6.8 Hz, 1H), 4.28 (dtd, *J* = 9.2, 6.2, 2.6 Hz, 1H), 2.66 – 2.50 (m, 1H), 2.51 – 2.36 (m, 2H), 2.30 (q, *J* = 7.0 Hz, 1H), 2.06 – 1.79 (m, 5H), 1.40 – 1.27 (m, 5H), 0.88 (t, *J* = 6.7 Hz, 3H) ppm.

^{13}C NMR (75 MHz, $CDCl_3$) δ 172.0 (s), 135.0 (s), 123.8 (s), 80.5 (s), 39.0 (s), 32.7 (s), 31.5 (s), 29.6 (s), 29.1 (s), 27.3 (s), 22.6 (s), 18.6 (s), 14.2 (s) ppm.

HRMS (ESI) m/z: [M+CH_3OH] 1- Calcd for $C_{12}H_{19}O_2$+CH_3OH 227.1653. Found 227.1652.

7aa. 6-(3-phenylprop-2-yn-1-yl)tetrahydro-2*H*-pyran-2-one

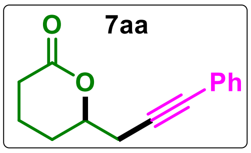

The compound was prepared according to the *GP3* using **1a** *N*-phenylhex-5-enamide (18.9 mg, 0.1 mmol, 1 eq.), **6a** (iodoethynyl)benzene (22.8 mg, 0.1 mmol, 1 eq.), $NaHCO_3$ (8.4 mg, 0.1 mmol, 1 eq.), AgOTf (27.0 mg, 0.105 mmol, 1.05 eq.), MeDalphosAuCl (3.2 mg, 0.005 mmol, 0.05 equiv.) in DCE (1 mL, 0.1 M). The mixture was heated at 45 °C (oil bath) for 8 hours, giving 62% product yield according to NMR analysis with CH_2Br_2 as standard. Purification by flash column chromatography with a mixture of hexane:EtOAc (100:0 to 90:10) giving 59% (12.6 mg) isolated yield as pale-yellow oil. 72% (70% isolated yield, 15.0 mg) obtained when 1.5 eq. **6a**, 2.1 eq. AgOTf, and 10 mol% MeDalphosAuCl were employed instead of the optimal conditions.

1 mmol scale

In a dry flask (100 mL), with a stirring bar, AgOTf (1.05 mmol, 1.05 eq.), $NaHCO_3$ (1 mmol, 1 eq.), MeDalphosAuCl (0.05 mmol, 5 mol%), *N*-phenylhex-5-enamide (**1a**, 1 mmol, 1 eq.) and **6a** (1 mmol, 1 eq.) are dissolved in 10 mL DCE under Ar atm. The mixture is stirred at 45 °C (oil bath) for 18 h. Then, the reaction is quenched with 20 mL of NH_4Cl sat. sol extracted with CH_2Cl_2 20 mL x 3 times. The organic phase is dried over dry $MgSO_4$, filtered and concentrated. Then, it is purified by flash column chromatography with a mixture of hexane:EtOAc (90:10 to 70:30) yielding 41% (88.5 mg) of **7aa**.

Spectra

^{1}H NMR (300 MHz, $CDCl_3$) δ 7.48 – 7.34 (m, 2H), 7.34 – 7.23 (m, 3H), 4.51 (ddt, *J* = 10.9, 7.8, 4.3 Hz, 1H), 2.90 (dd, *J* = 16.8, 4.7 Hz, 1H), 2.75 (dd, *J* = 16.8, 7.8 Hz, 1H), 2.68 – 2.58 (m, 1H), 2.55 – 2.40 (m, 1H), 2.28 – 2.11 (m, 1H), 2.06 – 1.85 (m, 2H), 1.83 – 1.61 (m, 1H) ppm.

^{13}C NMR (101 MHz, $CDCl_3$) δ 171.2 (s), 131.8 (s), 128.4 (s), 128.2 (s), 123.2 (s), 84.2 (s), 83.5 (s), 78.4 (s), 29.6 (s), 27.2 (s), 26.8 (s), 18.5 (s) ppm.

HRMS (ESI) m/z: $[M+Na]^+$ Calcd for $C_{14}H_{14}O_2Na$ 237.0886. Found 237.0885.

7aa-iminium. *N*-(6-(3-phenylprop-2-yn-1-yl)tetrahydro-2*H*-pyran-2-ylidene)benzenaminium trifluoromethanesulfonate

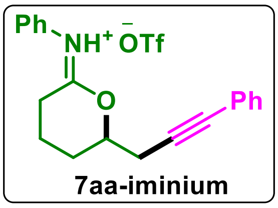

The compound was prepared according to the *GP3* using **1a** *N*-phenylhex-5-enamide (18.9 mg, 0.1 mmol, 1 eq.), **6a** (iodoethynyl)benzene (22.8 mg, 0.1 mmol, 1 eq.), AgOTf (27.0 mg, 0.105 mmol, 1.05 eq.), MeDalphosAuCl (3.2 mg, 0.005 mmol, 0.05 equiv.) in DCE (1 mL, 0.1 M). The mixture was heated at 45 °C (oil bath) for 8 hours, giving 32% product yield according to NMR analysis with CH_2Br_2 as standard (hydrolyse during the isolation in silica gel).

Spectra

^{1}H NMR (400 MHz, CD_2Cl_2) δ 12.23 (bs, 1H), 7.64 – 7.60 (m, 2H), 7.56 – 7.52 (m, 2H), 7.42 – 7.34 (m, 6H, 10H observed), 5.01 – 4.95 (m, 1H), 3.30 – 3.09 (m, 2H), 3.08 – 2.89 (m, 2H), 2.33 – 2.23 (m, 1H), 2.20 –2.12 (m, 1H), 2.03 – 1.84 (m, 2H) ppm.
^{13}C NMR (101 MHz, CD_2Cl_2) δ 176.1 (s), 132.9 (s), 132.0 (s), 129.8 (s), 129.4 (s), 128.8 (s), 123.6 (s), 123.0 (s), 85.4 (s), 81.9 (s), 74.0 (s), 26.9 (s), 26.7 (s), 26.3 (s), 16.2 (s) ppm.
^{19}F NMR (376 MHz, CD_2Cl_2) δ -79.82 ppm.
HRMS (ESI) m/z: $[M]^+$ Calcd for $C_{20}H_{20}NO$ 290.1539, Found 290.1539.

7ba. 7-(3-phenylprop-2-yn-1-yl)oxepan-2-one

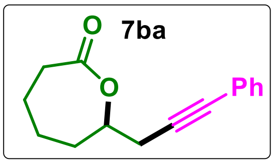

The compound was prepared according to the *GP3* using **1b** *N*-phenylhept-6-enamide (20.3 mg, 0.1 mmol, 1 eq.), **6a** (iodoethynyl)benzene (22.8 mg, 0.1 mmol, 1 eq.), $NaHCO_3$ (8.4 mg, 0.1 mmol, 1 eq.), AgOTf (27.0 mg, 0.105 mmol, 1.05 eq.), MeDalphosAuCl (3.2 mg, 0.005 mmol, 0.05 equiv.) in DCE (1 mL, 0.1 M). The mixture was heated at 45 °C (oil bath) for 18 hours, giving 41% product yield according to NMR analysis with CH_2Br_2 as standard. Purification by flash column chromatography with a mixture of hexane:EtOAc (100:0 to 90:10) giving 40% (9.2 mg) isolated yield as yellow oil.

Spectra
^{1}H NMR (300 MHz, $CDCl_3$) δ 7.50 – 7.36 (m, 2H), 7.33 – 7.25 (m, 3H), 4.52 – 4.39 (m, 1H), 2.88 (dd, *J* = 16.8, 5.1 Hz, 1H), 2.79 – 2.55 (m, 3H), 2.31 (d, *J* = 10.3 Hz, 1H), 2.06 – 1.93 (m, 2H), 1.65 (d, *J* = 9.1 Hz, 3H) ppm.
^{13}C NMR (75 MHz, $CDCl_3$) δ 175.0 (s), 131.8 (s), 128.4 (s), 128.2 (s), 123.3 (s), 85.1 (s), 78.8 (s), 77.4 (s), 35.1 (s), 33.8 (s), 28.4 (s), 27.3 (s), 23.1 (s) ppm.
HRMS (ESI) m/z: $[M+Na]^+$ Calcd for $C_{15}H_{16}O_2Na$ 251.1043. Found 251.1042.

7ca. 5-(3-phenylprop-2-yn-1-yl)dihydrofuran-2(3H)-one

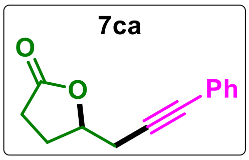

The compound was prepared according to the *GP3* using **1c** *N*-phenylpent-4-enamide (17.5 mg, 0.1 mmol, 1 eq.), **6a** (iodoethynyl)benzene (22.8 mg, 0.1 mmol, 1 eq.), $NaHCO_3$ (8.4 mg, 0.1 mmol, 1 eq.), AgOTf (27.0 mg, 0.105 mmol, 1.05 eq.), MeDalphosAuCl (3.2 mg, 0.005 mmol, 0.05 equiv.) in DCE (1 mL, 0.1 M). The mixture was heated at 45 °C (oil bath) for 18 hours, giving 16% product yield according to NMR analysis with CH_2Br_2 as standard. Purification by flash column chromatography with a mixture of hexane:EtOAc (100:0 to 90:10) giving 16% (3.2 mg) isolated yield as pale-yellow oil.

Spectra
^{1}H NMR (400 MHz, $CDCl_3$) δ 7.44 – 7.35 (m, 2H), 7.34 – 7.27 (m, 3H), 4.79 – 4.68 (m, 1H), 2.93 – 2.77 (m, 2H), 2.68 (ddd, *J* = 17.3, 9.9, 5.5 Hz, 1H), 2.62 – 2.38 (m, 2H), 2.21 (dddd, *J* = 12.9, 10.2, 7.6, 6.3 Hz, 1H) ppm.
^{13}C NMR (101 MHz, $CDCl_3$) δ 176.9 (s), 131.8 (s), 128.5 (s), 128.4 (s), 123.1 (s), 83.5 (s), 77.9 (s), 77.4 (s), 28.6 (s), 26.8 (s), 26.4 (s) ppm.
HRMS (ESI) m/z: $[M+Na]^+$ Calcd for $C_{13}H_{12}O_2Na$ 223.0730. Found 223.0729

7ja-1. (3*R*,5*R*)/(3*S*,5*S*)-3-phenyl-5-(3-phenylprop-2-yn-1-yl)dihydrofuran-2(3*H*)-one

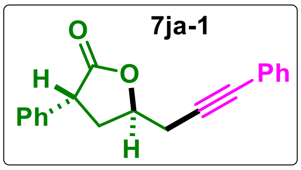

The compound was prepared according to the *GP3* using **1j** *N*,2-diphenylpent-4-enamide (25.1 mg, 0.1 mmol, 1 eq.), **6a** (iodoethynyl)benzene (22.8 mg, 0.1 mmol, 1 eq.), $NaHCO_3$ (8.4 mg, 0.1 mmol, 1 eq.), AgOTf (27.0 mg, 0.105 mmol, 1.05 eq.), MeDalphosAuCl (3.2 mg, 0.005 mmol, 0.05 equiv.) in DCE (1 mL, 0.1 M). The mixture was heated at 45 °C (oil bath) for 18 hours, giving 32% product yield

with 1:1 d.r. according to NMR analysis with CH_2Br_2 as standard. Purification by flash column chromatography with a mixture of hexane:EtOAc (100:0 to 85:15) giving 15% (4.2 mg) isolated for the *anti*-diastereoisomer **7ja-1** yield as yellow oil. Diastereoisomer attributed by comparison with **3ja-1.**

Spectra
^{1}H NMR (400 MHz, $CDCl_3$) δ 7.45 – 7.34 (m, 4H), 7.34 – 7.26 (m, 6H), 4.87 (dq, *J* = 7.7, 5.3 Hz, 1H), 4.17 – 4.02 (m, 1H), 2.92 (d, *J* = 5.6 Hz, 2H), 2.76 (ddd, *J* = 13.3, 9.8, 4.7 Hz, 1H), 2.63 (dt, *J* = 13.4, 7.9 Hz, 1H) ppm.
^{13}C NMR (101 MHz, $CDCl_3$) δ 177.0 (s), 137.3 (s), 131.8 (s), 129.2 (s), 128.5 (s), 128.5 (s), 127.8 (s), 127.8 (s), 123.0 (s), 83.8 (s), 83.6 (s), 76.0 (s), 45.6 (s), 35.6 (s), 26.3 (s) ppm.
HRMS (ESI) m/z: $[M+Na]^+$ Calcd for $C_{19}H_{16}O_2Na$ 299.1043. Found 299.1041.

7ja-2. (3*S*,5*R*)/(3*R*,5*S*)-3-phenyl-5-(3-phenylprop-2-yn-1-yl)dihydrofuran-2(3*H*)-one

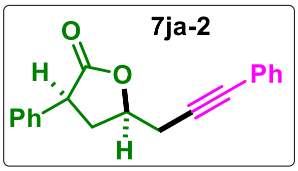

The compound was prepared according to the *GP3* using **1j** *N*,2-diphenylpent-4-enamide (25.1 mg, 0.1 mmol, 1 eq.), **6a** (iodoethynyl)benzene (22.8 mg, 0.1 mmol, 1 eq.), $NaHCO_3$ (8.4 mg, 0.1 mmol, 1 eq.), AgOTf (27.0 mg, 0.105 mmol, 1.05 eq.), MeDalphosAuCl (3.2 mg, 0.005 mmol, 0.05 equiv.) in DCE (1 mL, 0.1 M). The mixture was heated at 45 °C (oil bath) for 18 hours, giving 32% product yield with 1:1 d.r. according to NMR analysis with CH_2Br_2 as standard. Purification by flash column chromatography with a mixture of hexane:EtOAc (100:0 to 85:15) giving 16% (4.4 mg) isolated for the *syn* diastereoisomer **7ja-2** yield as yellow oil. Diastereoisomer attributed by comparison with **3ja-2**.

Spectra
^{1}H NMR (300 MHz, $CDCl_3$) δ 7.49 – 7.23 (m, 10H), 4.73 (dq, *J* = 10.8, 5.6 Hz, 1H), 3.94 (dd, *J* = 12.4, 9.1 Hz, 1H), 3.07 – 2.81 (m, 3H), 2.50 – 2.33 (m, 1H) ppm.
^{13}C NMR (101 MHz, $CDCl_3$) δ 176.5 (s), 136.7 (s), 131.9 (s), 129.1 (s), 128.5 (s), 128.4 (s), 128.3 (s), 127.9 (s), 123.1 (s), 83.9 (s), 83.3 (s), 75.7 (s), 47.2 (s), 36.9 (s), 26.0 (s) ppm.
HRMS (ESI) m/z: $[M+Na]^+$ Calcd for $C_{19}H_{16}O_2Na$ 299.1043. Found 299.1038.

7ea. 5-methyl-5-(3-phenylprop-2-yn-1-yl)dihydrofuran-2(3*H*)-one

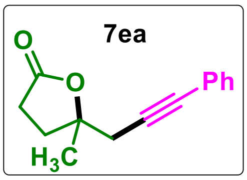

The compound was prepared according to the *GP3* using **1e** 4-methyl-*N*-phenylpent-4-enamide (18.9 mg, 0.1 mmol, 1 eq.), **6a** (iodoethynyl)benzene (22.8 mg, 0.1 mmol, 1 eq.), $NaHCO_3$ (8.4 mg, 0.1 mmol, 1 eq.), AgOTf (27.0 mg, 0.105 mmol, 1.05 eq.), MeDalphosAuCl (3.2 mg, 0.005 mmol, 0.05 equiv.) in DCE (1 mL, 0.1 M). The mixture was heated at 45 °C (oil bath) for 18 hours, giving 40% product yield according to NMR analysis with CH_2Br_2 as standard. Purification by flash column chromatography with a mixture of hexane:EtOAc (100:0 to 90:10) giving 36% (7.7 mg) isolated yield as yellow oil.

Spectra
^{1}H NMR (300 MHz, $CDCl_3$) δ 7.44 – 7.35 (m, 2H), 7.33 – 7.26 (m, 3H), 2.92 – 2.58 (m, 4H), 2.55 – 2.36 (m, 1H), 2.20 – 2.01 (m, 1H), 1.56 (s, 3H) ppm.
^{13}C NMR (75 MHz, $CDCl_3$) δ 176.5 (s), 131.8 (s), 128.5 (s), 128.4 (s), 123.1 (s), 85.1 (s), 84.4 (s), 83.5 (s), 32.4 (s), 29.5 (s), 26.6 (s) ppm.
HRMS (APCI) m/z: $[M+H]^+$ Calcd for $C_{14}H_{14}O_2H$ 215.1067. Found 215.1067.

7ab. 6-(3-(4-chlorophenyl)prop-2-yn-1-yl)tetrahydro-2H-pyran-2-one

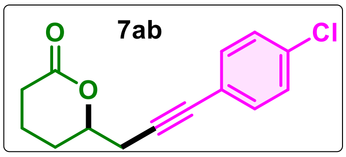

The compound was prepared according to the *GP3* using **1a** *N*-phenylhex-5-enamide (18.9 mg, 0.1 mmol, 1 eq.), **6b** 1-chloro-4-(iodoethynyl)benzene (26.3 mg, 0.1 mmol, 1 eq.), $NaHCO_3$ (8.4 mg, 0.1 mmol, 1 eq.), AgOTf (27.0 mg, 0.105 mmol, 1.05 eq.), MeDalphosAuCl (3.2 mg, 0.005 mmol, 0.05 equiv.) in DCE (1 mL, 0.1 M). The mixture was heated at 45 °C (oil bath) for 18 hours, giving 59% product yield according to NMR analysis with CH_2Br_2 as standard. Purification by flash column chromatography with a mixture of hexane:EtOAc (100:0 to 90:10) giving 58% (14.4 mg) isolated yield as yellow oil.

Spectra

^{1}H NMR (300 MHz, $CDCl_3$) δ 7.32 (d, *J* = 8.4 Hz, 2H), 7.26 (d, *J* = 8.4 Hz, 2H), 4.50 (ddt, *J* = 10.9, 7.9, 4.0 Hz, 1H), 2.81 (qd, *J* = 16.9, 6.1 Hz, 2H), 2.60 (t, *J* = 6.0 Hz, 1H), 2.56 – 2.37 (m, 1H), 2.16 (dd, *J* = 13.6, 4.2 Hz, 1H), 2.07 – 1.82 (m, 2H), 1.82 – 1.64 (m, 1H) ppm.

^{13}C NMR (75 MHz, $CDCl_3$) δ 171.1 (s), 134.3 (s), 133.0 (s), 128.7 (s), 121.7 (s), 85.3 (s), 82.4 (s), 78.3 (s), 29.6 (s), 27.2 (s), 26.8 (s), 18.5 (s) ppm.

HRMS (ESI) m/z: $[M+Na]^+$ Calcd for $C_{14}H_{13}ClO_2Na$ 271.0496. Found 271.0497.

7ac. 6-(3-(4-fluorophenyl)prop-2-yn-1-yl)tetrahydro-2*H*-pyran-2-one

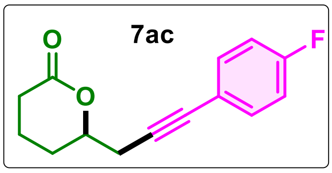

The compound was prepared according to the *GP3* using **1a** *N*-phenylhex-5-enamide (18.9 mg, 0.1 mmol, 1 eq.), **6c** 1-fluoro-4-(iodoethynyl)benzene (24.6 mg, 0.1 mmol, 1 eq.), $NaHCO_3$ (8.4 mg, 0.1 mmol, 1 eq.), AgOTf (27.0 mg, 0.105 mmol, 1.05 eq.), MeDalphosAuCl (3.2 mg, 0.005 mmol, 0.05 equiv.) in DCE (1 mL, 0.1 M). The mixture was heated at 45 °C (oil bath) for 18 hours, giving 41% product yield according to NMR analysis with CH_2Br_2 as standard. Purification by flash column chromatography with a mixture of hexane:EtOAc (100:0 to 90:10) giving 41% (9.4 mg) isolated yield as yellow oil.

Spectra

^{1}H NMR (300 MHz, $CDCl_3$) δ 7.37 (dd, *J* = 8.4, 5.5 Hz, 2H), 6.98 (t, *J* = 8.6 Hz, 2H), 4.50 (ddt, *J* = 10.5, 7.6, 4.0 Hz, 1H), 2.97 – 2.69 (m, 2H), 2.69 – 2.38 (m, 2H), 2.23 – 2.10 (m, 1H), 2.09 – 1.63 (m, 3H) ppm.

^{13}C NMR (75 MHz, $CDCl_3$) δ 171.1 (s), 162.5 (d, *J* = 249.2 Hz), 133.7 (d, *J* = 8.3 Hz), 119.4 (s), 115.7 (d, *J* = 22.1 Hz), 83.9 (s), 82.4 (s), 78.3 (s), 29.6 (s), 27.2 (s), 26.8 (s), 18.5 (s) ppm.

^{19}F NMR (282 MHz, $CDCl_3$) δ -111.27 ppm.

HRMS (ESI) m/z: $[M+Na]^+$ Calcd for $C_{14}H_{13}FO_2Na$ 255.0792. Found 255.079.

7ad. 6-(3-(2-fluorophenyl)prop-2-yn-1-yl)tetrahydro-2*H*-pyran-2-one

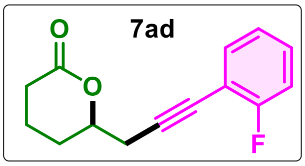

The compound was prepared according to the *GP3* using **1a** *N*-phenylhex-5-enamide (18.9 mg, 0.1 mmol, 1 eq.), **6d** 1-fluoro-2-(iodoethynyl)benzene (23.6 mg, 0.1 mmol, 1 eq.), $NaHCO_3$ (8.4 mg, 0.1 mmol, 1 eq.), AgOTf (27.0 mg, 0.105 mmol, 1.05 eq.), MeDalphosAuCl (3.2 mg, 0.005 mmol, 0.05 equiv.) in DCE (1 mL, 0.1 M). The mixture was heated at 45 °C (oil bath) for 18 hours, giving 65% product yield according to NMR analysis with CH_2Br_2 as standard. Purification by flash column chromatography with a mixture of hexane:EtOAc (100:0 to 90:10) giving 62% (14.4 mg) isolated yield as pale-yellow oil.

Spectra

^{1}H NMR (400 MHz, $CDCl_3$) δ 7.39 (t, *J* = 7.4 Hz, 1H), 7.34 – 7.15 (m, 1H), 7.11 – 7.00 (m, 2H), 4.53 (dddd, *J* = 10.9, 7.8, 4.6, 3.4 Hz, 1H), 2.93 (dd, *J* = 16.9, 4.6 Hz, 1H), 2.80 (dd, *J* = 16.9, 7.8 Hz, 1H), 2.69 – 2.53 (m, 1H), 2.48 (dd, *J* = 18.9, 8.1 Hz, 1H), 2.21 (dd, *J* = 13.6, 4.6 Hz, 1H), 2.04 – 1.97 (m, 1H), 1.97 – 1.86 (m, 1H), 1.86 – 1.71 (m, 1H) ppm.
^{13}C NMR (101 MHz, $CDCl_3$) δ 171.1 (s), 163.1 (d, *J* = 250.8 Hz), 133.7 (d, *J* = 1.4 Hz), 129.9 (d, *J* = 8.0 Hz), 124.0 (d, *J* = 3.7 Hz), 115.6 (d, *J* = 20.9 Hz), 111.8 (d, *J* = 15.6 Hz), 89.7 (d, *J* = 3.4 Hz), 78.3 (s), 76.9 (s), 29.4 (s), 27.1 (s), 27.0 (s), 18.5 (s) ppm.
^{19}F NMR (376 MHz, $CDCl_3$) δ -111.68 ppm.
HRMS (ESI) m/z: $[M+Na]^+$ Calcd for $C_{14}H_{13}FO_2Na$ 255.0792. Found 255.0793.

7ae. 6-(3-(3-fluorophenyl)prop-2-yn-1-yl)tetrahydro-2*H*-pyran-2-one

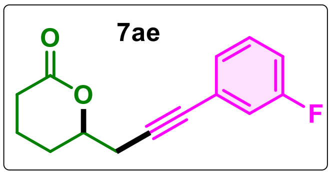

The compound was prepared according to the *GP3* using **1a** *N*-phenylhex-5-enamide (18.9 mg, 0.1 mmol, 1 eq.), **6e** 1-fluoro-3-(iodoethynyl)benzene (24.6 mg, 0.1 mmol, 1 eq.), $NaHCO_3$ (8.4 mg, 0.1 mmol, 1 eq.), AgOTf (27.0 mg, 0.105 mmol, 1.05 eq.), MeDalphosAuCl (3.2 mg, 0.005 mmol, 0.05 equiv.) in DCE (1 mL, 0.1 M). The mixture was heated at 45 °C (oil bath) for 18 hours, giving 61% product yield according to NMR analysis with CH_2Br_2 as standard. Purification by flash column chromatography with a mixture of hexane:EtOAc (100:0 to 90:10) giving 57% (13.3 mg) isolated yield as yellow oil.

Spectra
^{1}H NMR (400 MHz, $CDCl_3$) δ 7.25 (td, *J* = 8.0, 5.8 Hz, 1H), 7.17 (dt, *J* = 7.7, 1.3 Hz, 1H), 7.09 (dt, *J* = 9.5, 2.1 Hz, 1H), 7.05 – 6.96 (m, 1H), 4.51 (dddd, *J* = 10.8, 7.8, 4.8, 3.3 Hz, 1H), 2.88 (dd, *J* = 16.9, 4.8 Hz, 1H), 2.76 (dd, *J* = 16.9, 7.6 Hz, 1H), 2.63 (dt, *J* = 17.8, 6.6 Hz, 1H), 2.55 – 2.39 (m, 1H), 2.23 – 2.11 (m, 1H), 2.07 – 1.94 (m, 1H), 1.94 – 1.80 (m, 1H), 1.80 – 1.67 (m, 1H) ppm.
^{13}C NMR (75 MHz, $CDCl_3$) δ 171.1 (s), 162.5 (d, *J* = 246.4 Hz), 130.0 (d, *J* = 8.7 Hz), 127.7 (d, *J* = 3.1 Hz), 125.1 (d, *J* = 9.5 Hz), 118.6 (d, *J* = 22.7 Hz), 115.6 (d, *J* = 21.2 Hz), 85.4 (s), 82.3 (d, *J* = 3.4 Hz), 78.2 (s), 29.8 (s), 27.2 (s), 26.8 (s), 18.5 (s) ppm.
^{19}F NMR (376 MHz, $CDCl_3$) δ -114.01 ppm.
HRMS (ESI) m/z: $[M+H]^+$ Calcd for $C_{14}H_{13}FO_2H$ 233.0972. Found 233.0972.

7af. 6-(3-(4-(trifluoromethyl)phenyl)prop-2-yn-1-yl)tetrahydro-2*H*-pyran-2-one

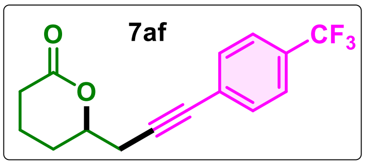

The compound was prepared according to the *GP3* using **1a** *N*-phenylhex-5-enamide (18.9 mg, 0.1 mmol, 1 eq.), **6f** 1-(iodoethynyl)-4-(trifluoromethyl)benzene (29.6 mg, 0.1 mmol, 1 eq.), $NaHCO_3$ (8.4 mg, 0.1 mmol, 1 eq.), AgOTf (27.0 mg, 0.105 mmol, 1.05 eq.), MeDalphosAuCl (3.2 mg, 0.005 mmol, 0.05 equiv.) in DCE (1 mL, 0.1 M). The mixture was heated at 45 °C (oil bath) for 18 hours, giving 50% product yield according to NMR analysis with CH_2Br_2 as standard. Purification by flash column chromatography with a mixture of hexane:EtOAc (100:0 to 90:10) giving 48% (13.5 mg) isolated yield as yellow oil.

Spectra
^{1}H NMR (400 MHz, $CDCl_3$) δ 7.58 (d, *J* = 8.3 Hz, 2H), 7.52 (d, *J* = 8.3 Hz, 2H), 4.60 – 4.49 (m, 1H), 2.93 (dd, *J* = 16.9, 4.9 Hz, 1H), 2.82 (dd, *J* = 17.0, 7.4 Hz, 1H), 2.72 – 2.60 (m, 1H), 2.59 – 2.43 (m, 1H), 2.19 (dd, *J* = 13.7, 4.6 Hz, 1H), 2.11 – 2.00 (m, 1H), 2.00 – 1.87 (m, 1H), 1.84 – 1.68 (m, 1H) ppm.

^{13}**C NMR** (101 MHz, $CDCl_3$) δ 171.0 (s), 132.1 (s), 130.1 (d, *J* = 32.6 Hz), 127.1 (s), 125.4 (q, *J* = 3.9 Hz), 124.8 (q, *J* = 271.6 Hz), 87.0 (s), 82.3 (s), 78.1 (s), 29.6 (s), 27.2 (s), 26.9 (s), 18.5 (s) ppm.
^{19}**F NMR** (376 MHz, $CDCl_3$) δ -63.80 ppm.
HRMS (ESI) m/z: $[M+Na]^+$ Calcd for $C_{15}H_{13}F_3O_2Na$ 305.0760. Found 305.0759.

7ag. 6-(3-(4-nitrophenyl)prop-2-yn-1-yl)tetrahydro-2*H*-pyran-2-one

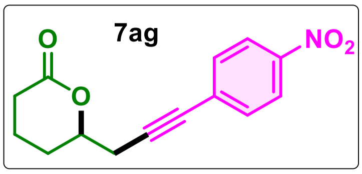

The compound was prepared according to the *GP3* using **1a** *N*-phenylhex-5-enamide (18.9 mg, 0.1 mmol, 1 eq.), **6g** 1-(iodoethynyl)-4-nitrobenzene (27.3 mg, 0.1 mmol, 1 eq.), $NaHCO_3$ (8.4 mg, 0.1 mmol, 1 eq.), AgOTf (27.0 mg, 0.105 mmol, 1.05 eq.), MeDalphosAuCl (3.2 mg, 0.005 mmol, 0.05 equiv.) in DCE (1 mL, 0.1 M). The mixture was heated at 45 °C (oil bath) for 18 hours, giving 42% product yield according to NMR analysis with CH_2Br_2 as standard. Purification by flash column chromatography with a mixture of hexane:EtOAc (100:0 to 80:20) giving 41% (10.6 mg) isolated yield as pale-yellow solid.

Spectra
^{1}**H NMR** (400 MHz, $CDCl_3$) δ 8.17 (d, *J* = 8.9 Hz, 2H), 7.54 (d, *J* = 8.9 Hz, 2H), 4.59 – 4.48 (m, 1H), 2.87 (qd, *J* = 17.1, 6.0 Hz, 2H), 2.72 – 2.59 (m, 1H), 2.57 – 2.44 (m, 1H), 2.16 (dq, *J* = 13.8, 4.6 Hz, 1H), 2.07 – 1.96 (m, 1H), 1.97 – 1.84 (m, 1H), 1.75 (ddd, *J* = 24.6, 10.9, 5.2 Hz, 1H) ppm.
^{13}**C NMR** (101 MHz, $CDCl_3$) δ 170.9 (s), 147.2 (s), 132.6 (s), 130.2 (s), 123.7 (s), 90.2 (s), 81.9 (s), 77.9 (s), 29.6 (s), 27.3 (s), 27.0 (s), 18.5 (s) ppm.
HRMS (ESI) m/z: $[M+Na]^+$ Calcd for $C_{14}H_{13}NO_4Na$ 282.0737. Found 282.0736.
Melting point: 135-145 °C.

7ah. 4-(3-(6-oxotetrahydro-2*H*-pyran-2-yl)prop-1-yn-1-yl)benzonitrile

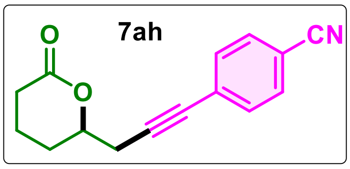

The compound was prepared according to the *GP3* using **1a** *N*-phenylhex-5-enamide (18.9 mg, 0.1 mmol, 1 eq.), **6h** 4-(iodoethynyl)benzonitrile (25.3 mg, 0.1 mmol, 1 eq.), $NaHCO_3$ (8.4 mg, 0.1 mmol, 1 eq.), AgOTf (27.0 mg, 0.105 mmol, 1.05 eq.), MeDalphosAuCl (3.2 mg, 0.005 mmol, 0.05 equiv.) in DCE (1 mL, 0.1 M). The mixture was heated at 45 °C (oil bath) for 18 hours, giving 79% product yield according to NMR analysis with CH_2Br_2 as standard. Purification by flash column chromatography with a mixture of hexane:EtOAc (100:0 to 90:10) giving 75% (17.9 mg) isolated yield as yellow oil.

Spectra
^{1}**H NMR** (300 MHz, $CDCl_3$) δ 7.58 (d, *J* = 8.3 Hz, 2H), 7.47 (d, *J* = 8.3 Hz, 2H), 4.59 – 4.45 (m, 1H), 2.97 – 2.74 (m, 2H), 2.64 (dt, *J* = 18.6, 6.1 Hz, 1H), 2.56 – 2.41 (m, 1H), 2.14 (dt, *J* = 13.0, 3.6 Hz, 1H), 2.05 – 1.84 (m, 2H), 1.84 – 1.67 (m, 1H) ppm.
^{13}**C NMR** (75 MHz, $CDCl_3$) δ 170.9 (s), 132.4 (s), 132.1 (s), 128.2 (s), 118.6 (s), 111.7 (s), 89.3 (s), 82.1 (s), 78.0 (s), 29.6 (s), 27.3 (s), 26.9 (s), 18.5 (s) ppm.
HRMS (ESI) m/z: $[M+Na]^+$ Calcd for $C_{15}H_{13}NO_2Na$ 262.0838. Found 262.0835.

7ai. 6-(3-(p-tolyl)prop-2-yn-1-yl)tetrahydro-2*H*-pyran-2-one

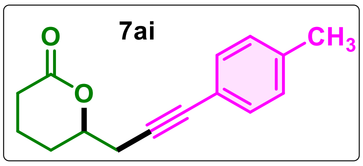

The compound was prepared according to the *GP3* using **1a** *N*-phenylhex-5-enamide (18.9 mg, 0.1 mmol, 1 eq.), **6i** 1-(iodoethynyl)-4-methylbenzene (24.2 mg, 0.1 mmol, 1 eq.), $NaHCO_3$ (8.4 mg, 0.1 mmol, 1 eq.), AgOTf (27.0 mg, 0.105 mmol, 1.05 eq.), MeDalphosAuCl (3.2 mg, 0.005 mmol, 0.05 equiv.) in DCE (1 mL, 0.1 M). The mixture was heated at 45 °C (oil bath) for 18 hours, giving 40% product yield according to NMR analysis with CH_2Br_2 as standard. Purification by flash column chromatography with a mixture of hexane:EtOAc (100:0 to 90:10) giving 35% (7.9 mg) isolated yield as pale-yellow oil.

Spectra

^{1}H NMR (300 MHz, $CDCl_3$) δ 7.29 (d, *J* = 8.1 Hz, 2H), 7.10 (d, *J* = 8.1 Hz, 2H), 4.57 – 4.43 (m, 1H), 2.89 (dd, *J* = 16.8, 4.6 Hz, 1H), 2.74 (dd, *J* = 16.9, 7.9 Hz, 1H), 2.62 (dt, *J* = 17.9, 6.0 Hz, 1H), 2.57 – 2.39 (m, 1H), 2.34 (s, 3H), 2.19 (dq, *J* = 8.8, 4.5 Hz, 1H), 2.08 – 1.82 (m, 2H), 1.82 – 1.62 (m, 1H) ppm.
^{13}C NMR (101 MHz, $CDCl_3$) δ 171.2 (s), 138.3 (s), 131.7 (s), 129.2 (s), 120.2 (s), 83.5 (s), 83.4 (s), 78.5 (s), 29.6 (s), 27.2 (s), 26.8 (s), 21.6 (s), 18.5 (s) ppm.
HRMS (ESI) m/z: $[M+H]^+$ Calcd for $C_{15}H_{16}O_2H$ 229.1223. Found 229.1221.

7aj. 6-(3-(m-tolyl)prop-2-yn-1-yl)tetrahydro-2*H*-pyran-2-one

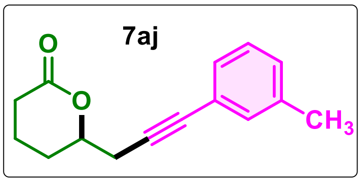

The compound was prepared according to the *GP3* using **1a** *N*-phenylhex-5-enamide (18.9 mg, 0.1 mmol, 1 eq.), **6j** 1-(iodoethynyl)-3-methylbenzene (24.2 mg, 0.1 mmol, 1 eq.), $NaHCO_3$ (8.4 mg, 0.1 mmol, 1 eq.), AgOTf (27.0 mg, 0.105 mmol, 1.05 eq.), MeDalphosAuCl (3.2 mg, 0.005 mmol, 0.05 equiv.) in DCE (1 mL, 0.1 M). The mixture was heated at 45 °C (oil bath) for 18 hours, giving 51% product yield according to NMR analysis with CH_2Br_2 as standard. Purification by flash column chromatography with a mixture of hexane:EtOAc (100:0 to 90:10) giving 51% (11.6 mg) isolated yield as pale-yellow oil.

Spectra

^{1}H NMR (400 MHz, $CDCl_3$) δ 7.24 – 7.15 (m, 3H), 7.14 – 7.06 (m, 1H), 4.50 (dddd, *J* = 10.9, 7.9, 4.6, 3.4 Hz, 1H), 2.89 (dd, *J* = 16.8, 4.6 Hz, 1H), 2.75 (dd, *J* = 16.8, 7.9 Hz, 1H), 2.63 (dddd, *J* = 17.8, 6.9, 5.0, 1.2 Hz, 1H), 2.49 (ddd, *J* = 17.8, 9.1, 7.1 Hz, 1H), 2.32 (s, 3H), 2.25 – 2.14 (m, 1H), 2.06 – 1.94 (m, 1H), 1.94 – 1.81 (m, 1H), 1.81 – 1.65 (m, 1H) ppm.
^{13}C NMR (101 MHz, $CDCl_3$) δ 171.2 (s), 138.1 (s), 132.4 (s), 129.1 (s), 128.8 (s), 128.3 (s), 123.0 (s), 83.8 (s), 83.6 (s), 78.5 (s), 29.6 (s), 27.2 (s), 26.8 (s), 21.3 (s), 18.5 (s) ppm.
HRMS (ESI) m/z: $[M+H]^+$ Calcd for $C_{15}H_{16}O_2H$ 229.1223. Found 229.1222.

7ak. 6-(3-(3,5-bis(trifluoromethyl)phenyl)prop-2-yn-1-yl)tetrahydro-2*H*-pyran-2-one

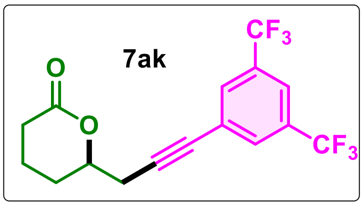

The compound was prepared according to the *GP3* using **1a** *N*-phenylhex-5-enamide (18.9 mg, 0.1 mmol, 1 eq.), **6k** 1-(iodoethynyl)-3,5-bis(trifluoromethyl)benzene (54.6 mg, 0.1 mmol, 1 eq.), $NaHCO_3$ (8.4 mg, 0.1 mmol, 1 eq.), AgOTf (27.0 mg, 0.105 mmol, 1.05 eq.), MeDalphosAuCl (3.2 mg, 0.005 mmol, 0.05 equiv.) in DCE (1 mL, 0.1 M). The mixture was heated at 45 °C (oil bath) for 18 hours, giving 45% product yield according to NMR analysis with CH_2Br_2 as standard. Purification by flash column chromatography with a mixture of hexane:EtOAc (100:0 to 90:10) giving 43% (15.1 mg) isolated yield as yellow oil.

Spectra
^{1}H NMR (400 MHz, $CDCl_3$) δ 7.83 (s, 2H), 7.79 (s, 1H), 4.58 – 4.47 (m, 1H), 2.91 (dd, *J* = 17.1, 5.1 Hz, 1H), 2.81 (dd, *J* = 17.0, 7.1 Hz, 1H), 2.65 (dt, *J* = 17.9, 6.5 Hz, 1H), 2.51 (ddd, *J* = 17.9, 9.2, 7.1 Hz, 1H), 2.24 – 2.11 (m, 1H), 2.11 – 1.96 (m, 1H), 1.98 – 1.86 (m, 1H), 1.87 – 1.69 (m, 1H) ppm.
^{13}C{^{1}H} NMR (75 MHz, $CDCl_3$) δ 170.9 (s), 132.0 (q, *J* = 33.7 Hz), 131.8 (s), 125.6 (s), 123.3 (q, *J* = 338.3 Hz), 121.7 (d, *J* = 4.0 Hz), 88.4 (s), 80.8 (s), 77.9 (s), 29.5 (s), 27.3 (s), 26.8 (s), 18.5 (s) ppm.
^{19}F NMR (282 MHz, $CDCl_3$) δ -63.16 ppm.
HRMS (APCI) m/z: $[M+K]^+$ Calcd for $C_{16}H_{12}F_6O_2K$ 389.0373. Found 389.0375.

7al. 6-(3-(3-fluoro-4-(trifluoromethoxy)phenyl)prop-2-yn-1-yl)tetrahydro-2*H*-pyran-2-one

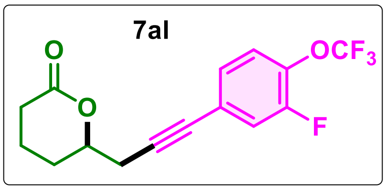

The compound was prepared according to the *GP3* using **1a** *N*-phenylhex-5-enamide (18.9 mg, 0.1 mmol, 1 eq.), **6l** 2-fluoro-4-(iodoethynyl)-1-(trifluoromethoxy)benzene (49.5 mg, 0.1 mmol, 1 eq.), $NaHCO_3$ (8.4 mg, 0.1 mmol, 1 eq.), AgOTf (27.0 mg, 0.105 mmol, 1.05 eq.), MeDalphosAuCl (3.2 mg, 0.005 mmol, 0.05 equiv.) in DCE (1 mL, 0.1 M). The mixture was heated at 45 °C (oil bath) for 18 hours, giving 53% product yield according to NMR analysis with CH_2Br_2 as standard. Purification by flash column chromatography with a mixture of hexane:EtOAc (100:0 to 90:10) giving 53% (16.8 mg) isolated yield as yellow oil.

Spectra
^{1}H NMR (300 MHz, $CDCl_3$) δ ^{1}H NMR (400 MHz, $CDCl_3$) δ 7.25 – 7.16 (m, 3H), 4.50 (td, *J* = 10.6, 5.9 Hz, 1H), 2.87 (dd, *J* = 16.9, 5.0 Hz, 1H), 2.77 (dd, *J* = 16.9, 7.2 Hz, 1H), 2.70 – 2.57 (m, 1H), 2.49 (ddd, *J* = 17.9, 9.3, 7.1 Hz, 1H), 2.15 (dq, *J* = 14.0, 4.6 Hz, 1H), 2.06 – 1.95 (m, 1H), 1.96 – 1.85 (m, 1H), 1.79 – 1.65 (m, 1H) ppm.
^{13}C NMR (101 MHz, $CDCl_3$) δ 171.0 (s), 154.1 (d, *J* = 253.5 Hz), 136.6 (d, *J* = 11.1 Hz), 128.3 (d, *J* = 3.8 Hz), 123.8 (s), 123.7 (s), 120.6 (d, *J* = 20.0 Hz), 120.5 (d, *J* = 259.2 Hz), 86.5 (s), 81.2 (d, *J* = 2.8 Hz), 78.0 (s), 29.6 (s), 27.2 (s), 26.8 (s), 18.5 (s) ppm.
^{19}F NMR (376 MHz, $CDCl_3$) δ -59.73 (d, *J* = 4.8 Hz), -129.38 (q, *J* = 4.8 Hz) ppm.
HRMS (APCI) m/z: $[M+K]^+$ Calcd for $C_{15}H_{12}F_4O_3K$ 355.0354. Found 355.0356.

7am. 6-(5-phenylpenta-2,4-diyn-1-yl)tetrahydro-2*H*-pyran-2-one

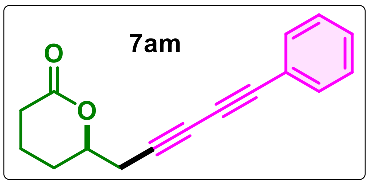

The compound was prepared according to the *GP3* using **1a** *N*-phenylhex-5-enamide (18.9 mg, 0.1 mmol, 1 eq.), **6m** (iodobuta-1,3-diyn-1-yl)benzene (25.2 mg, 0.1 mmol, 1 eq.), $NaHCO_3$ (8.4 mg, 0.1 mmol, 1 eq.), AgOTf (27.0 mg, 0.105 mmol, 1.05 eq.), MeDalphosAuCl (3.2 mg, 0.005 mmol, 0.065 equiv.) in DCE (1 mL, 0.1 M). The mixture was heated at 45 °C (oil bath) for 18 hours, giving 28% product yield according to NMR analysis with CH_2Br_2 as standard. Purification by flash column chromatography with a mixture of hexane:EtOAc (100:0 to 90:10) giving 26% (6.3 mg) isolated yield as pale-yellow oil.

Spectra
^{1}H NMR (400 MHz, $CDCl_3$) δ 7.48 (dd, *J* = 8.1, 1.6 Hz, 2H), 7.39 – 7.28 (m, 3H), 4.47 (dddd, *J* = 10.8, 7.8, 4.8, 3.3 Hz, 1H), 2.85 (dd, *J* = 17.3, 4.7 Hz, 1H), 2.72 (dd, *J* = 17.3, 7.6 Hz, 1H), 2.69 – 2.55 (m, 1H), 2.50 (ddd, *J* = 17.8, 9.3, 7.1 Hz, 1H), 2.21 – 2.11 (m, 1H), 2.04 – 1.94 (m, 1H), 1.94 – 1.82 (m, 1H), 1.82 – 1.64 (m, 1H) ppm.
^{13}C NMR (101 MHz, $CDCl_3$) δ 170.8 (s), 132.7 (s), 129.3 (s), 128.6 (s), 121.7 (s), 78.3 (s), 77.9 (s), 76.0 (s), 73.9 (s), 68.1 (s), 29.6 (s), 27.2 (s), 27.0 (s), 18.5 (s) ppm.
HRMS (APCI) m/z: $[M+H]^+$ Calcd for $C_{16}H_{14}O_2H$ 239.1067. Found 239.1066.

7an. 6-(non-2-yn-1-yl)tetrahydro-2*H*-pyran-2-one

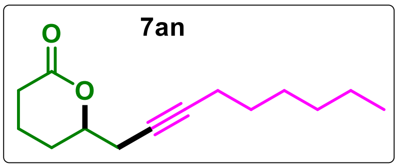

The compound was prepared according to the *GP3* using **1a** *N*-phenylhex-5-enamide (18.9 mg, 0.1 mmol, 1 eq.), **6n** 1-iodooct-1-yne (23.6 mg, 0.1 mmol, 1 eq.), $NaHCO_3$ (8.4 mg, 0.1 mmol, 1 eq.), AgOTf (27.0 mg, 0.105 mmol, 1.05 eq.), MeDalphosAuCl (3.2 mg, 0.005 mmol, 0.05 equiv.) in DCE (1 mL, 0.1 M). The mixture was heated at 45 °C (oil bath) for 18 hours, giving 24% product yield according to NMR analysis with CH_2Br_2 as standard. Purification by flash column chromatography with a mixture of hexane:EtOAc (100:0 to 80:20) giving 23% (5.2 mg) isolated yield as yellow oil.

Spectra

^{1}H NMR (400 MHz, $CDCl_3$) δ 4.38 (ddt, *J* = 11.3, 8.0, 4.2 Hz, 1H), 2.73 – 2.56 (m, 2H), 2.53 – 2.34 (m, 2H), 2.22 – 2.05 (m, 2H), 2.02 – 1.74 (m, 2H), 1.76 – 1.58 (m, 1H), 1.53 – 1.42 (m, 2H), 1.42 – 1.18 (m, 7H), 0.89 (t, *J* = 6.9 Hz, 3H) ppm.
^{13}C NMR (75 MHz, $CDCl_3$) δ 171.3 (s), 83.7 (s), 78.9 (s), 74.4 (s), 31.5 (s), 29.7 (s), 29.0 (s), 28.7 (s), 27.0 (s), 26.2 (s), 22.7 (s), 18.9 (s), 18.5 (s), 14.2 (s) ppm.
HRMS (APCI) m/z: $[M+Na]^+$ Calcd for $C_{14}H_{22}O_2Na$ 245.1512. Found 245.1516

7ao. 6-(hept-2-yn-1-yl)tetrahydro-2*H*-pyran-2-one

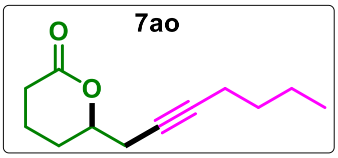

The compound was prepared according to the *GP3* using **1a** *N*-phenylhex-5-enamide (18.9 mg, 0.1 mmol, 1 eq.), **6o** 1-iodohex-1-yne (20.8 mg, 0.1 mmol, 1 eq.), $NaHCO_3$ (8.4 mg, 0.1 mmol, 1 eq.), AgOTf (27.0 mg, 0.105 mmol, 1.05 eq.), MeDalphosAuCl (3.2 mg, 0.005 mmol, 0.05 equiv.) in DCE (1 mL, 0.1 M). The mixture was heated at 45 °C (oil bath) for 18 hours, giving 37% product yield according to NMR analysis with CH_2Br_2 as standard. Purification by flash column chromatography with a mixture of hexane:EtOAc (100:0 to 80:20) giving 34% (6.6 mg) isolated yield as colorless oil.

Spectra

^{1}H NMR (400 MHz, $CDCl_3$) δ 4.39 – 4.24 (m, 1H), 2.74 – 2.61 (m, 2H), 2.41 (t, *J* = 7.4 Hz, 2H), 2.10 – 1.88 (m, 4H), 1.88 – 1.76 (m, 2H), 1.56 (dt, *J* = 15.2, 7.5 Hz, 2H), 1.35 – 1.27 (m, 2H), 0.90 (t, *J* = 7.3 Hz, 3H) ppm.
^{13}C NMR (101 MHz, $CDCl_3$) δ 171.8 (s), 79.6 (s), 77.4 (s), 42.9 (s), 37.8 (s), 29.7 (s), 28.3 (s), 26.1 (s), 22.5 (s), 18.6 (s), 14.0 (s) ppm.
HRMS (ESI) m/z: $[M+H]^+$ Calcd for $C_{12}H_{18}O_2H$ 195.1380. Found 195.1378.

7. XRD Data

X-Ray crystal structure determination

Single crystals of **3ja-1**, **5fa** and **Au2** (MeCagephosAuCl) were selected, mounted onto a cryoloop and transferred into a cold nitrogen gas stream. Intensity data were collected with a Bruker Kappa-APEX2 CCD diffractometer using a micro-focused CuKα radiation (for **3ja-1** and **5fa**) or a graphite-monochromated MoKα radiation (**Au2**). Data collections, unit-cell parameters determinations, integration and data reductions were performed with the Bruker APEX/SAINT[65] suite at 200K. The structures were solved with SHELXT[66] and refined anisotropically by full-matrix least-squares methods with SHELXL[67], using Olex2[68] software (except H atoms). Then, Olex2.refine[69] software enables the use of ORCA 5.0[70] quantum chemistry software, using NoSpherA2[71] tool, so as to improve electron density simulation. Thus, H atoms are located freely.

The structures were deposited at the Cambridge Crystallographic Data Centre with numbers CCDC and can be obtained free of charge via www.ccdc.cam.ac.uk.

Crystal data 3ja-1. CCDC 2534751 . $C_{18}H_{18}O_3$ (M =282.342 g/mol): monoclinic, space group $P2_1/n$ (no. 14), a = 15.7165(9) Å, b = 5.6118(4) Å, c = 16.4882(12) Å, $β$ = 91.091(4) ,V= 1453.96(17) Å^3, Z = 4, T = 200.39 K, μ(Cu Kα) = 0.699 mm^{-1}, $Dcalc$ = 1.290 g/cm^3, 12094 reflections measured (3.85 ≤ 2Θ ≤ 66.55), 2466 unique (R_{int} = 0.0814, R_{sigma} = 0.0625) which were used in all calculations. The final R_1 was 0.8568 (I>=2u(I)) and wR_2 was 0.0808 (all data).

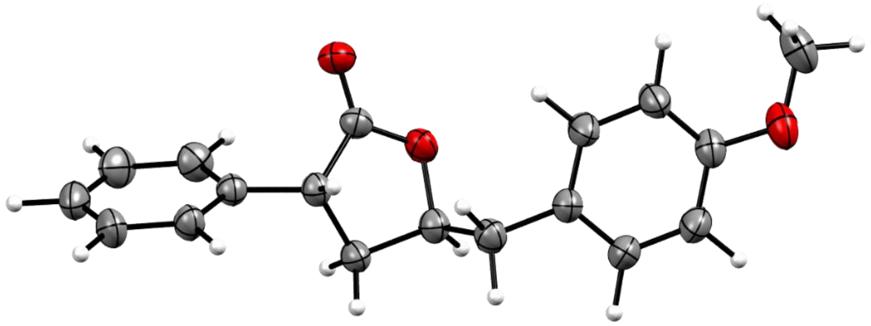

Figure S8. ORTEP representation of **3ja-1**. All atoms except hydrogen were drawn at 30% probability. Atom labelling: carbon: grey; oxygen: red; and hydrogen: white.

Crystal data 5fa. CCDC 2534752. $C_{15}H_{16}O_2$ (*M* =228.293 g/mol): triclinic, space group P_{-1} (no. 2), *a* = 9.0003(3) Å, *b* = 11.9830(4) Å, *c* = 13.0809(5) Å, *β* = 99.812(2), *V* = 1232.83(8) Å^3, *Z* = 4, *T* = 200.41 K, μ(Cu Kα) = 0.638 mm^{-1}, *Dcalc* = 1.230 g/cm^3, 18629 reflections measured (3.86 ≤ 2Θ ≤ 66.86), 4376 unique (R_{int} = 0.0368, R_{sigma} = 0.0317) which were used in all calculations. The final R_1 was 0.0817 (I>=2u(I)) and wR_2 was 0.0855 (all data).

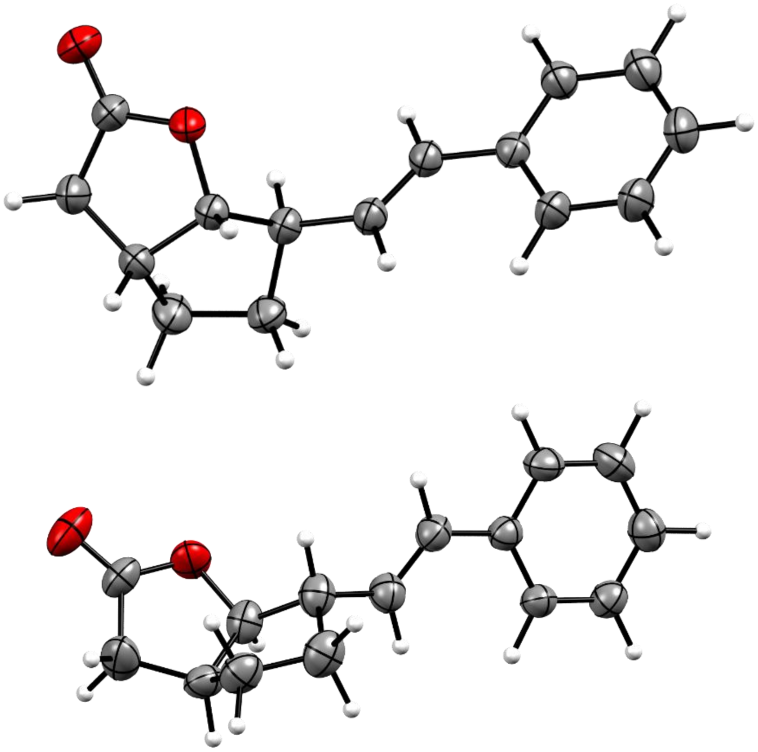

Figure S9. ORTEP representation of **5fa** (two enantiomers observed). All atoms except hydrogen were drawn at 30% probability.
Atom labelling: carbon= grey; oxygen= red; and hydrogen= white.

Crystal data Au2. CCDC number: 2534753. $C_{18}H_{26}AuClNO_3P$ (*M* = 567.78 g/mol): triclinic, space group P_{-1} (no. 2), *a* = 8.5639(13) Å, *b* = 10.8449(16) Å, *c* = 10.8724(15) Å, α = 84.816(4), *β* = 88.369(4), γ = 77.794(4), *V*= 982.9(2) Å^3, *Z* = 2, *T* = 200 K, μ(Mo Kα) = 7.718 mm^{-1}, *Dcalc* = 1.919 g/cm^3, 32445 reflections measured (3.762 ≤ 2Θ ≤ 61.558), 6092 unique (R_{int} = 0.0656, R_{sigma} = 0.0468) which were used in all calculations. The final R_1 was 0.0428 (I>=2u(I)) and wR_2 was 0.1111 (all data).

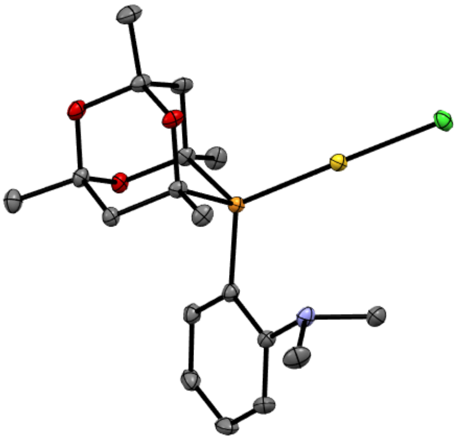

Figure S10. ORTEP representation of **Au2**. All atoms are drawn at 30% probability. Hydrogens are omitted for clarity.
Atom labelling: carbon= grey; oxygen= red; nitrogen= blue; phosphorus= orange; gold= yellow; chlorine= green.

8. References

[29]Herrera-Luna, J. C.; Hagui, W.; Tocqueville, D.; Anelli, E.; Espagne, A.; Ollivier, C.; Mansuy, V. M.; Fensterbank, L. Gold(I)-Catalyzed Csp^3-Csp Cross Coupling. Access to Alkynylated Tetrahydrofuran Derivatives. *Adv. Syn. Catal.* **2026**. DOI: 10.1002/adsc.70474

[37]Muratov, K.; Zaripov, E.; Berezovski, M. V.; Gagosz, F. DFT-Enabled Development of Hemilabile (P^N) Ligands for Gold(I/III) RedOx Catalysis: Application to the Thiotosylation of Aryl Iodides. *J. Am. Chem. Soc.* **2024**, *146*, 3660–3674.

[40]Jankins, T. C.; Martin-Montero, R.; Cooper, P.; Martin, R.; Engle, K. M. Low-Valent Tungsten Catalysis Enables Site-Selective Isomerization–Hydroboration of Unactivated Alkenes. *J. Am. Chem. Soc.* **2021**, *143*, 37, 14981–14986

[41]Zhu, X.; Zhang, M.; Shen, L.; Su, W. Visible-Light-Induced Hydrodifluoromethylation of Unactivated Alkenes with Difluoroacetic Anhydride. *J. Org. Chem.* **2024**, *89*, 12, 8828–8835

[42]Melder, J. J.; Heldner, M. L.; Kugler, R.; Ziegenhagen, L. A.; Rominger, F.; Rudolph, M.; Hashmi, A. S. K. Easy Access to Functionalized Indolines and Tetrahydroquinolines via a Photochemical Cascade Cyclization Reaction. *J. Am. Chem. Soc.* **2024**, *146*, 21, 14521–14527

[43]Miller, D. C.; Choi, G. J.; Orbe, H. S.; Knowles, R. R. Catalytic Olefin Hydroamidation Enabled by Proton-Coupled Electron Transfer. *J. Am. Chem. Soc.* **2015**, *137*, 42, 13492–13495

[44]Wang, J.-J.; Yu, W. Anti-Markovnikov Hydroazidation of Alkenes by Visible-Light Photoredox Catalysis. *Chem. Eur. J.* **2019**, *25*, 3510–3514

[45]Sissengaliyeva, G.; Dénès, F.; Girbu, V.; Kulcitki, V.; Hofstetter, E.; Renaud, P. Radical-Mediated Hydroperfluoroalkylation of Unactivated Alkenes. *Adv. Synth. Catal.* **2023**, *365*, 2568–2676

[46]Yang, G.; Zhang, W. Regioselective Pd-Catalyzed Aerobic Aza-Wacker Cyclization for Preparation of Isoindolinones and Isoquinolin-1(2H)-ones. *Org. Lett.* **2012**, *14*, 1, 268–271

[47]Chen, Z.; Wu, L.; Fang, H.; Zhang, T.; Mao, Z.; Zou, Y.; Zhang, X.; Yan, M. Intramolecular Hydroamidation of ortho-Vinyl Benzamides Promoted by Potassium tert-Butoxide/N,N-Dimethylformamide. *Adv. Synth. Catal.* **2017**, *359*, 3894–3899

[48]Knapp, S.; Levorse, A. T. Synthesis and reactions of iodo lactams. *J. Org. Chem.* **1988**, *53*, 17, 4006–4014

[49]van Vliet, K. M.; Polak, L. H.; Siegler, M. A.; van der Vlugt, J. I.; Guerra, C. F.; de Bruin, B. Efficient Copper-Catalyzed Multicomponent Synthesis of N-Acyl Amidines via Acyl Nitrenes. *J. Am. Chem. Soc.* **2019**, *141*, 38, 15240–15249

[50]Curran, D. P.; Liu, H. Radical translocation reactions across amides. 1,5-Hydrogen-transfer reactions of o-iodobenzamides and *N*-(o-iodobenzyl) amides. *J. Chem. Soc., Perkin Trans. 1*, **1994**, 1377-1393

[51]Elbert, B. L.; Lim, D. S. W.; Gudmundsson, H. G.; O'Hanlon, J. A.; Anderson, E. A. Synthesis of Cyclic Alkenylsiloxanes by Semihydrogenation: A Stereospecific Route to (*Z*)-Alkenyl Polyenes. *Chem. Eur. J.*, **2014**, *20*, 8594-8598.

[52]*Syntheses Using Alkyne-Derived Alkenyl- and Alkynylaluminum Compounds. In Organic Reactions, (Ed.).* **2004**

[53]Xia, Z.; Corcé, V.; Zhao, F.; Przybylski, C.; Espagne, A.; Jullien, L.; Le Saux, T.; Gimbert, Y.; Dossmann, H.; Mouriès-Mansuy, V.; Ollivier, C.; Fensterbank, L. Photosensitized oxidative addition to gold(i) enables alkynylative cyclization of o-alkylnylphenols with iodoalkynes. *Nat. Chem.* **2019**, *11*, 797

[54]Dumele, D. Wu, N. Trapp, N. Goroff, F. Diederich, "Halogen Bonding of (Iodoethynyl)benzene Derivatives in Solution" *Org. Lett.* **2014**, *16*, 18, 4722-4725

[55]Zhao, F.; Abdellaoui, M.; Hagui, W.; Ballarin-Marion, M.; Berthet, J.; Corcé, V.; Delbaere, S.; Dossmann, H.; Espagne, A.; Forté, J.; Jullien, L.; Le Saux, T.; Mouriès-Mansuy, V.; Ollivier, C.; Fensterbank, L. Reactant-induced photoactivation of in situ generated organogold intermediates leading to alkynylated indoles via Csp^2-Csp cross-coupling. *Nat Commun.* **2022**, *13*, 2295

[56]Rao, D. S.; Reddya, T. R.; Kashyap, S. Chemoselective and stereospecific iodination of alkynes using sulfonium iodate(i) salt. *Org. Biomol. Chem.,* **2018**, *16*, 1508- 1518

[57]Nguyen, S. T.; Ellington, T. L.; Allen, K. E.; Gorden, J. D.; Rheingold, A. L.; Tschumper, G. S.; Hammer, N. I.; Watkins, D. L. Systematic Experimental and Computational Studies of Substitution and Hybridization Effects in Solid-State Halogen Bonded Assemblies. *Cryst. Growth Des.* **2018**, *18*, 5, 3244-3254

[58]Govdi, A. I.; Danilkina, N. A.; Ponomarev, A. V.; Balova, I. A. 1-Iodobuta-1,3-diynes in Copper-Catalyzed Azide–Alkyne Cycloaddition: A One-Step Route to 4-Ethynyl-5-iodo-1,2,3-triazoles. *J. Org. Chem.* **2019**, *84*, 4, 1925–1940

[59]Yamagishi, M.; Nishigai, K.; Hata, T.; Urabe, H. Nucleophilic Addition of Sulfonamides to Bromoacetylenes: Facile Preparation of Pyrroles. *Org. Lett.* **2011**, *13*, 18, 4873-4875

[60]He, Y.; Zhong, Y.; Ballarin-Marion, M.; Herrera-Luna, J. C.; Ma, W.; Hu, Y.; Ollivier, C.; Mouriès-Mansuy, V.; Fensterbank, L.; Zhao, F.; Xia, Z.; Fan, B. Visible light-mediated gold-catalyzed alkynylative cyclization of allenoates with iodoalkynes for the synthesis of β-alkynyl-γ-butenolides. *Org. Chem. Front.*, **2024**, *11*, 5695-5702.

[61]Zhang, S.; Wang, C.; Ye, X.; Shi, X. Intermolecular Alkene Difunctionalization via Gold-Catalyzed Oxyarylation. *Angew. Chem. Int. Ed.* **2020**, *59*, 20470-20474

[62]Hahn, C.; Cruz, L.; Villalobos, A.; Garza, L.; Adeosun, S. Synthesis, structure and catalytic activity of a gold(i) complex containing 1,2-bis(diphenylphosphino)benzene monoxide. *Dalton Trans.*, **2014**, *43*, 16300-16309

[63]Brenstrum, T.; Gerristma, D. A.; Adjabeng, G. M.; Frampton, C. S.; Britten, J.; Robertson, A. J.; McNulty, J.; Capretta, A. Phosphaadamantanes as Ligands for Palladium Catalyzed Cross-Coupling Chemistry: Library Synthesis, Characterization, and Screening in the Suzuki Coupling of Alkyl Halides and Tosylates Containing β-Hydrogens with Boronic Acids and Alkylboranes. *J. Org. Chem.* **2004**, *69*, 7635–7639

[64] Wang, D. M.; He, Y-Q.; Wu, Y.; Tang, Y.; Wang, P. Construction of Lactones via Ligand-Enabled Ni-Catalyzed Alkene Hydroxylarylation/Lactonization. *Org. Lett.* **2024**, *26*, 38, 8171–8176

[65]Bruker. *APEX / SAINT*. Bruker AXS Inc., Madison, Wisconsin, USA, **2012** https://www.bruker.com/en/products-and-solutions/diffractometers-and-x-ray-microscopes/single-crystal-x-ray-diffractometers/sc-xrd-software/apex.html (accessed 2023-06-26).

[66]Sheldrick, G. M. *SHELXT* – Integrated Space-Group and Crystal-Structure Determination. *Acta Crystallogr A Found Adv* **2015**, *71*, 3–8.

[67]Sheldrick, G. M. Crystal Structure Refinement with *SHELXL*. *Acta Crystallogr C Struct Chem* **2015**, *71*, 3–8.

[68]Dolomanov, O. V.; Bourhis, L. J.; Gildea, R. J.; Howard, J. A. K.; Puschmann, H. *OLEX2* : A Complete Structure Solution, Refinement and Analysis Program. *J Appl Crystallogr* **2009**, *42*, 339–341.

[69]Bourhis, L. J.; Dolomanov, O. V.; Gildea, R. J.; Howard, J. A. K.; Puschmann, H. The anatomy of a comprehensive constrained, restrained refinement program for the modern computing environment – Olex2 dissected. *Acta Crystallogr A Found Adv* **2015**, *71*, 59-75.

[70]Neese, F. The ORCA program system. *Wiley Interdiscip. Rev.: Comput. Mol. Sci*., **2012**, *2*, 1, 73–78.

[71]Kleemiss, F.; Dolomanov, O. V.; Bodensteiner, M.; Peyerimhoff, N.; Midgley, M.; Bourhis, L. J.; Genoni, A.; Malaspina, L. A.; Jayatilaka, D.; Spencer, J. L.; White, F.; Grundkoetter-Stock, B.; Steinhauer, S.; Lentz, D.; Puschmann, H.; Grabowsky, S. Accurate crystal structures and chemical properties from NoSpherA2. *Chem. Sci.*, **2021**, *12*, 1675-1692.

9. NMR Spectra

^{1}H NMR 300MHz, $CDCl_3$

1a

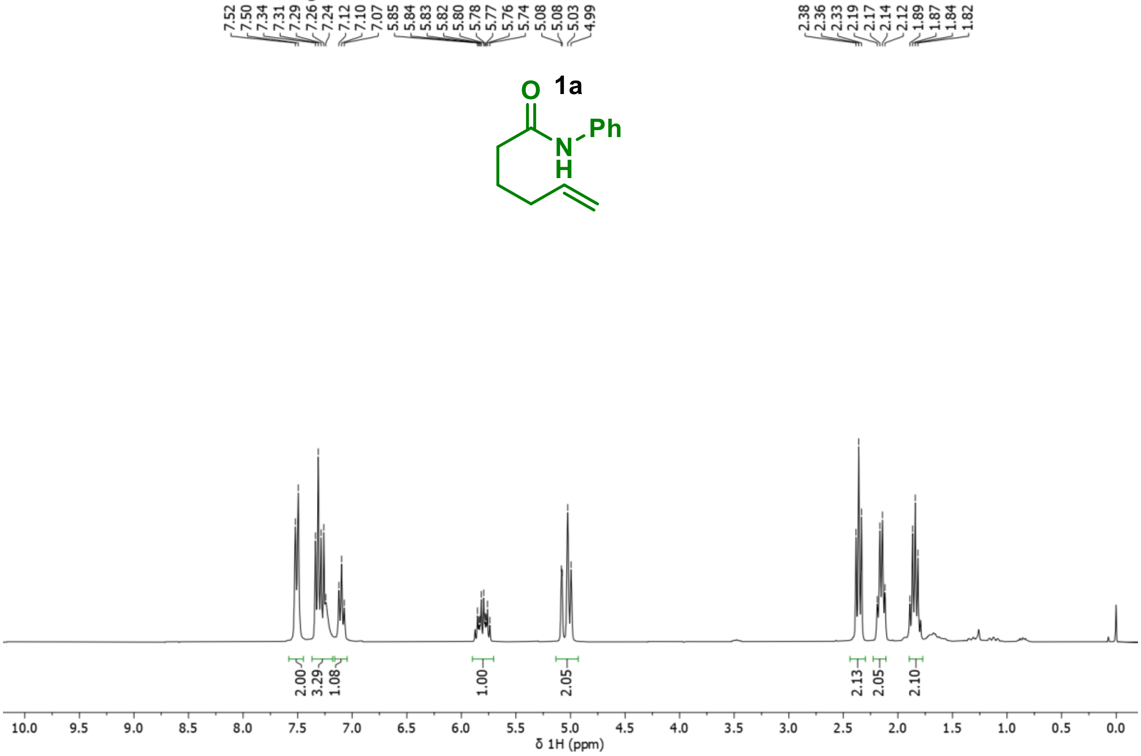

^{1}H NMR 300MHz, $CDCl_3$

1b

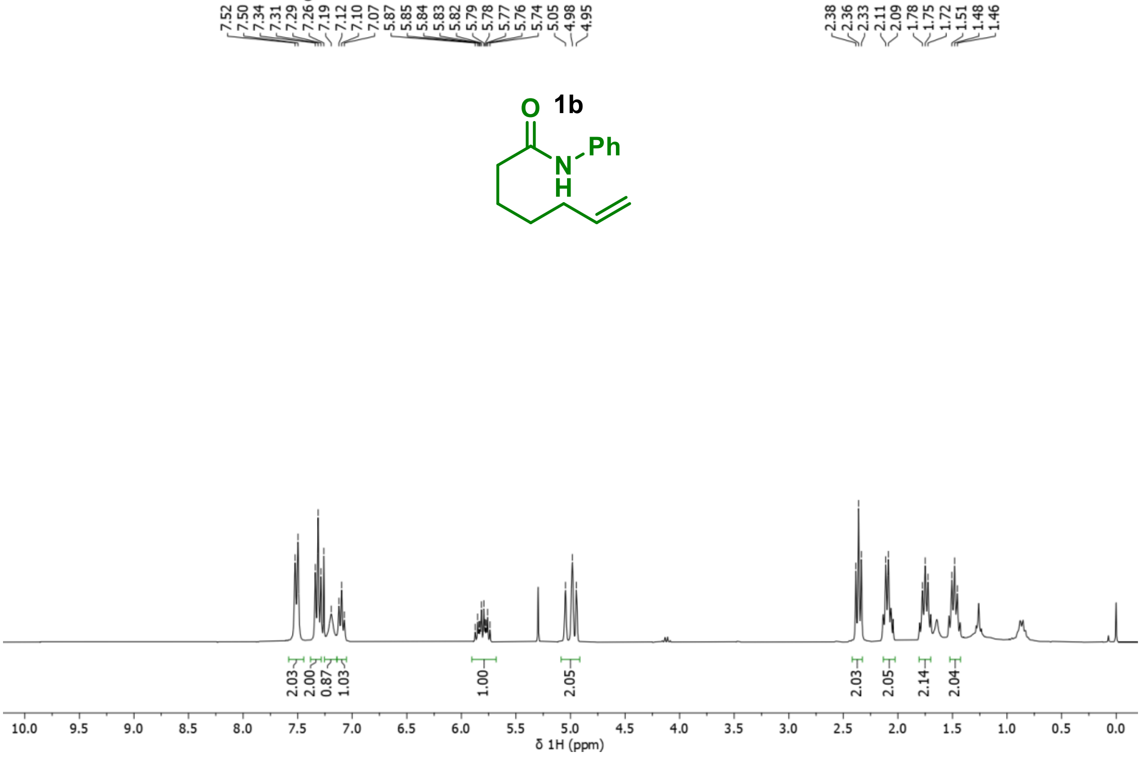

^{1}H NMR 300MHz, $CDCl_3$

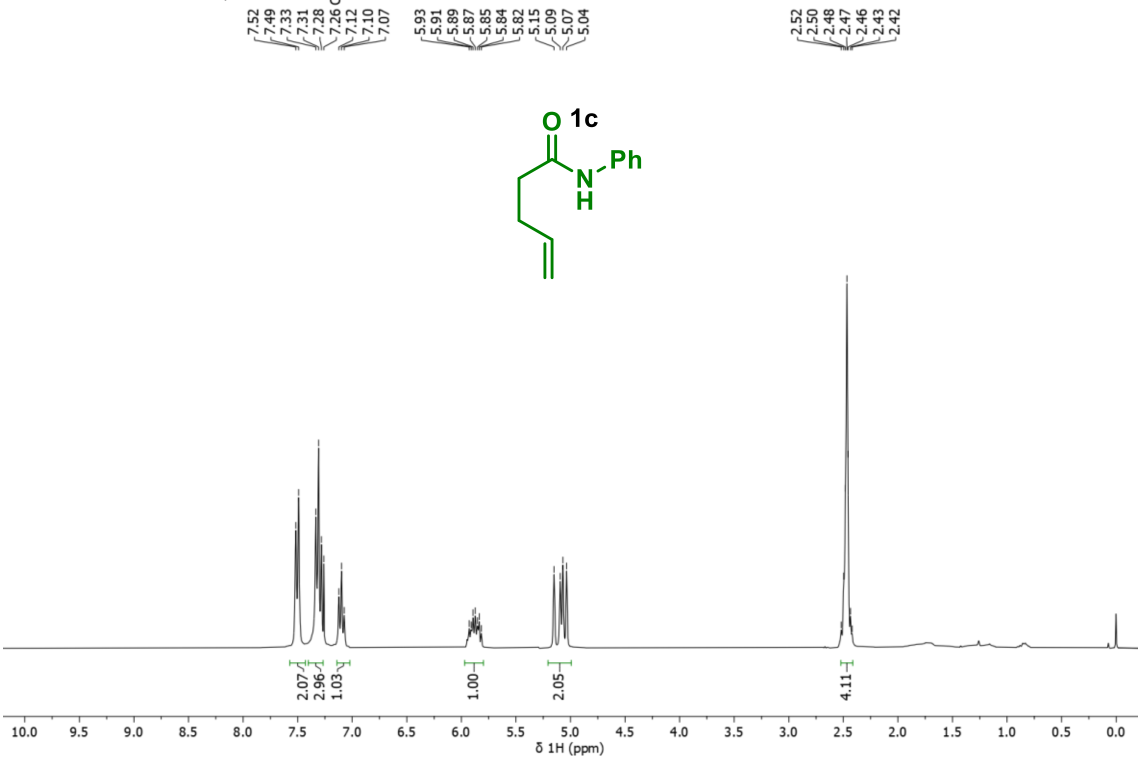

^{1}H NMR 300MHz, $CDCl_3$

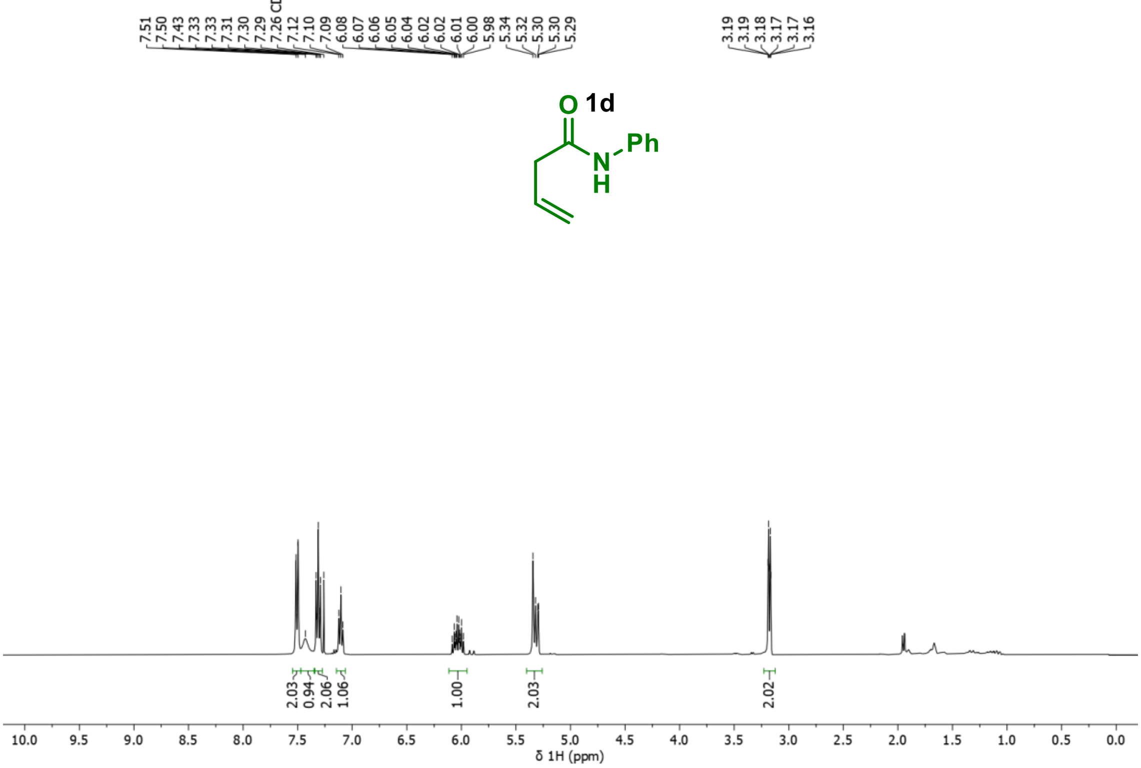

^{1}H NMR 300MHz, $CDCl_3$

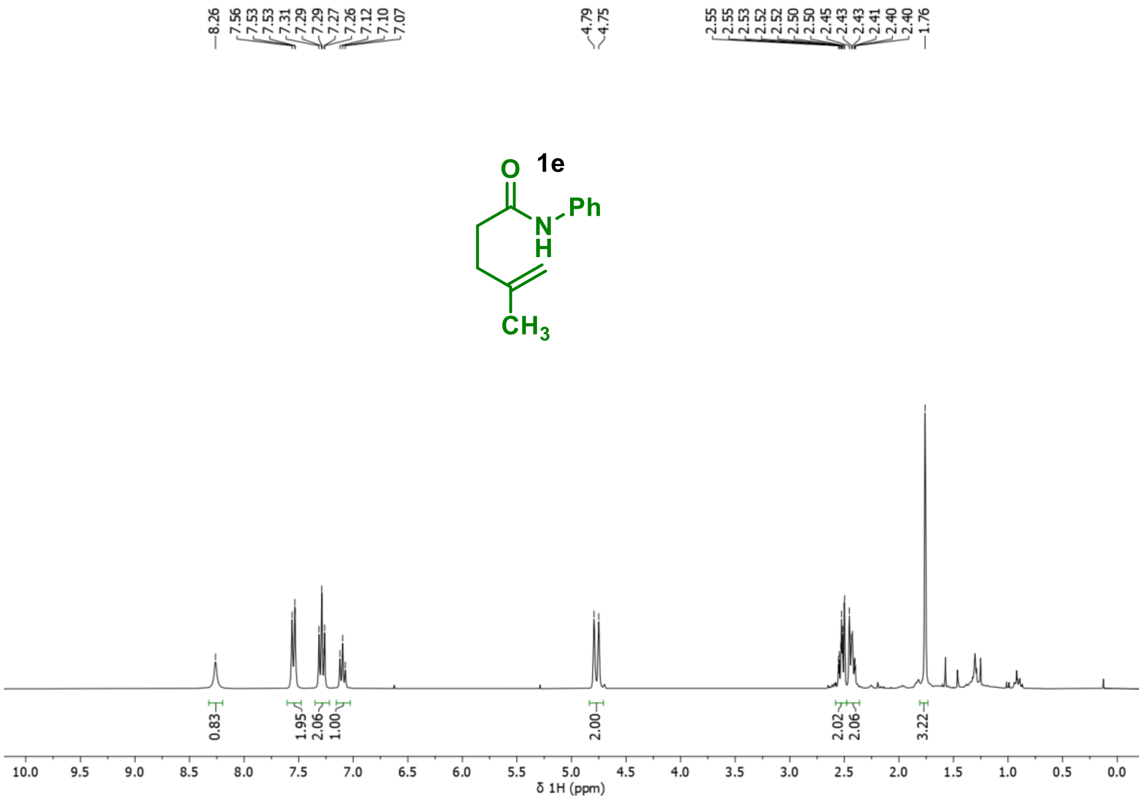

^{1}H NMR 300MHz, $CDCl_3$

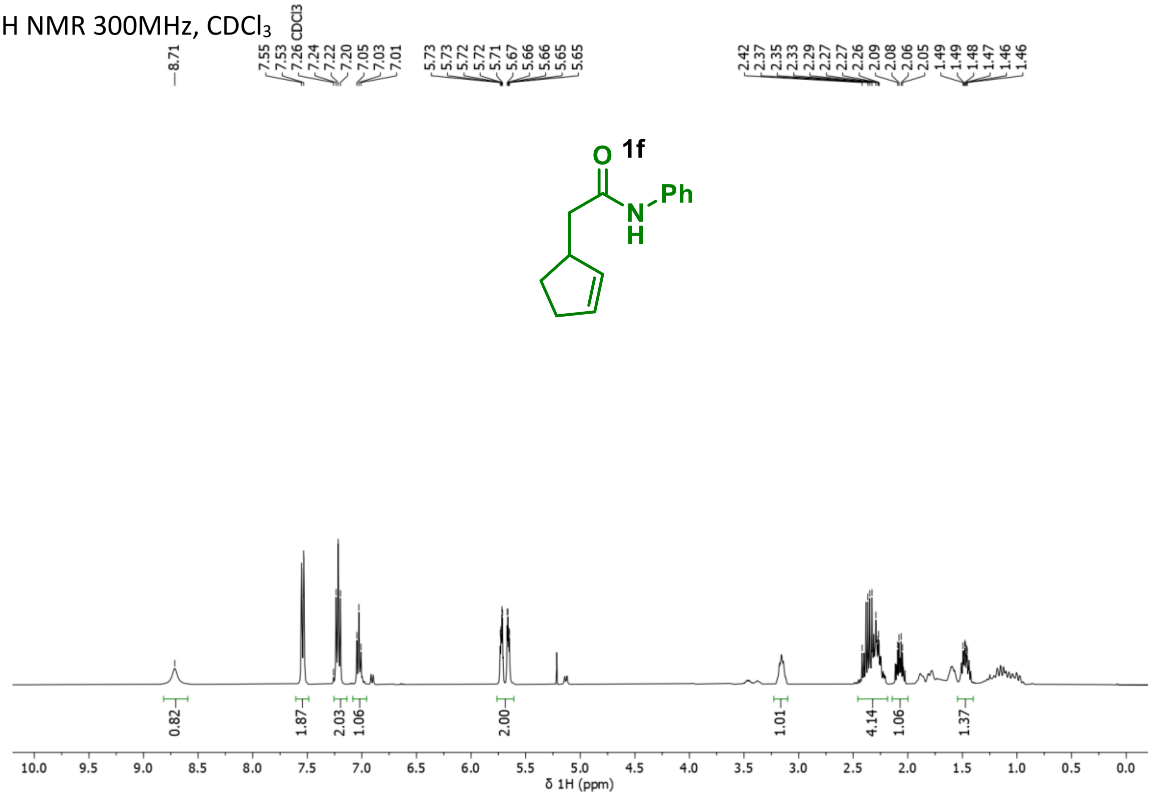

^{1}H NMR 300MHz, $CDCl_3$

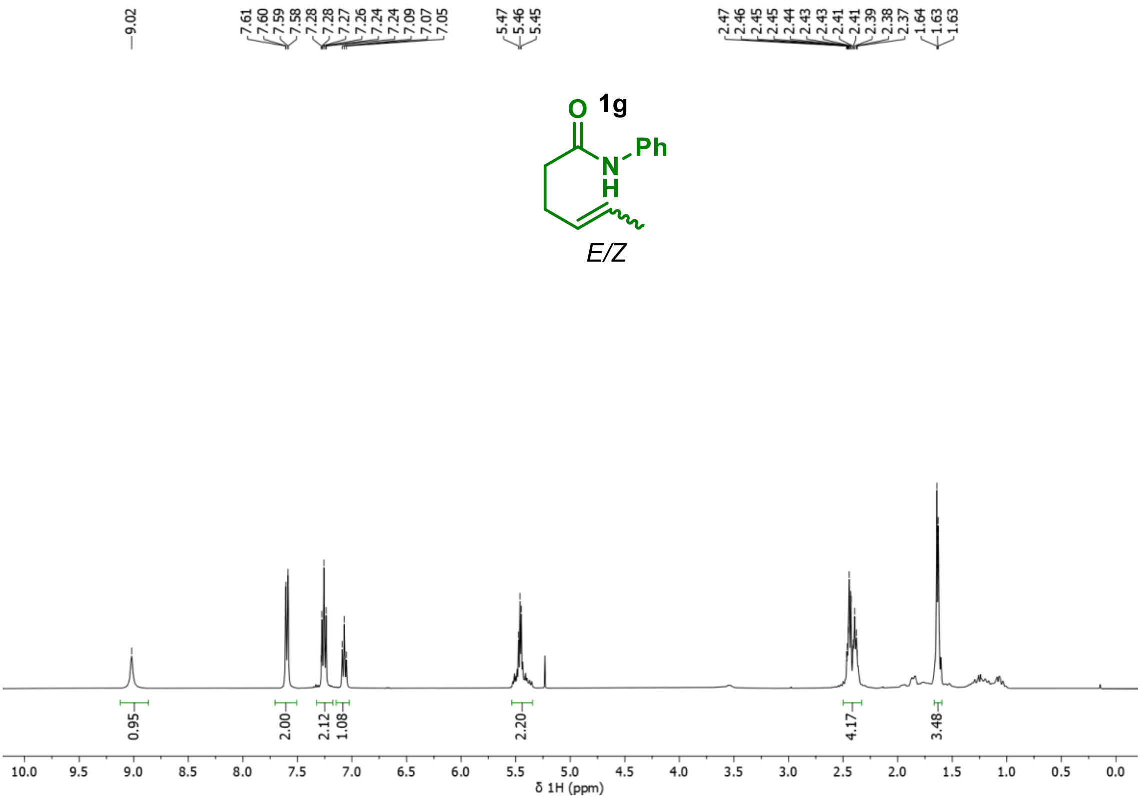

^{1}H NMR 300MHz, $CDCl_3$

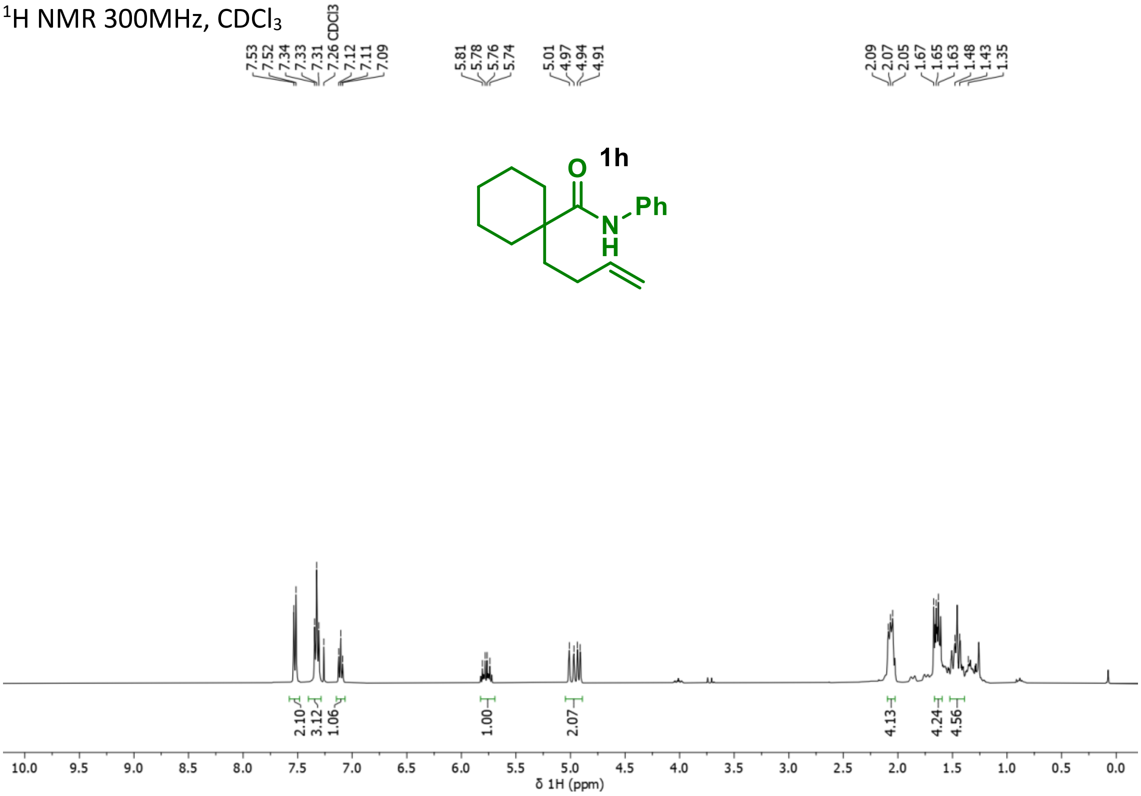

^{13}C NMR 101MHz, $CDCl_3$

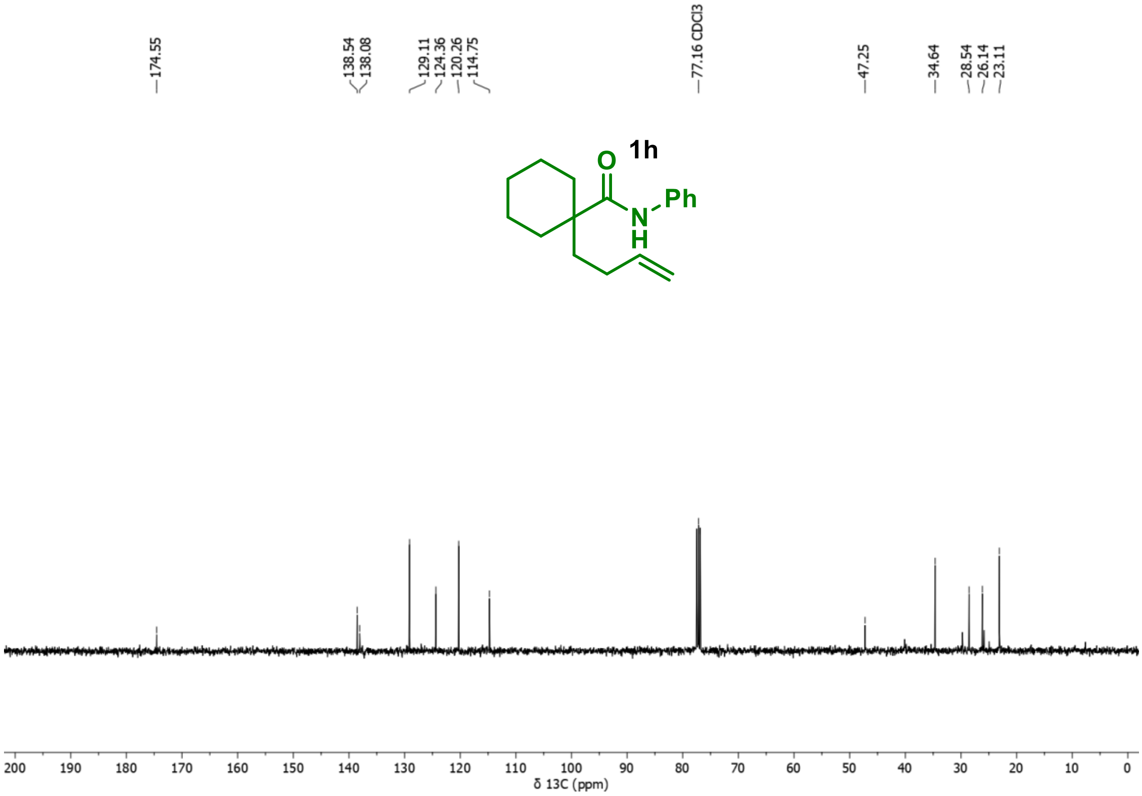

^{1}H NMR 300MHz, $CDCl_3$

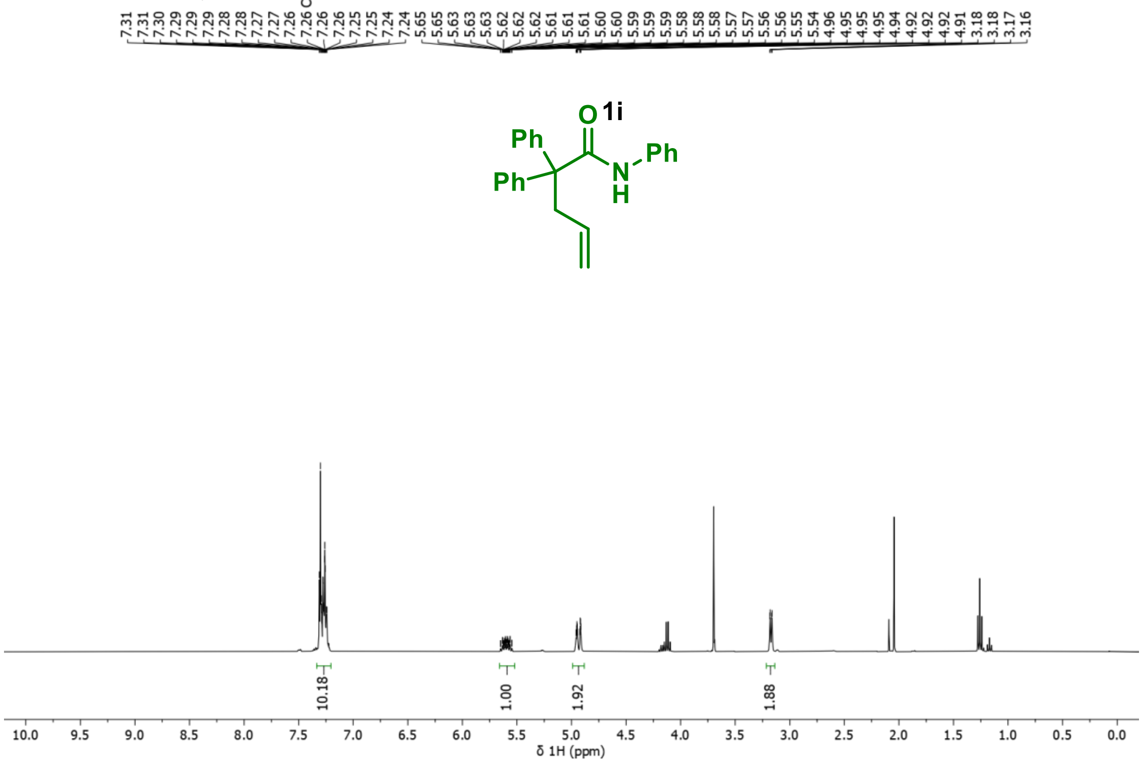

Table of contents

^{1}H NMR 300MHz, $CDCl_3$

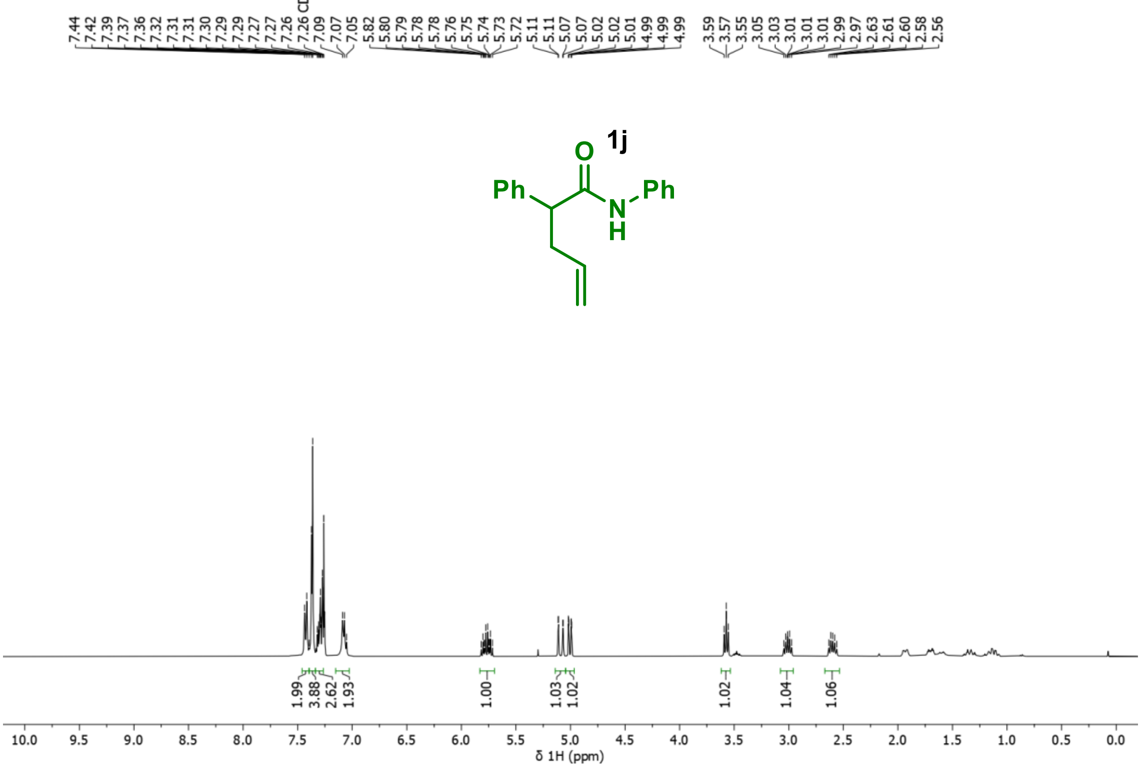

^{1}H NMR 300MHz, $CDCl_3$

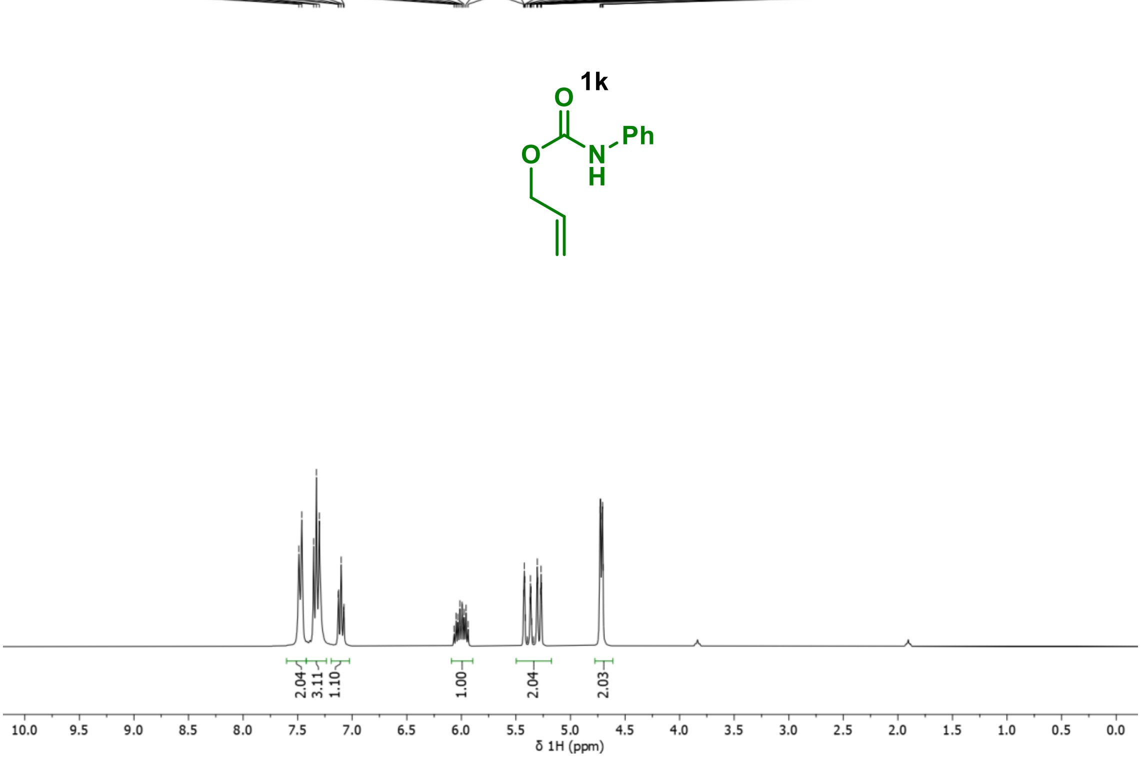

Table of contents

^{1}H NMR 300MHz, CD_3CN

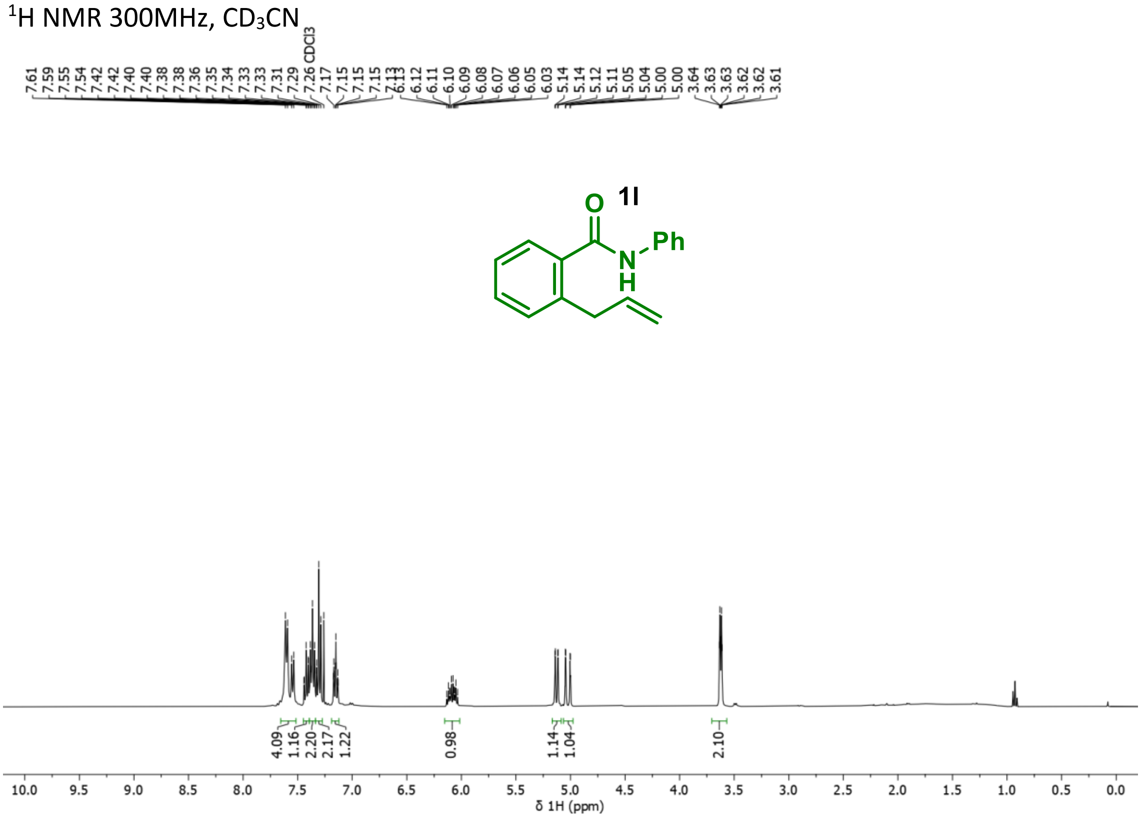

^{1}H NMR 300MHz, $CDCl_3$

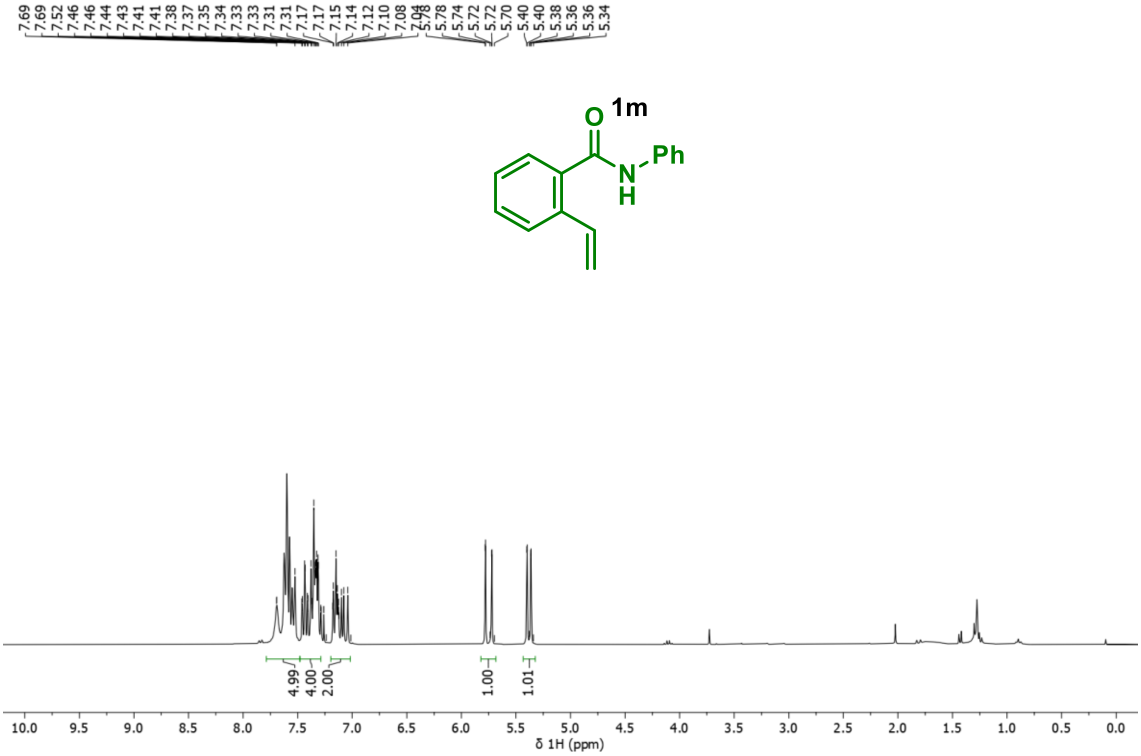

Table of contents

^{1}H NMR 300MHz, $CDCl_3$

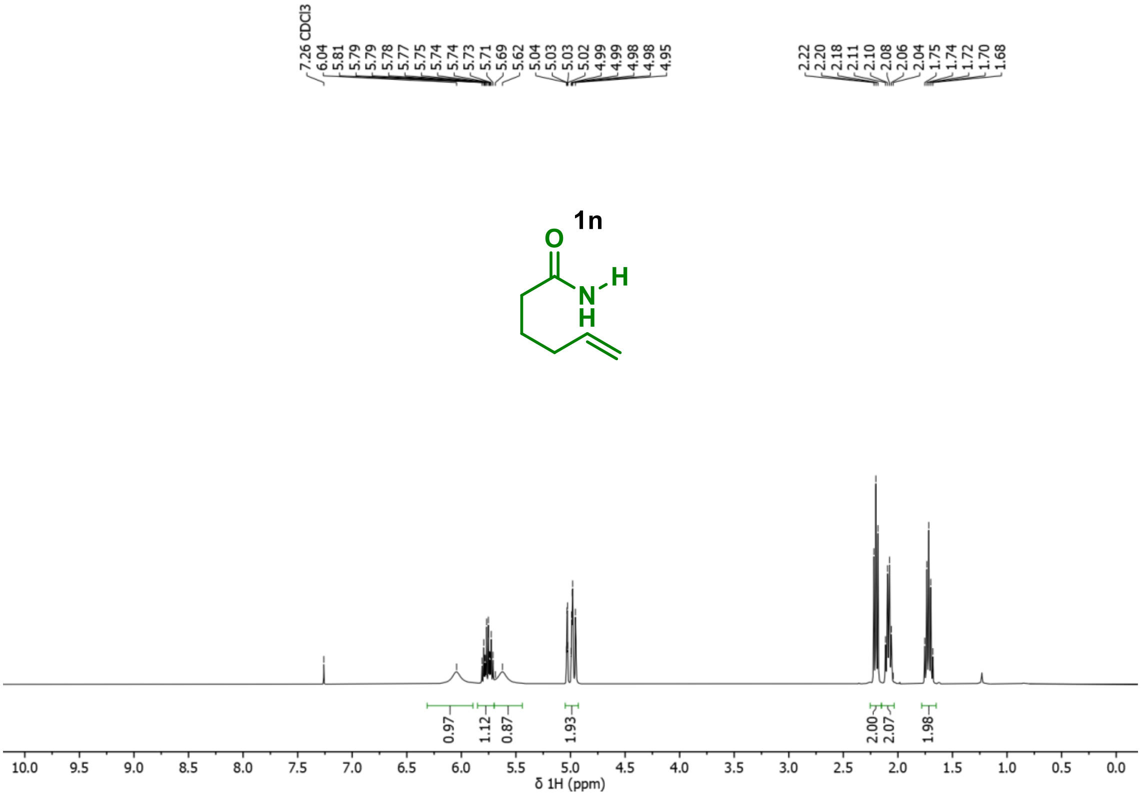

^{1}H NMR 300MHz, $CDCl_3$

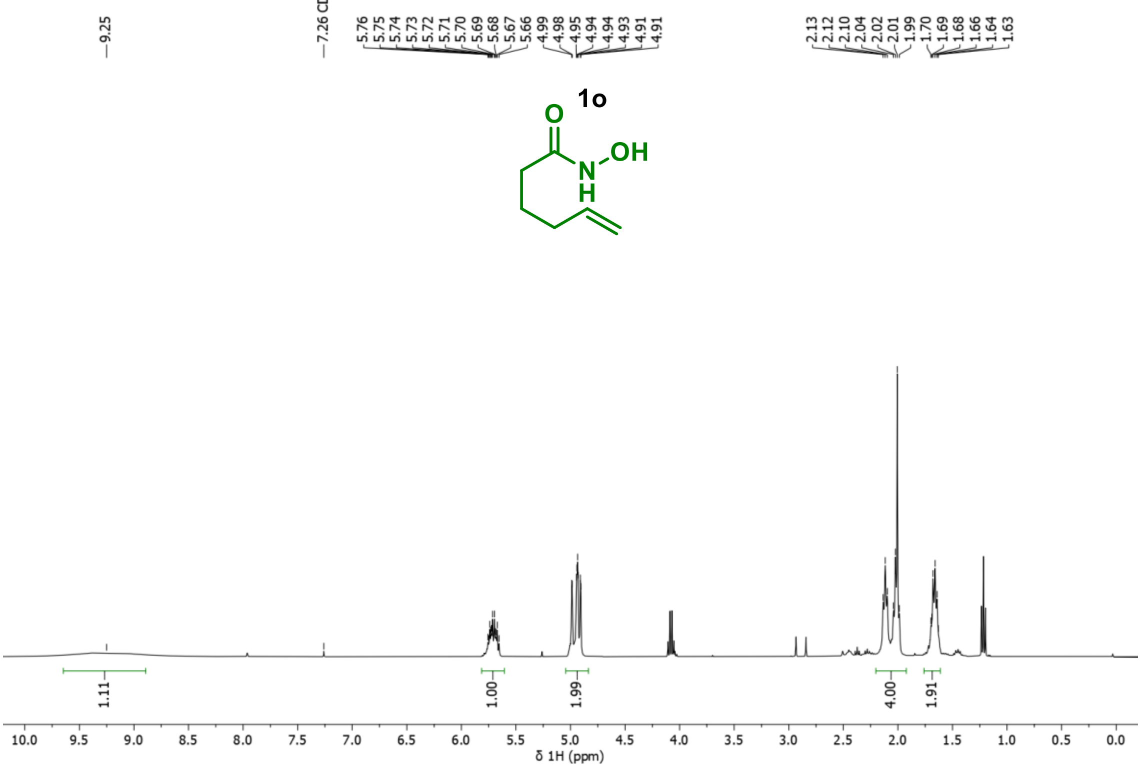

^{1}H NMR 300MHz, $CDCl_3$

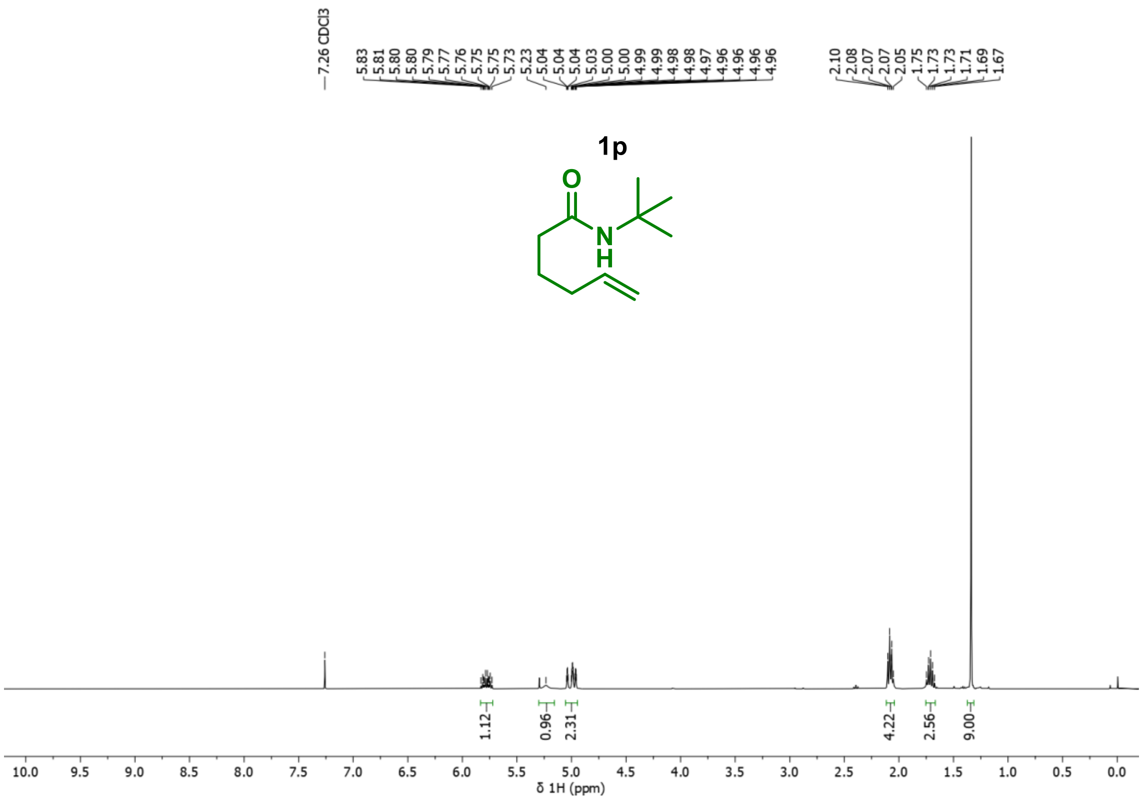

^{1}H NMR 300MHz, $CDCl_3$

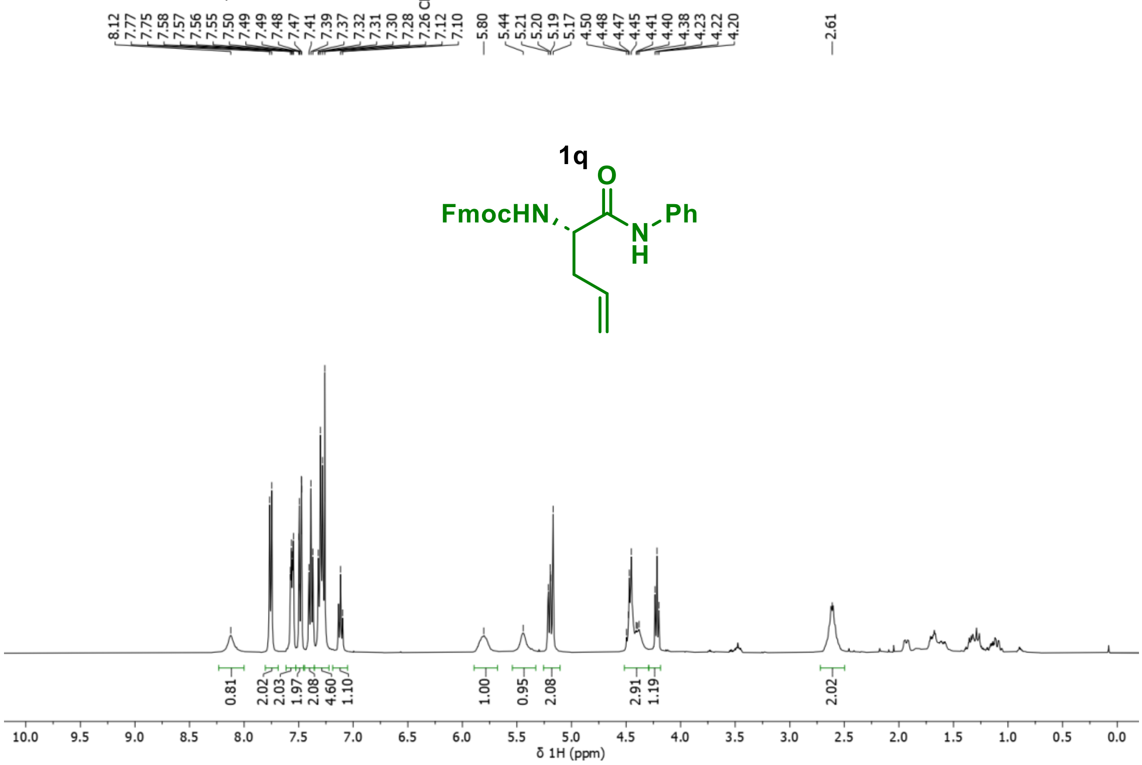

^{13}C NMR 101MHz, $CDCl_3$

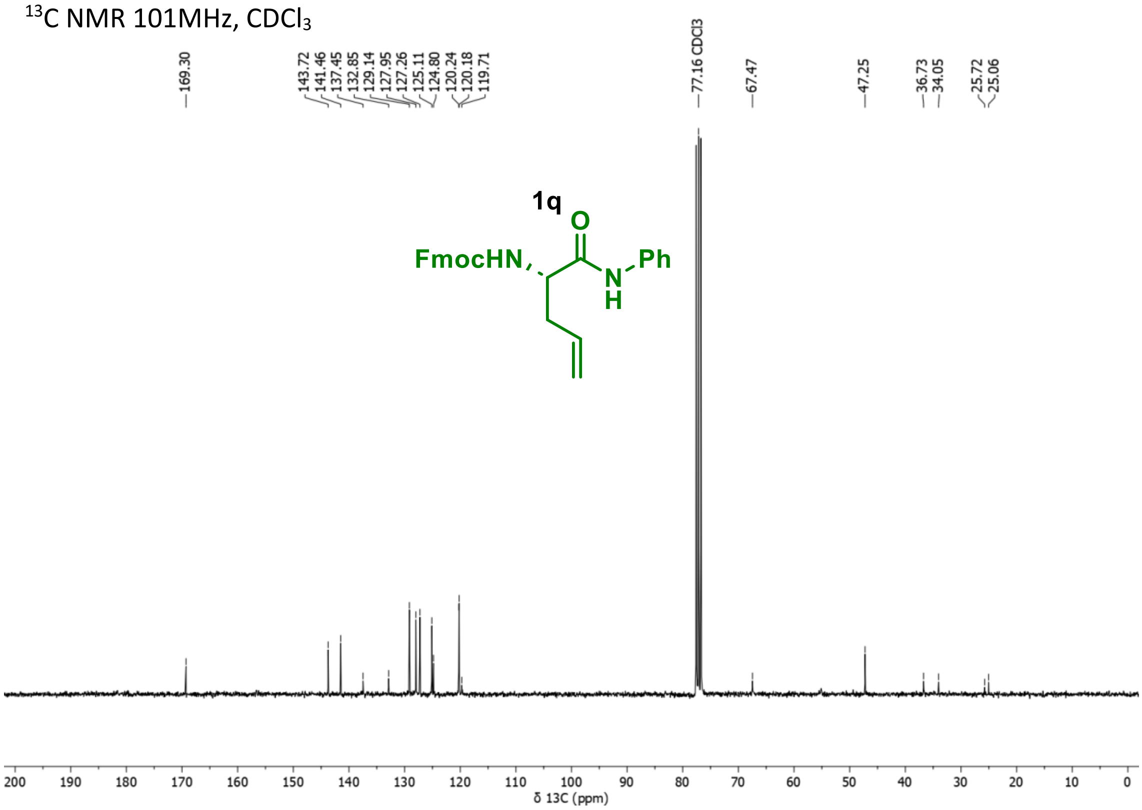

^{1}H NMR 300MHz, $CDCl_3$

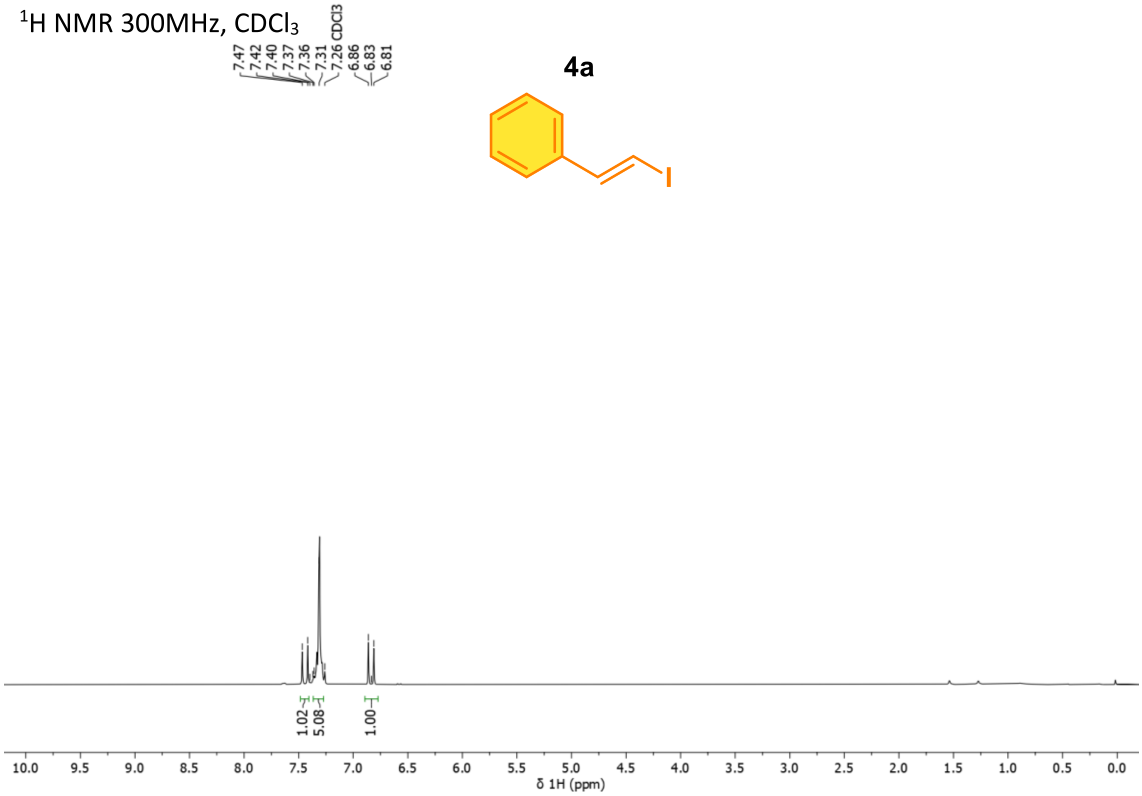

^1H NMR 300MHz, $CDCl_3$

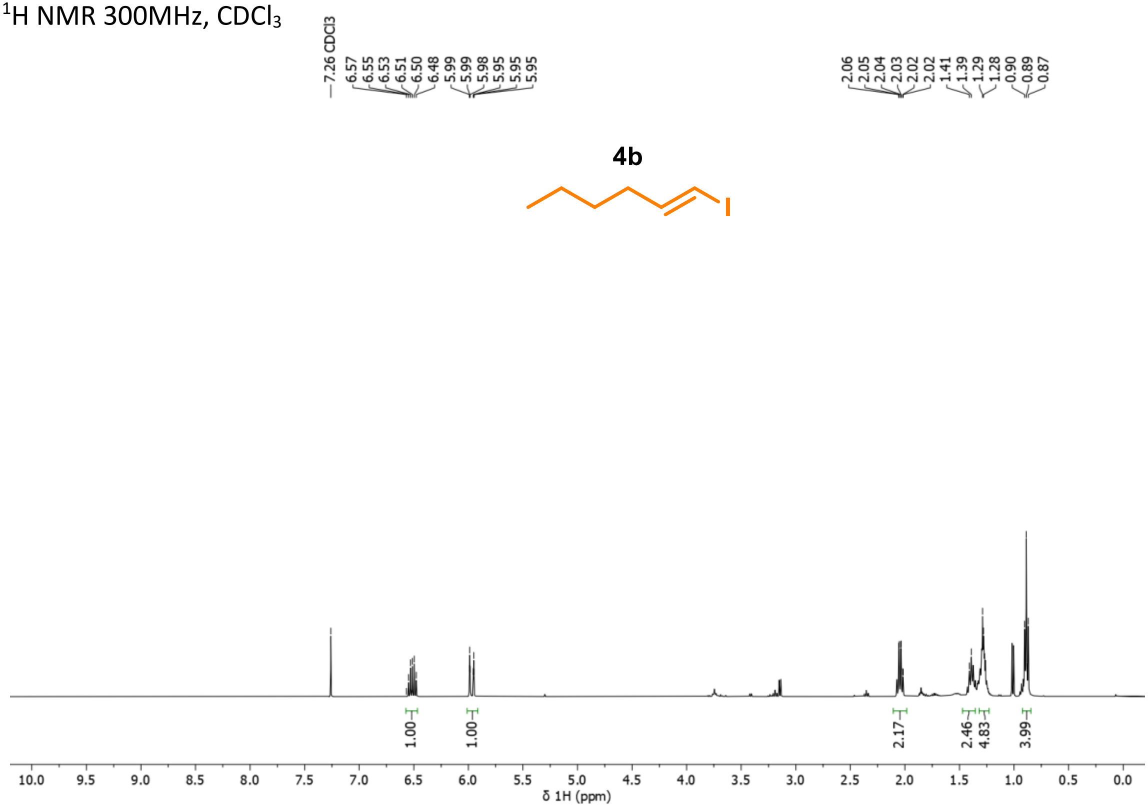

^1H NMR 300MHz, $CDCl_3$

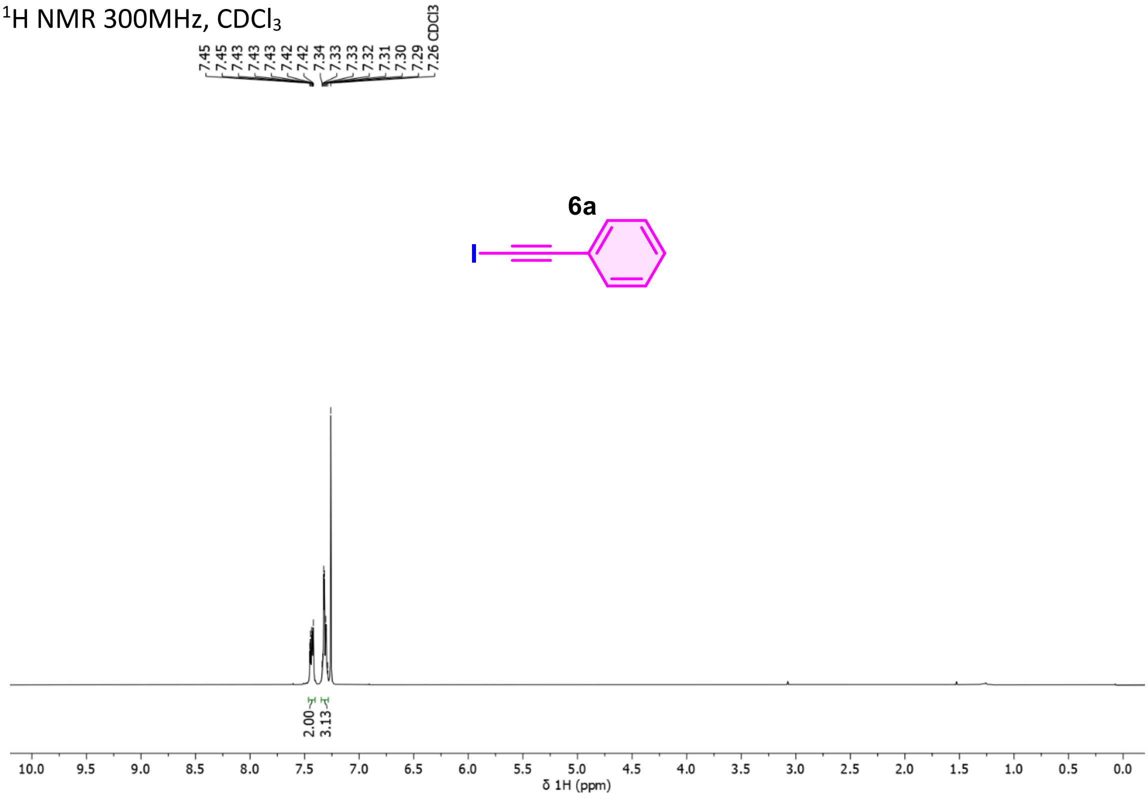

^{1}H NMR 300MHz, $CDCl_3$

6b

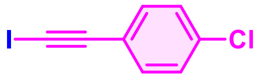

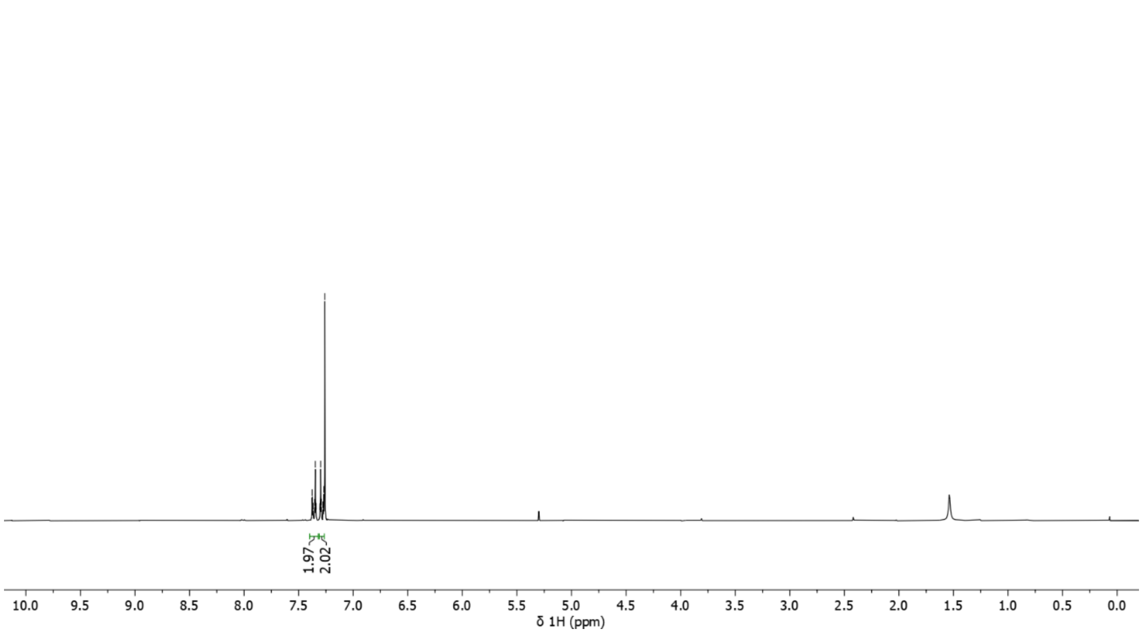

^{1}H NMR 300MHz, $CDCl_3$

6c

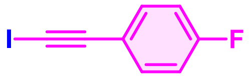

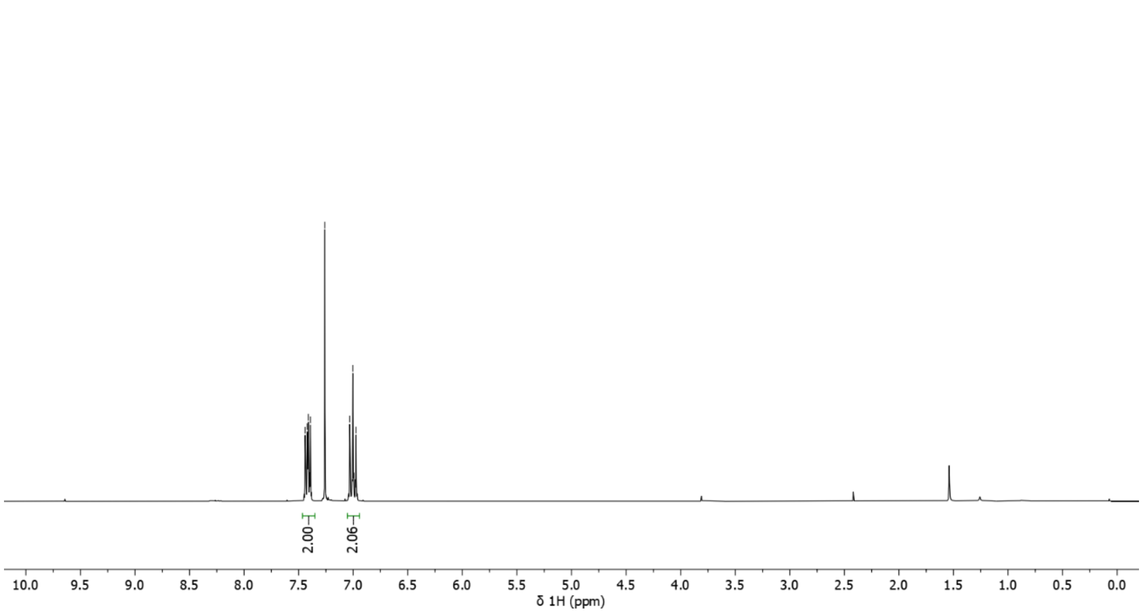

^{19}F NMR 282MHz, $CDCl_3$

6c

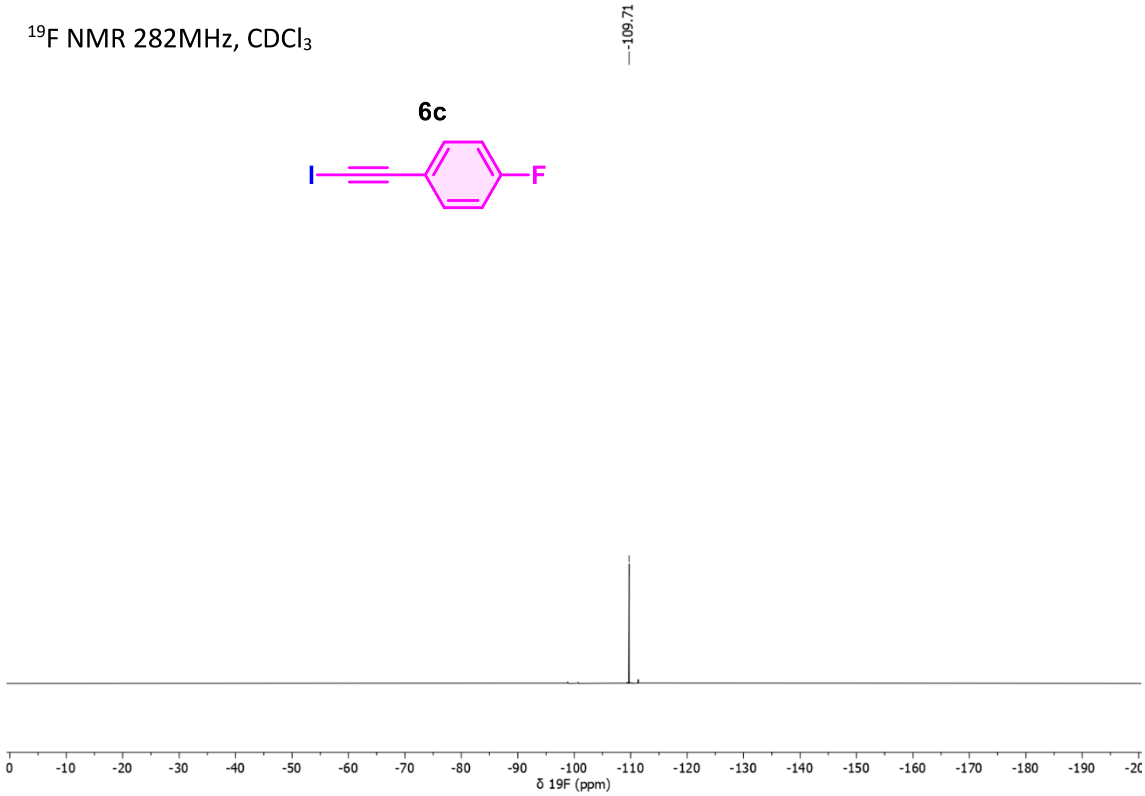

^{1}H NMR 300MHz, $CDCl_3$

6d

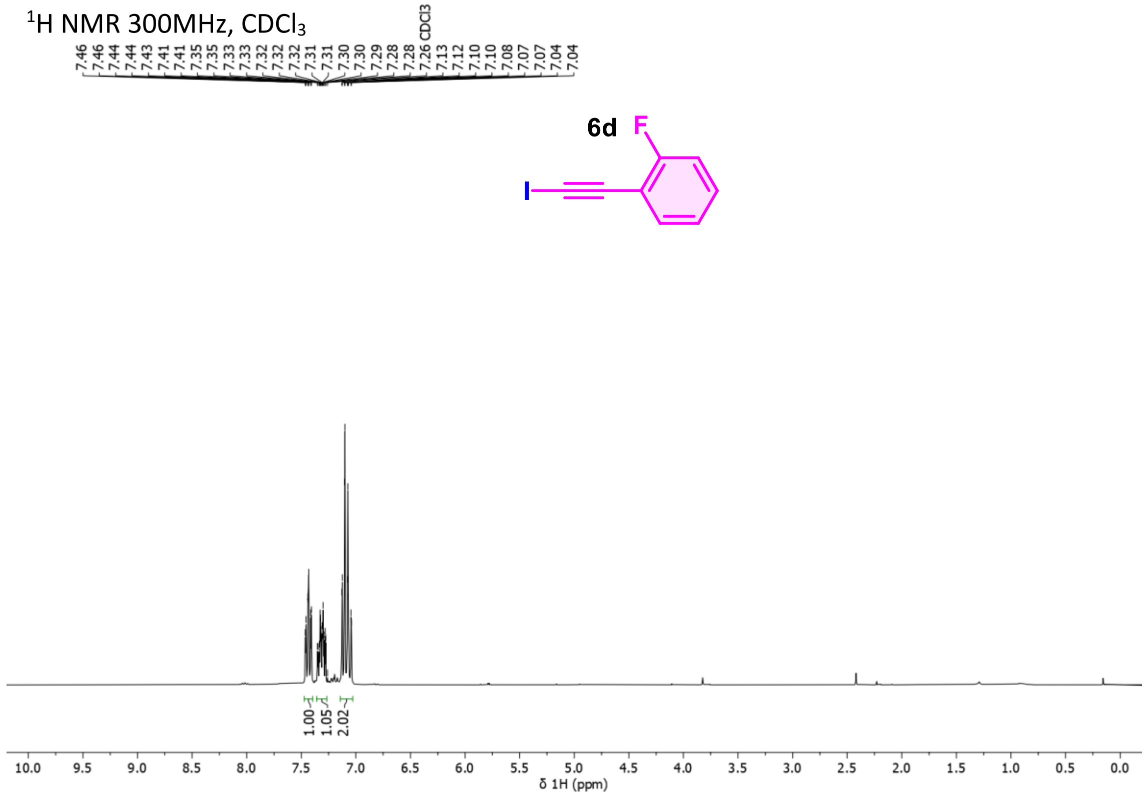

^{19}F NMR 282MHz, $CDCl_3$

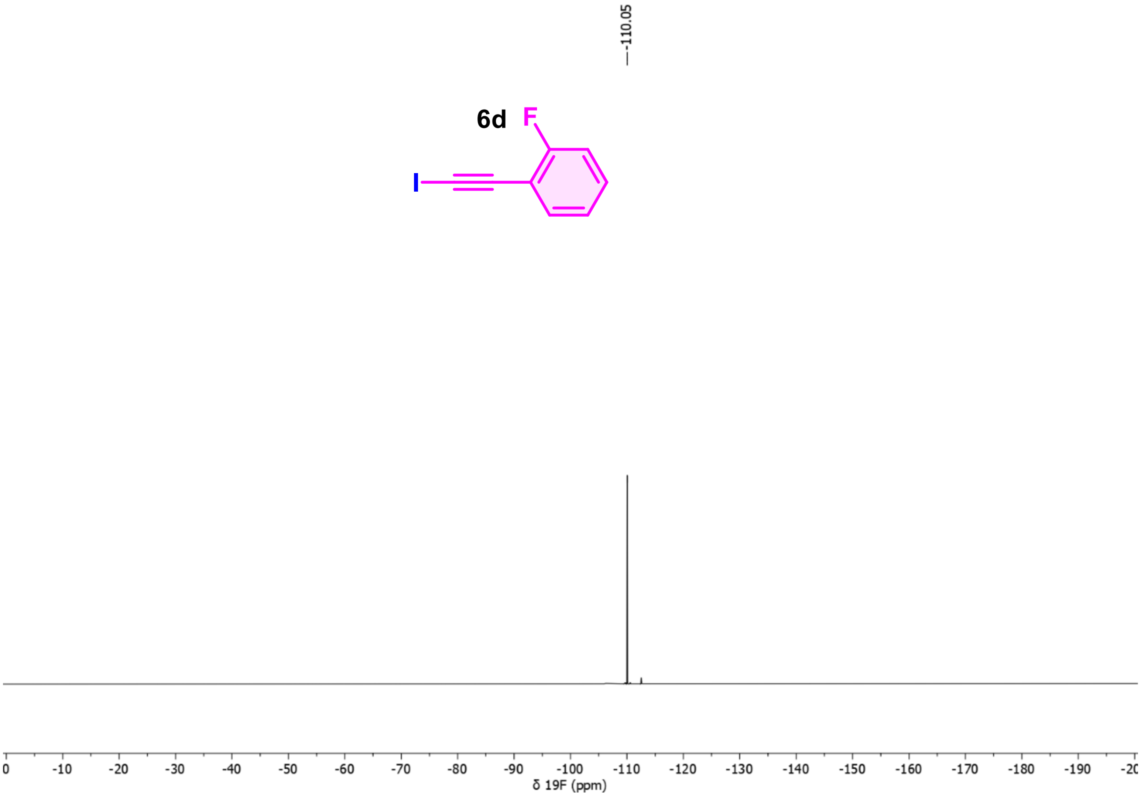

^{1}H NMR 300MHz, $CDCl_3$

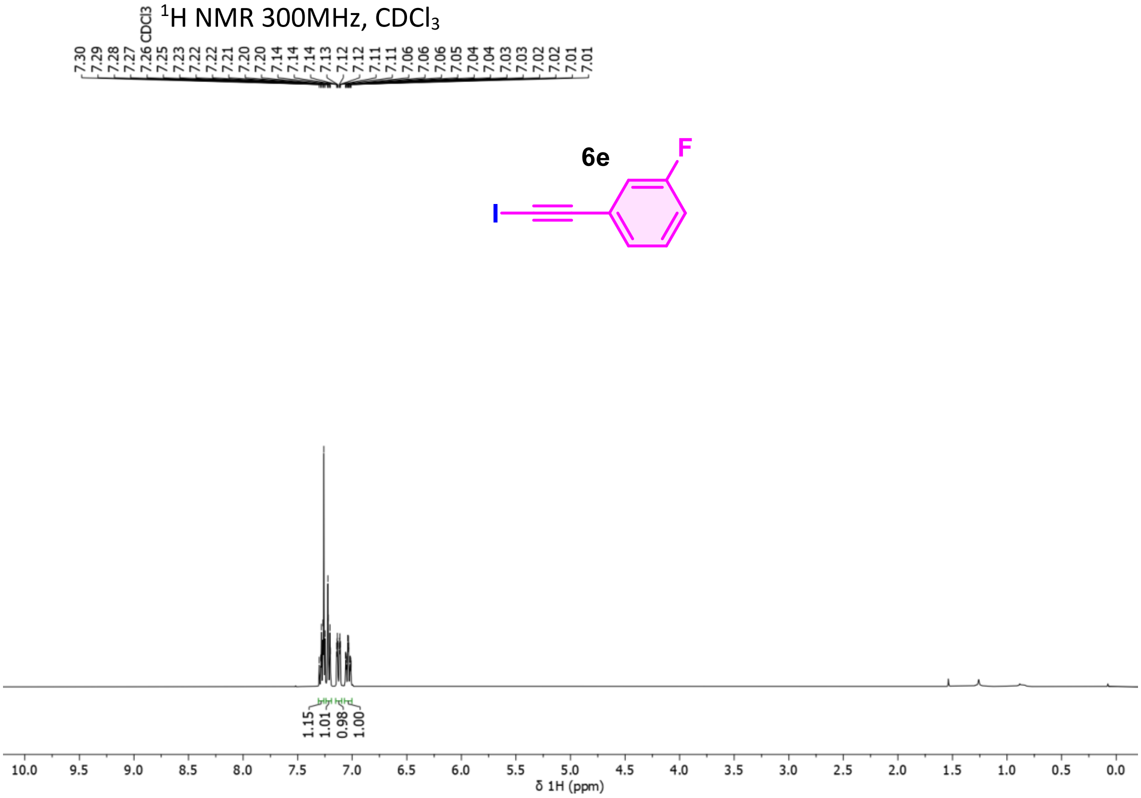

^{19}F NMR 282MHz, $CDCl_3$

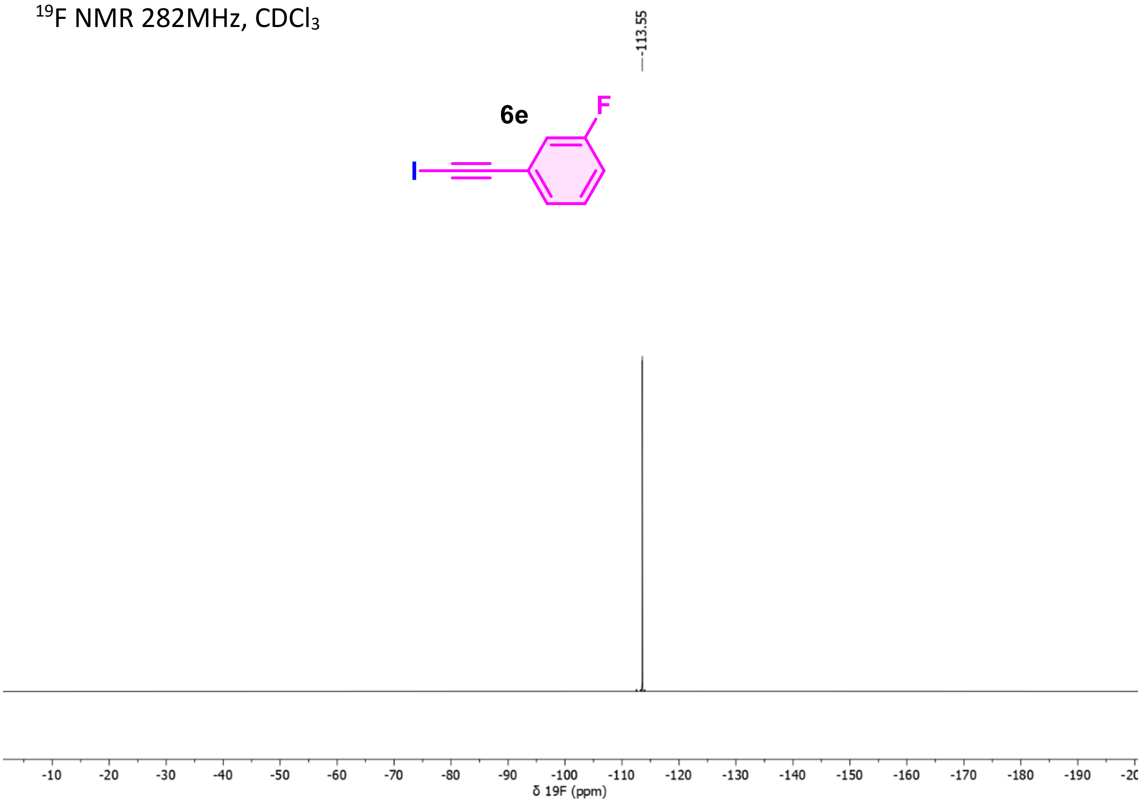

^{1}H NMR 300MHz, $CDCl_3$

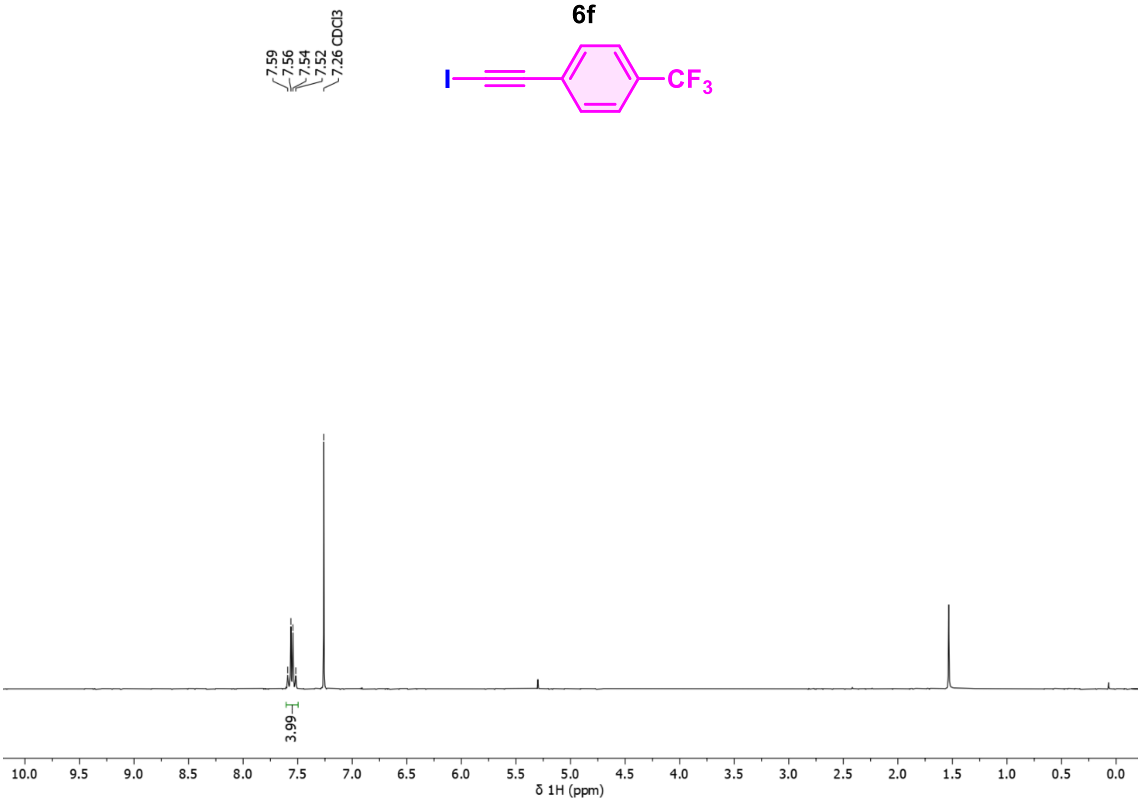

[Table of contents]

^{19}F NMR 282MHz, $CDCl_3$

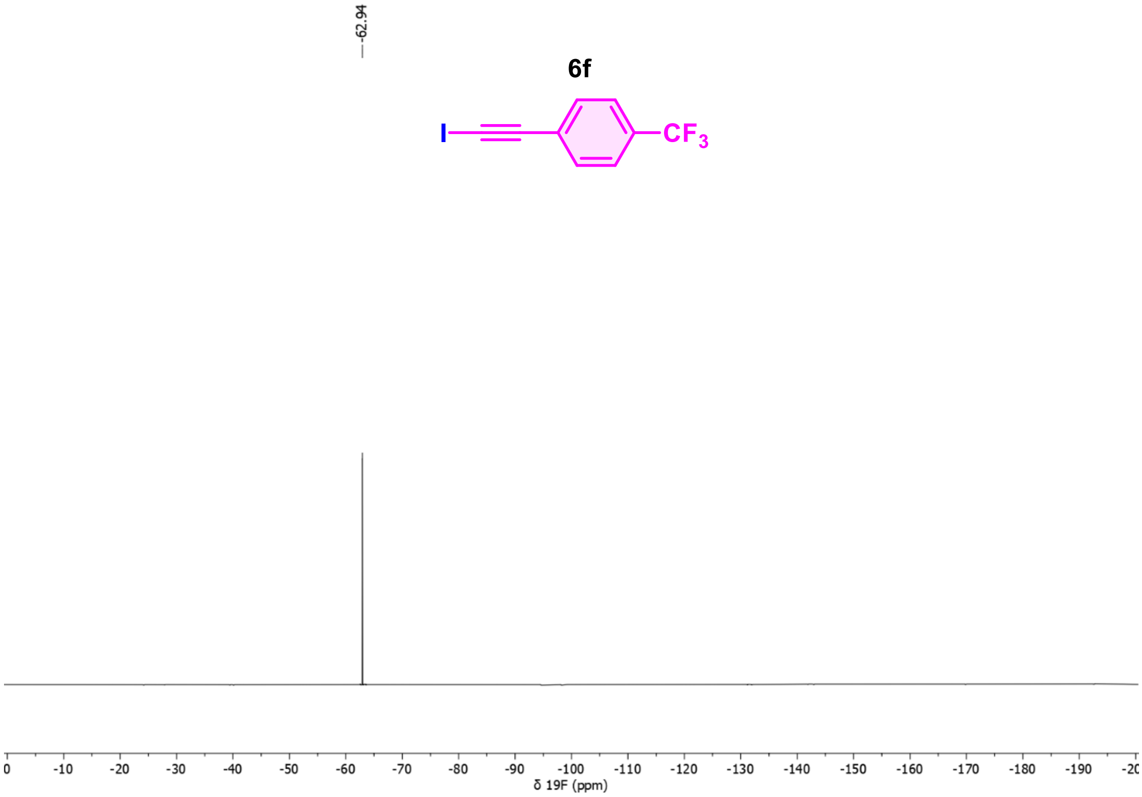

^{1}H NMR 300MHz, $CDCl_3$

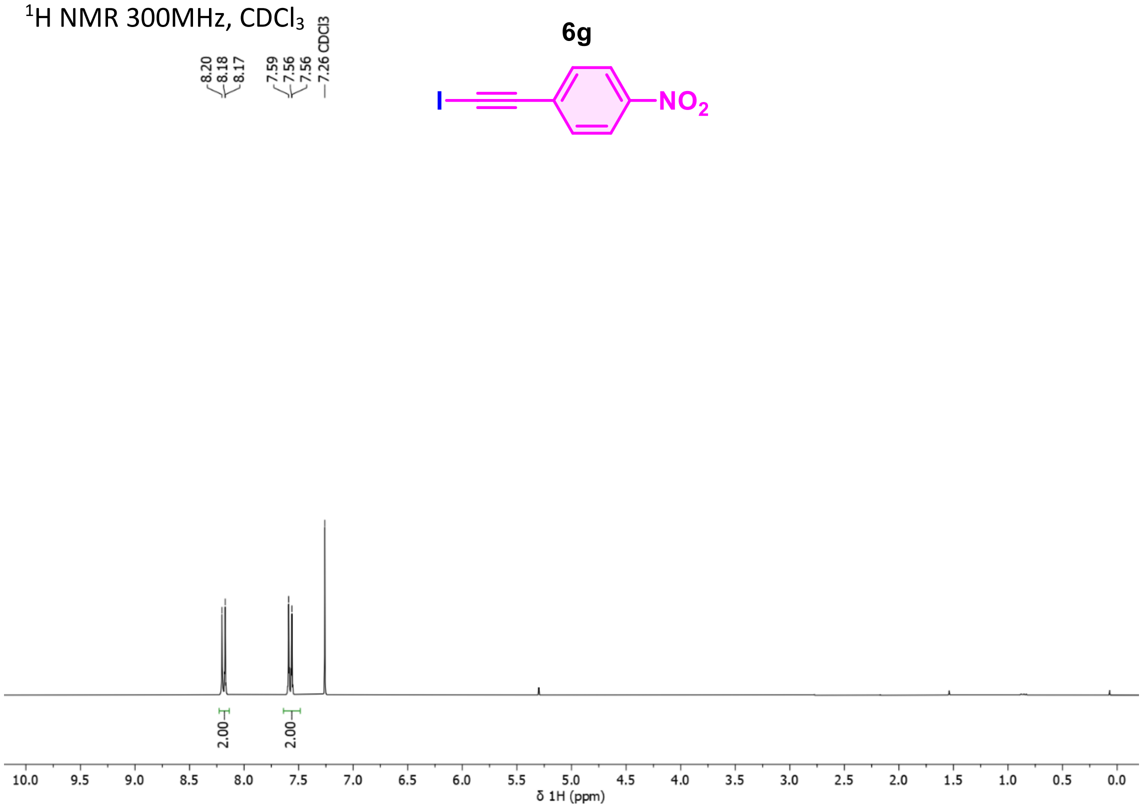

^{1}H NMR 300MHz, $CDCl_3$

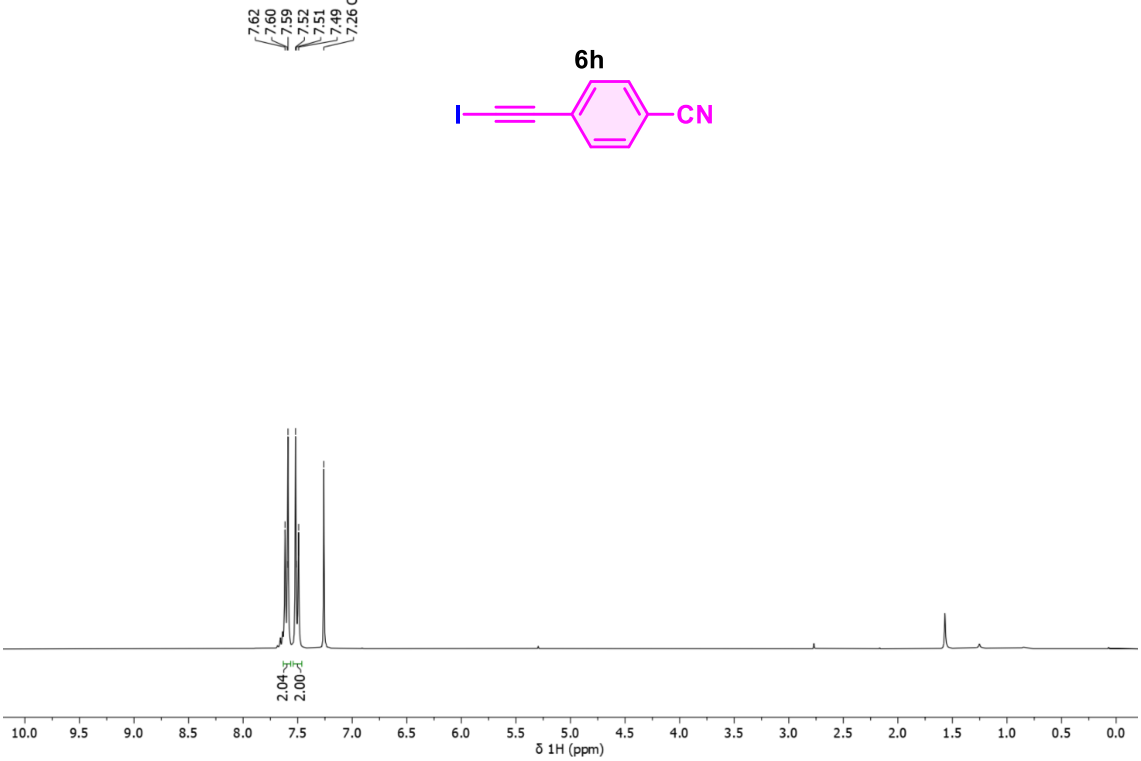

^{1}H NMR 300MHz, $CDCl_3$

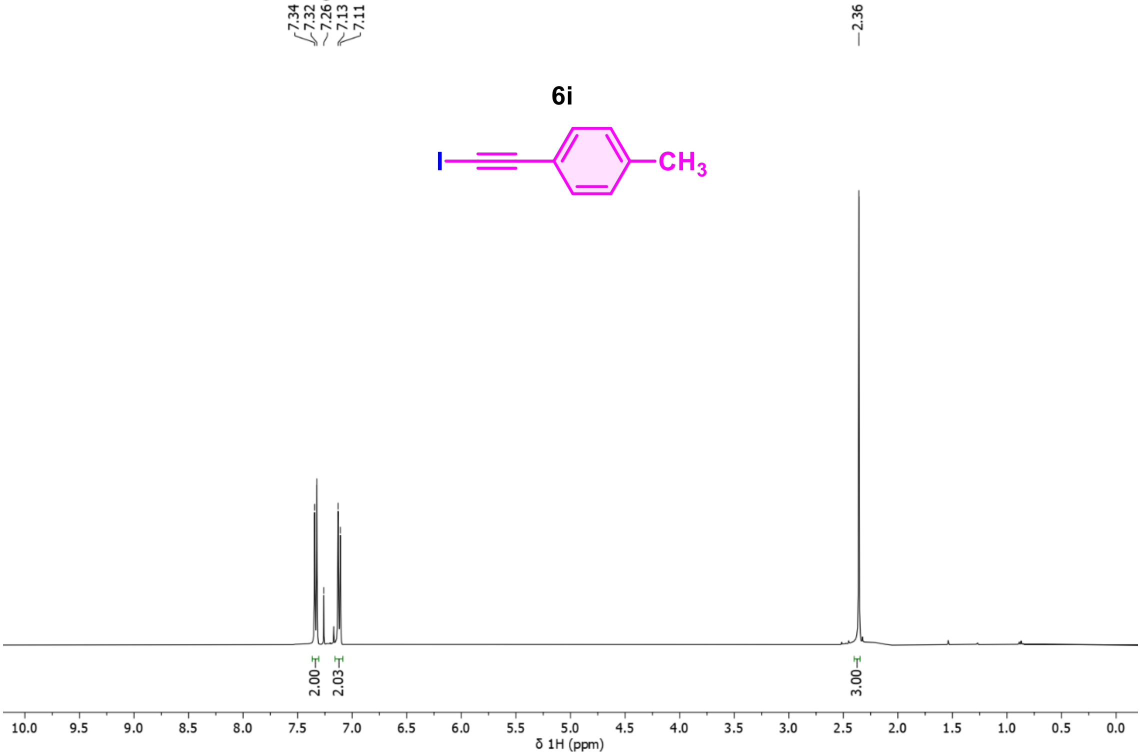

^1H NMR 300MHz, $CDCl_3$

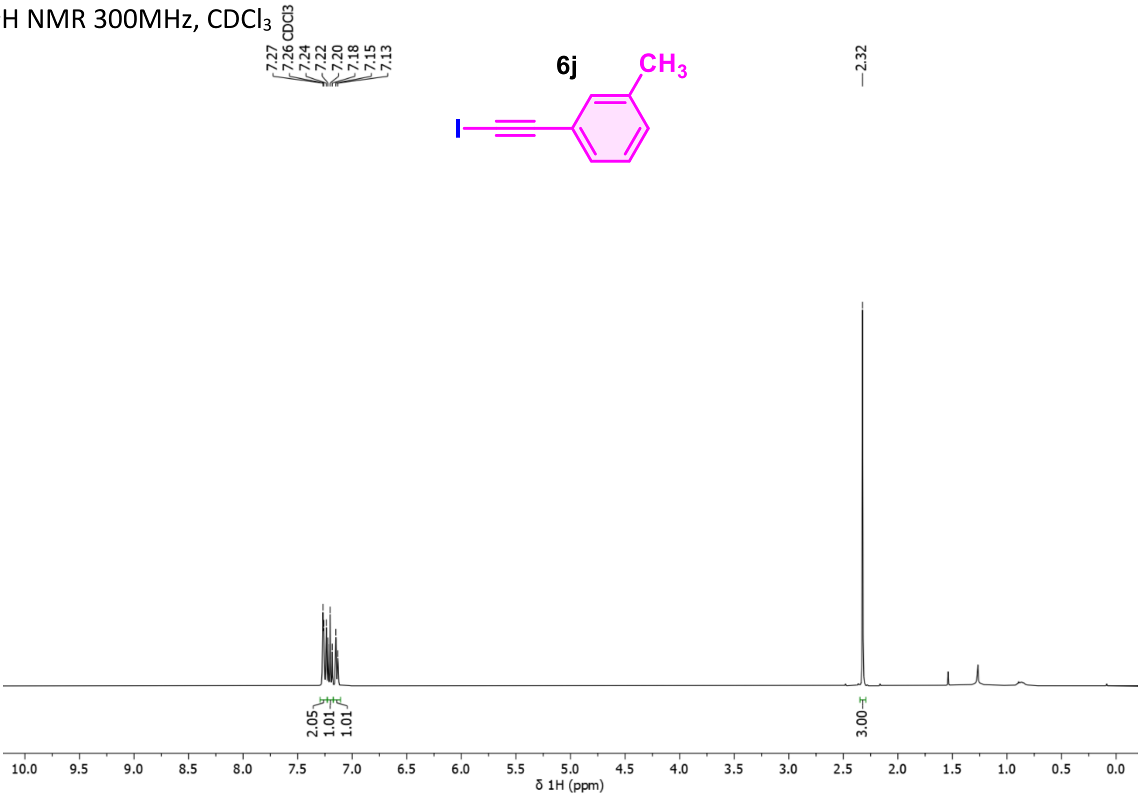

^1H NMR 300MHz, $CDCl_3$

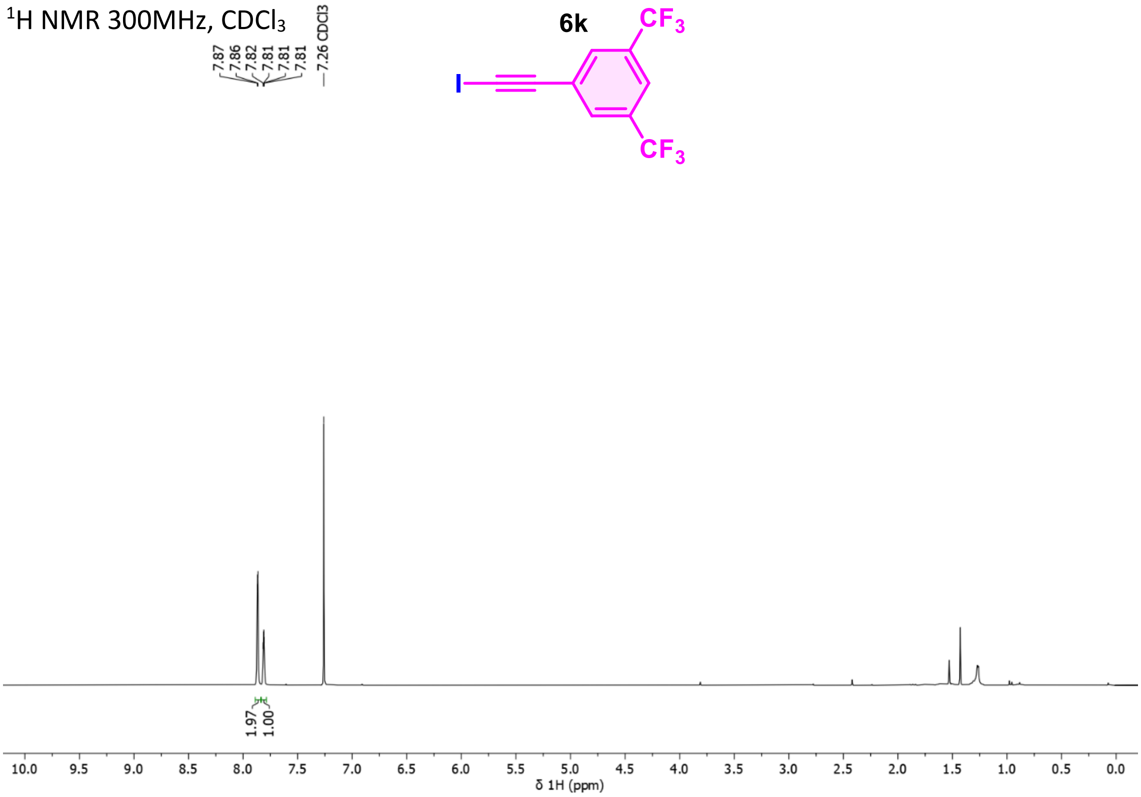

Table of contents

^{19}F NMR 282MHz, $CDCl_3$

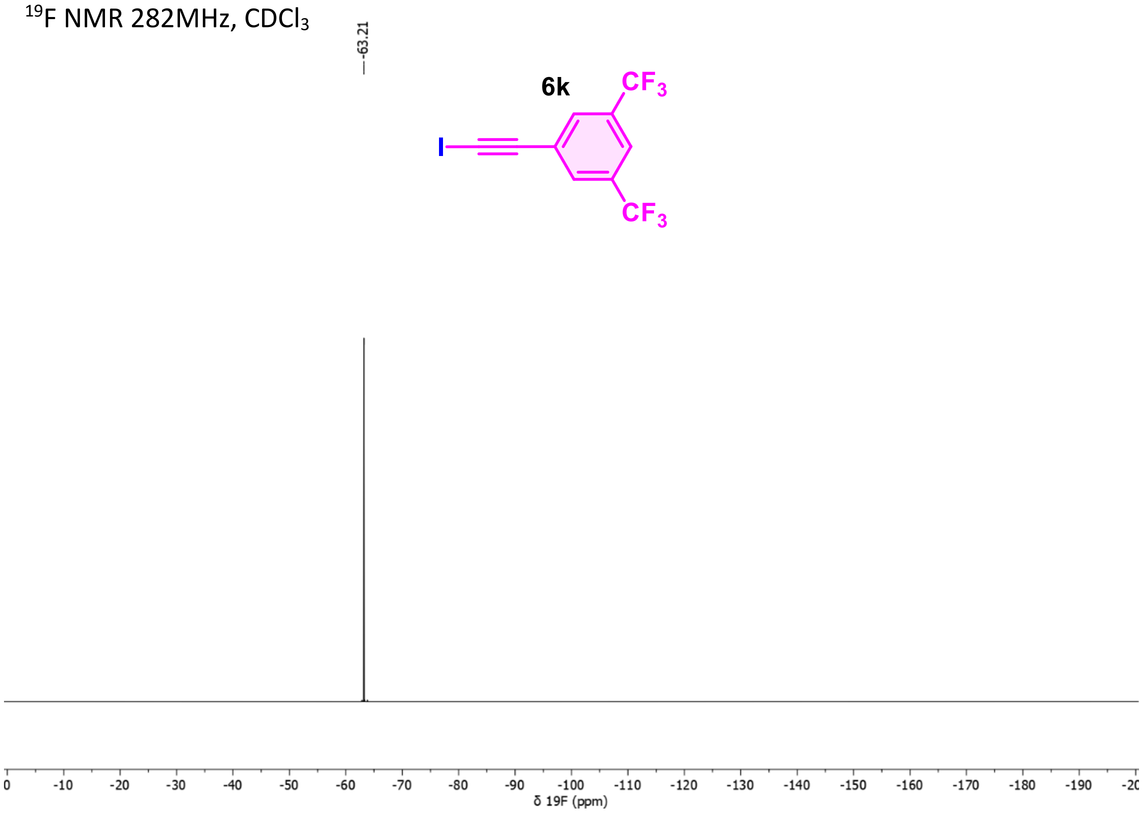

^{1}H NMR 300MHz, $CDCl_3$

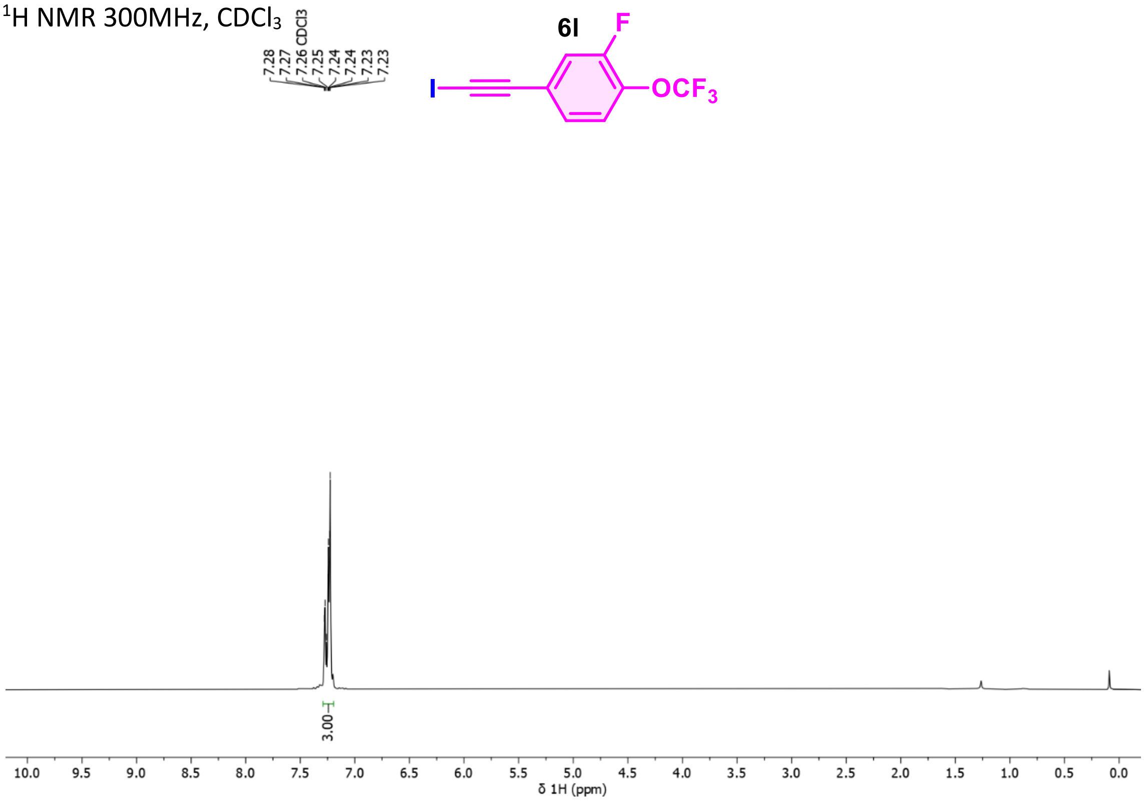

^{19}F NMR 282MHz, $CDCl_3$

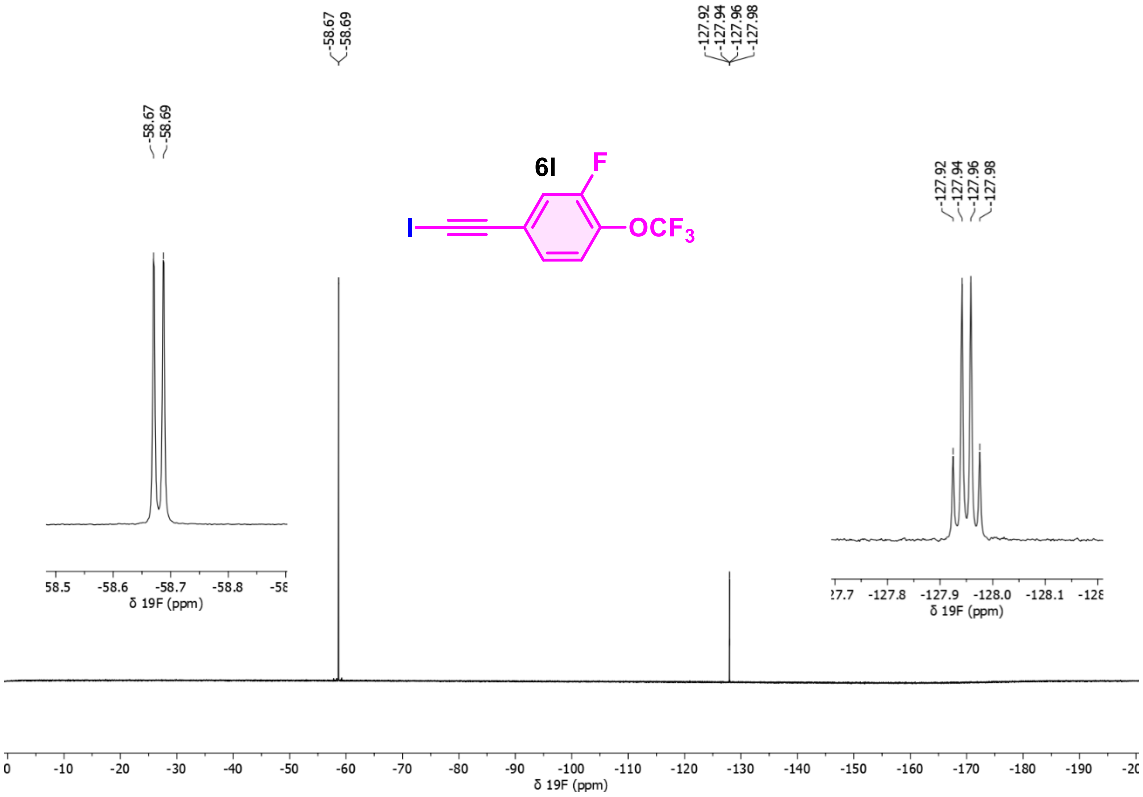

^{1}H NMR 300MHz, $CDCl_3$

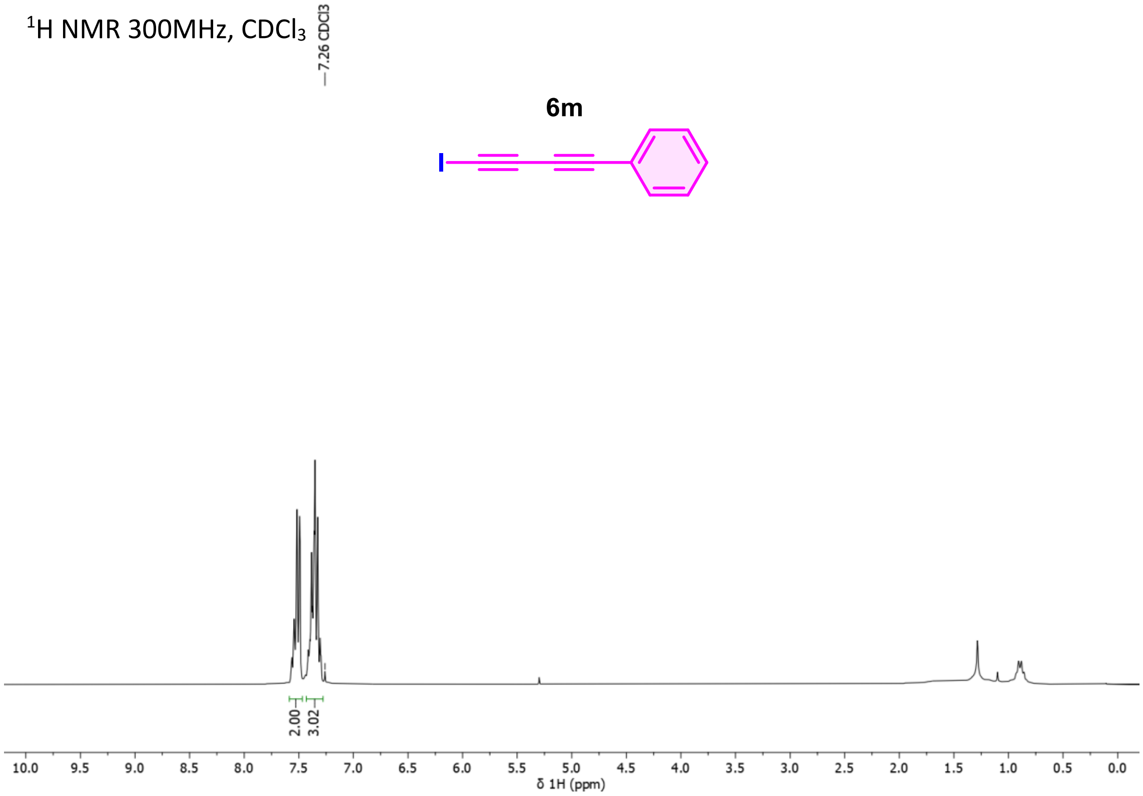

^1H NMR 300MHz, $CDCl_3$

6n

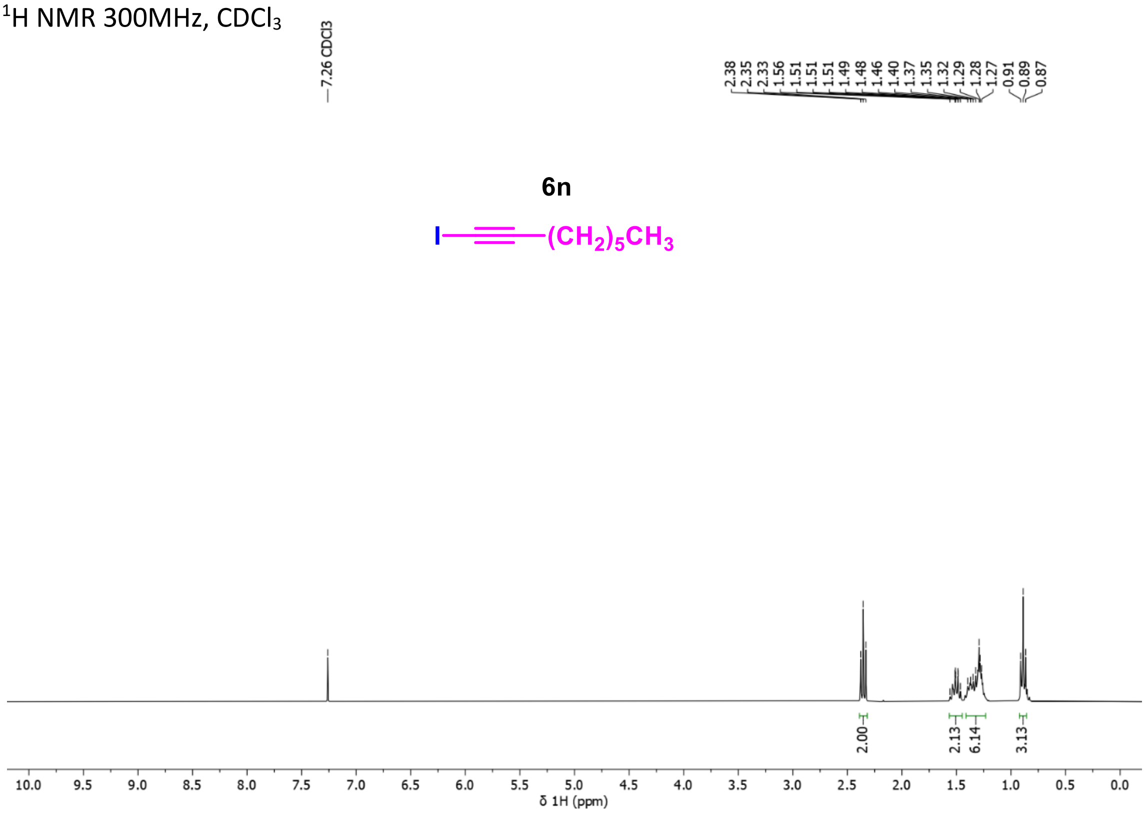

^1H NMR 300MHz, $CDCl_3$

6o

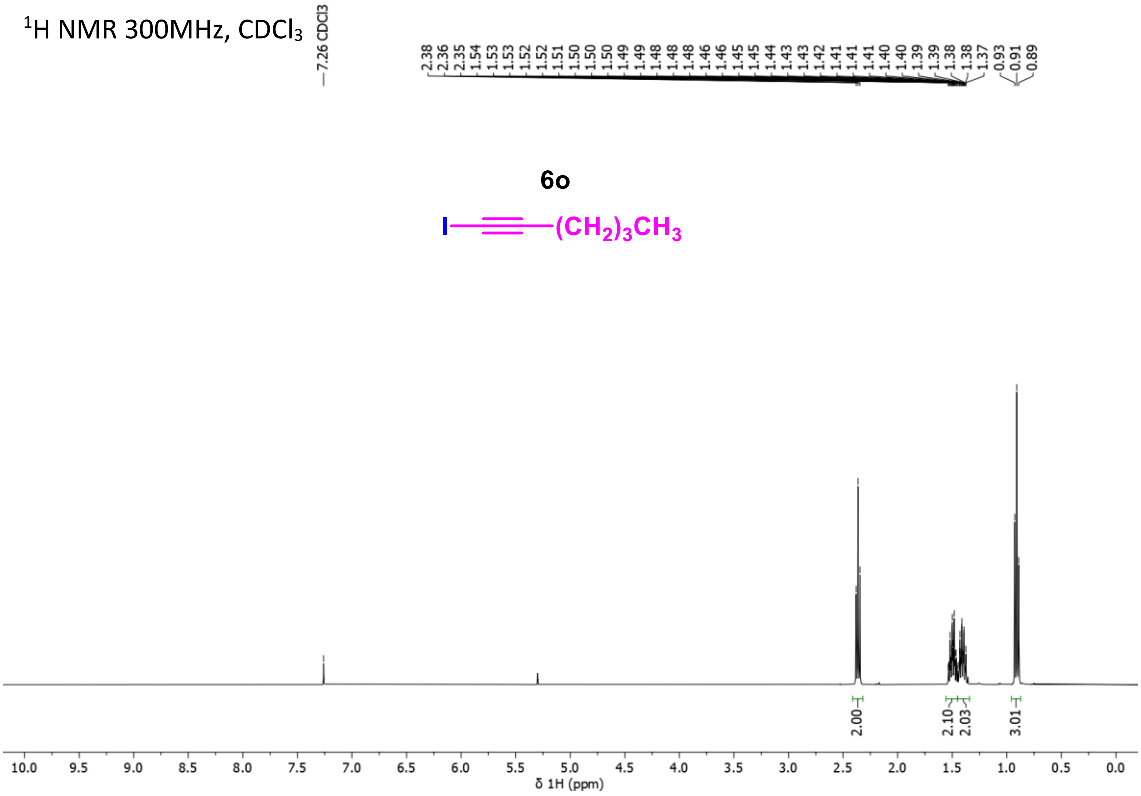

^{1}H NMR 300MHz, $CDCl_3$

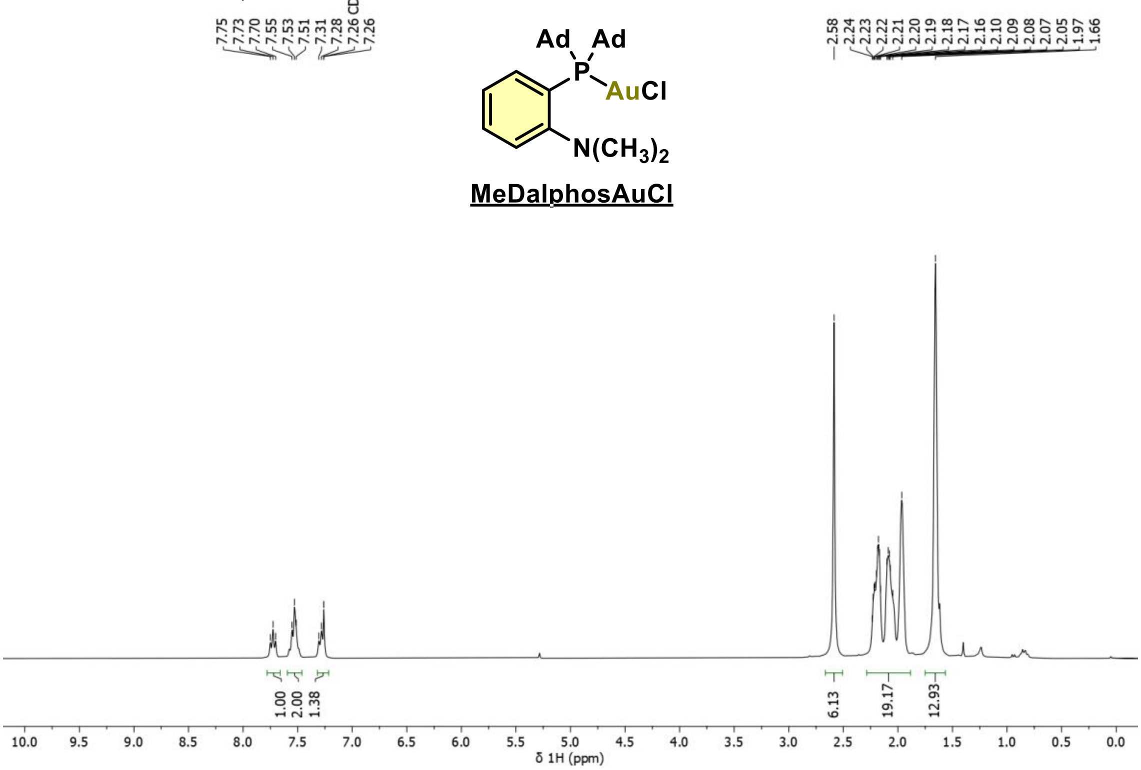

^{31}P NMR 162MHz, $CDCl_3$

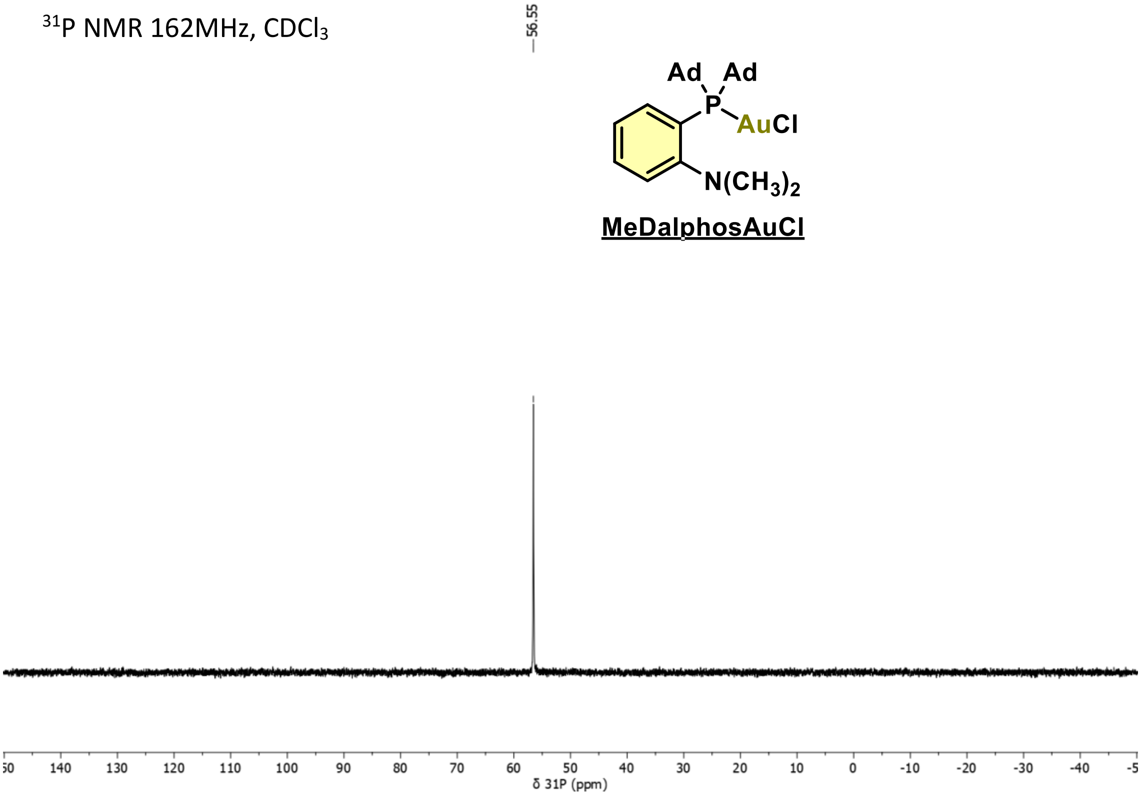

Table of contents

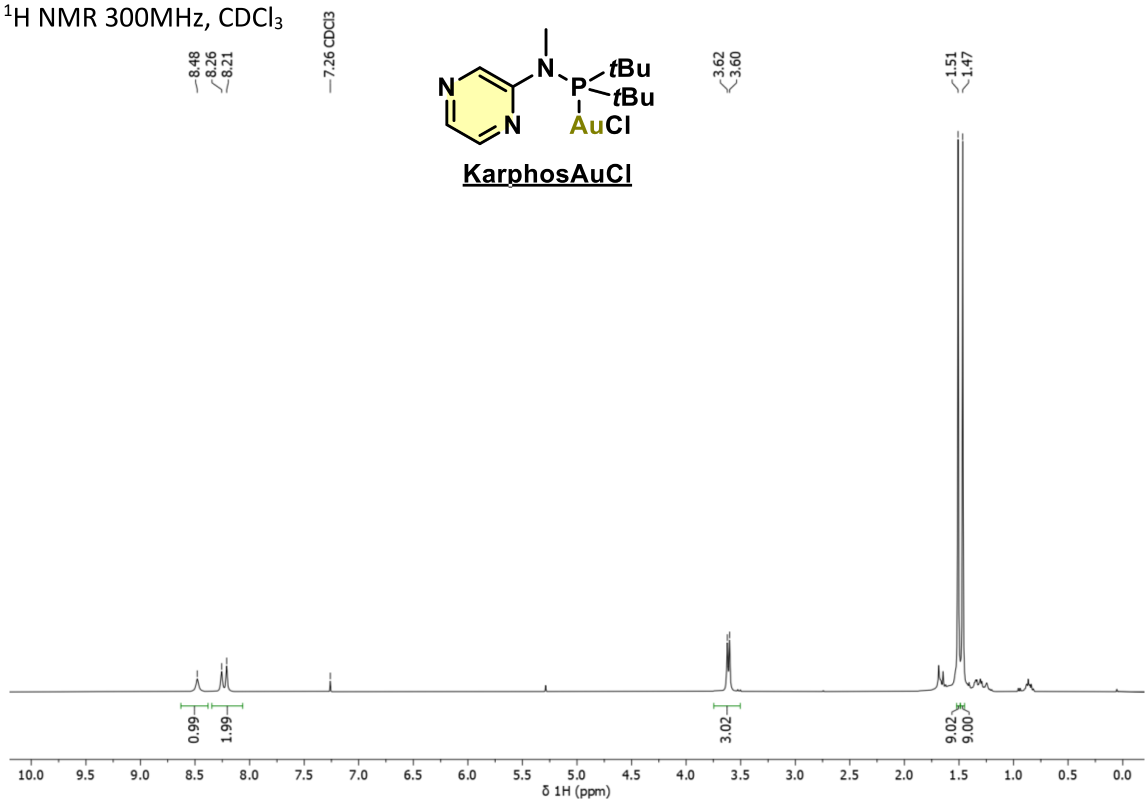

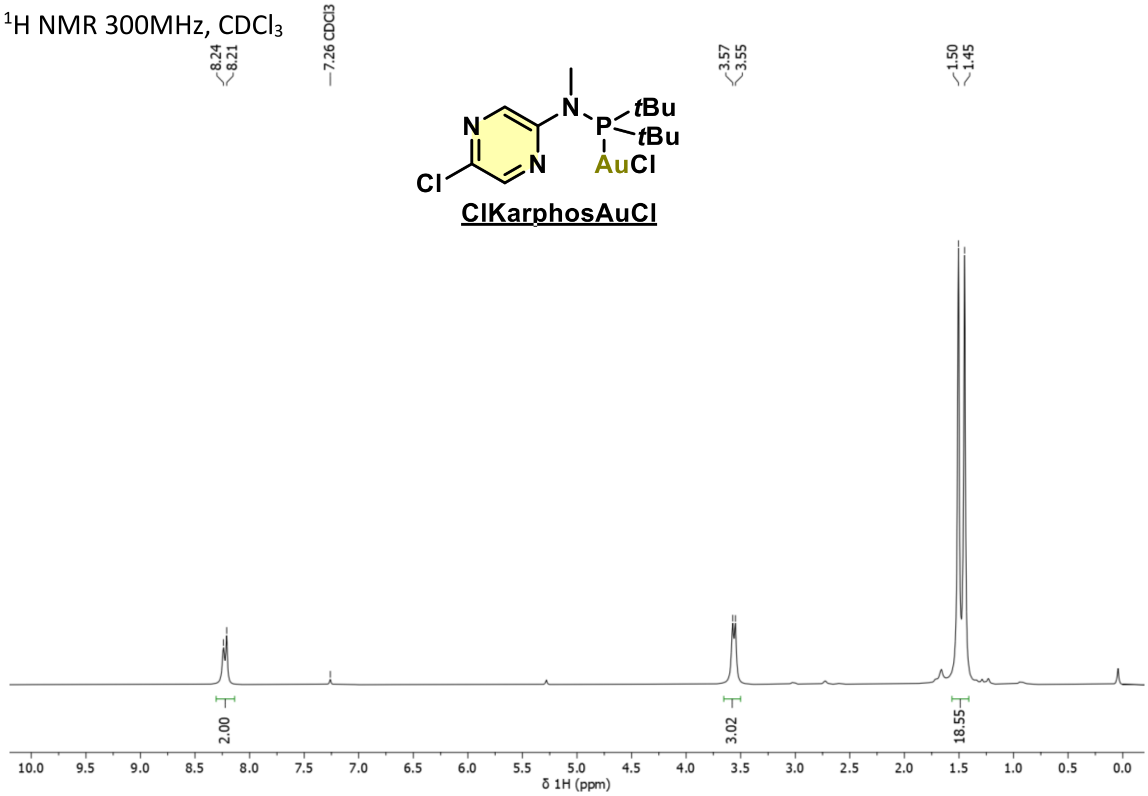

Table of contents

^{31}P NMR 162MHz, $CDCl_3$

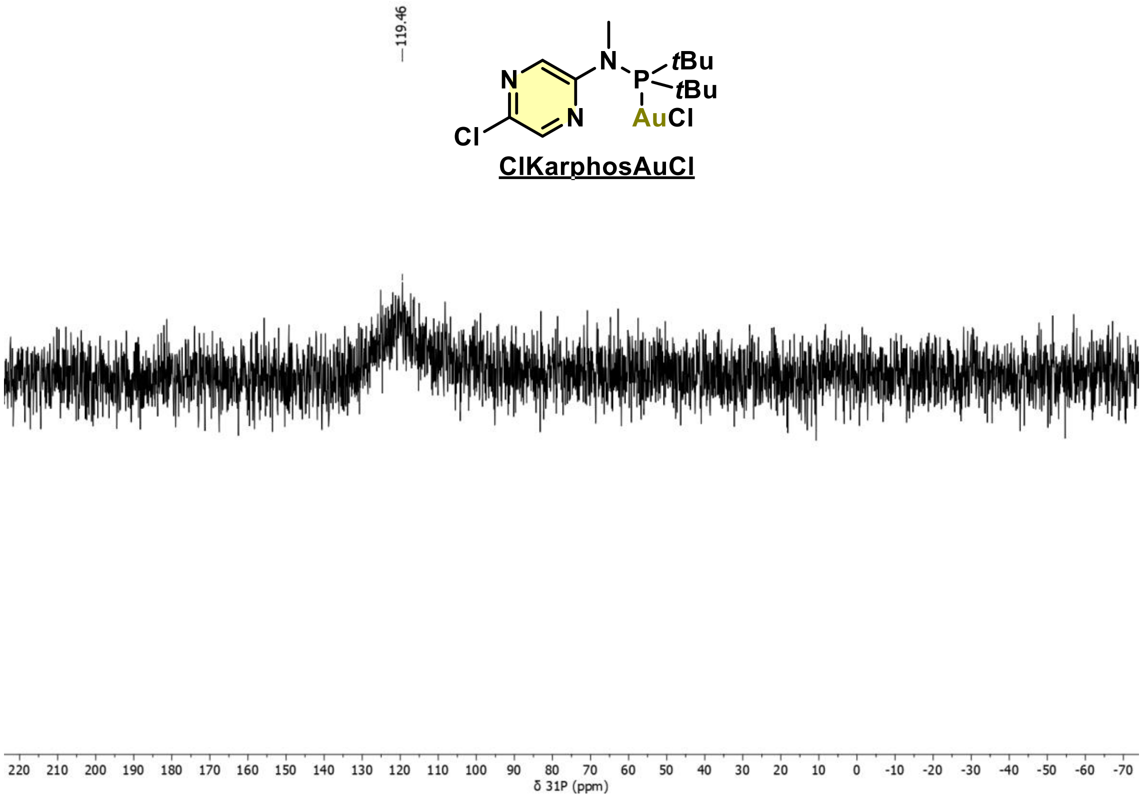

^{1}H NMR 300MHz, $CDCl_3$

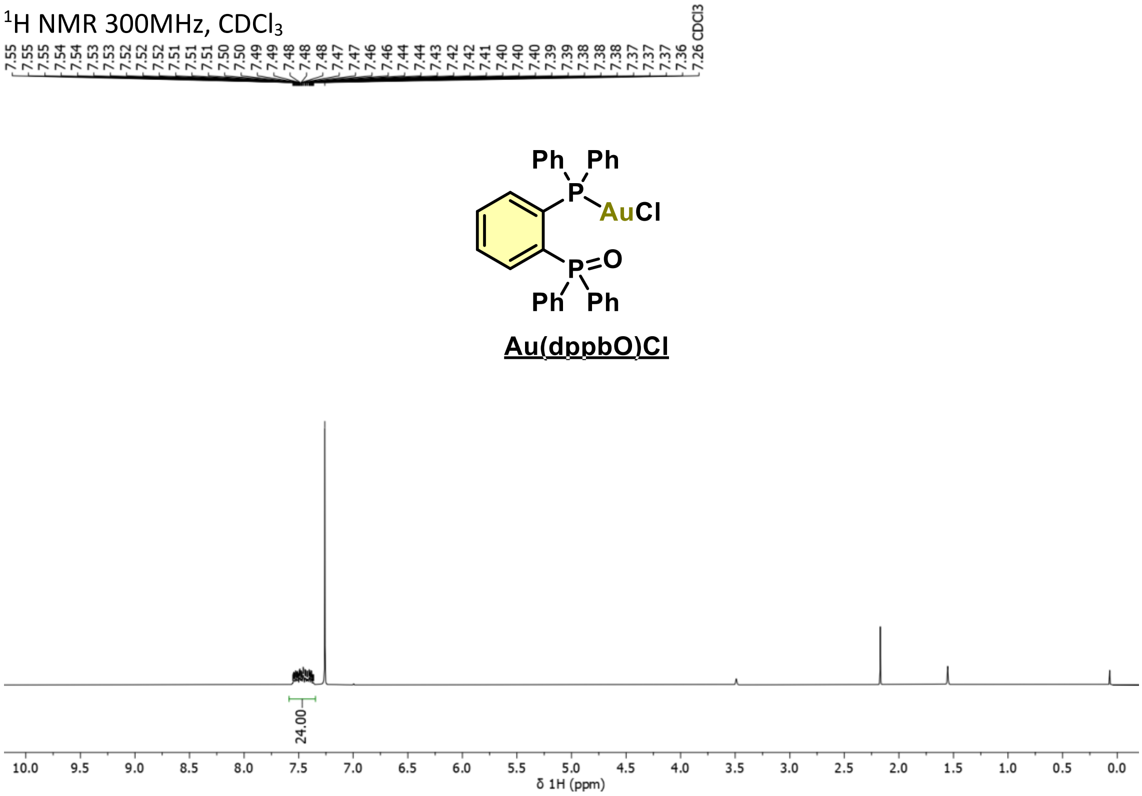

Table of contents

^{31}P NMR 162MHz, $CDCl_3$

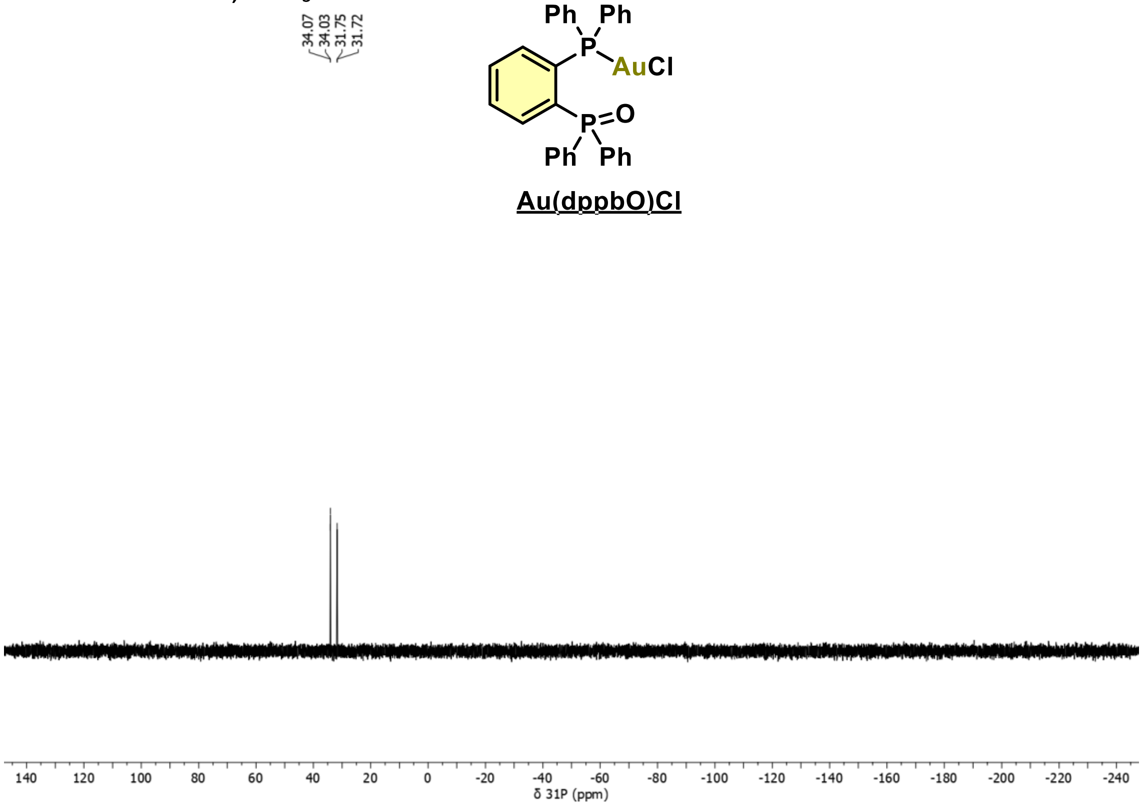

^{1}H NMR 400MHz, $CDCl_3$

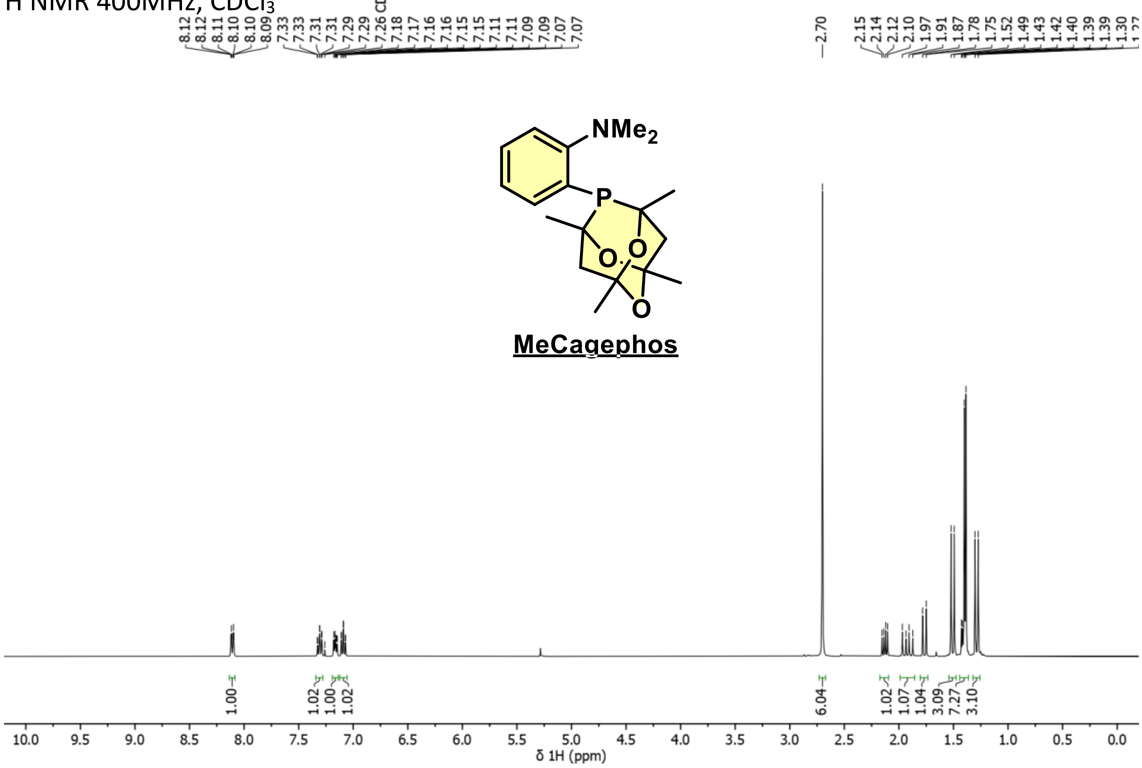

^{31}P NMR 162MHz, $CDCl_3$

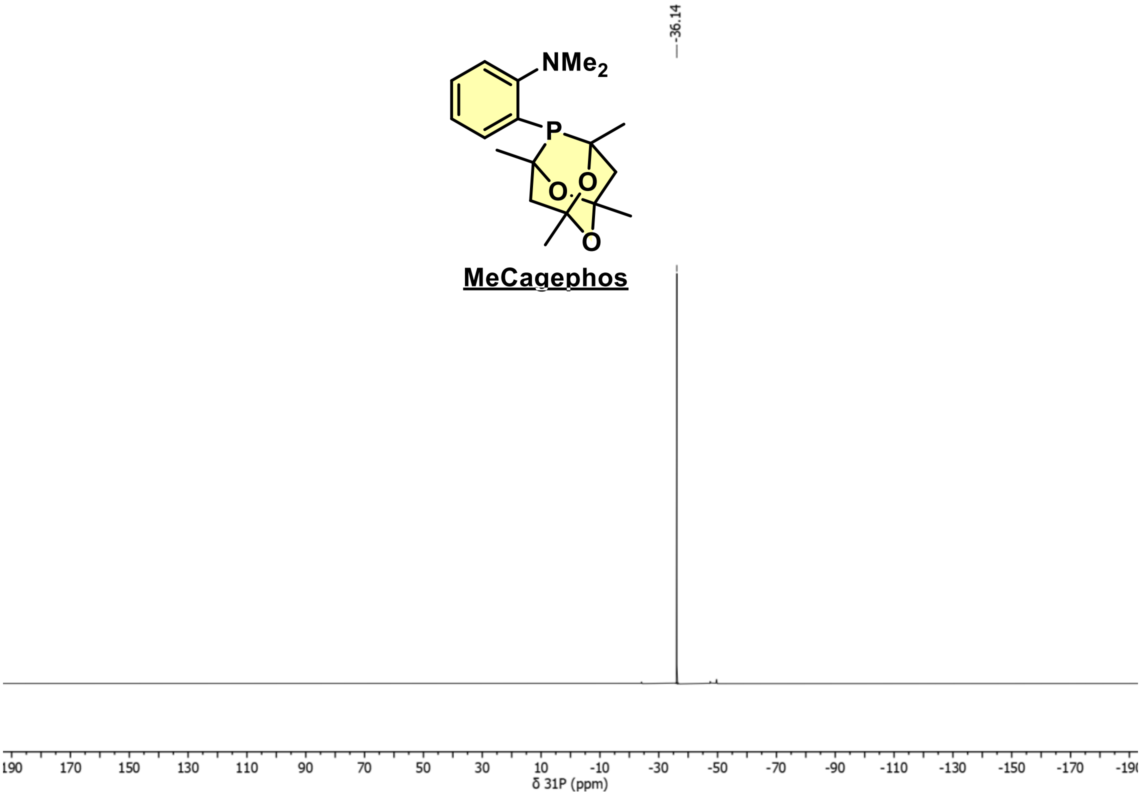

^{1}H NMR 400MHz, $CDCl_3$

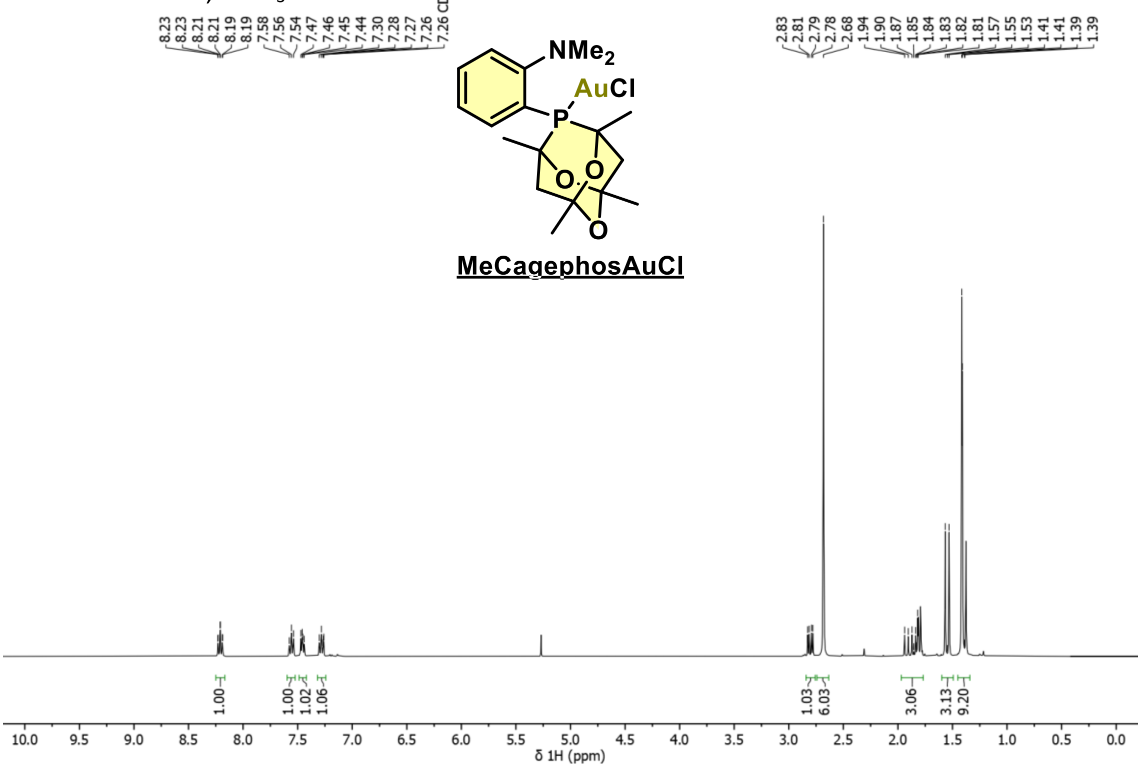

^{13}C NMR 101MHz, $CDCl_3$

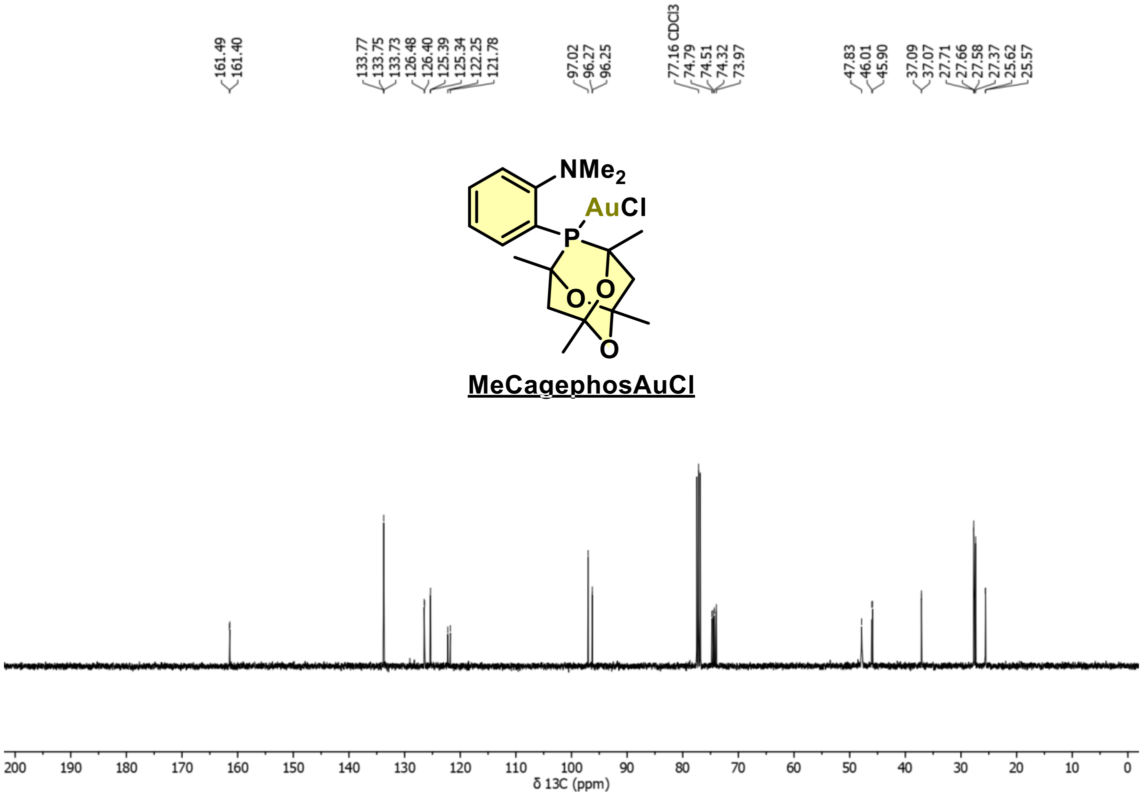

^{31}P NMR 162MHz, $CDCl_3$

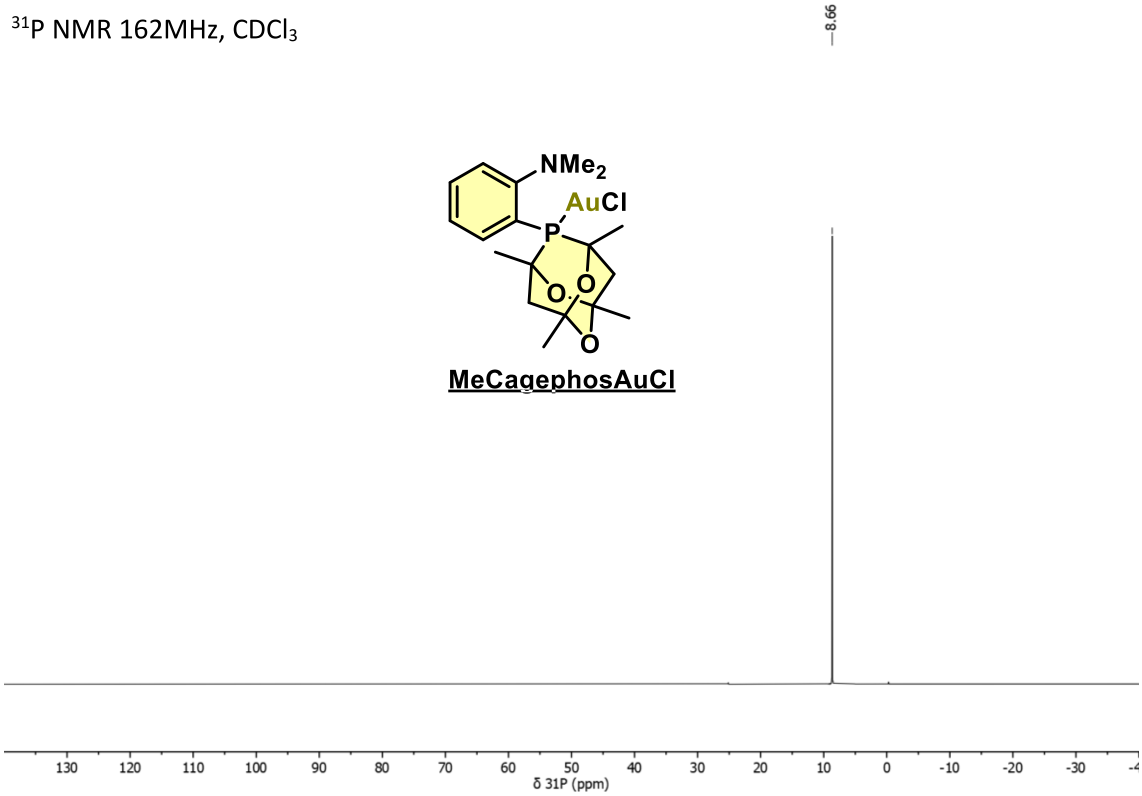

^1H NMR 300MHz, $CDCl_3$

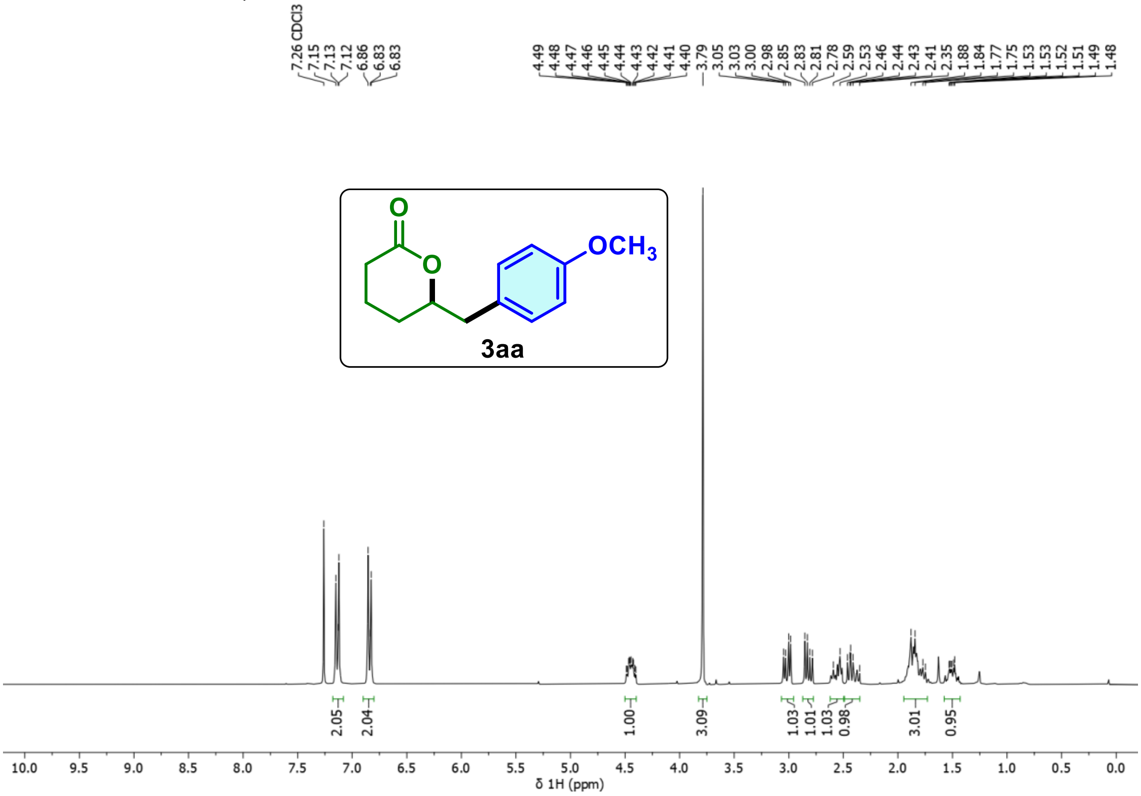

^{13}C NMR 101MHz, $CDCl_3$

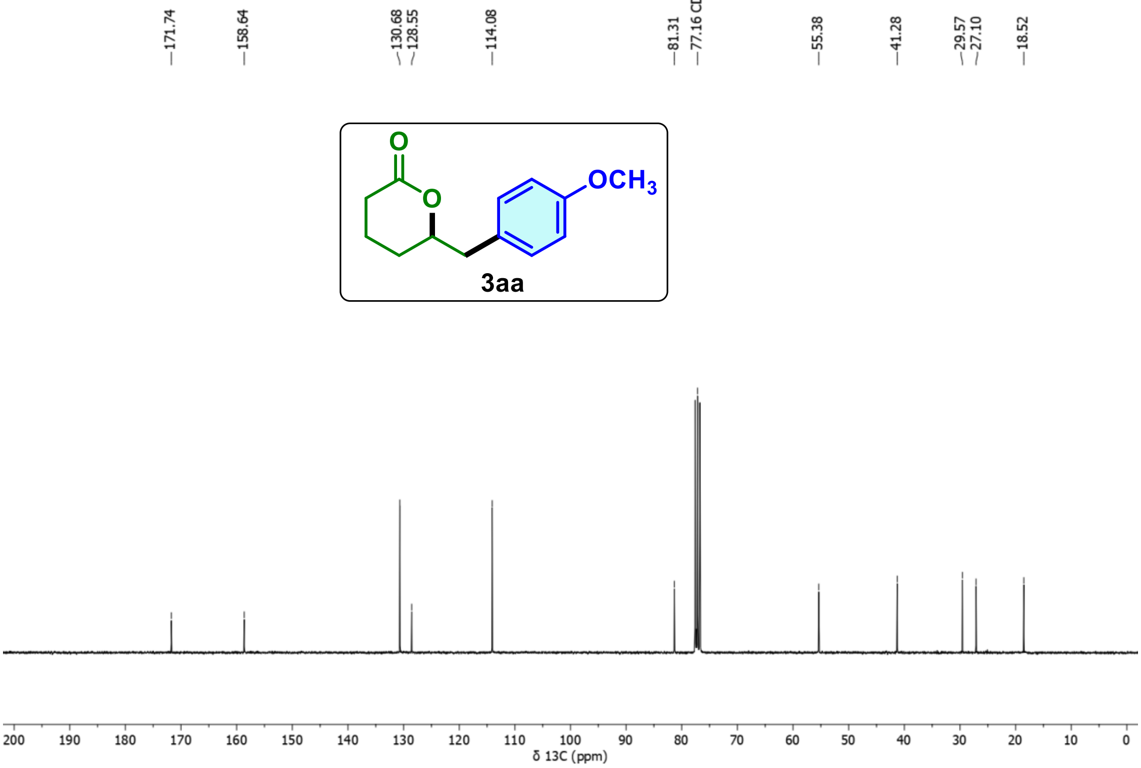

Table of contents

^{1}H NMR 300MHz, $CDCl_3$

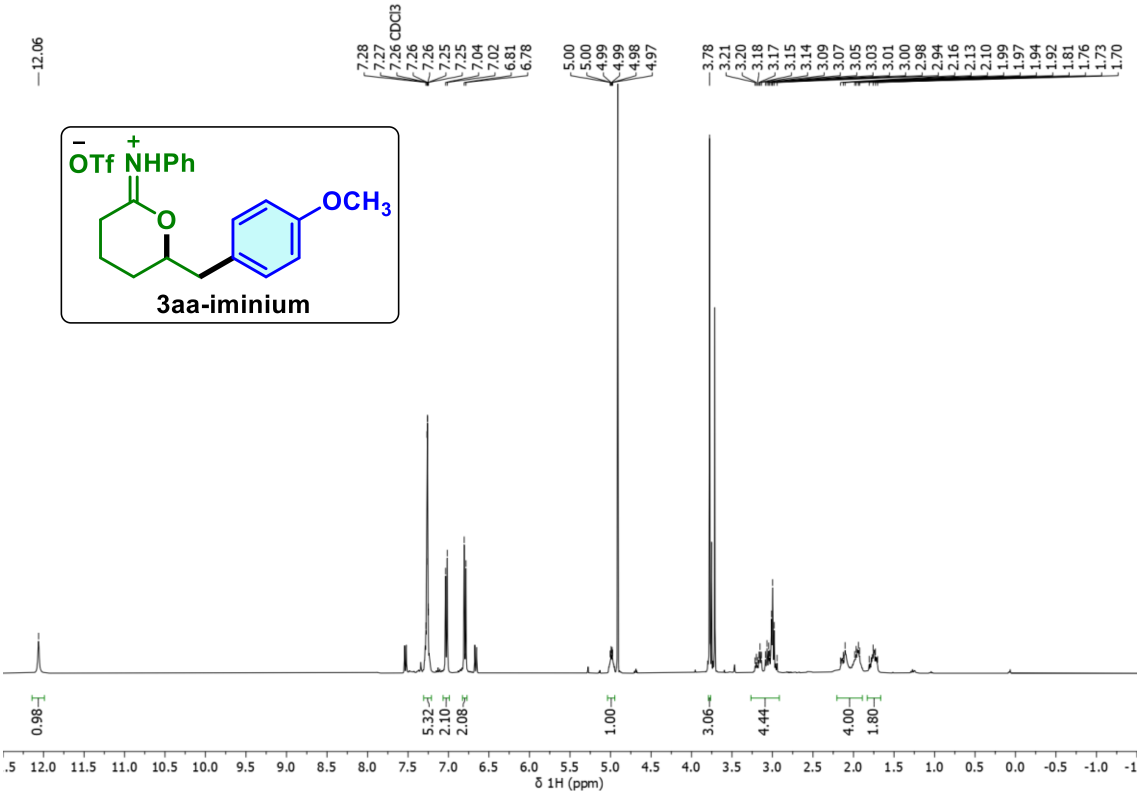

^{13}C NMR 101MHz, $CDCl_3$

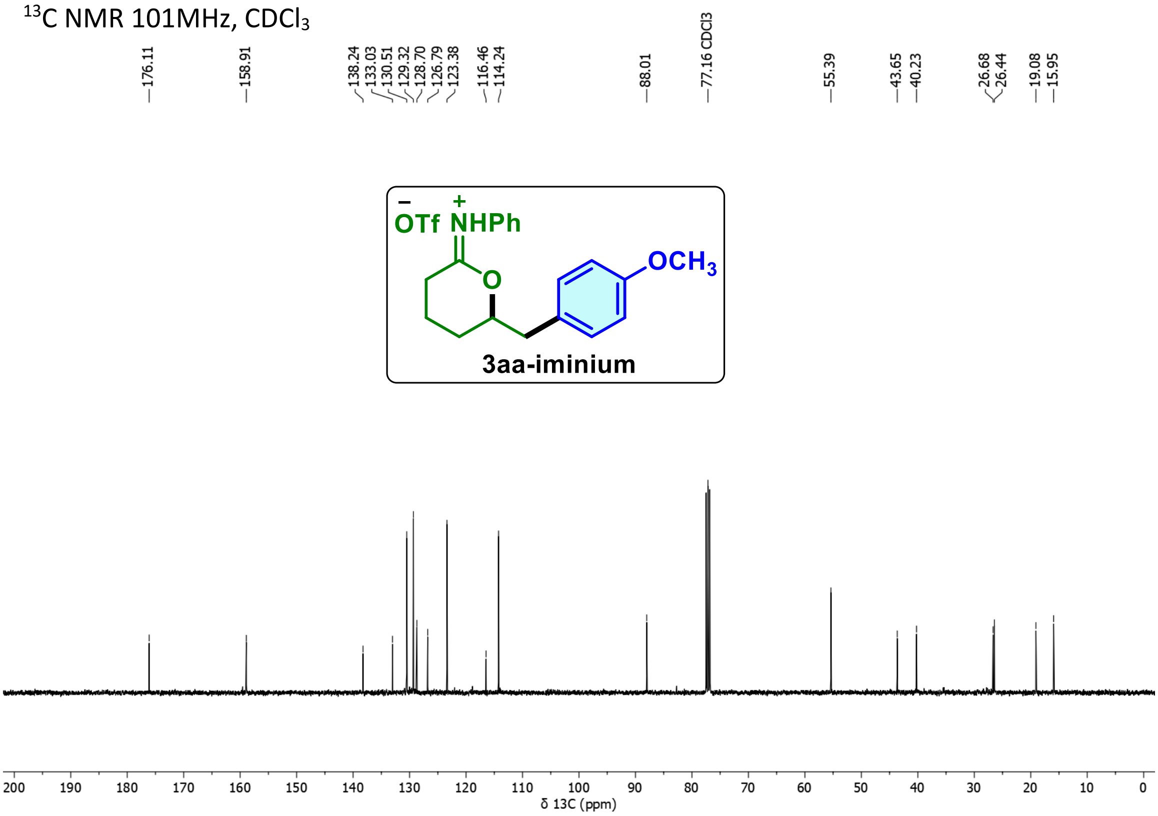

Table of contents

^{19}F NMR 282MHz, $CDCl_3$

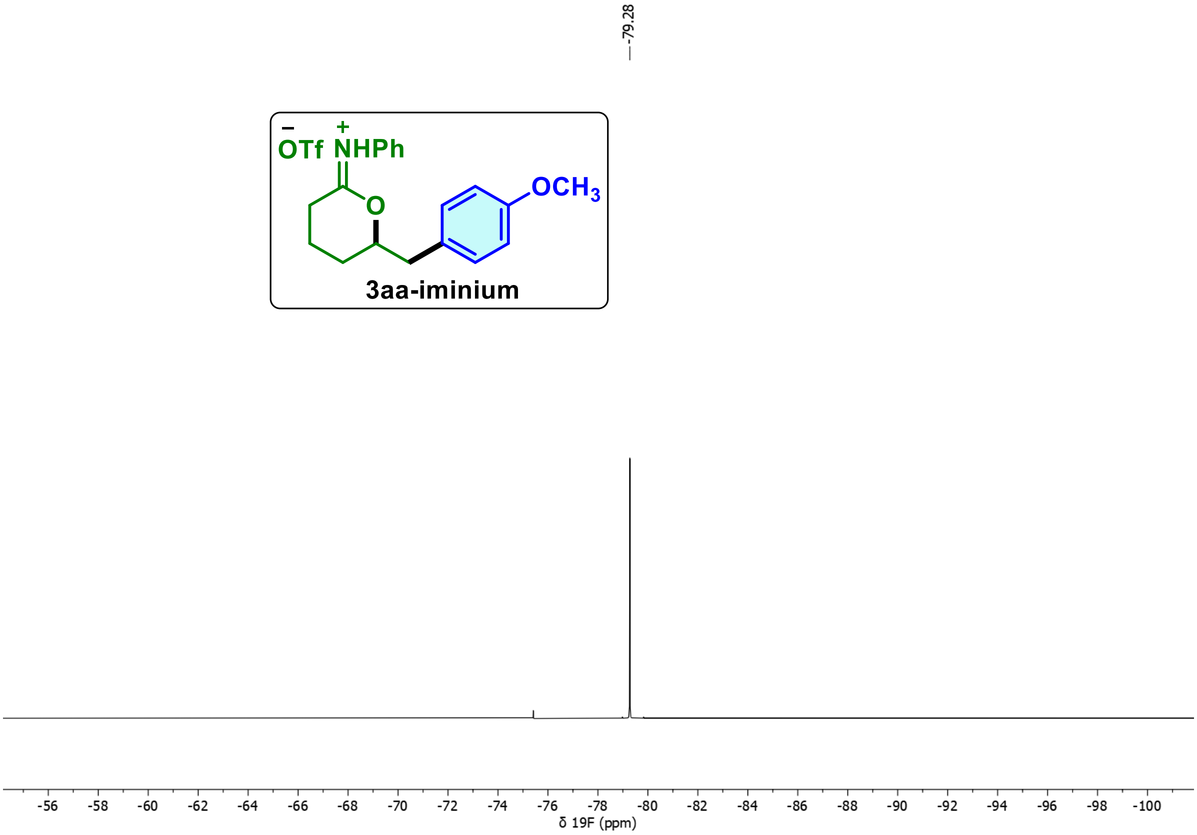

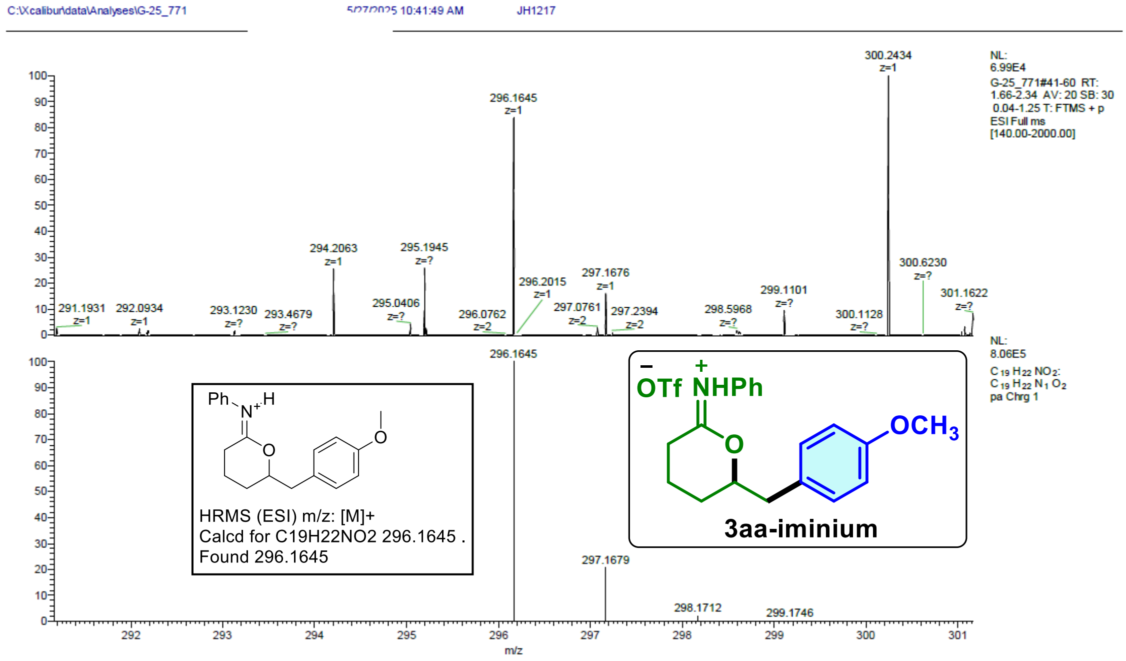

^{1}H NMR 300MHz, $CDCl_3$

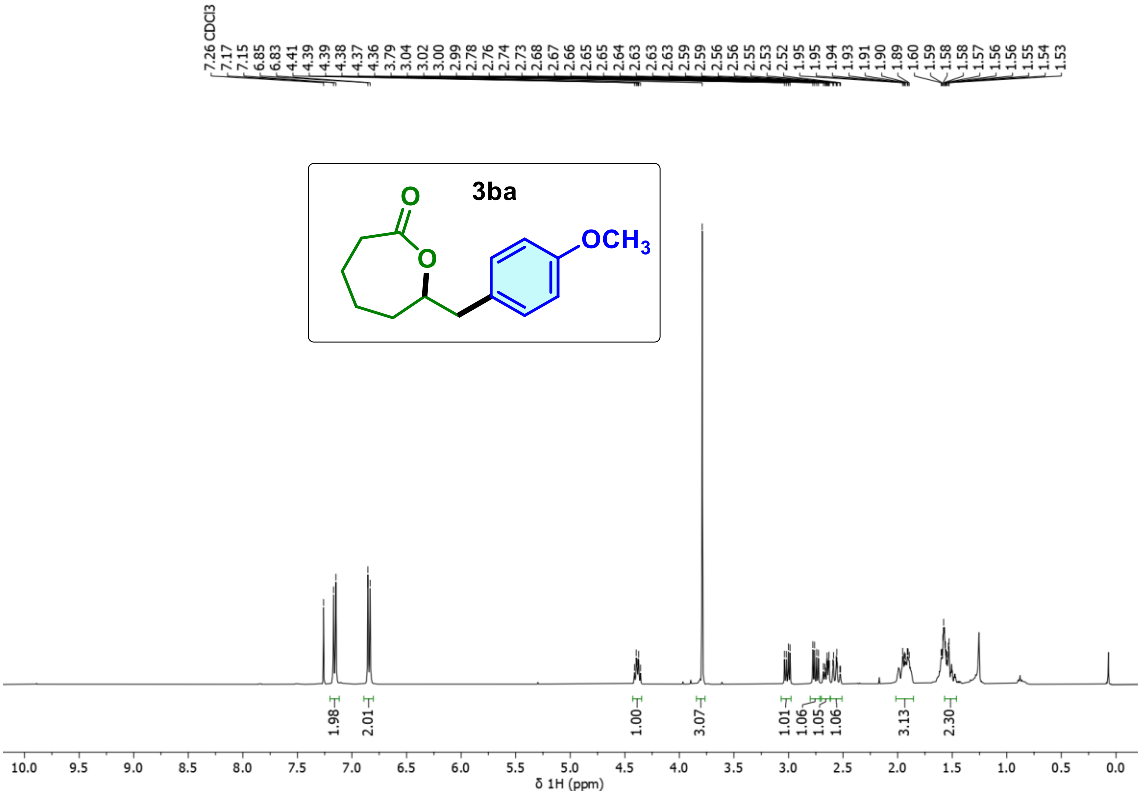

^{13}C NMR 101MHz, $CDCl_3$

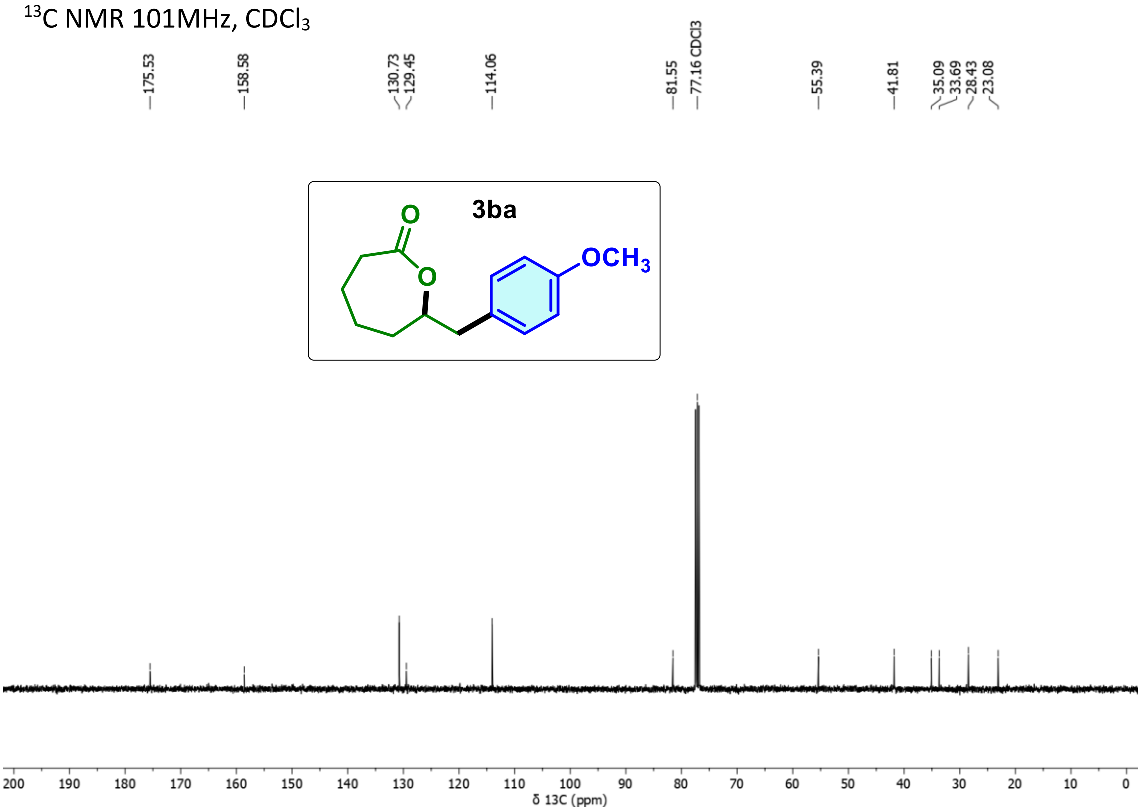

^{1}H NMR 300MHz, $CDCl_3$

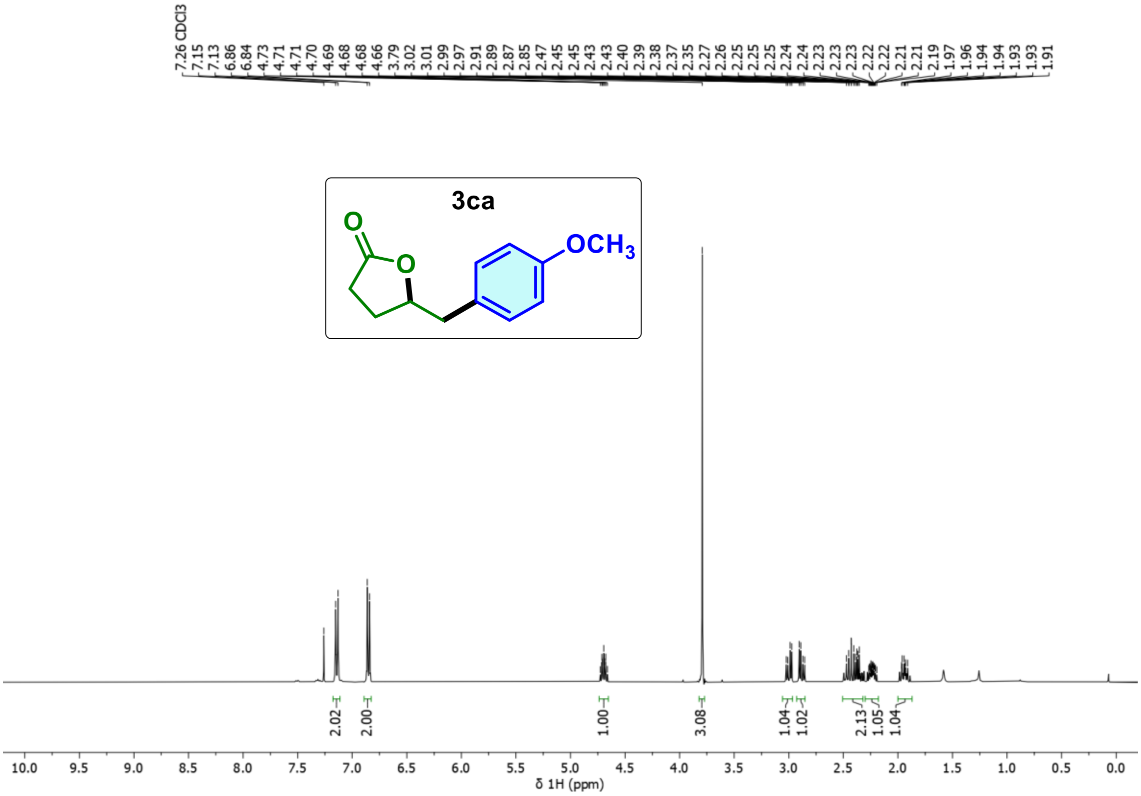

^{13}C NMR 101MHz, $CDCl_3$

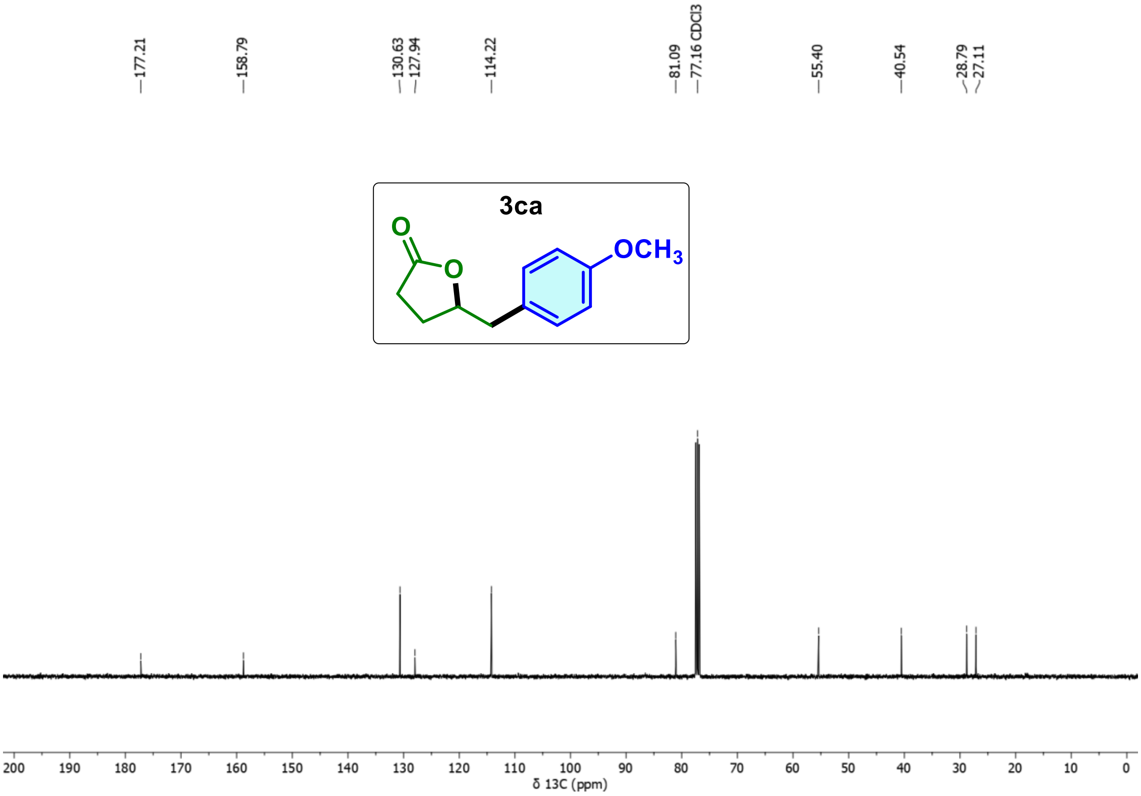

Table of contents

^{1}H NMR 300MHz, $CDCl_3$

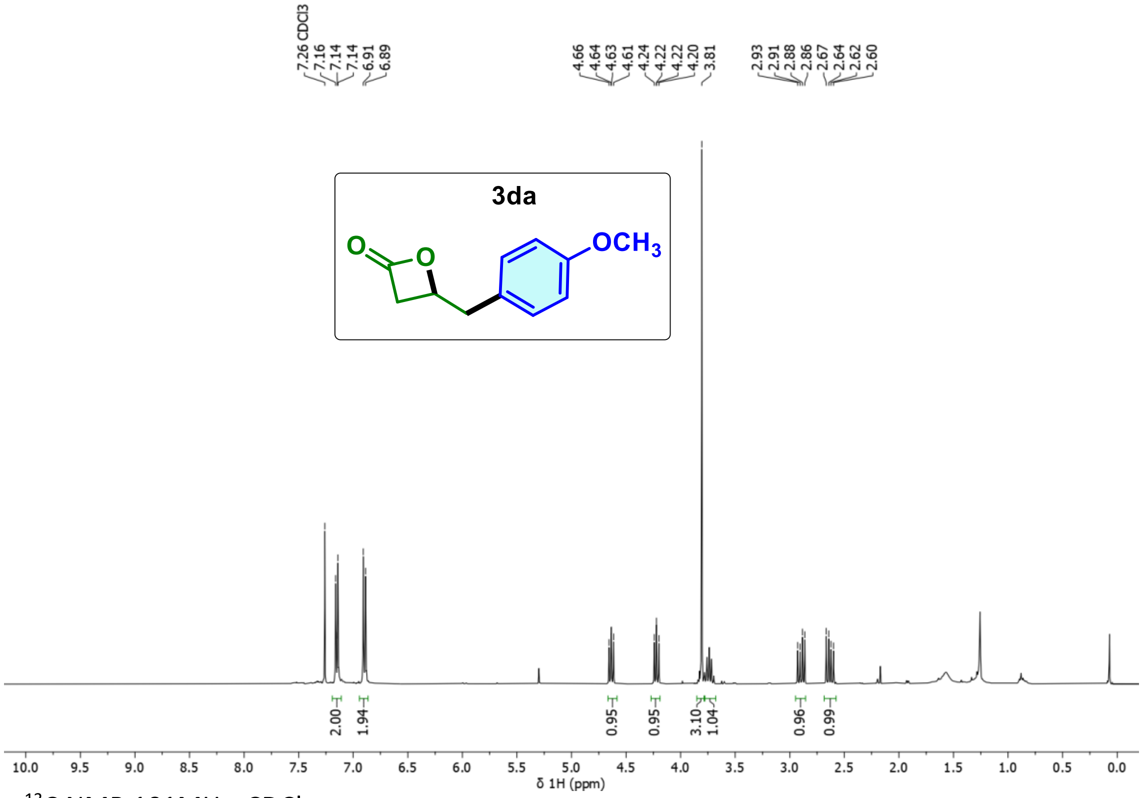

^{13}C NMR 101MHz, $CDCl_3$

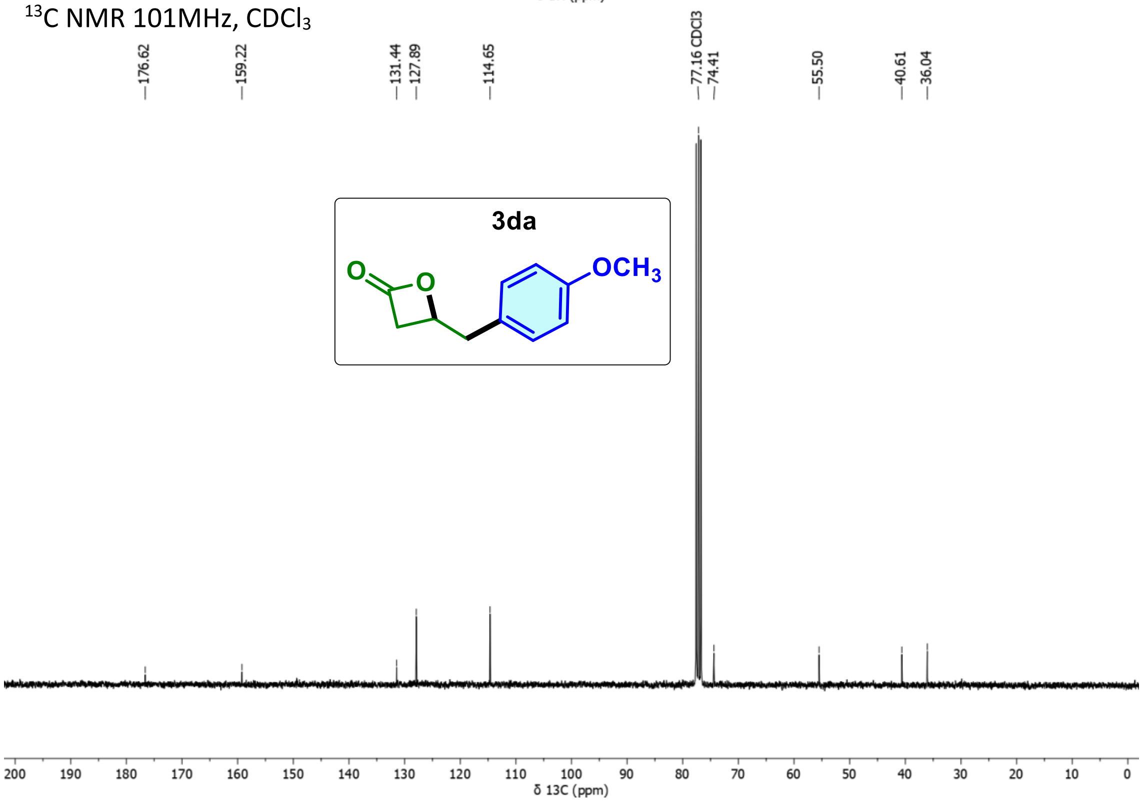

^{1}H NMR 300MHz, $CDCl_3$

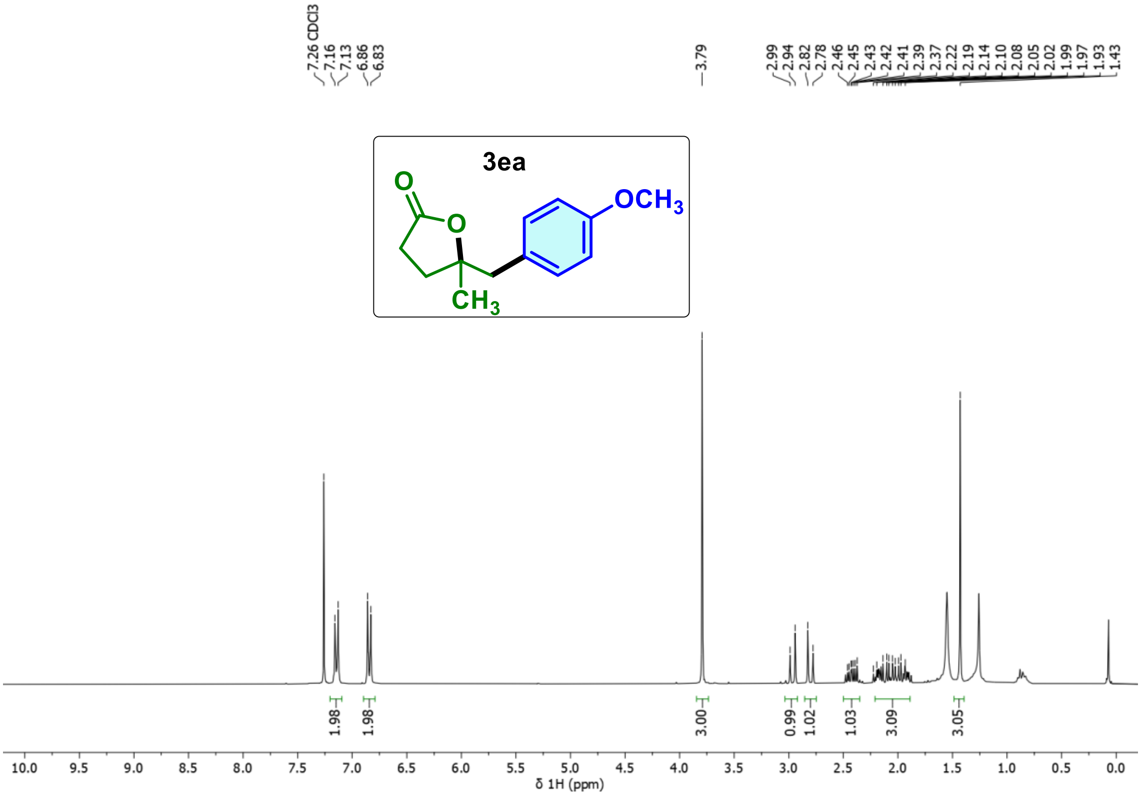

^{13}C NMR 101MHz, $CDCl_3$

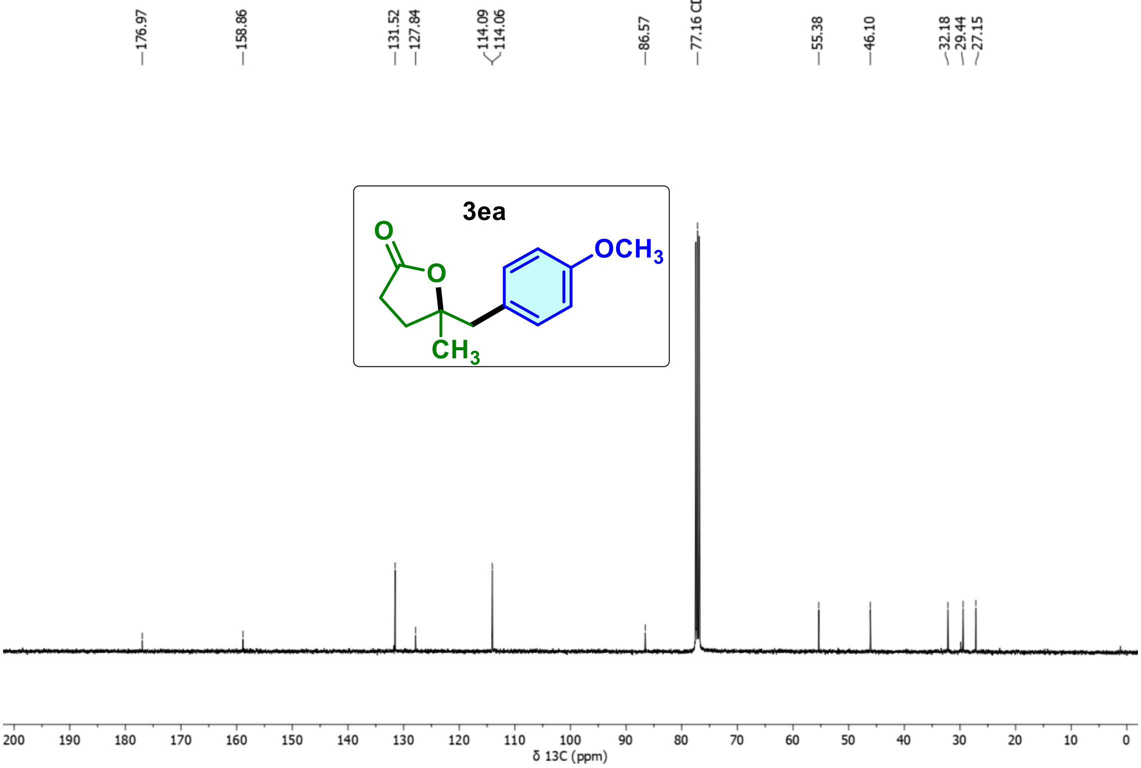

Table of contents

^{1}H NMR 300MHz, $CDCl_3$

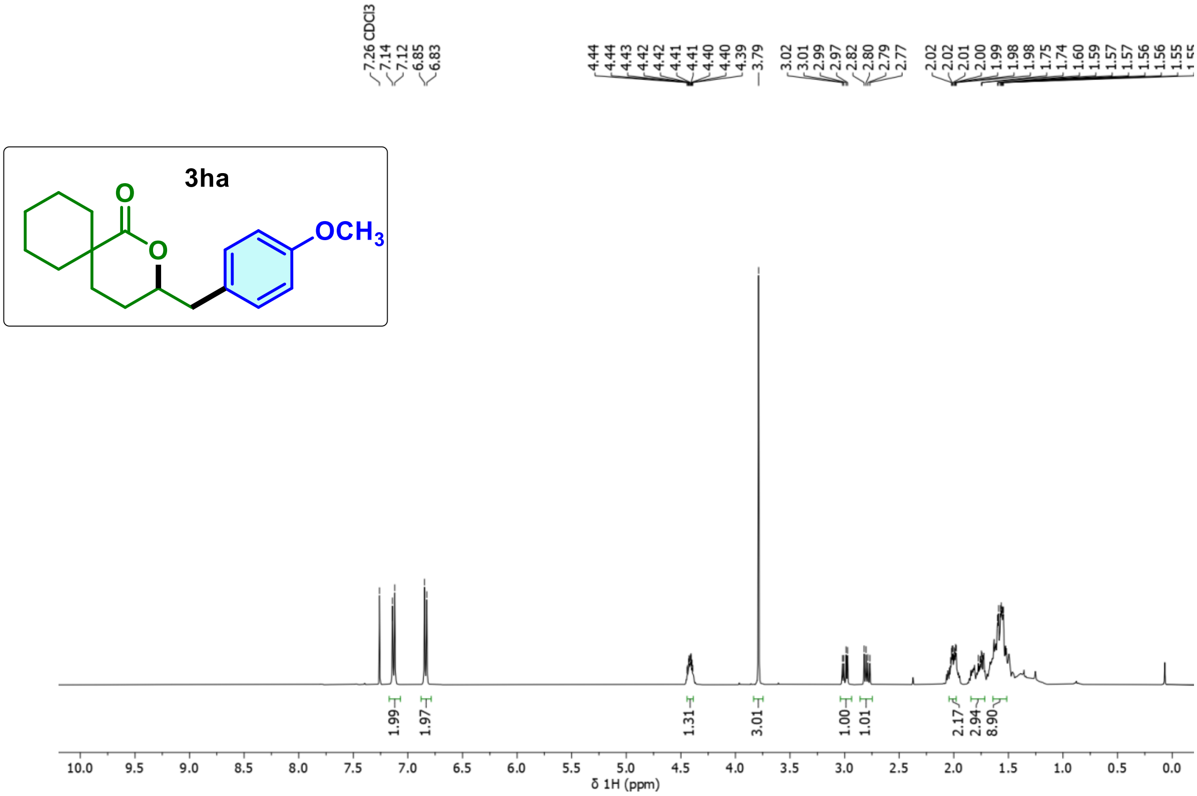

^{13}C NMR 101MHz, $CDCl_3$

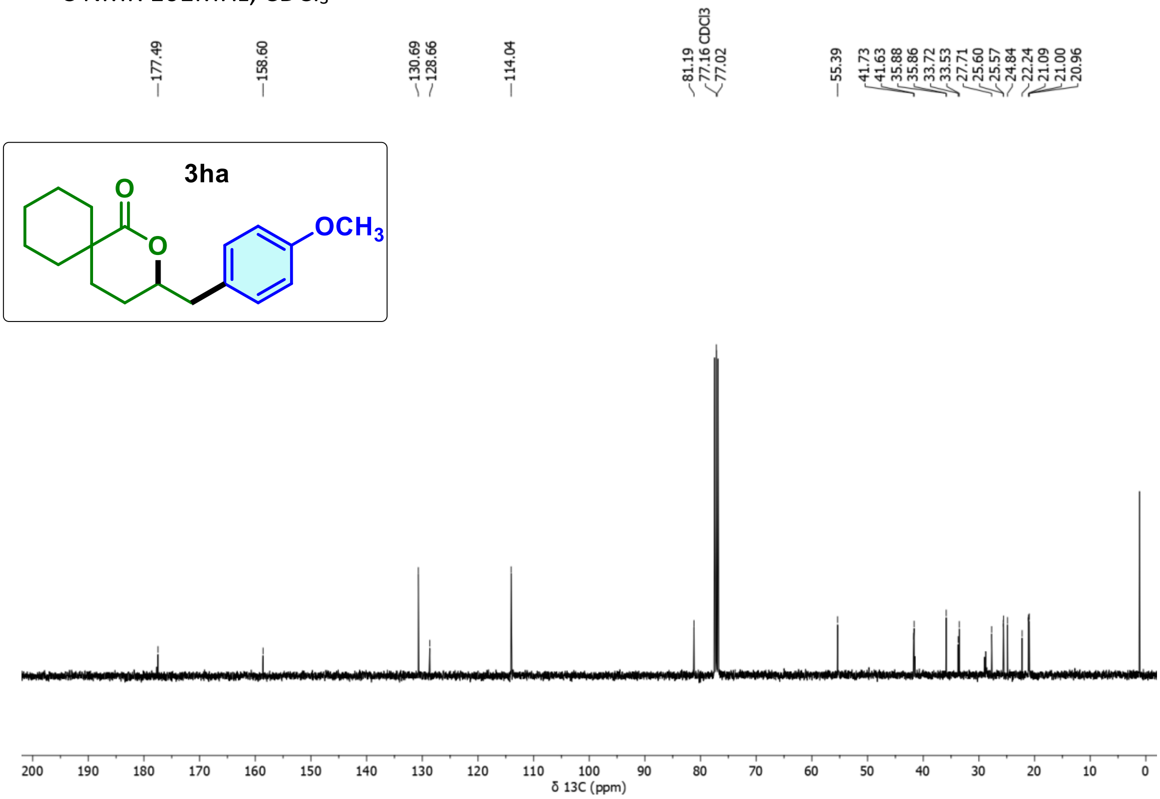

Table of contents

^{1}H NMR 300MHz, $CDCl_3$

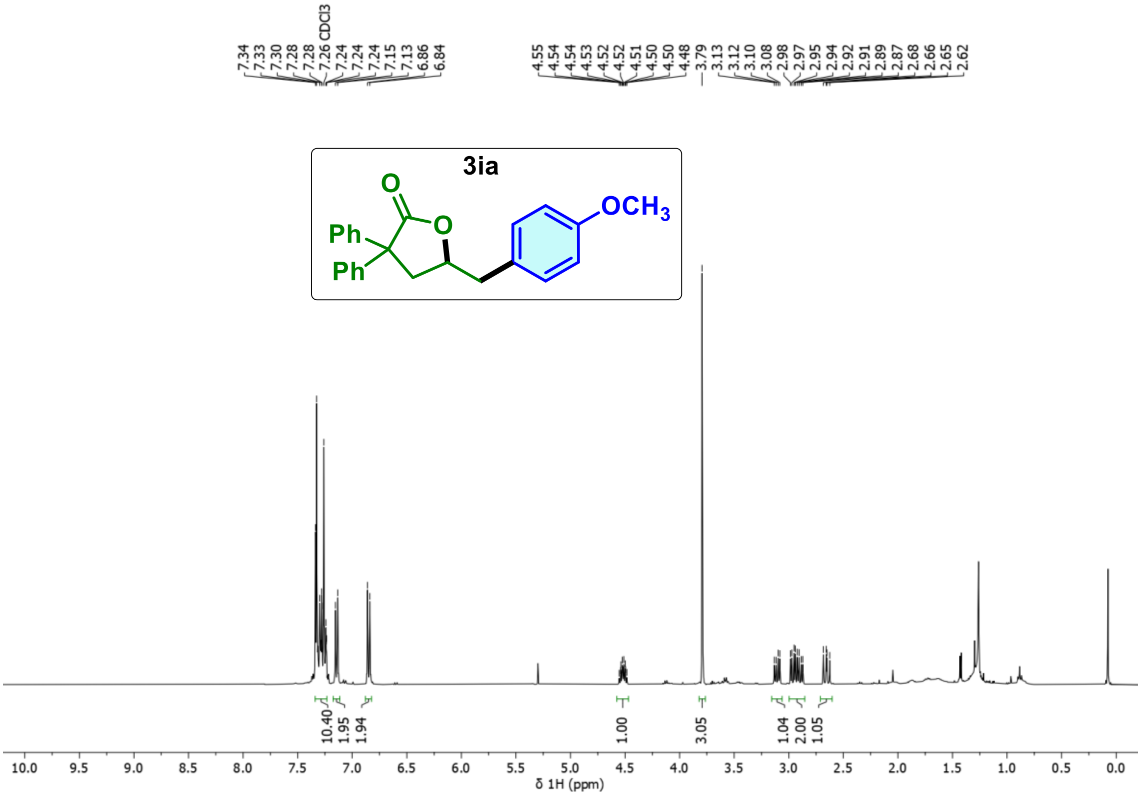

^{13}C NMR 101MHz, $CDCl_3$

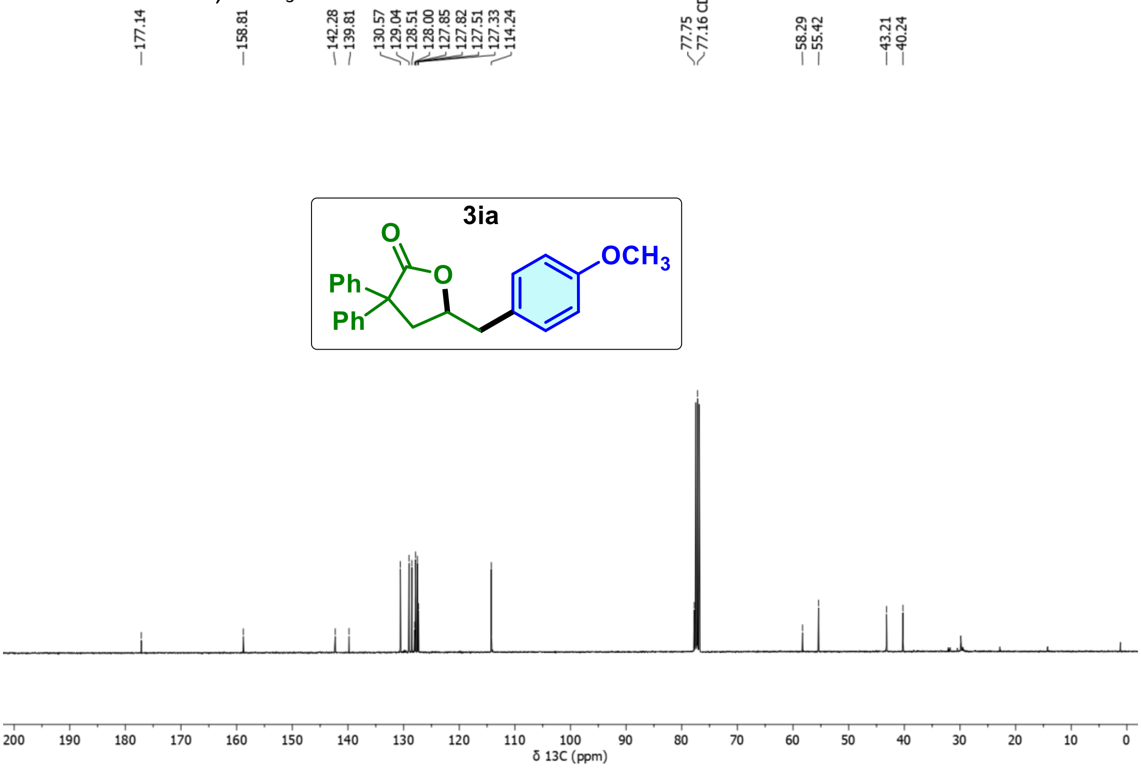

^{1}H NMR 300MHz, $CDCl_3$

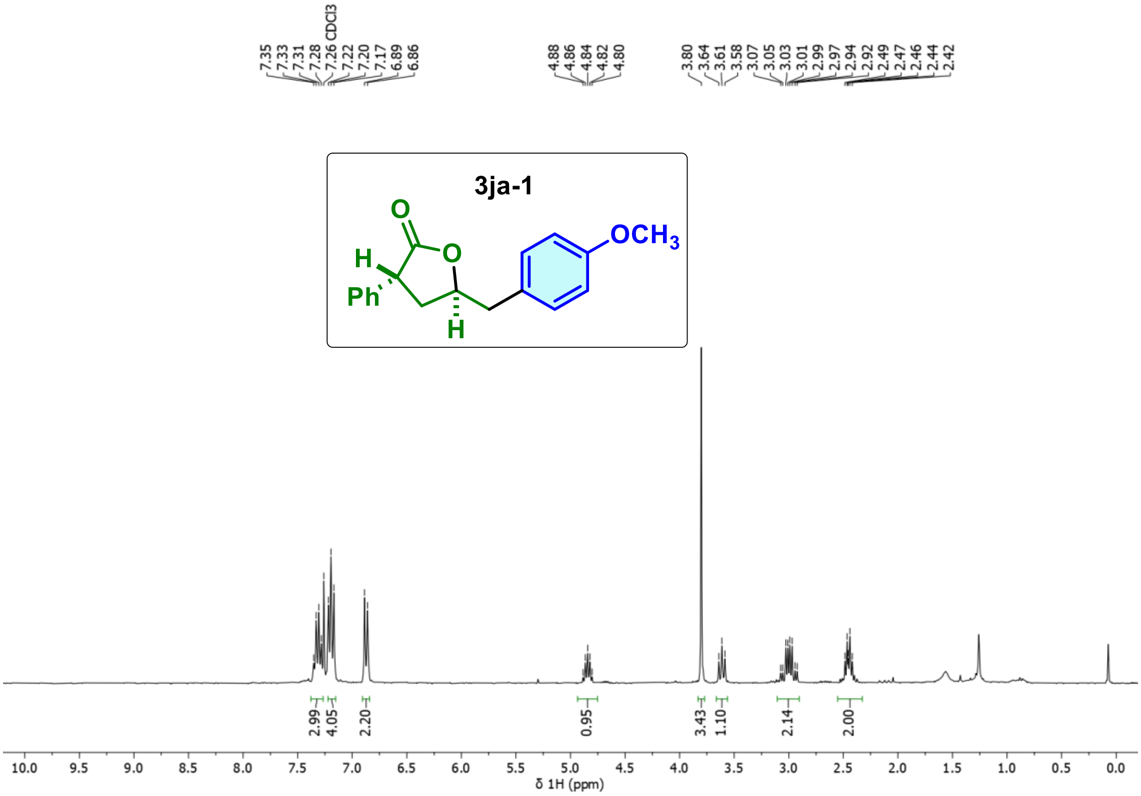

^{13}C NMR 101MHz, $CDCl_3$

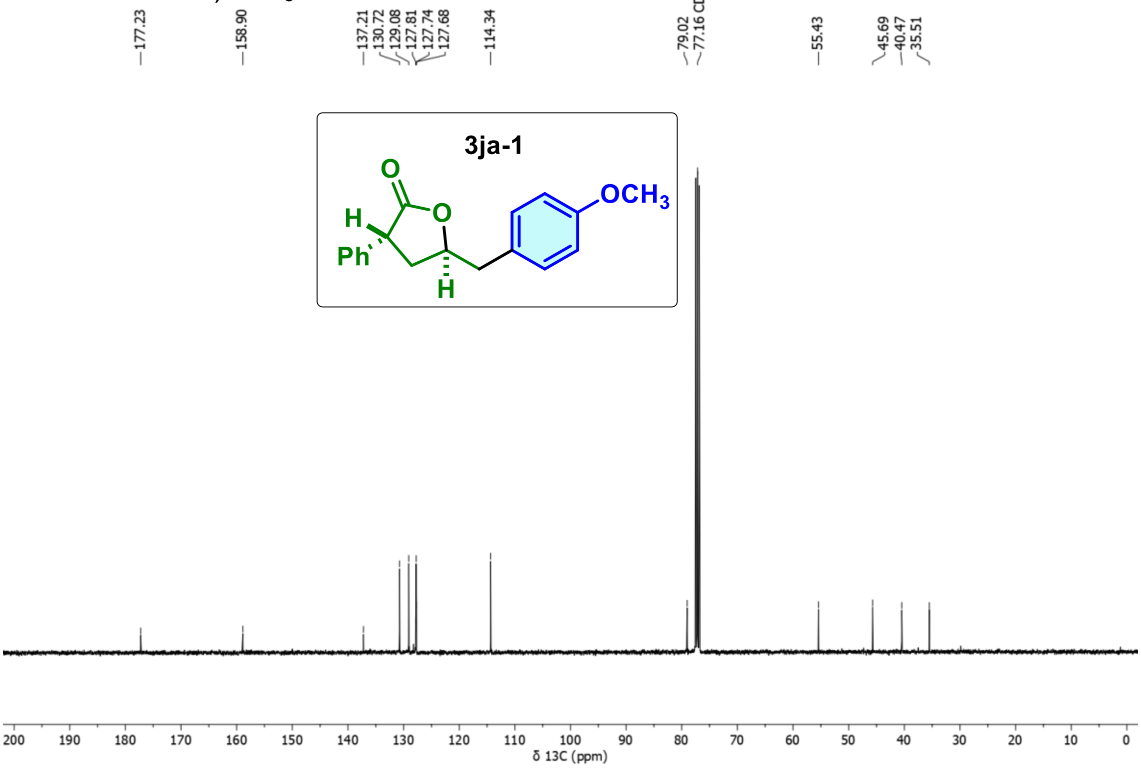

^{1}H NMR 300MHz, $CDCl_3$

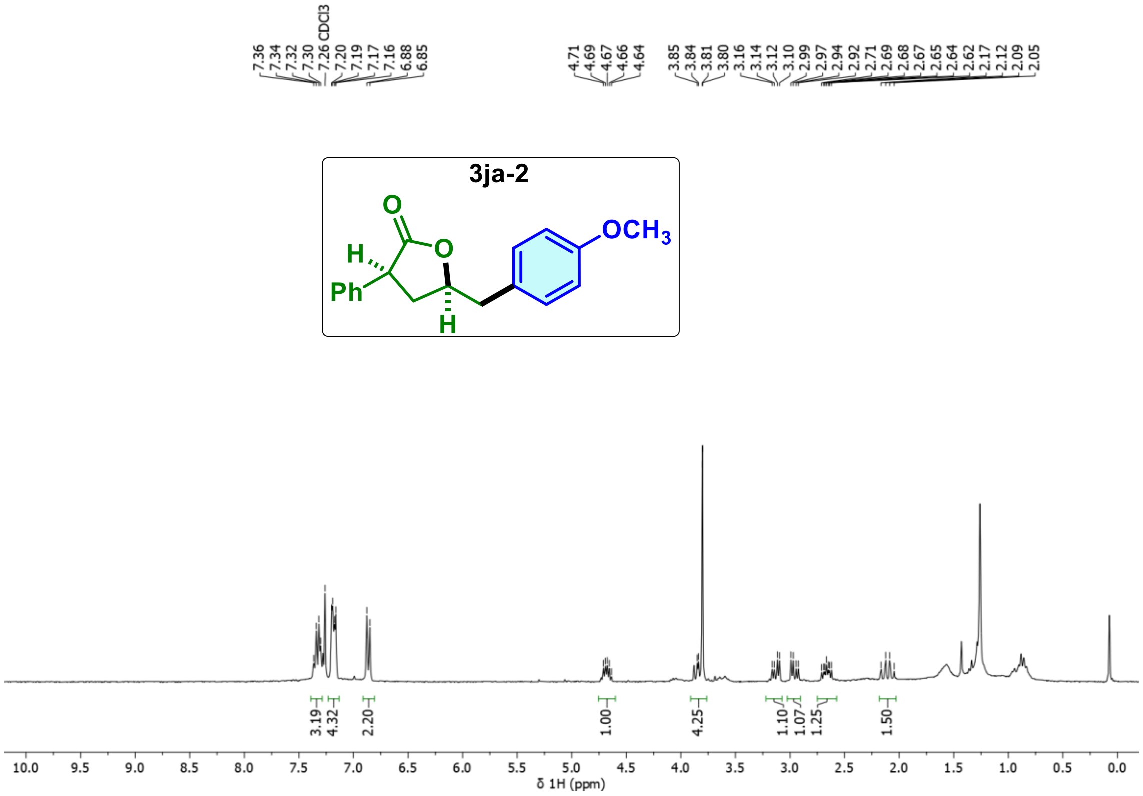

^{13}C NMR 101MHz, $CDCl_3$

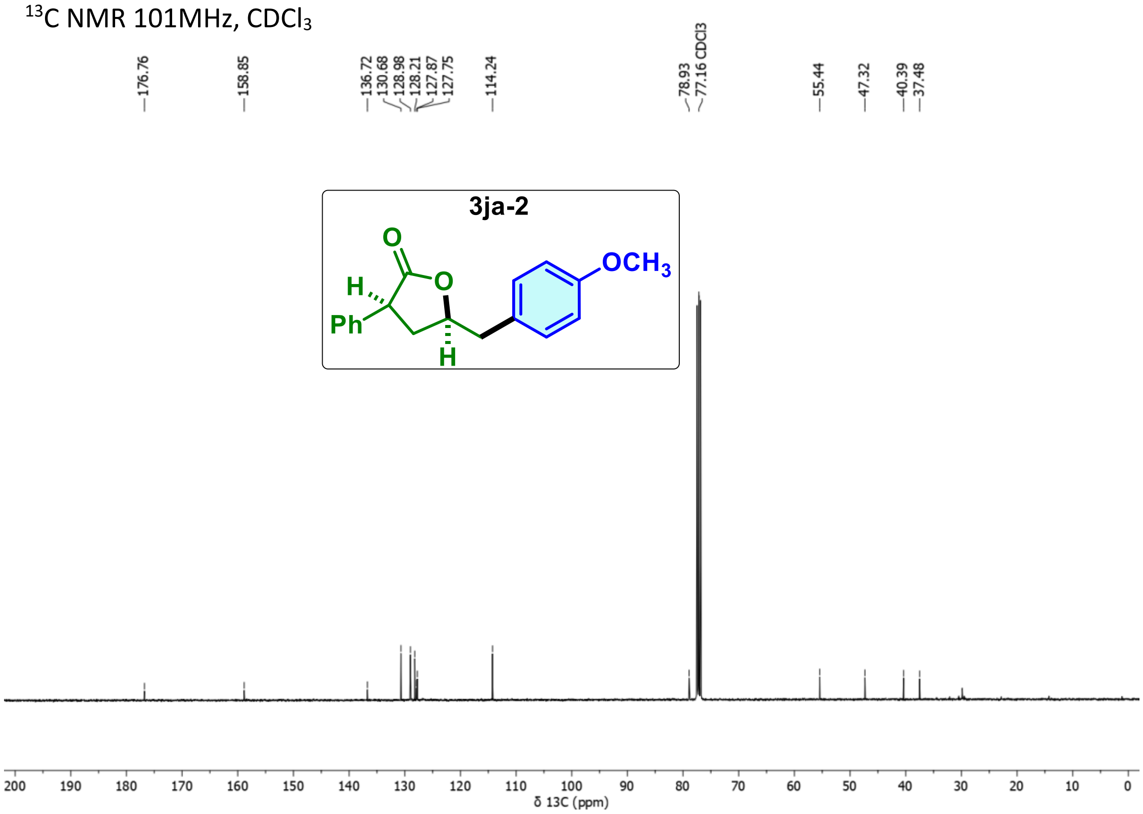

^{1}H NMR 300MHz, $CDCl_3$

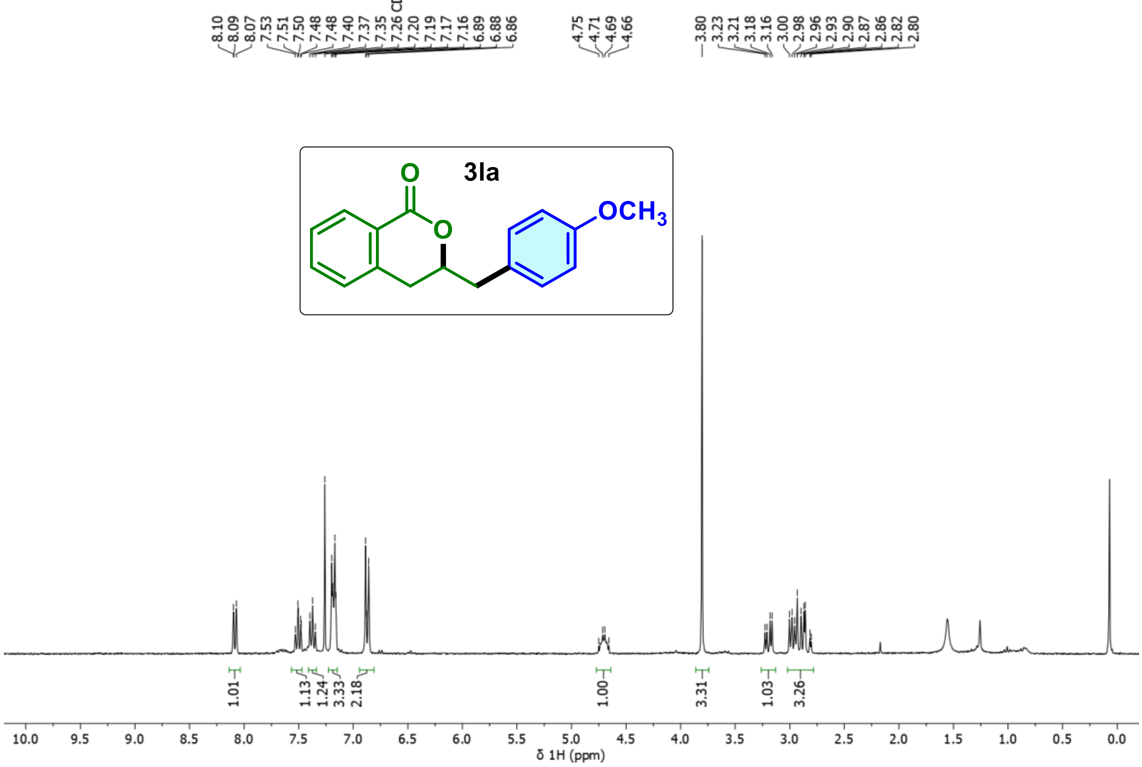

^{13}C NMR 101MHz, $CDCl_3$

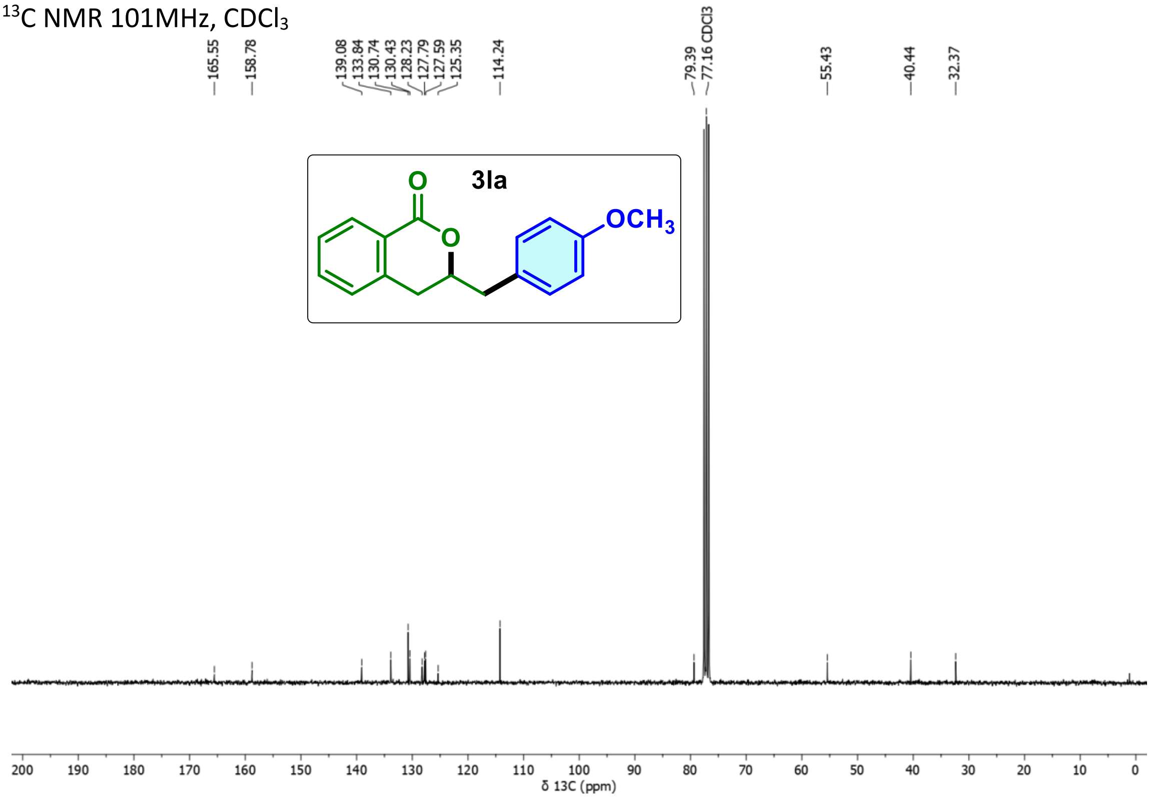

^{1}H NMR 300MHz, $CDCl_3$

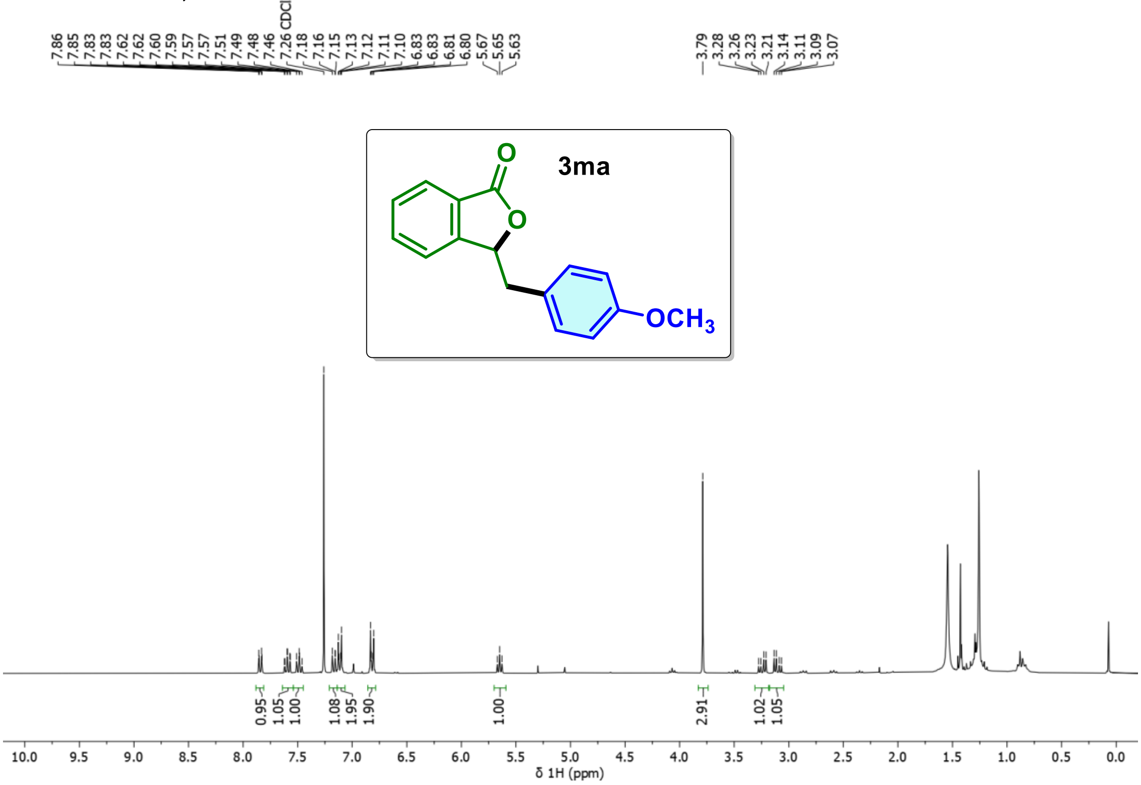

^{1}H NMR 300MHz, $CDCl_3$

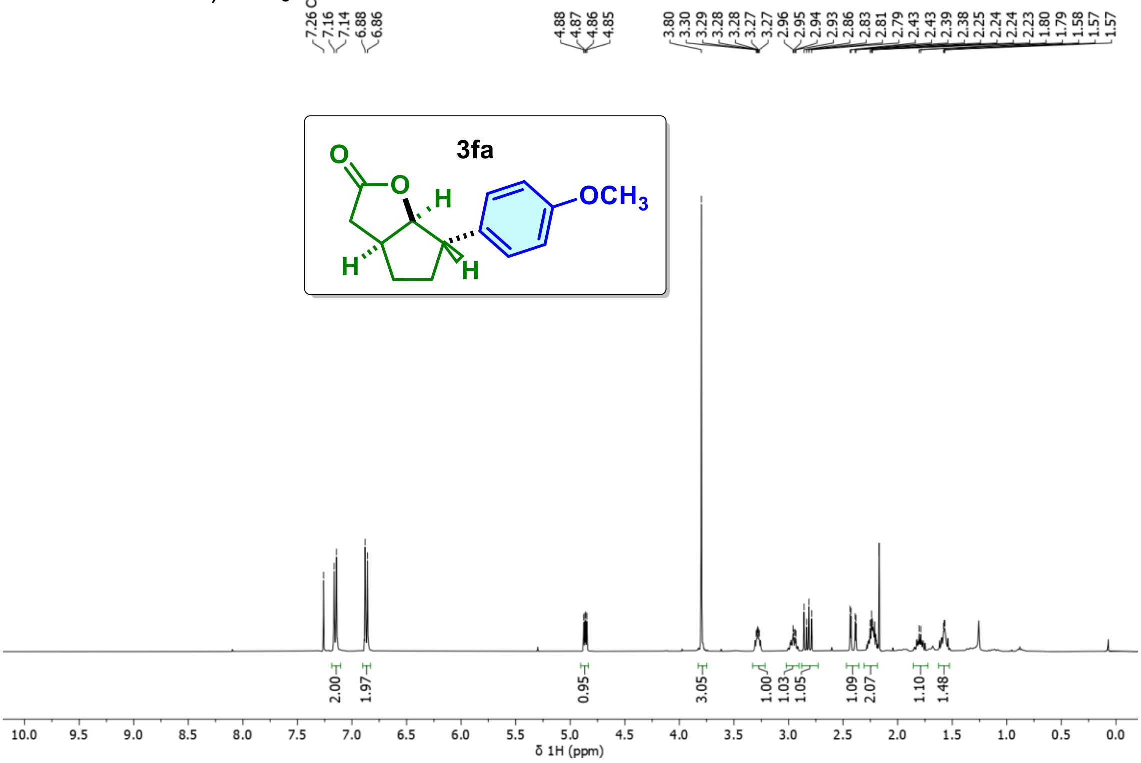

^{13}C NMR 101MHz, $CDCl_3$

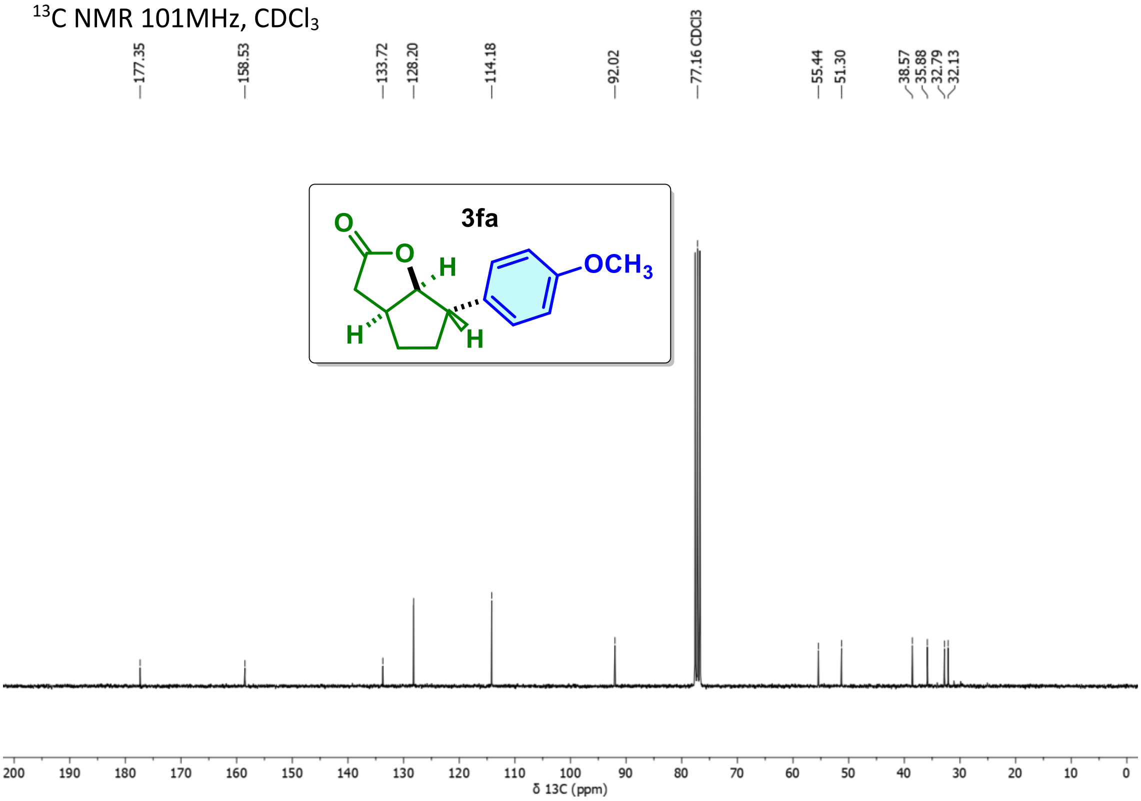

^{1}H NMR 300MHz, $CDCl_3$

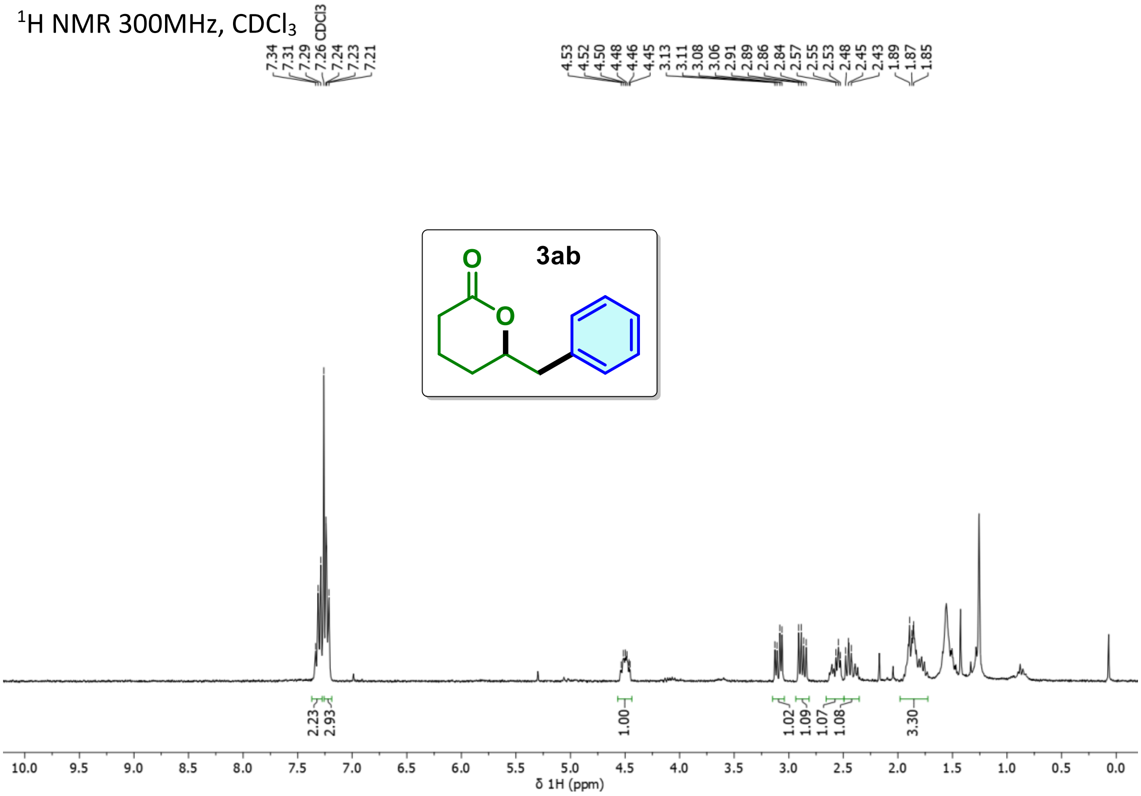

^{13}C NMR 101MHz, $CDCl_3$

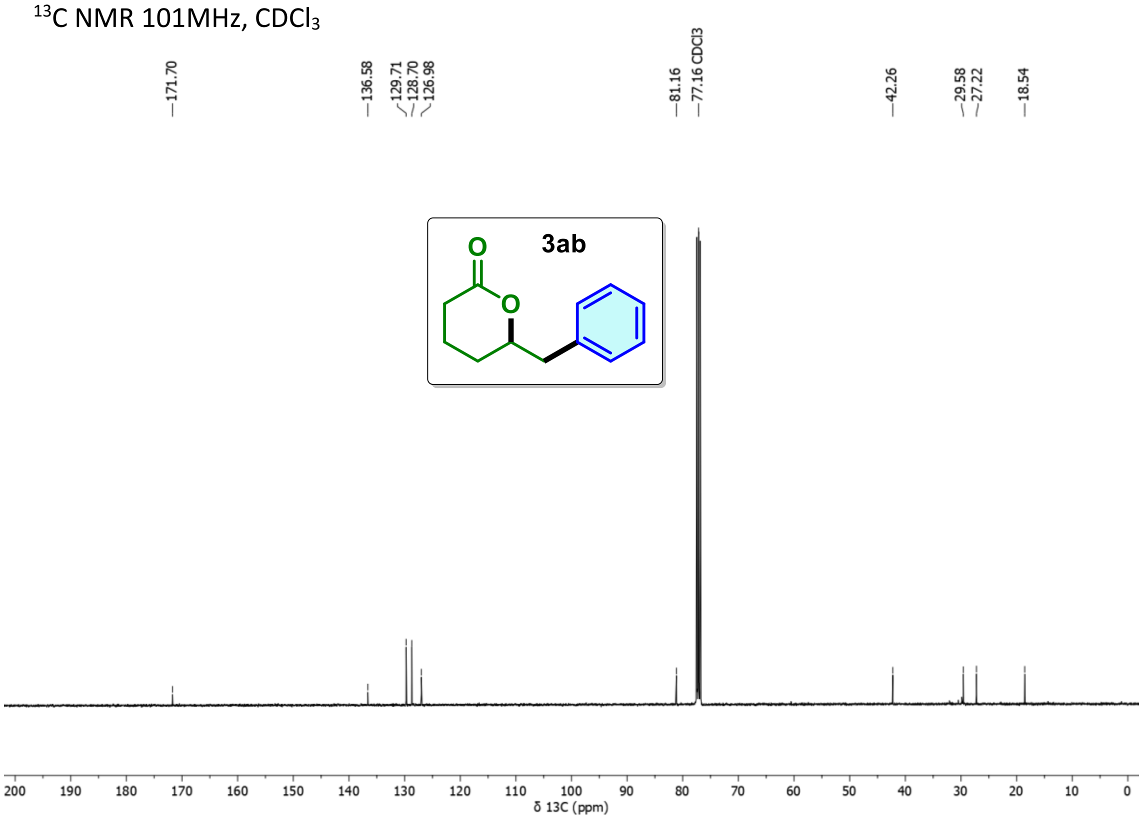

^{1}H NMR 300MHz, $CDCl_3$

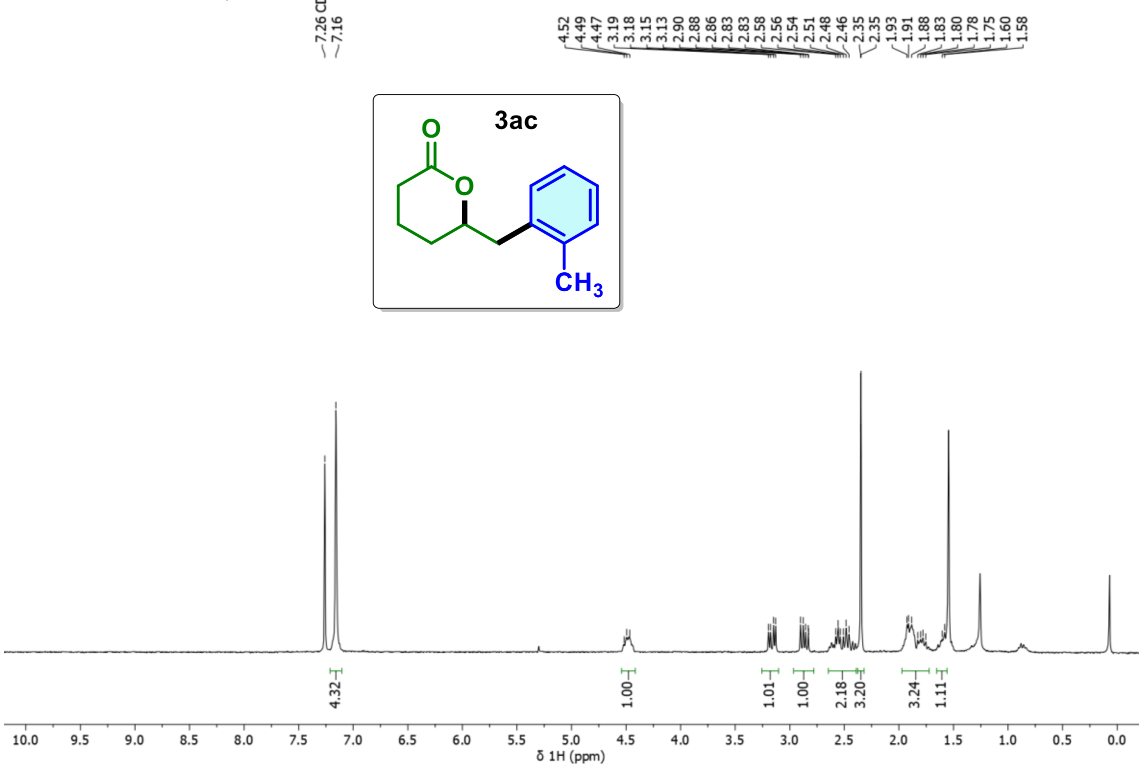

^{13}C NMR 101MHz, $CDCl_3$

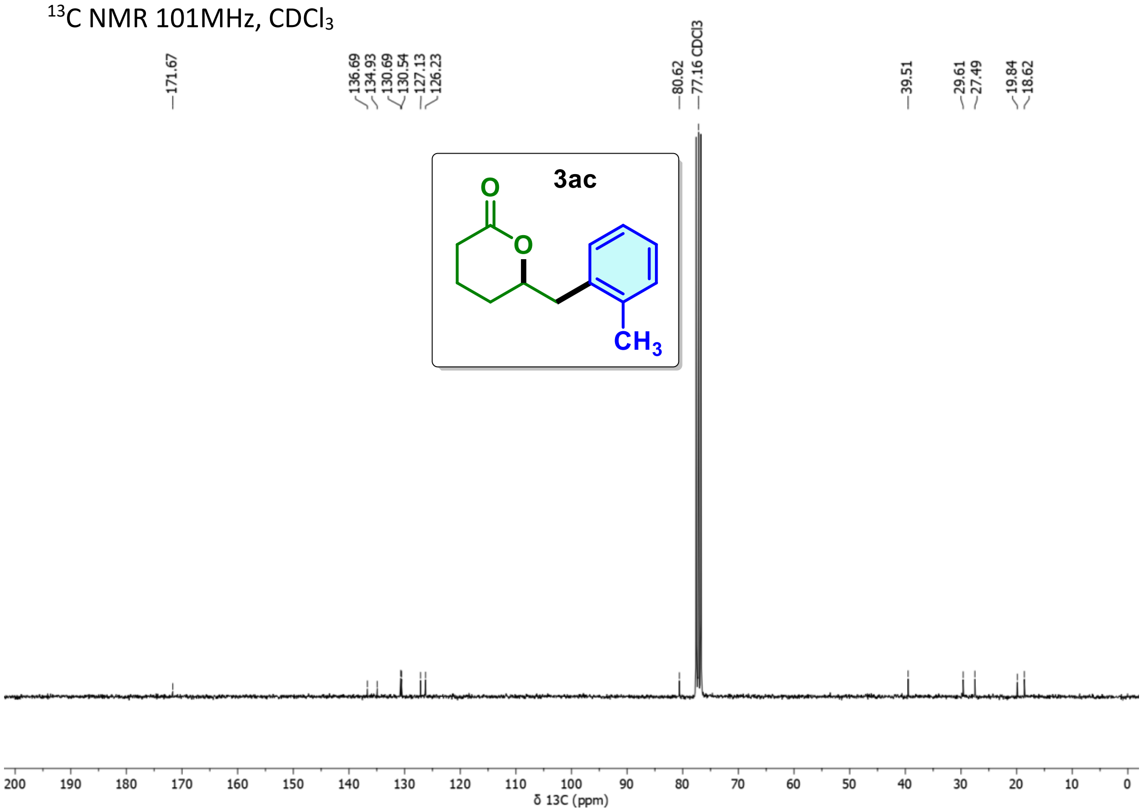

^1H NMR 300MHz, $CDCl_3$

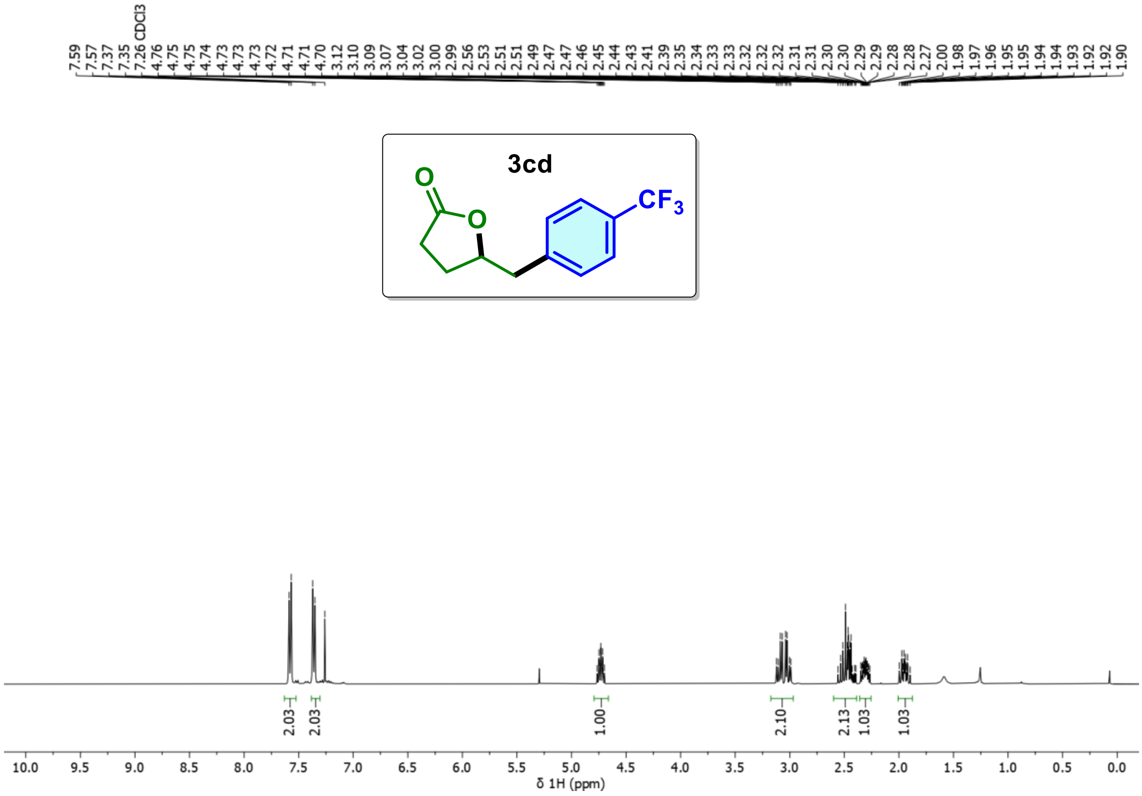

^{19}F NMR 376MHz, $CDCl_3$

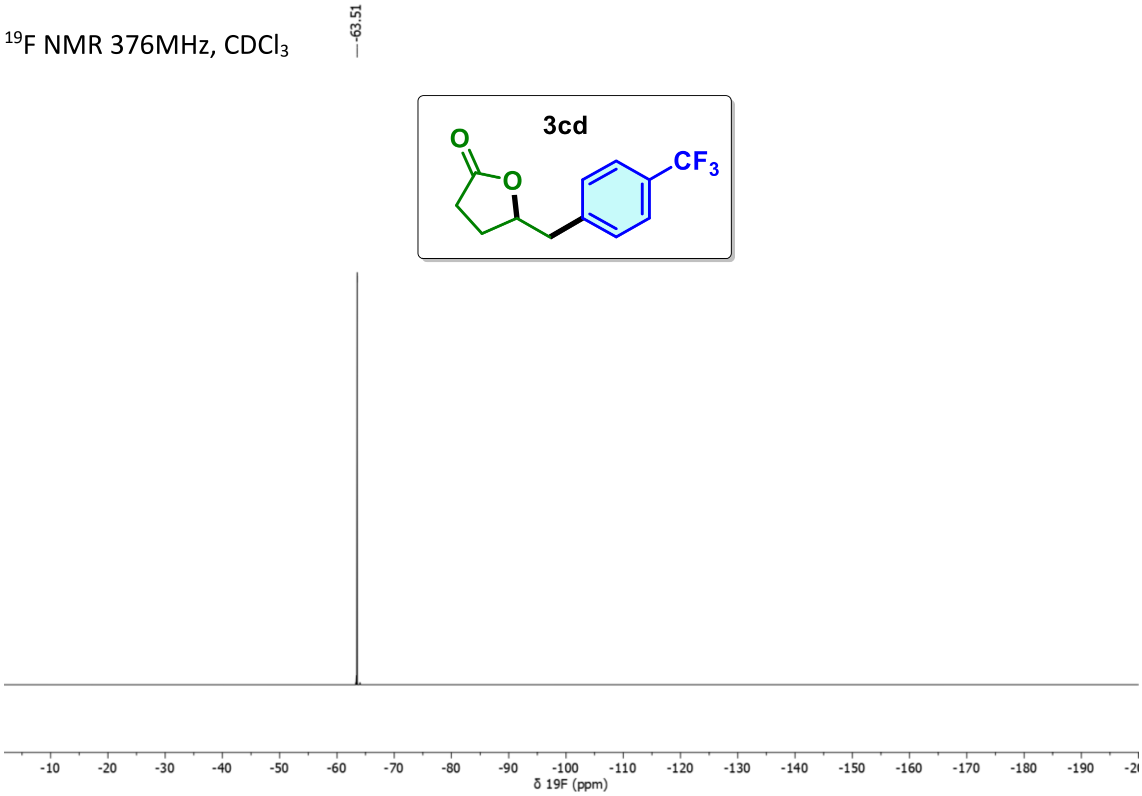

^{13}C NMR 101MHz, $CDCl_3$

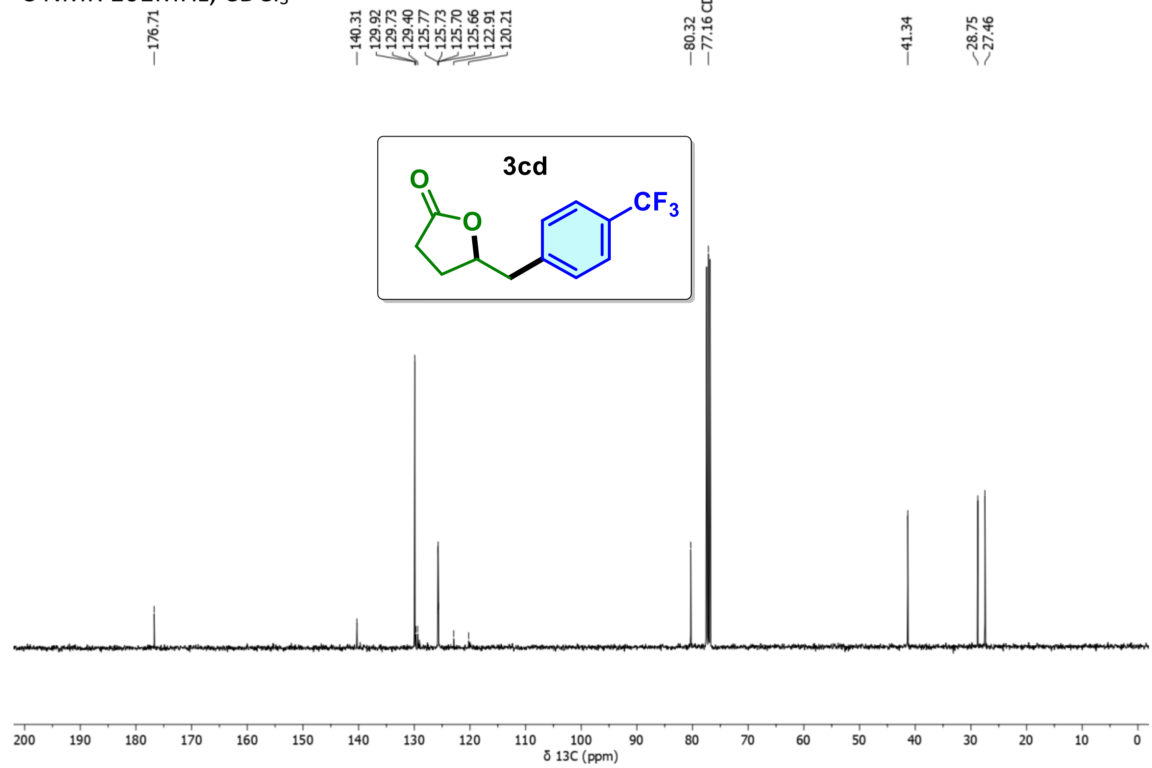

Table of contents

^{1}H NMR 300MHz, $CDCl_3$

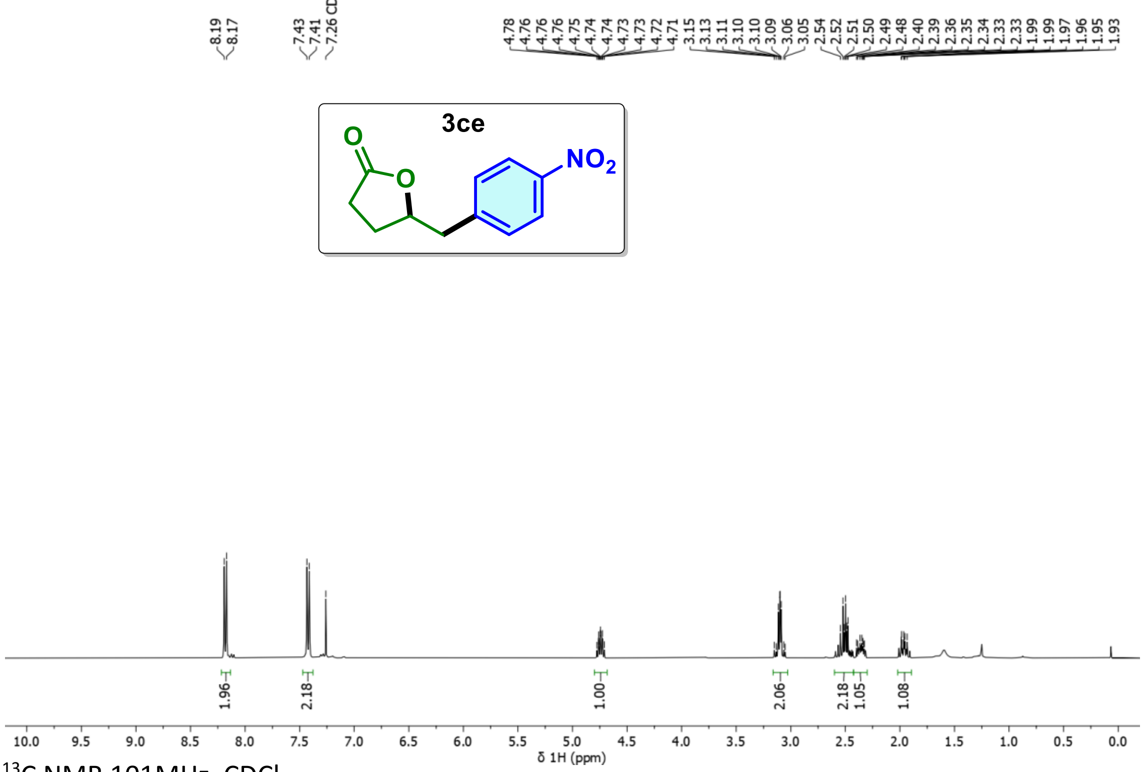

^{13}C NMR 101MHz, $CDCl_3$

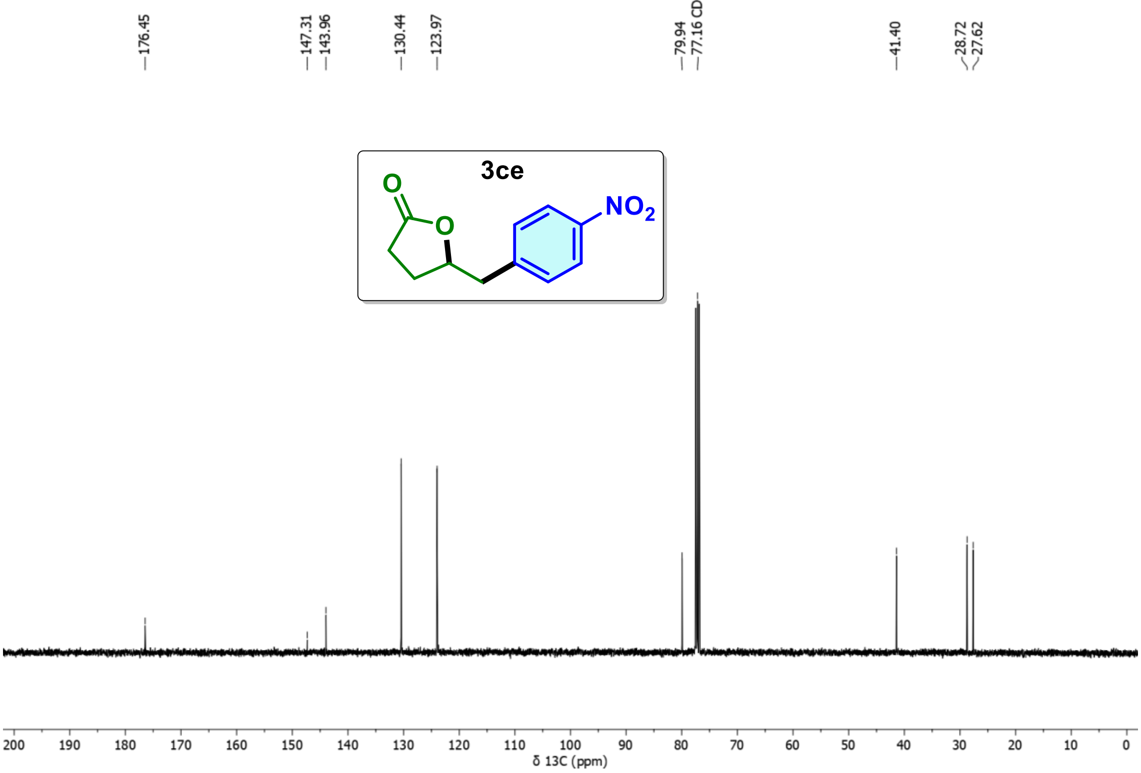

^{1}H NMR 300MHz, $CDCl_3$

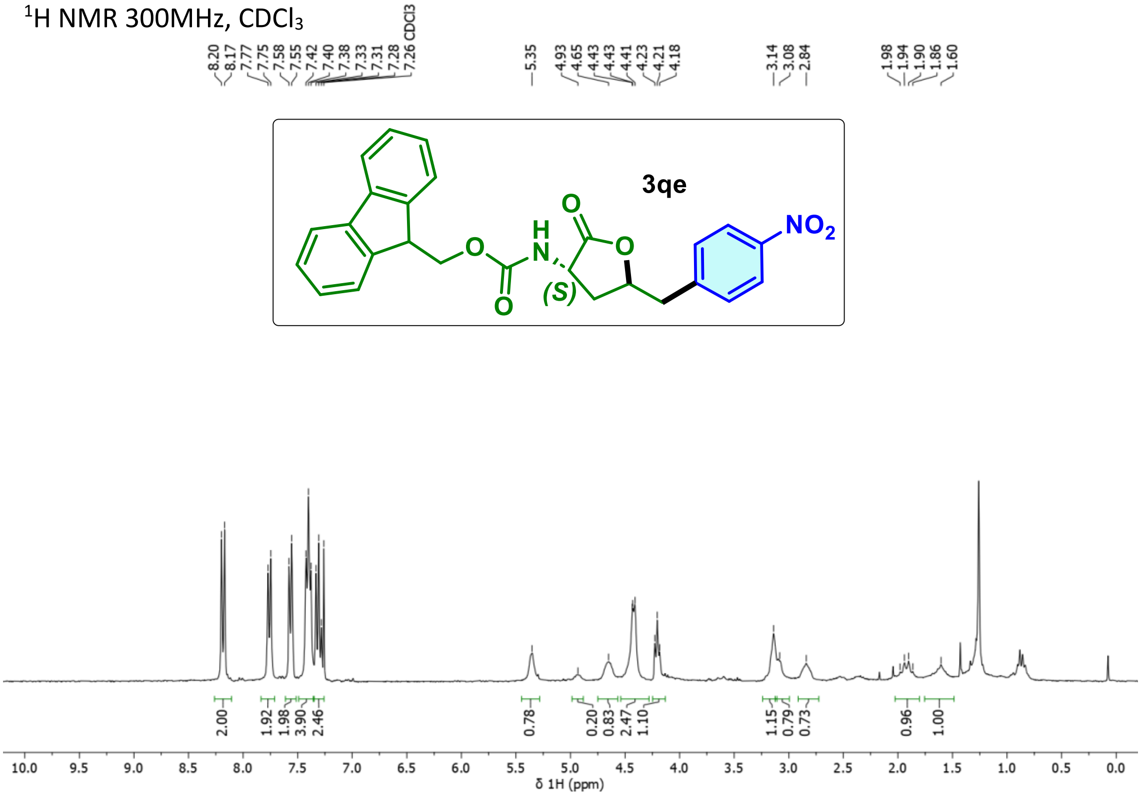

^{13}C NMR 101MHz, $CDCl_3$

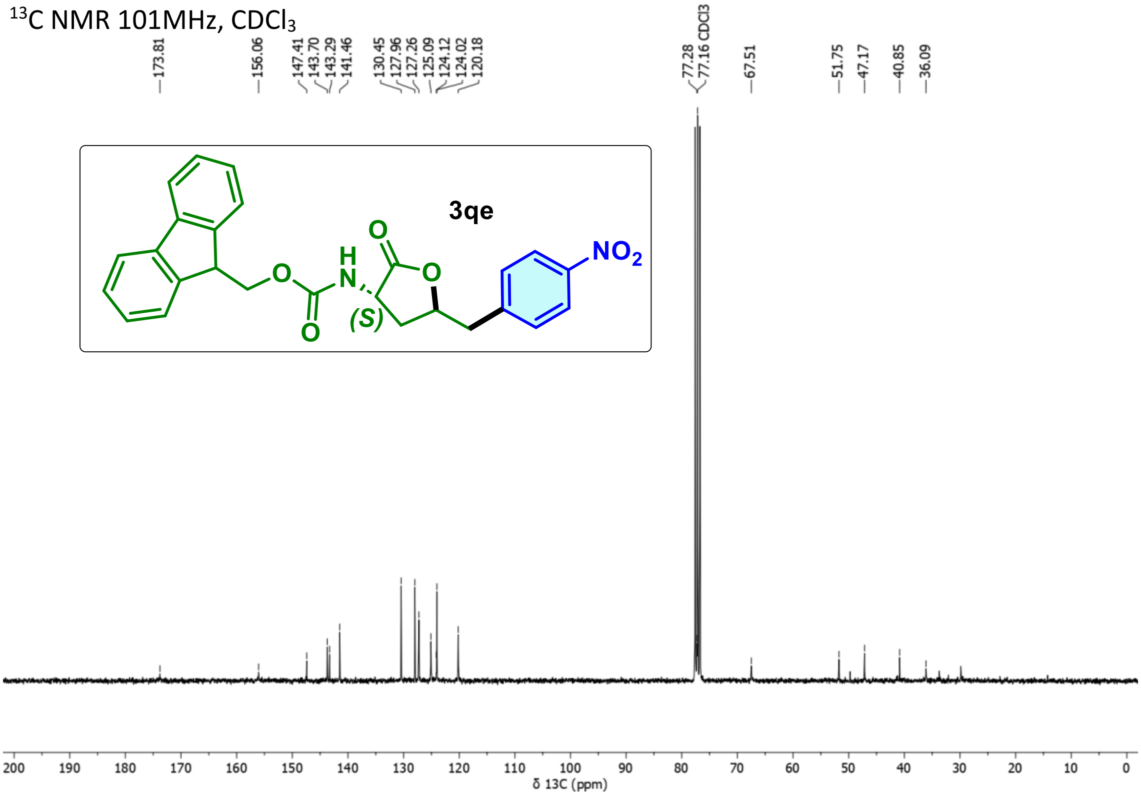

^{1}H NMR 300MHz, $CDCl_3$

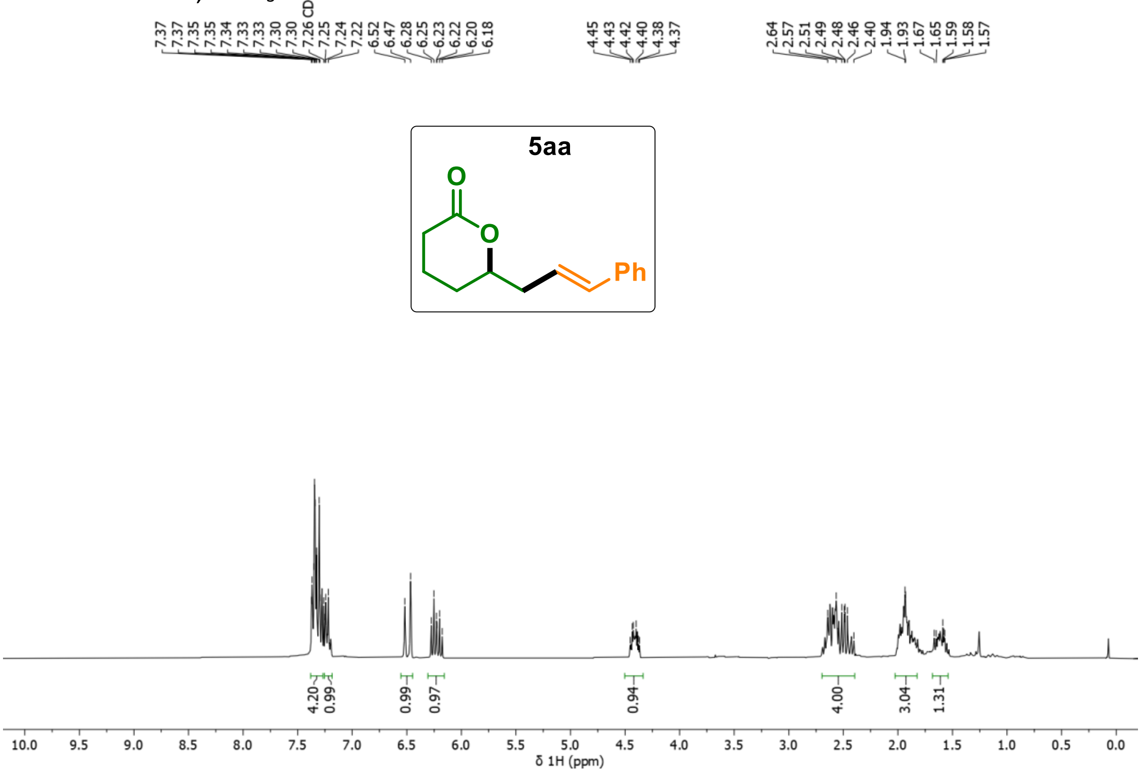

^{13}C NMR 101MHz, $CDCl_3$

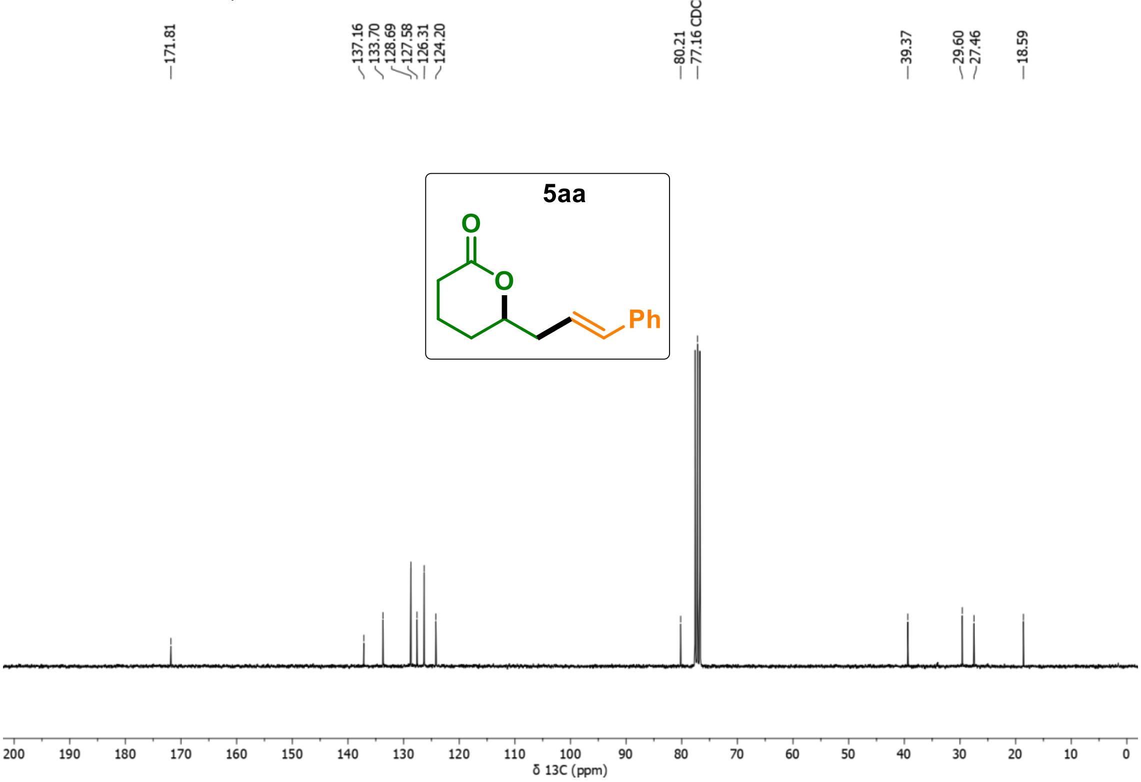

^{1}H NMR 300MHz, CD_2Cl_2

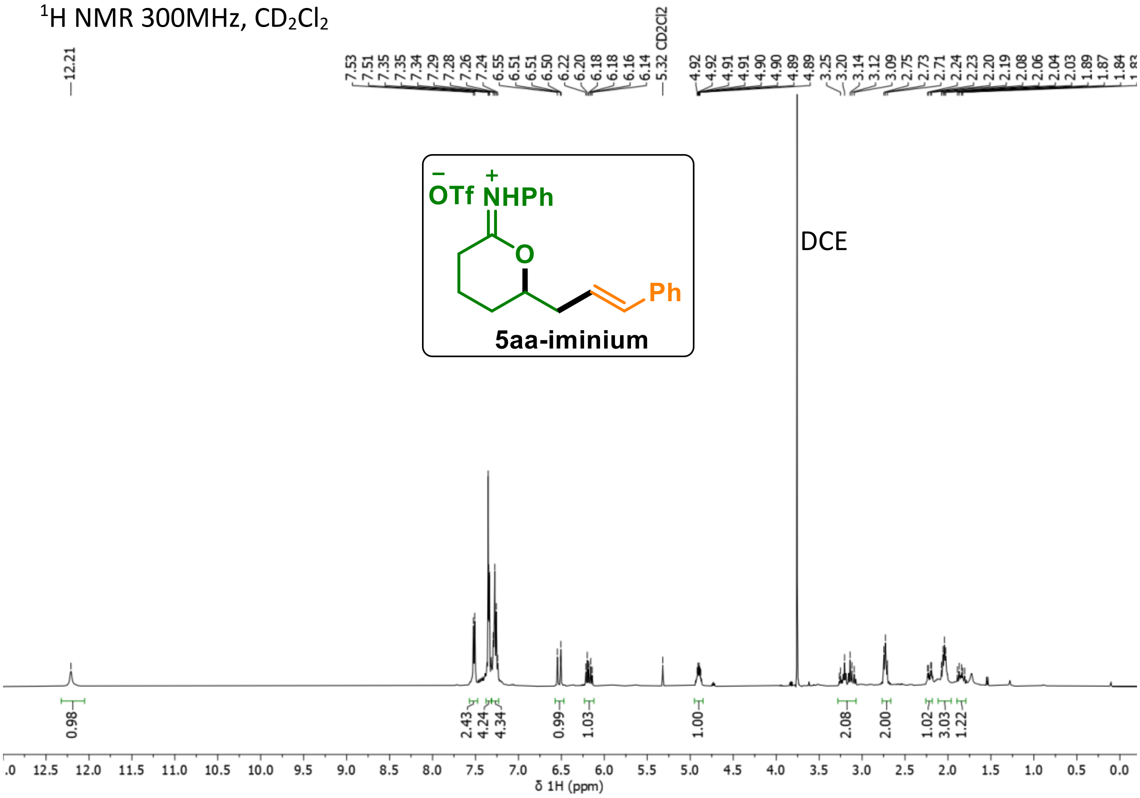

^{13}C NMR 101MHz, CD_2Cl_2

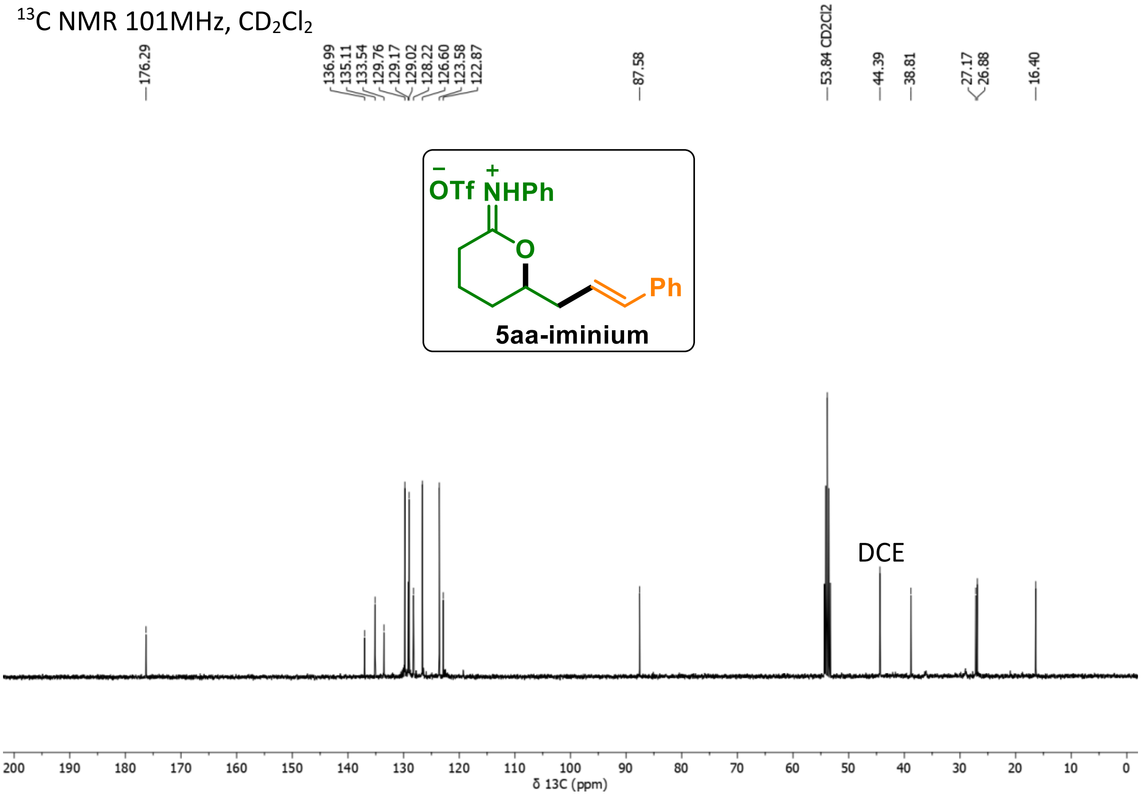

^{19}F NMR 282MHz, CD_2Cl_2

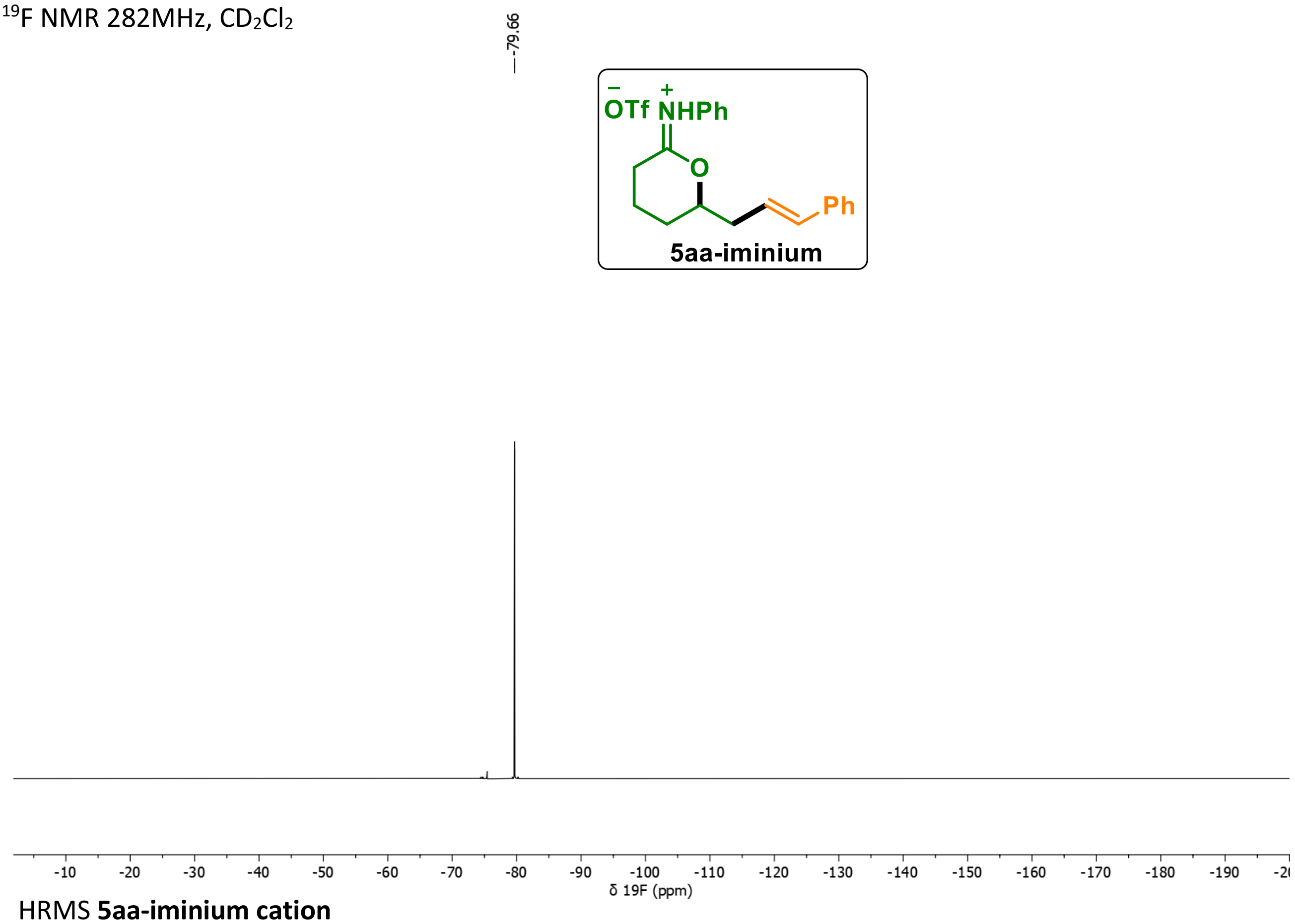

HRMS **5aa-iminium cation**

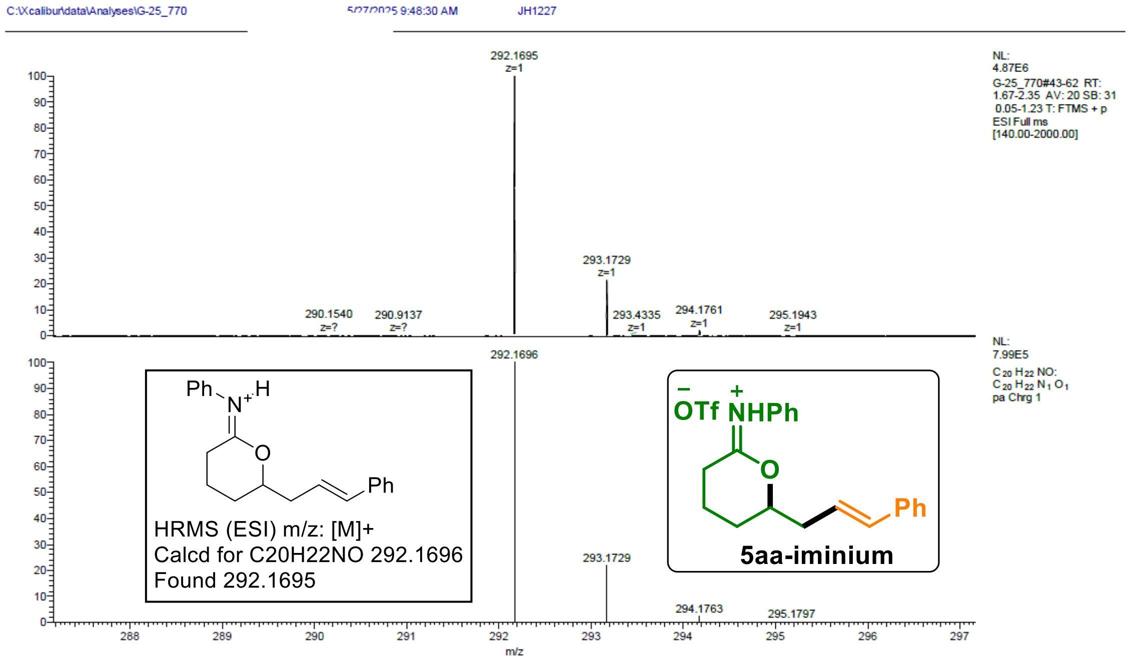

Table of contents

^{1}H NMR 300MHz, $CDCl_3$

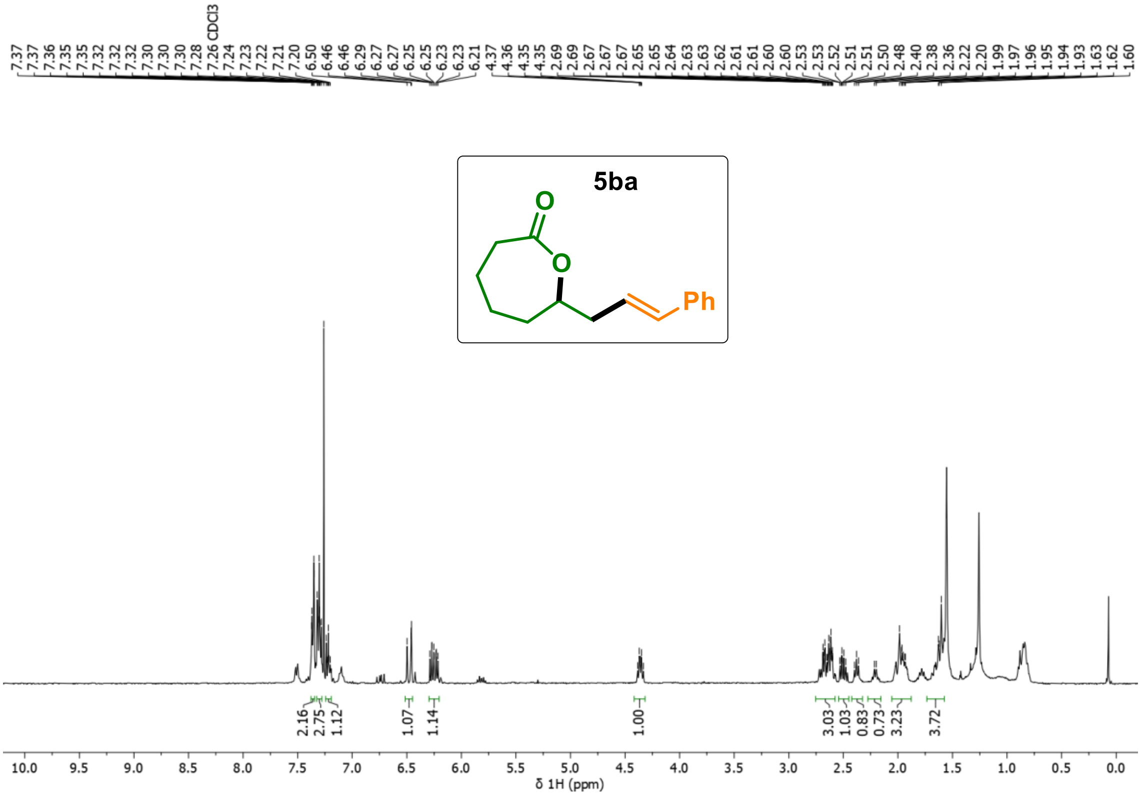

^{13}C NMR 101MHz, $CDCl_3$

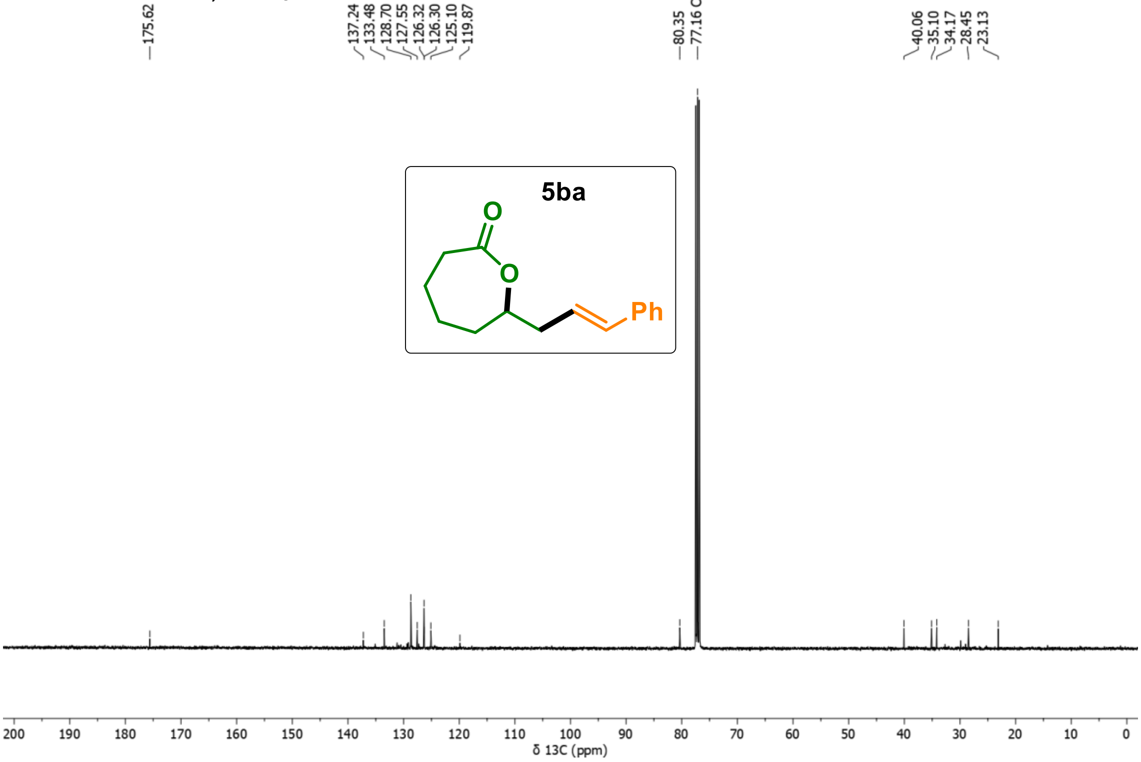

Table of contents

^{1}H NMR 300MHz, $CDCl_3$

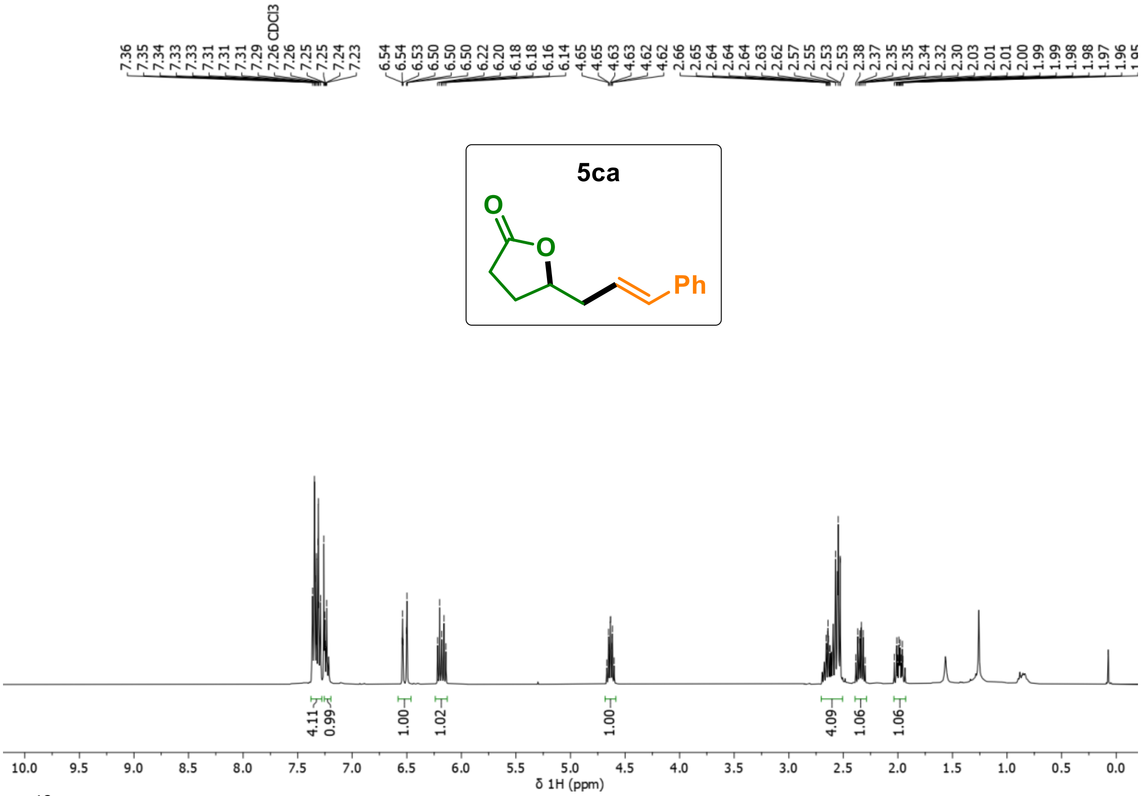

^{13}C NMR 101MHz, $CDCl_3$

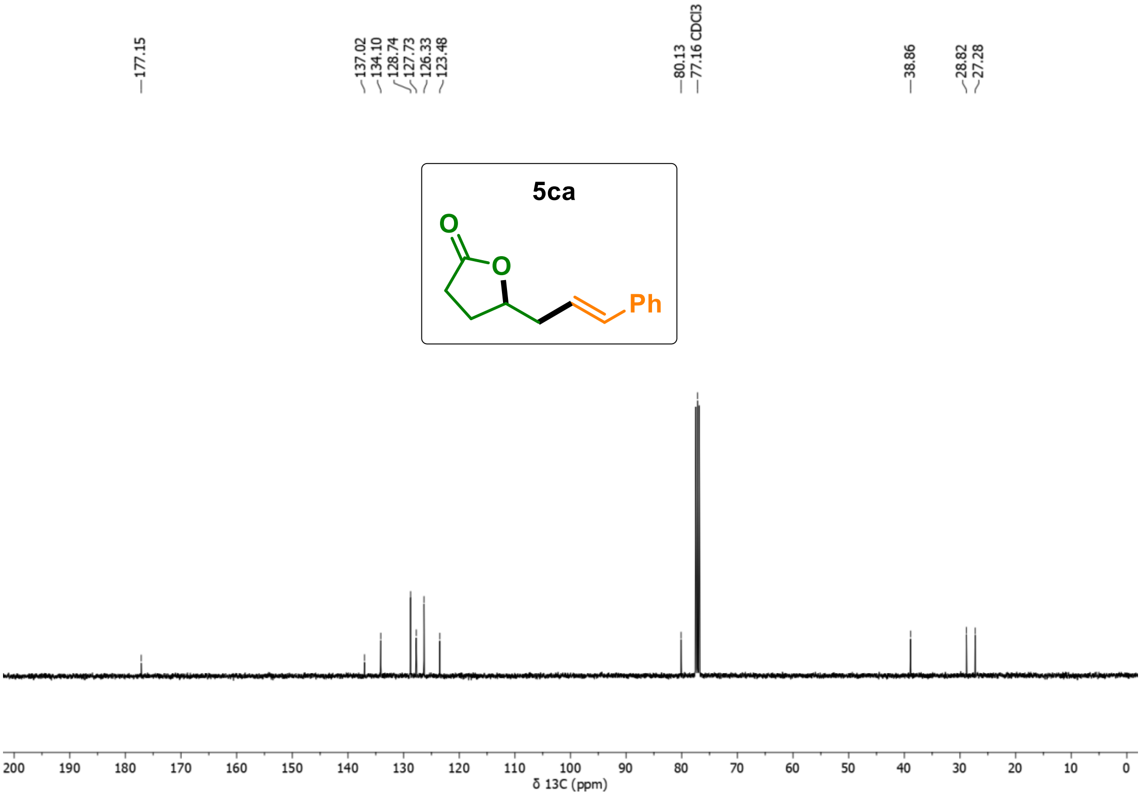

^{1}H NMR 300MHz, $CDCl_3$

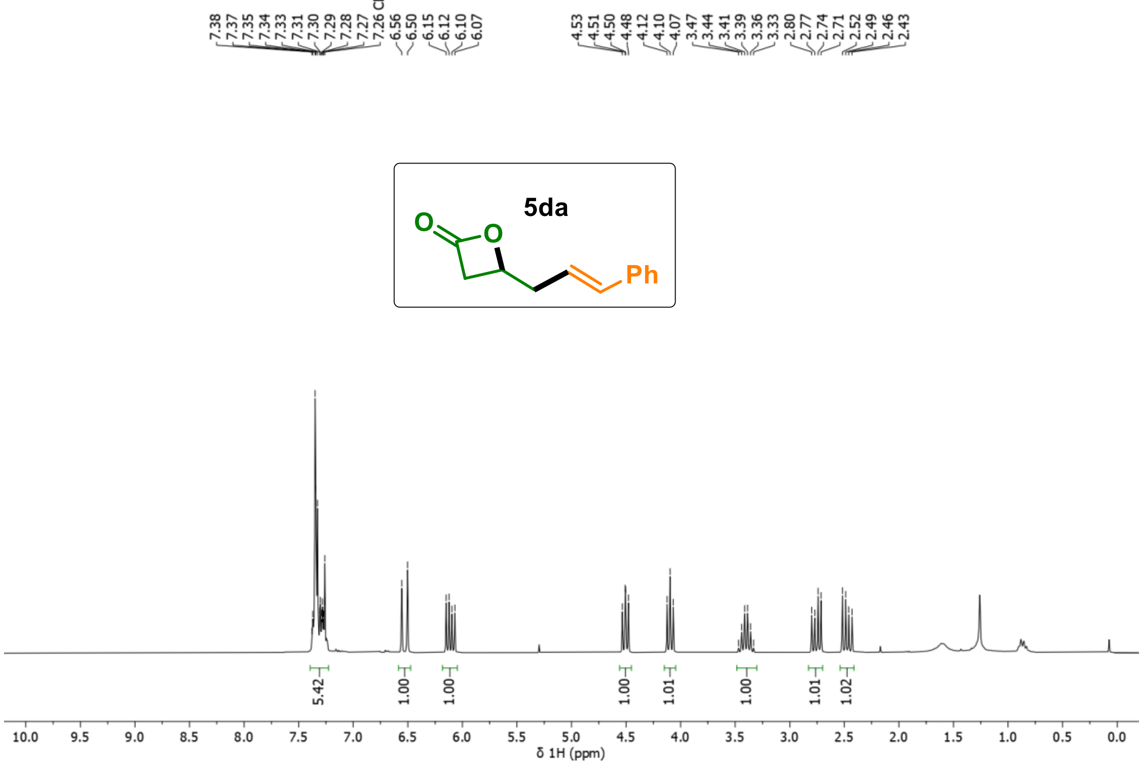

^{13}C NMR 101MHz, $CDCl_3$

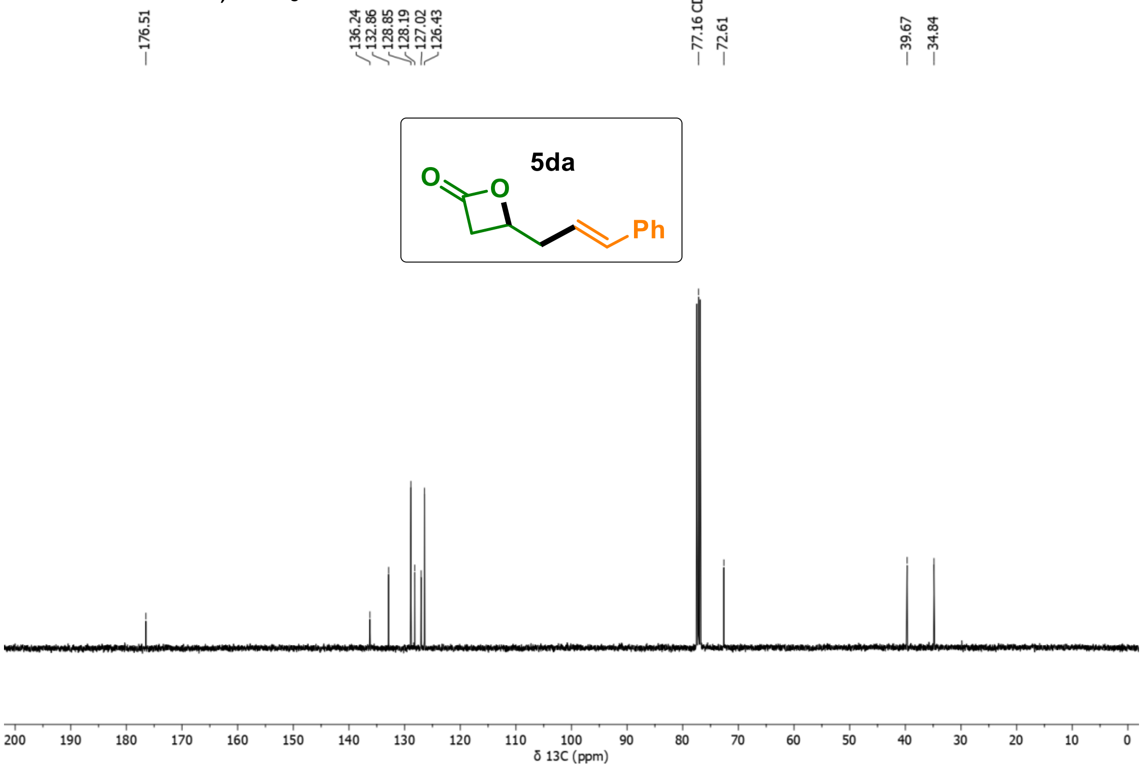

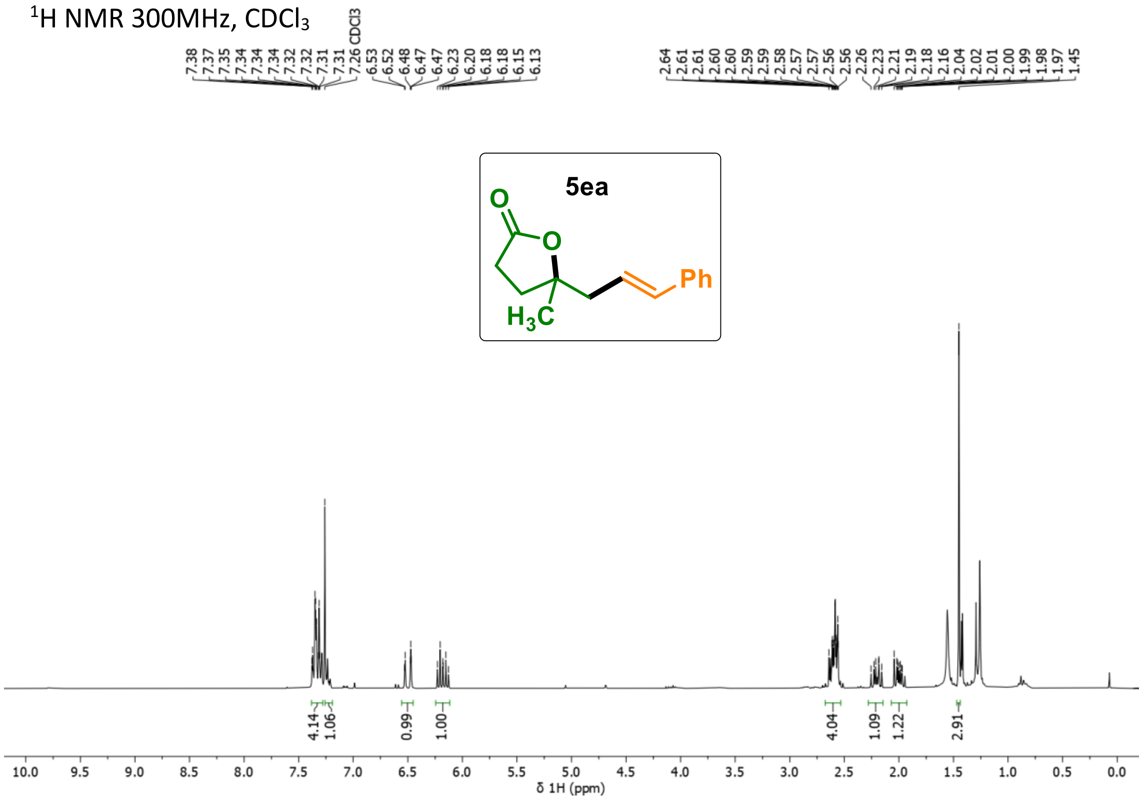
^{1}H NMR 300MHz, CDCl$_3$
7.38
7.37
7.35
7.34
7.34
7.34
7.32
7.32
7.31
7.31
7.26 CDCl3
6.53
6.52
6.48
6.47
6.47
6.23
6.20
6.18
6.18
6.15
6.13
2.64
2.61
2.61
2.60
2.60
2.59
2.59
2.58
2.57
2.57
2.56
2.56
2.26
2.23
2.21
2.19
2.18
2.16
2.04
2.02
2.01
2.00
1.99
1.98
1.97
1.45
5ea
O
O
Ph
H3C
4.14
1.06
0.99
1.00
4.04
1.09
1.22
2.91
δ 1H (ppm)

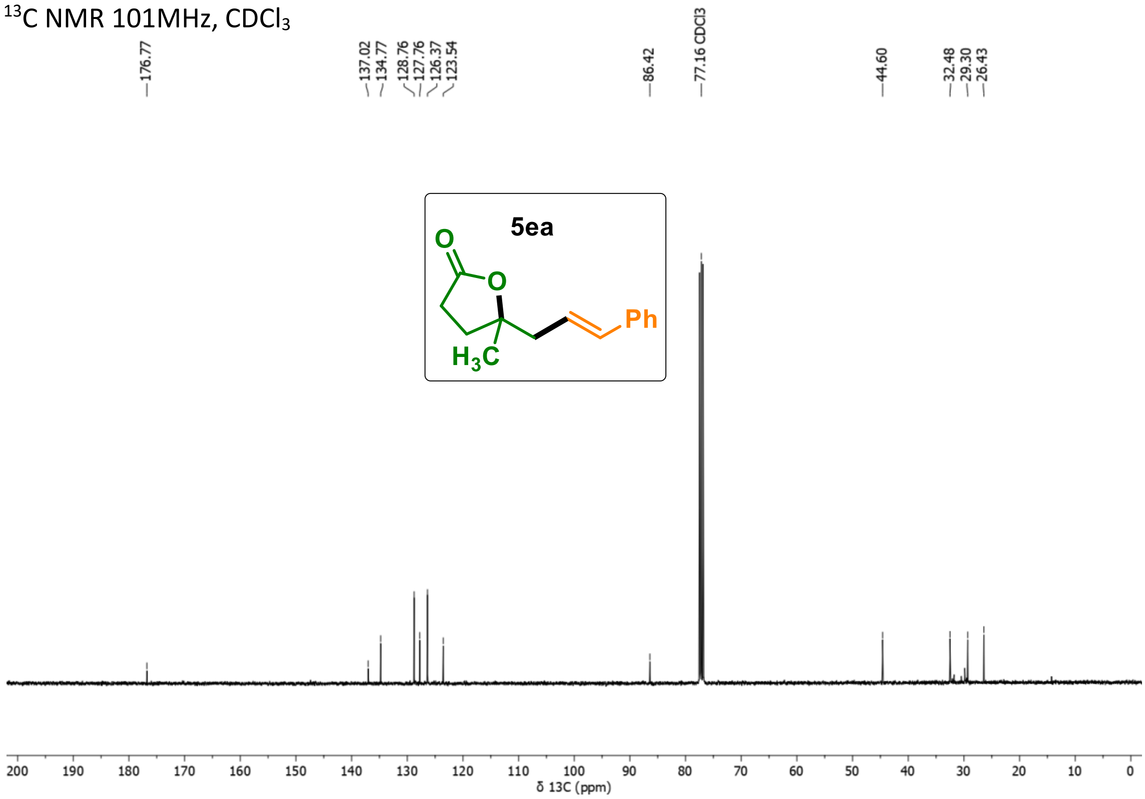
^{13}C NMR 101MHz, CDCl$_3$
176.77
137.02
134.77
128.76
127.76
126.37
123.54
86.42
77.16 CDCl3
44.60
32.48
29.30
26.43
5ea
O
O
Ph
H3C
δ 13C (ppm)

^{1}H NMR 300MHz, $CDCl_3$

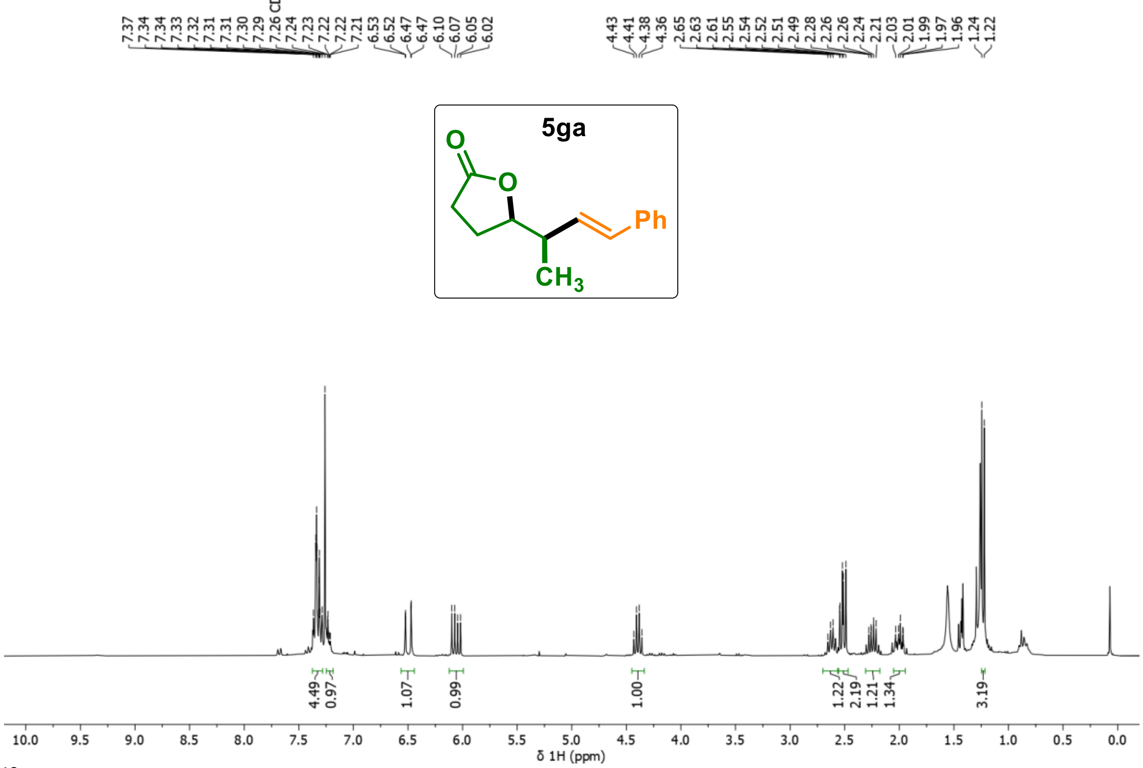

^{13}C NMR 101MHz, $CDCl_3$

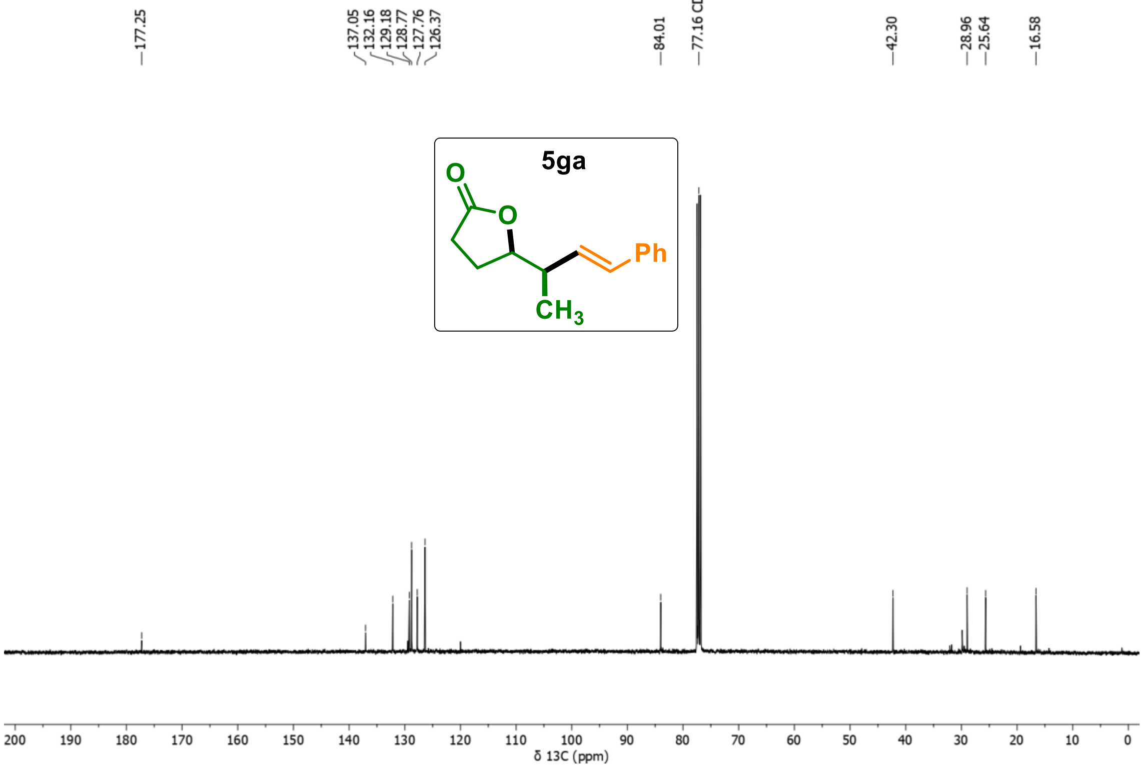

^{1}H NMR 300MHz, $CDCl_3$

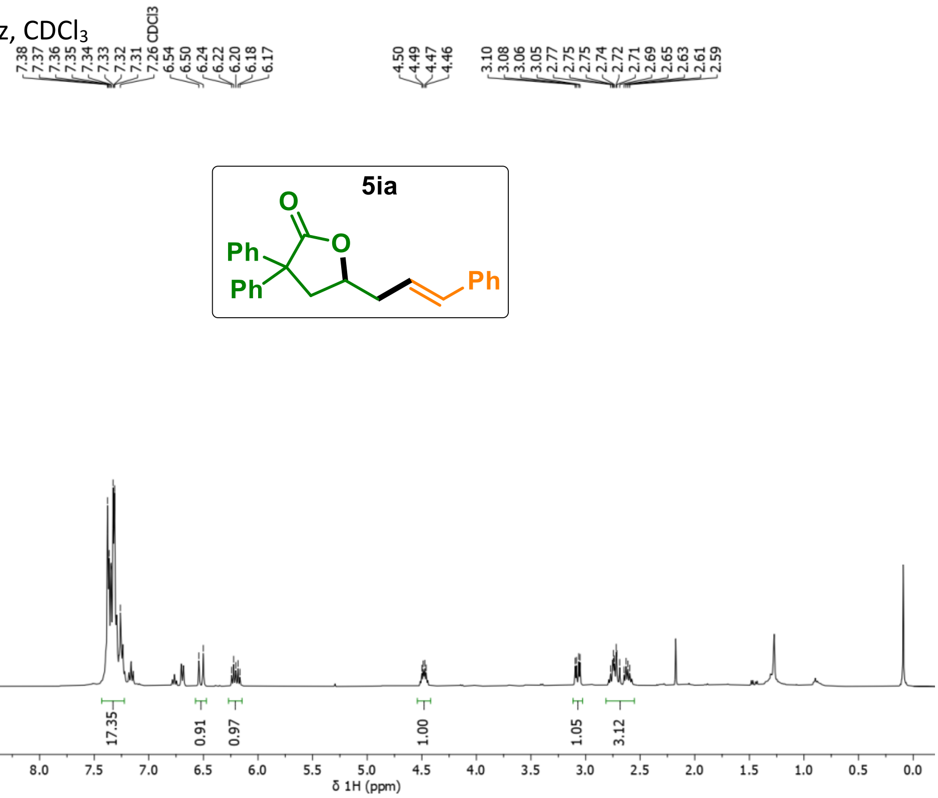

^{13}C NMR 101MHz, $CDCl_3$

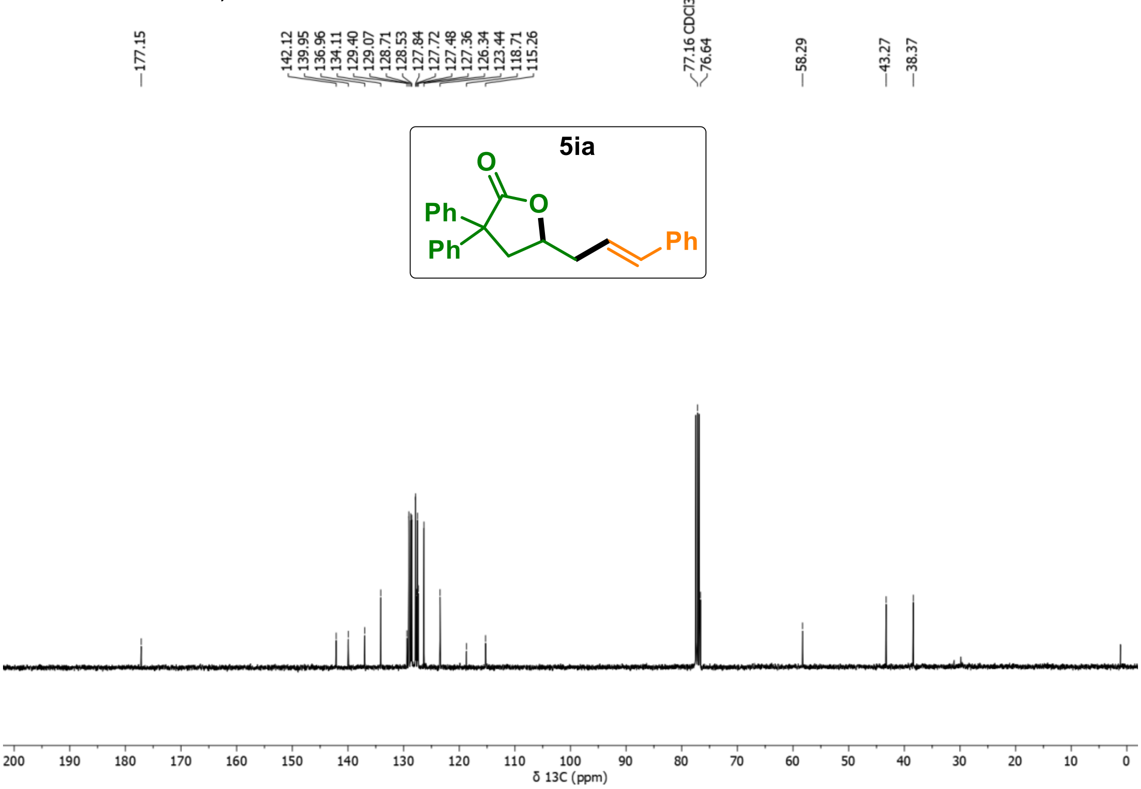

^{1}H NMR 300MHz, $CDCl_3$

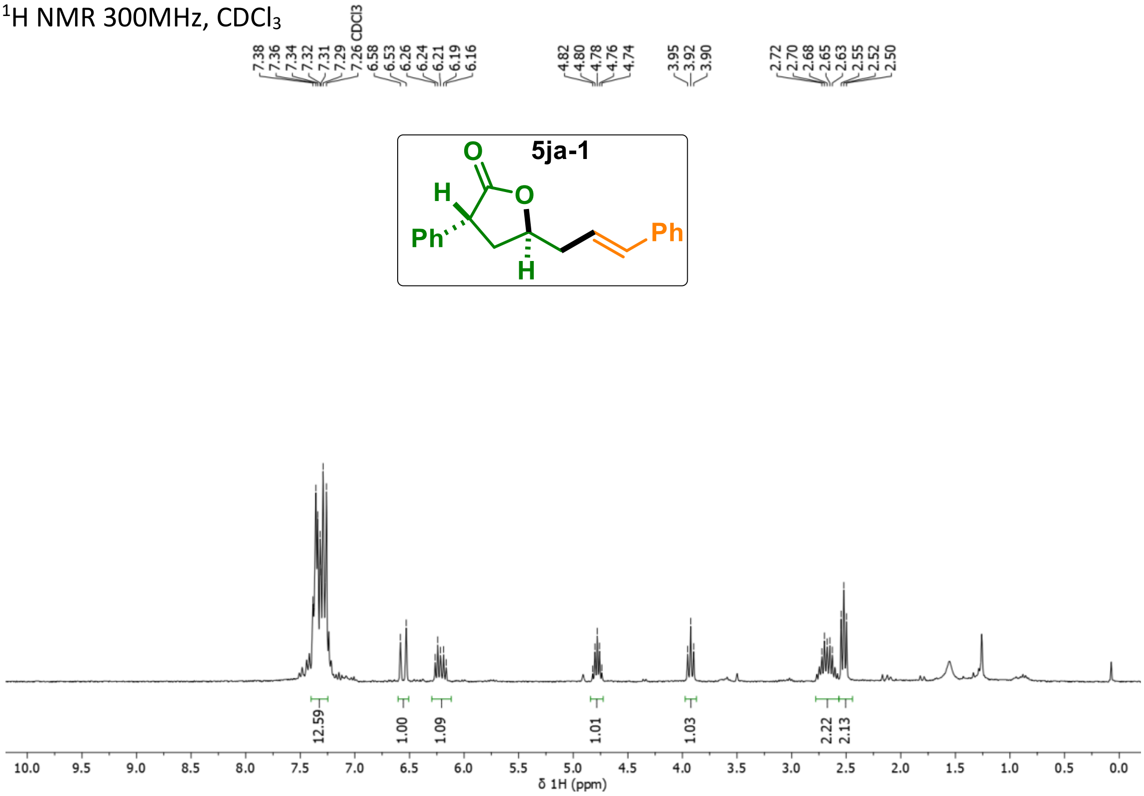

^{13}C NMR 101MHz, $CDCl_3$

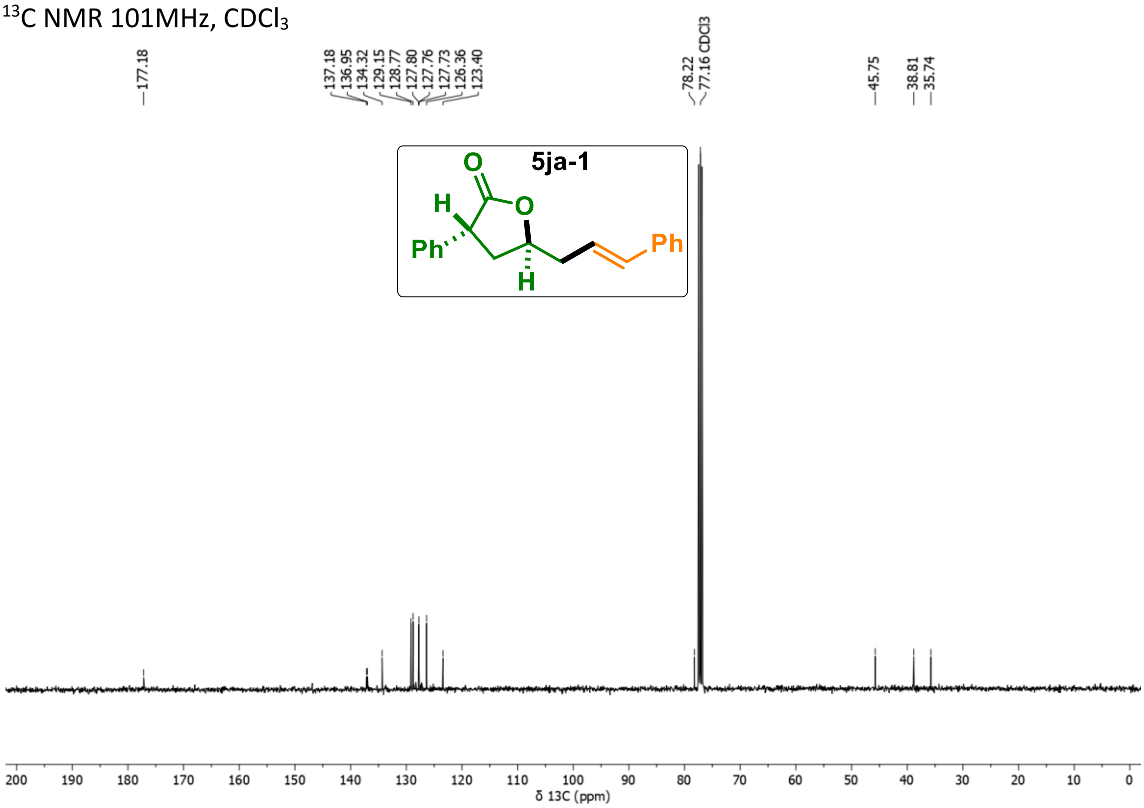

^{1}H NMR 300MHz, $CDCl_3$

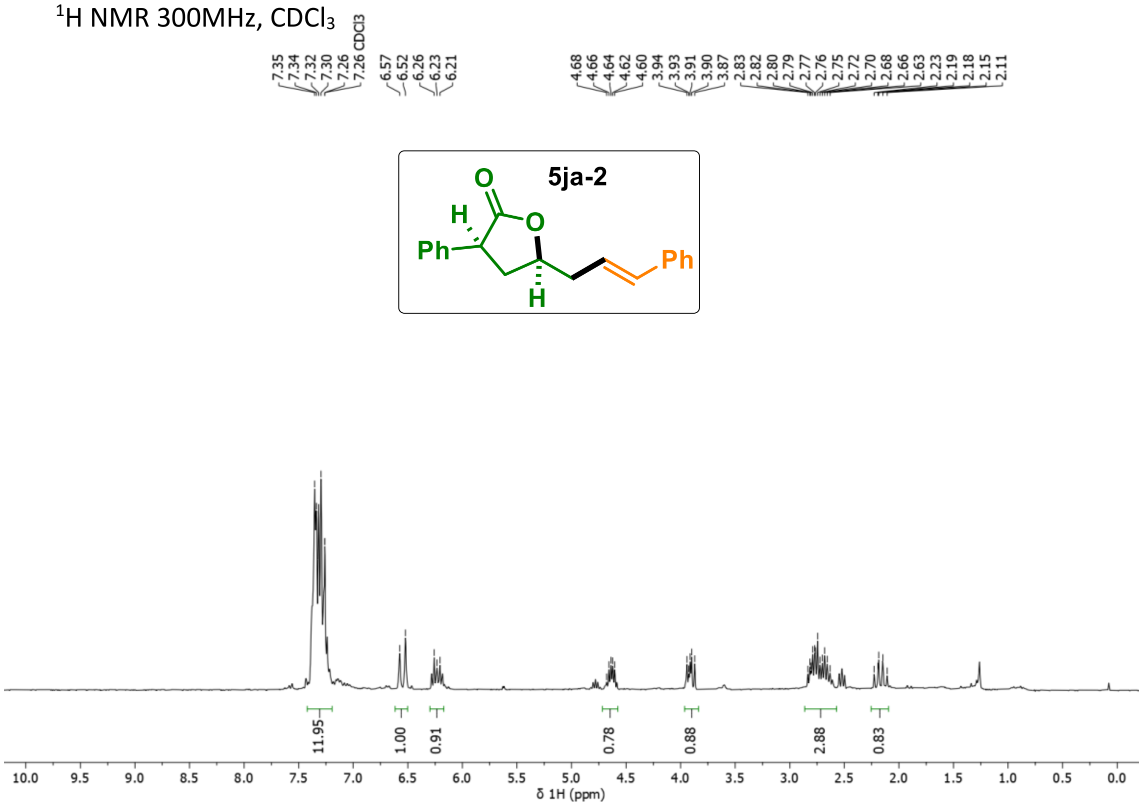

^{13}C NMR 101MHz, $CDCl_3$

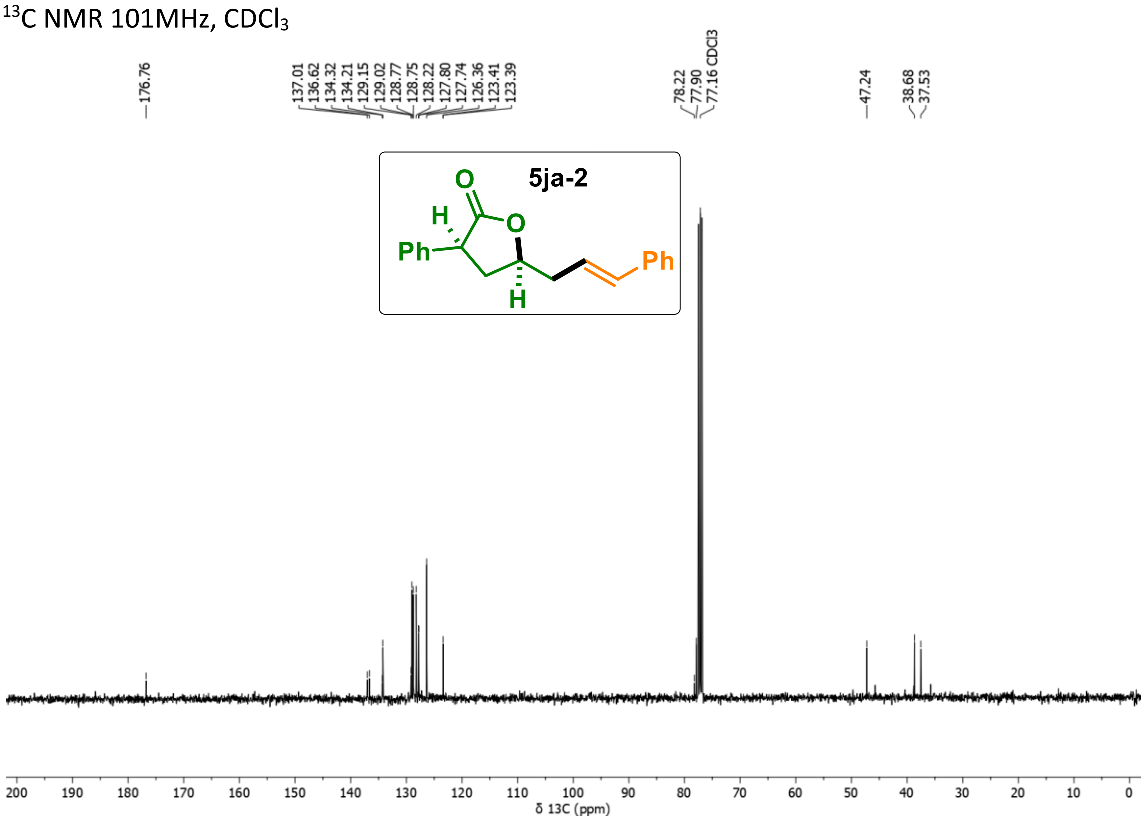

^{1}H NMR 300MHz, $CDCl_3$

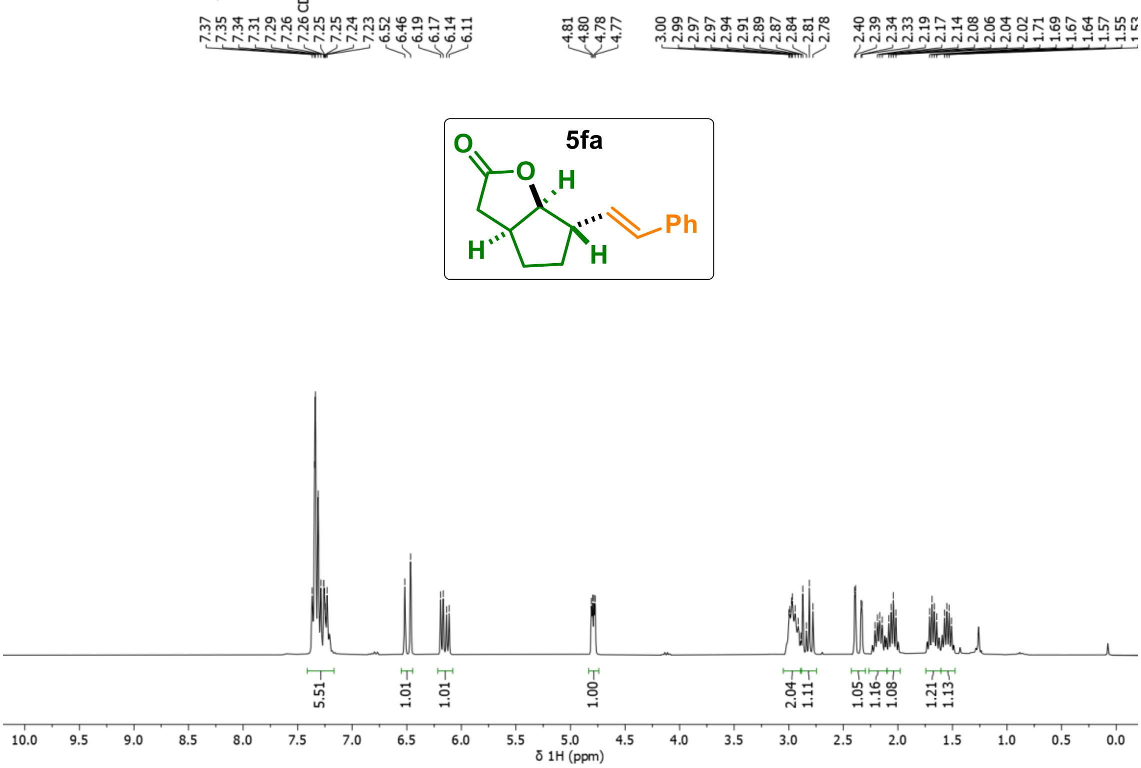

^{13}C NMR 101MHz, $CDCl_3$

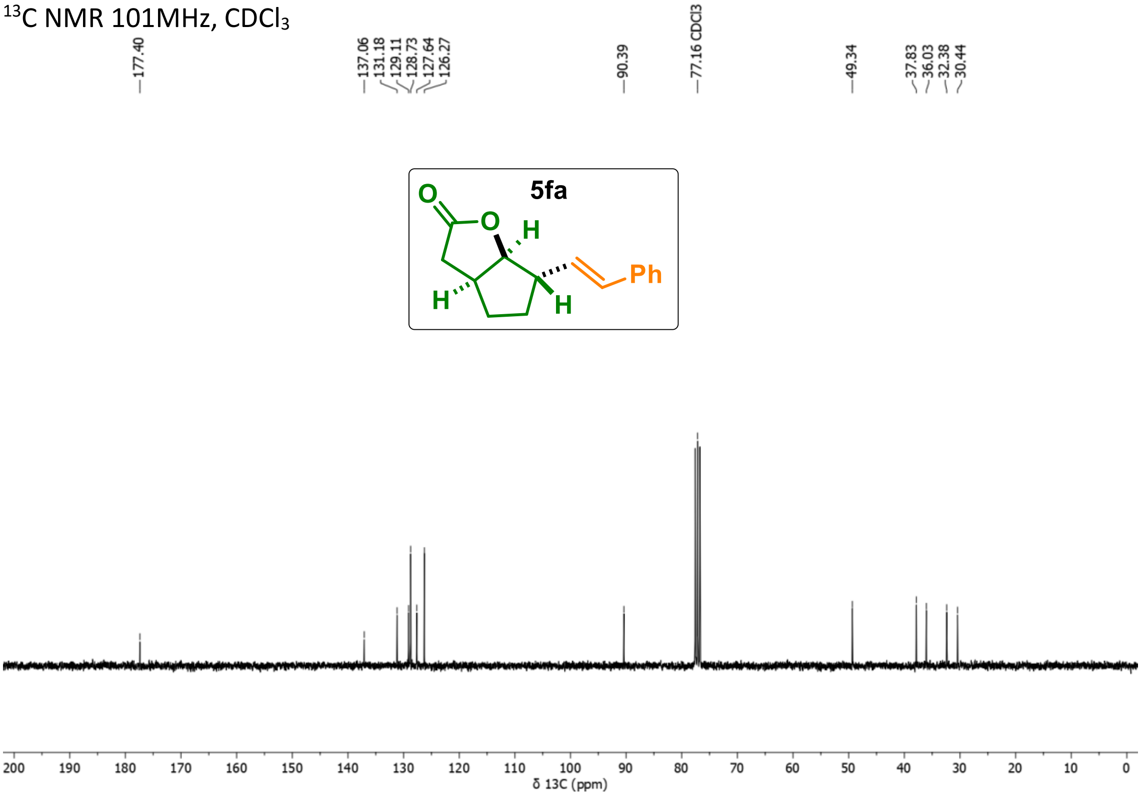

^{1}H NMR 300MHz, $CDCl_3$

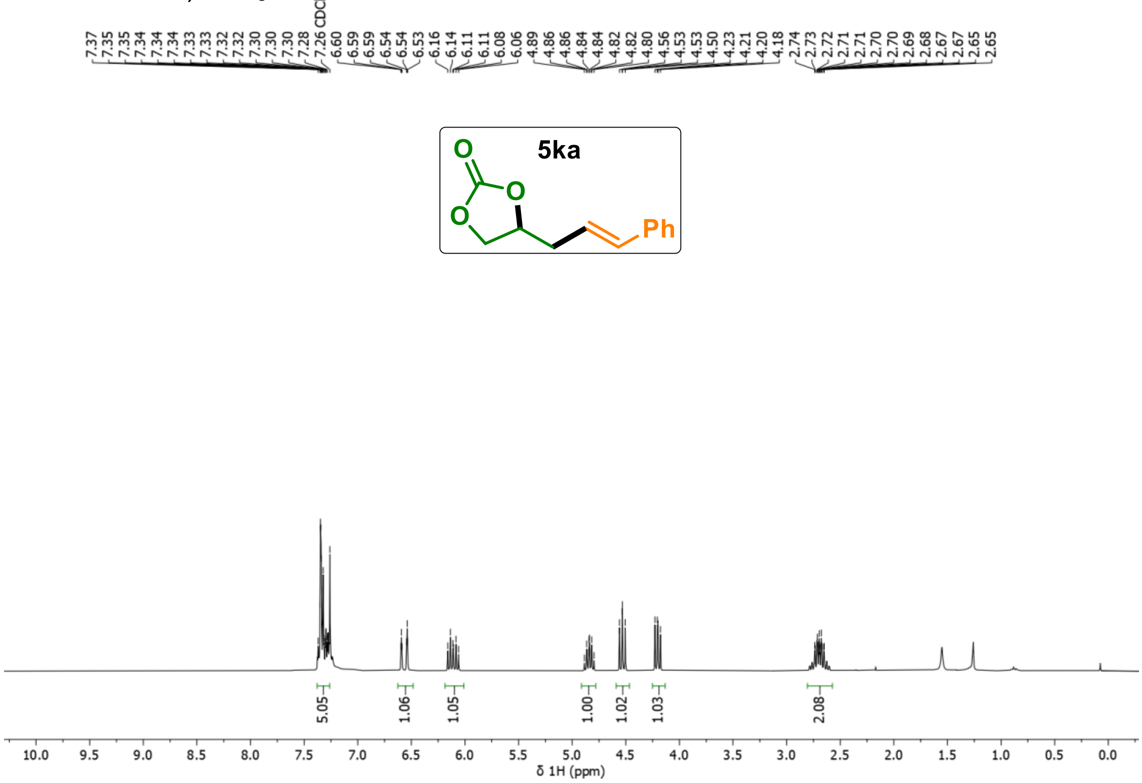

^{13}C NMR 101MHz, $CDCl_3$

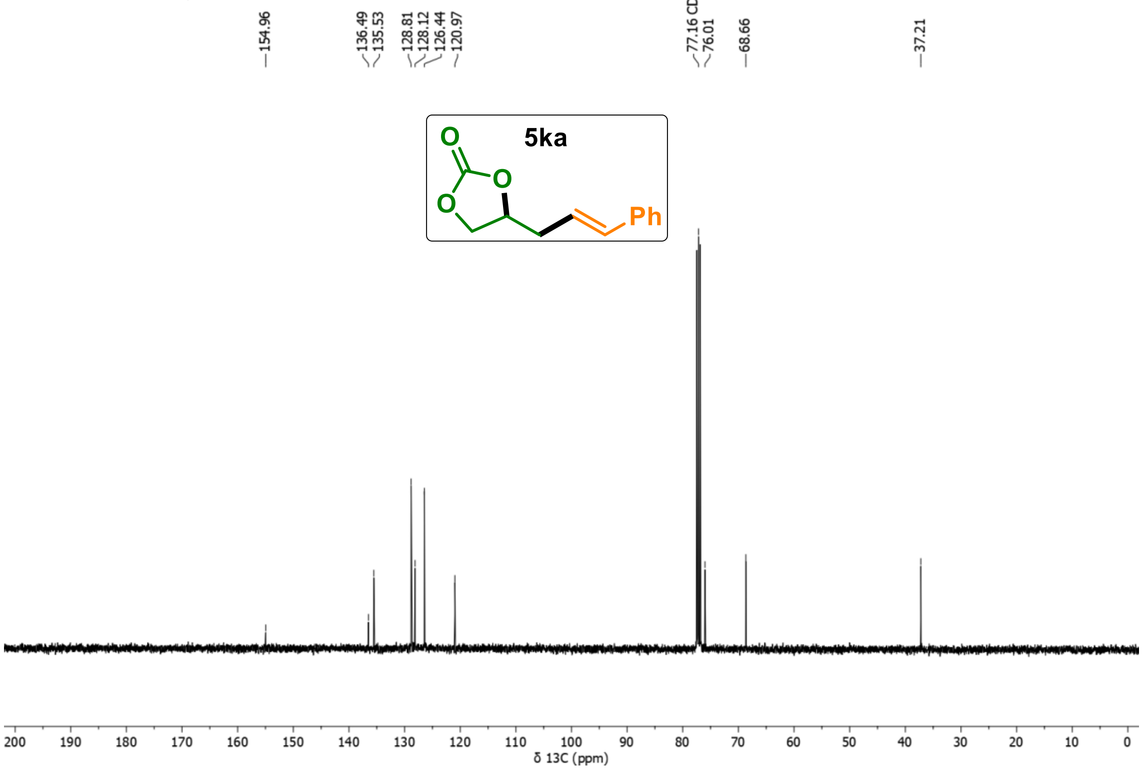

Table of contents

^{1}H NMR 300MHz, $CDCl_3$

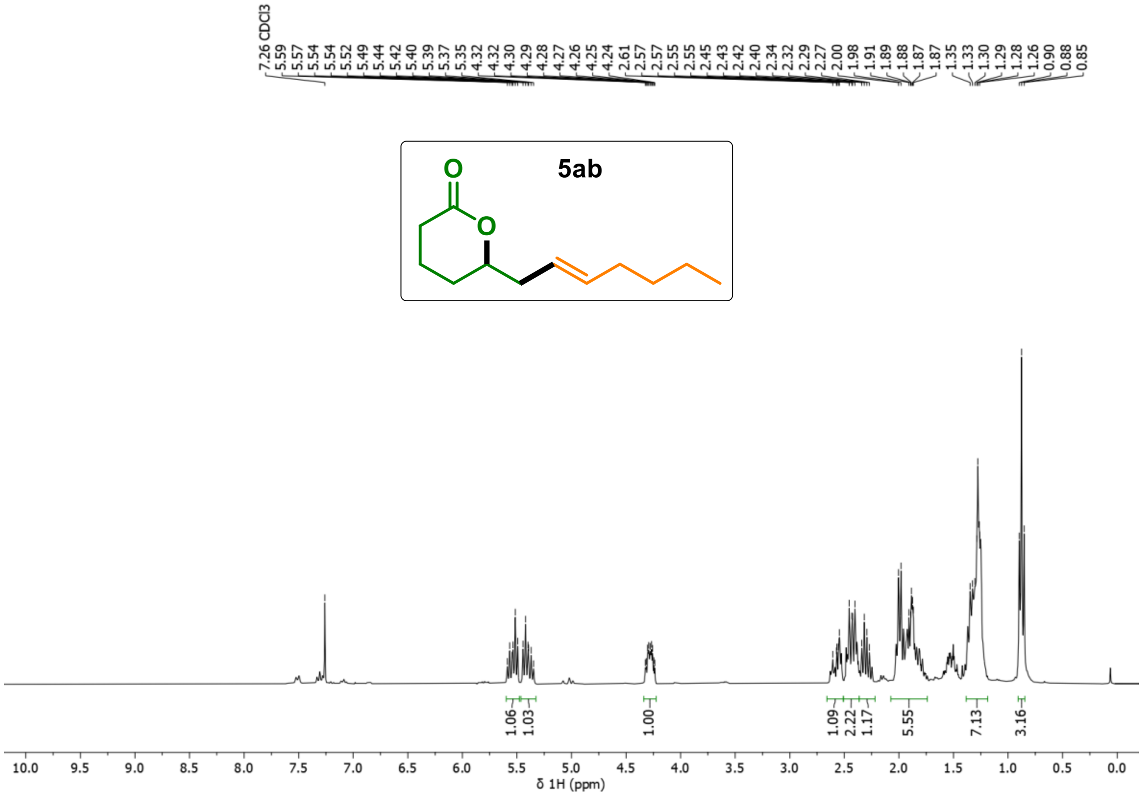

^{13}C NMR 101MHz, $CDCl_3$

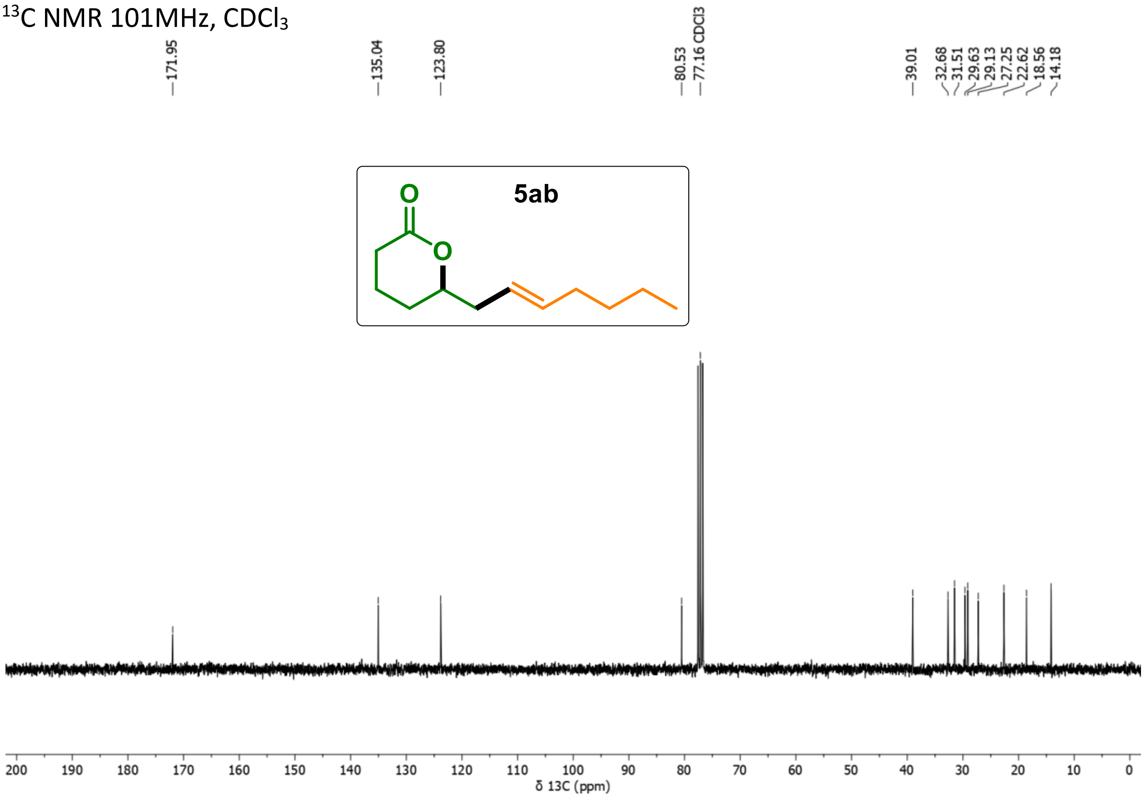

^{1}H NMR 300MHz, $CDCl_3$

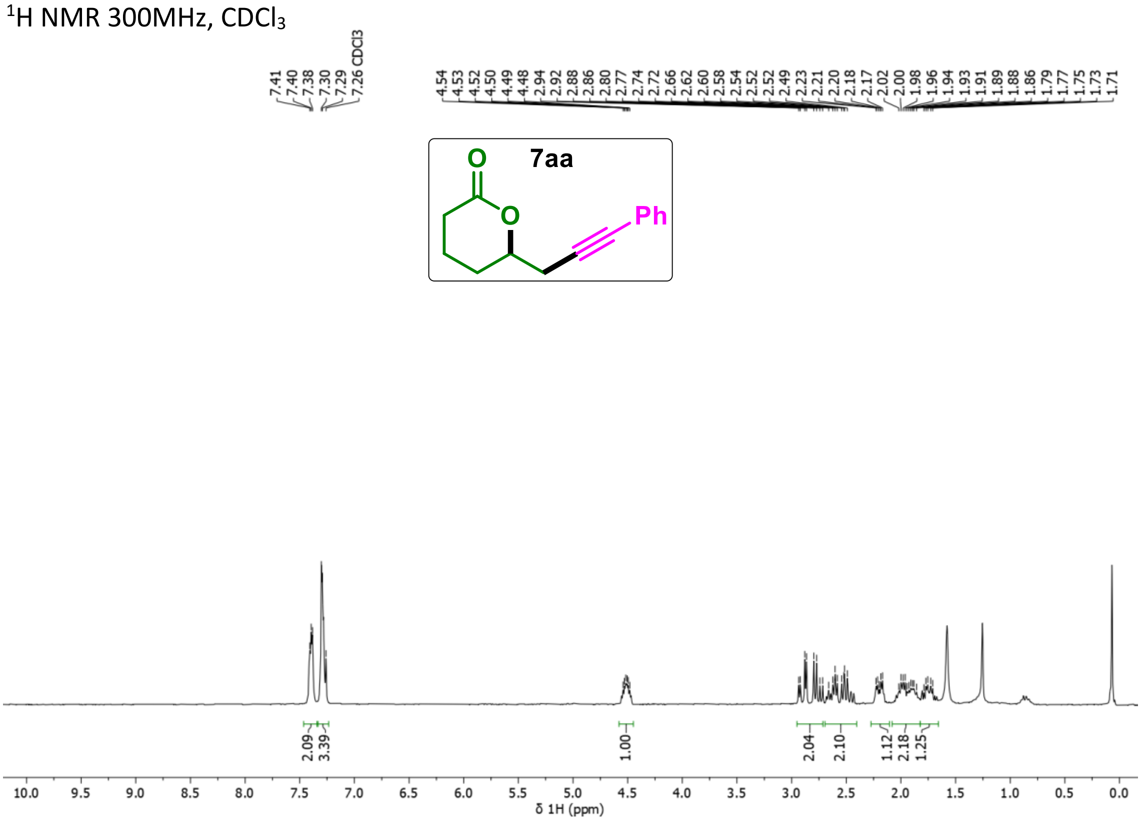

^{13}C NMR 101MHz, $CDCl_3$

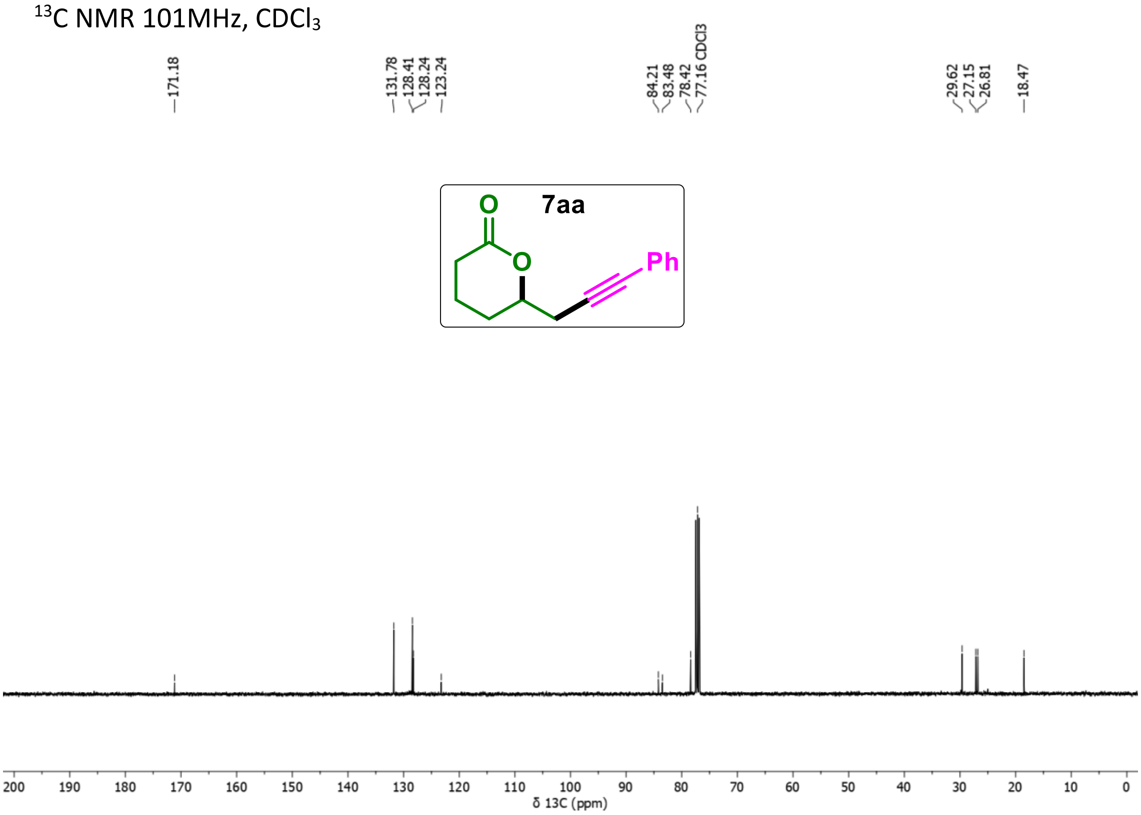

^{1}H NMR 300MHz, CD_2Cl_2

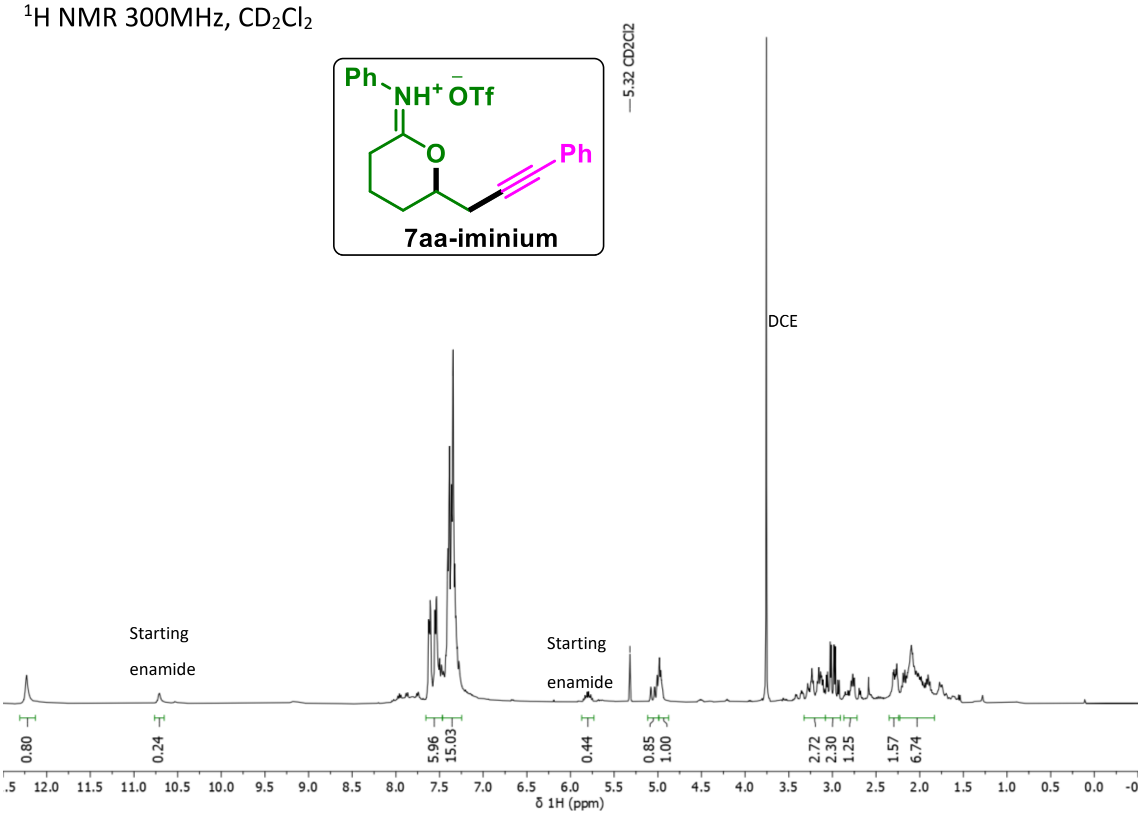

^{19}F NMR 282MHz, CD_2Cl_2

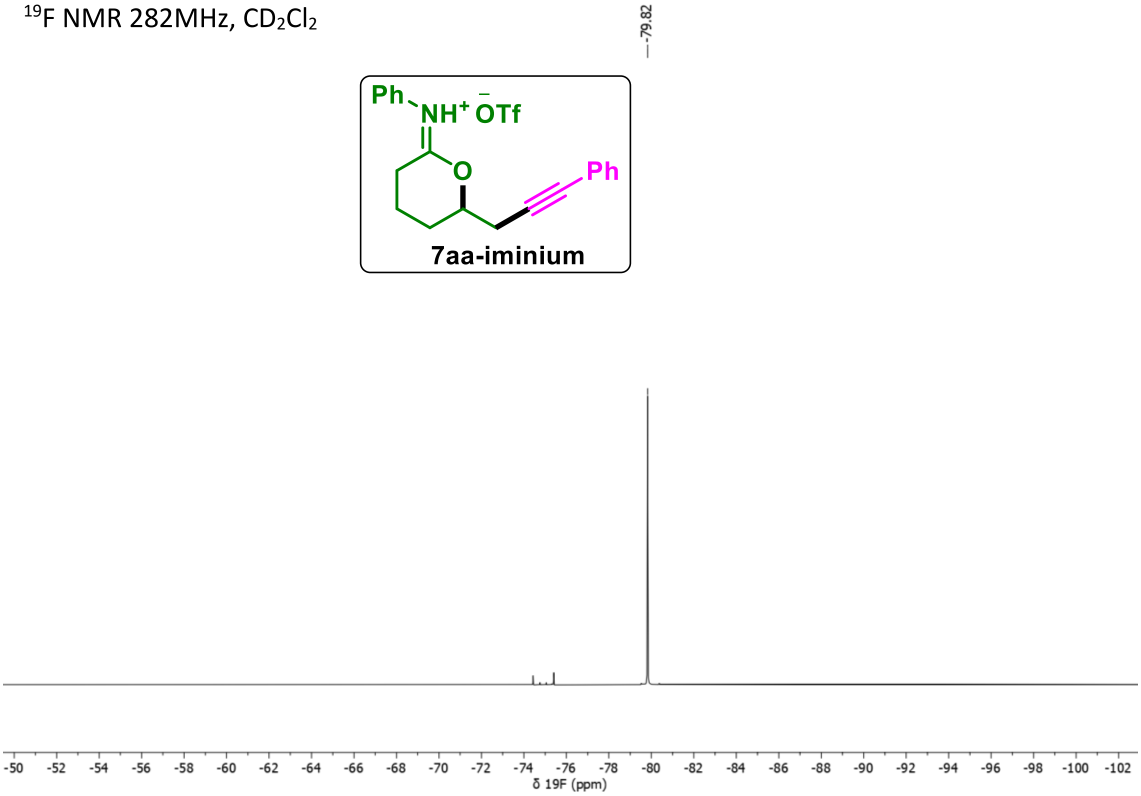

Table of contents

^{13}C NMR 101MHz, CD_2Cl_2

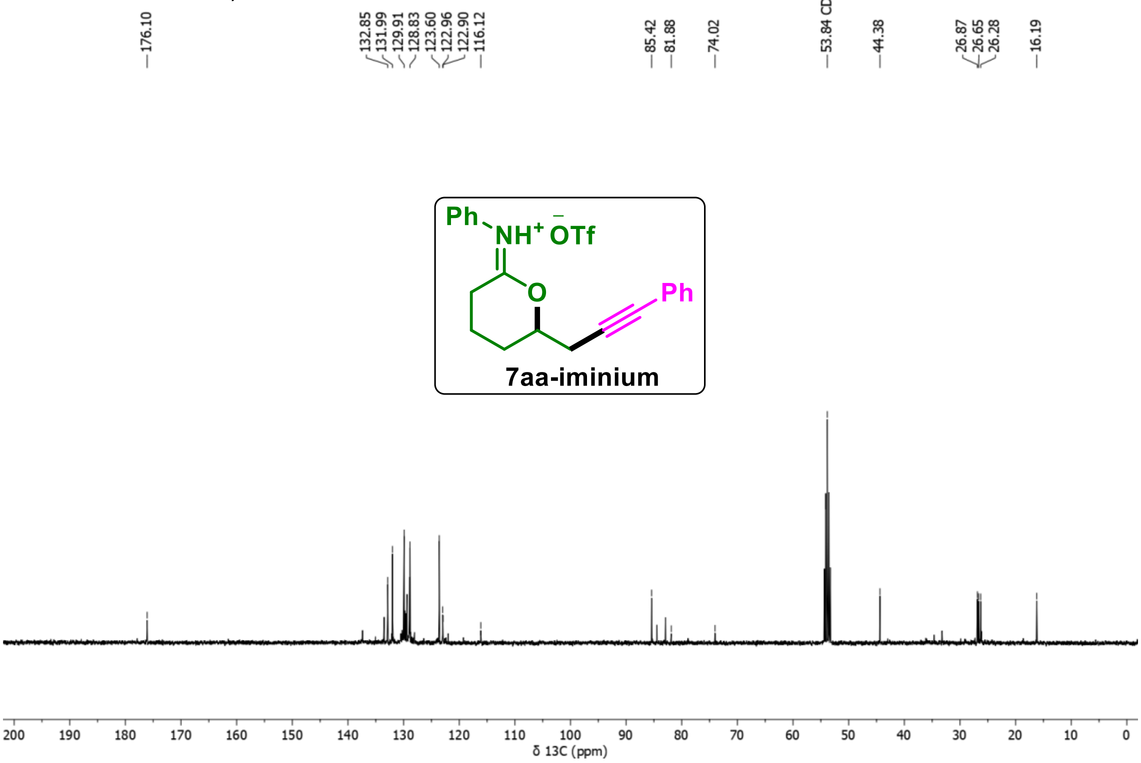

HRMS **7aa-iminium cation**

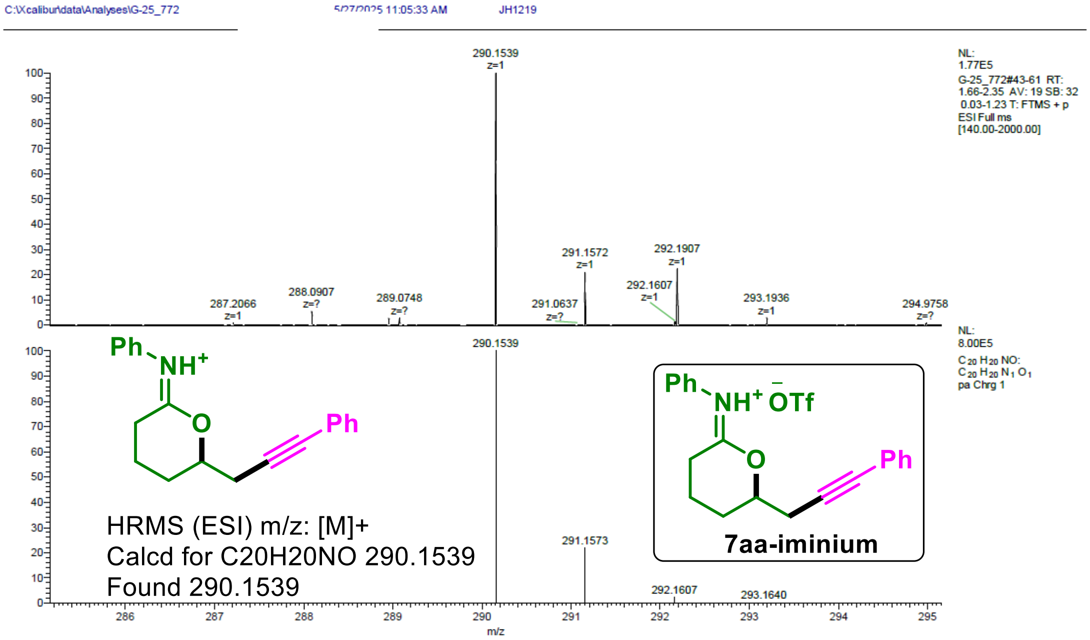

^{1}H NMR 300MHz, $CDCl_3$

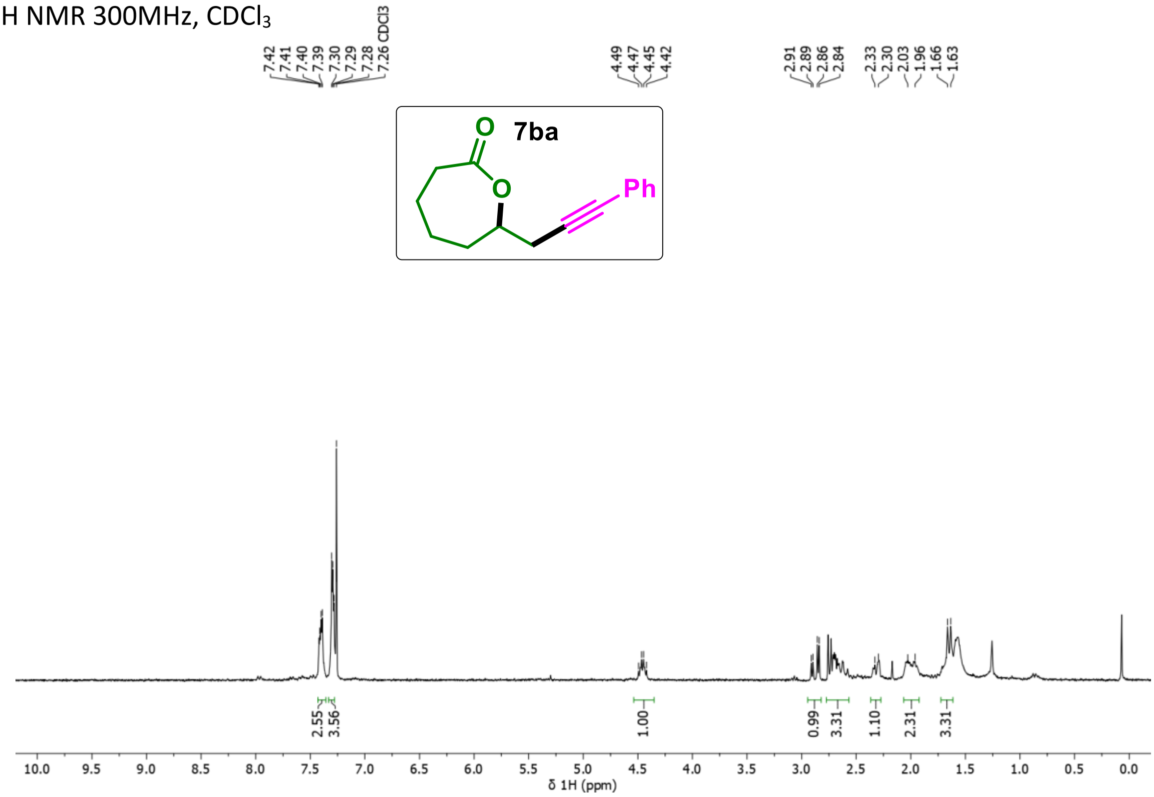

^{13}C NMR 101MHz, $CDCl_3$

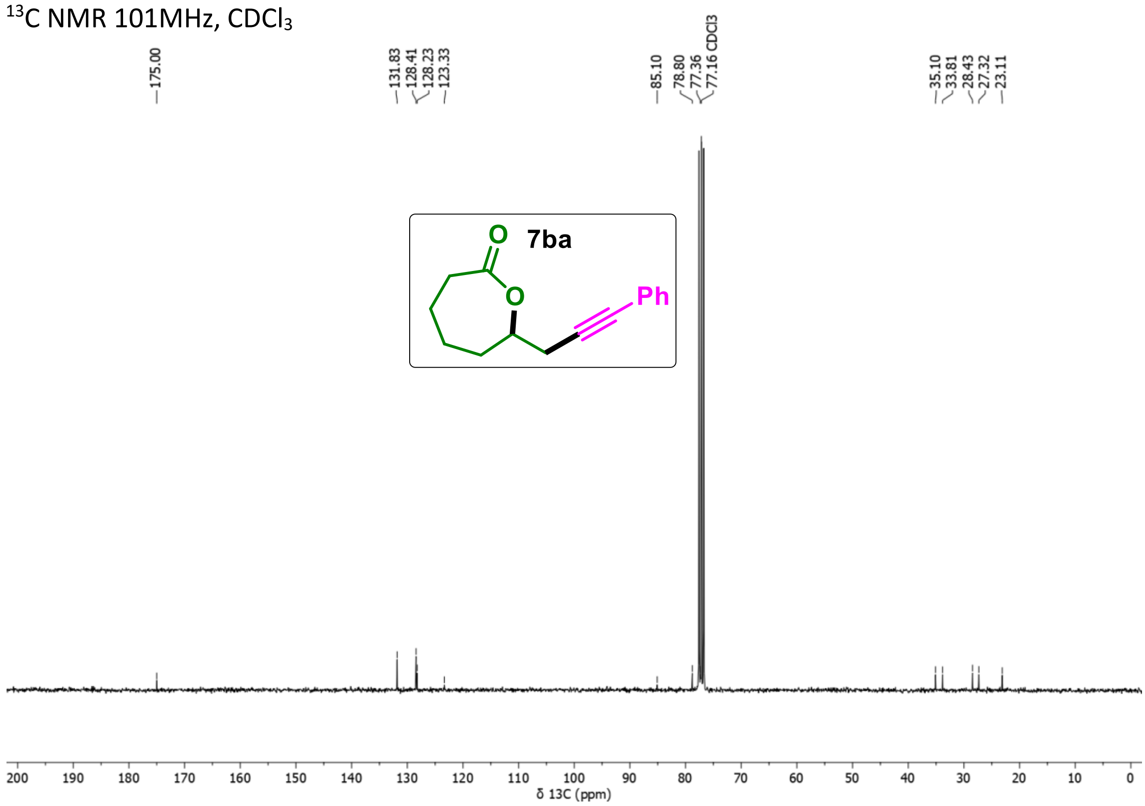

Table of contents

^{1}H NMR 300MHz, $CDCl_3$

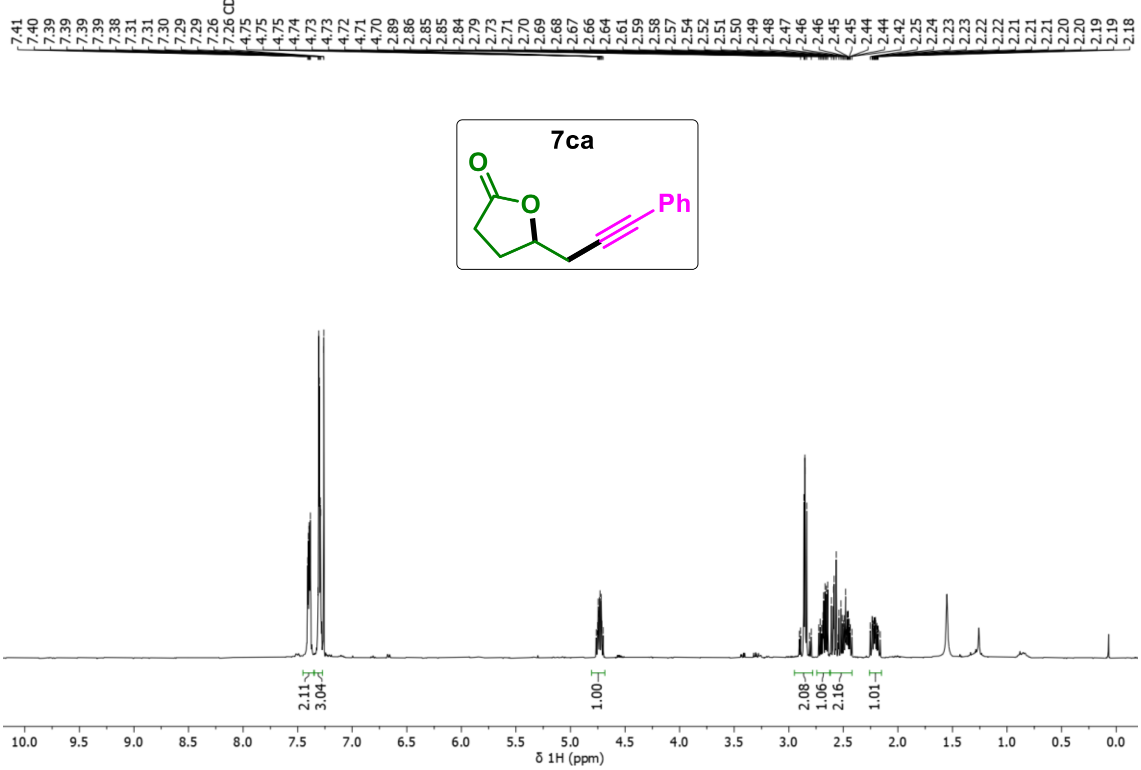

^{13}C NMR 101MHz, $CDCl_3$

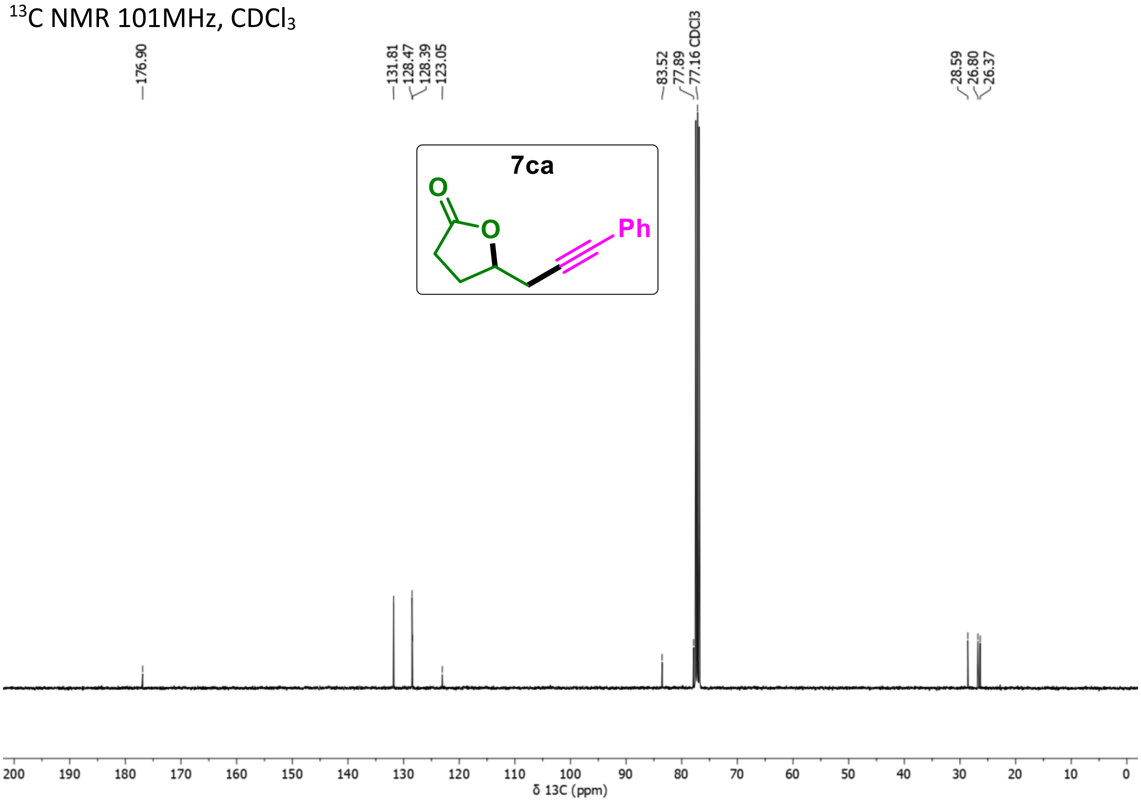

^1H NMR 300MHz, $CDCl_3$

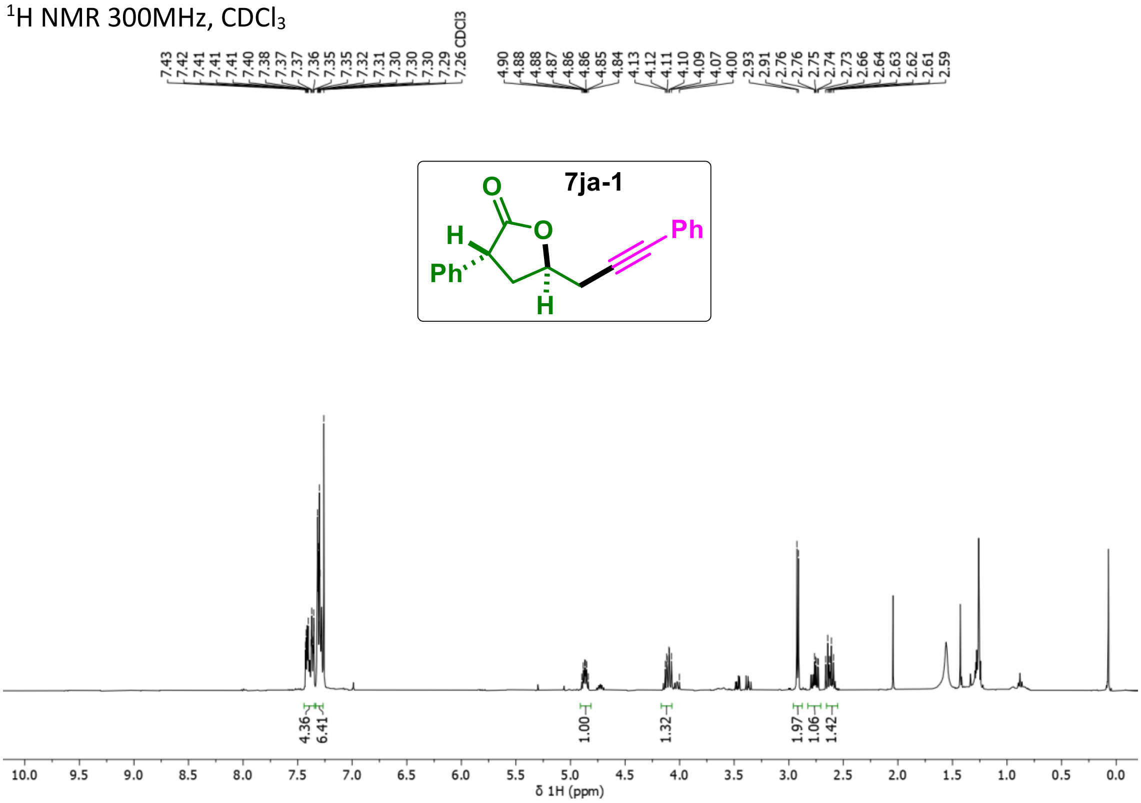

^{13}C NMR 101MHz, $CDCl_3$

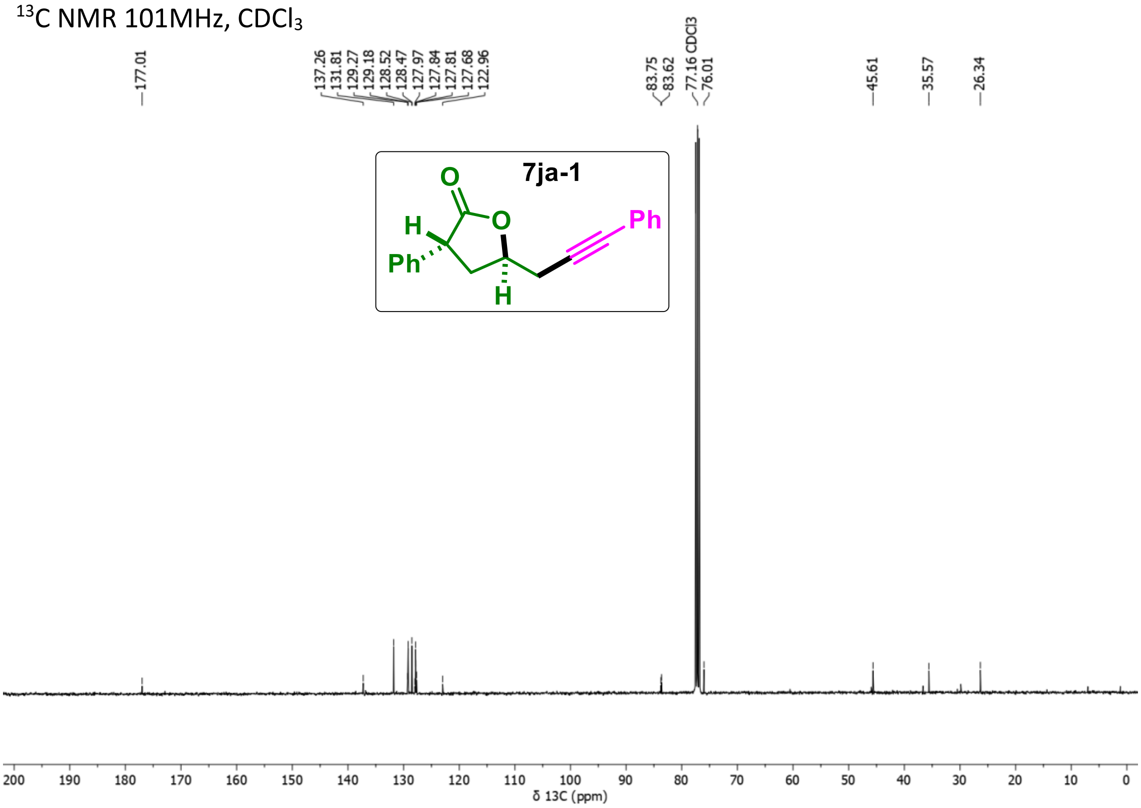

^{1}H NMR 300MHz, $CDCl_3$

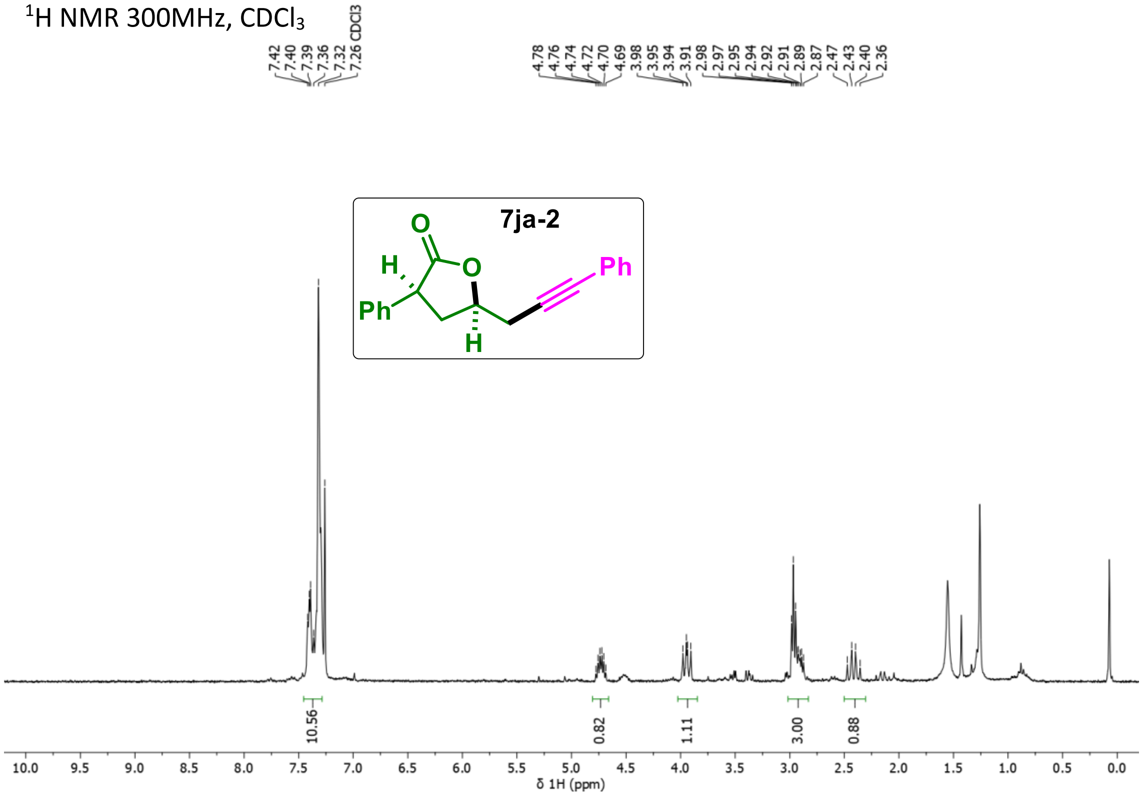

^{13}C NMR 101MHz, $CDCl_3$

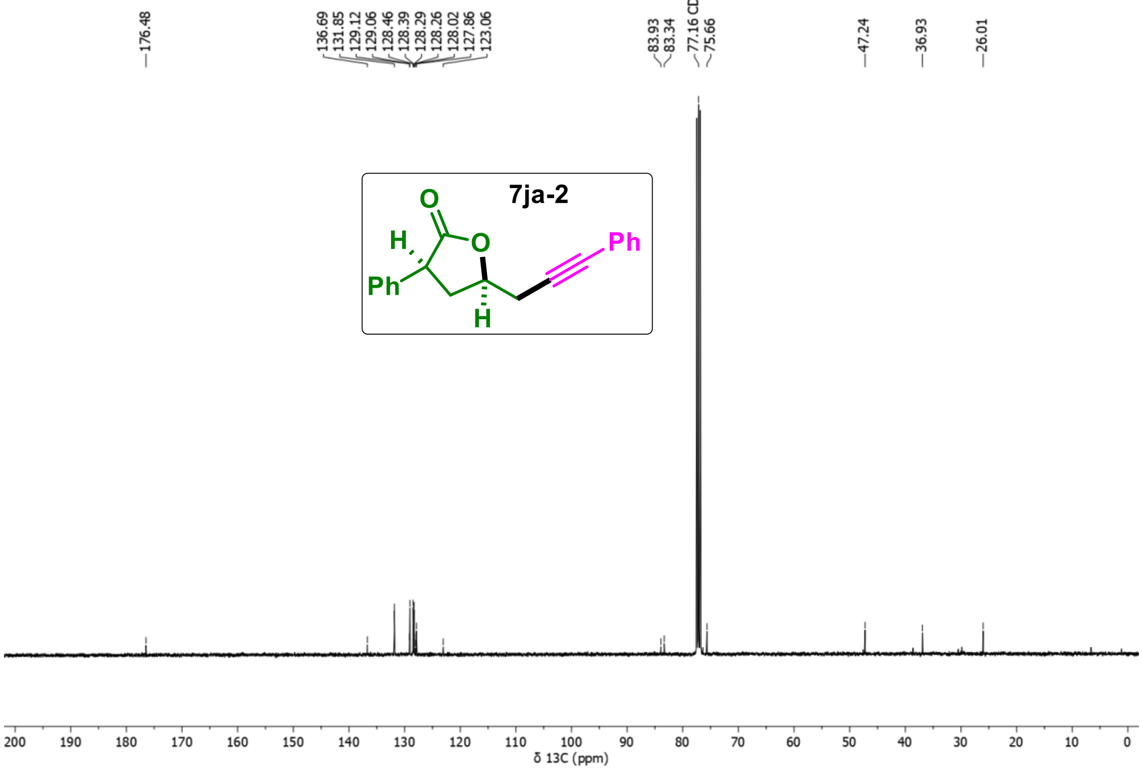

^{1}H NMR 300MHz, $CDCl_3$

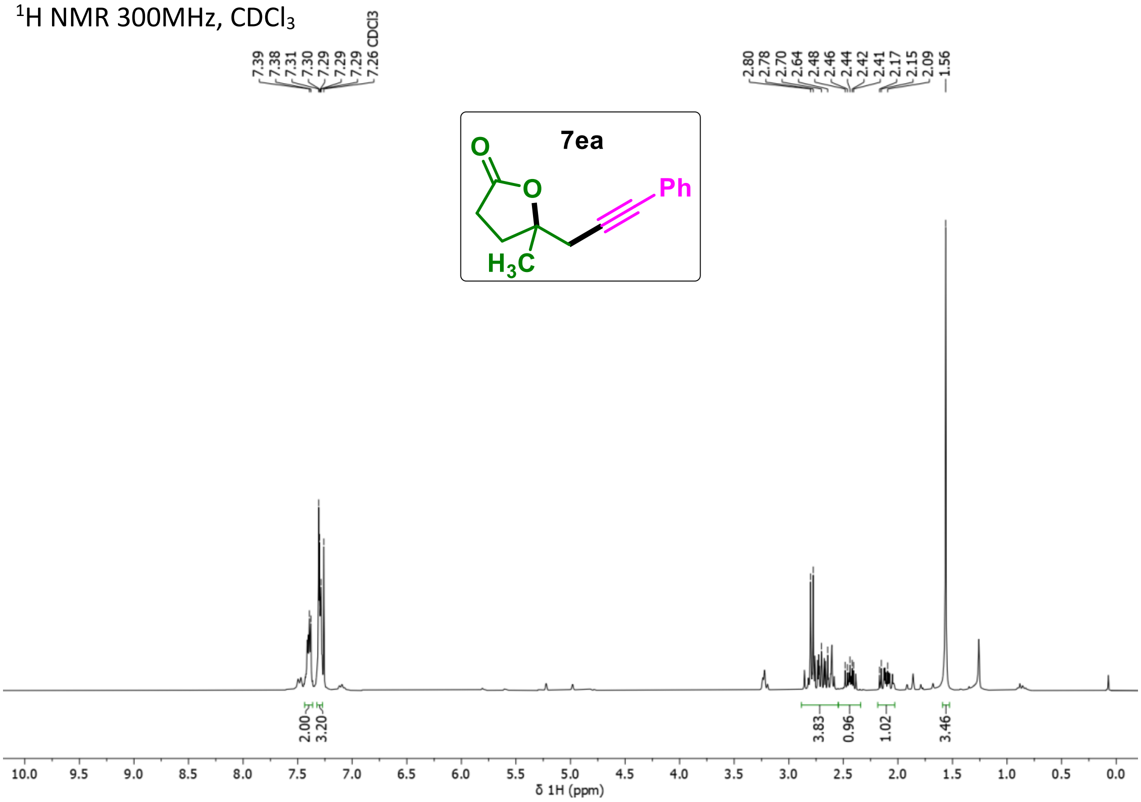

^{13}C NMR 101MHz, $CDCl_3$

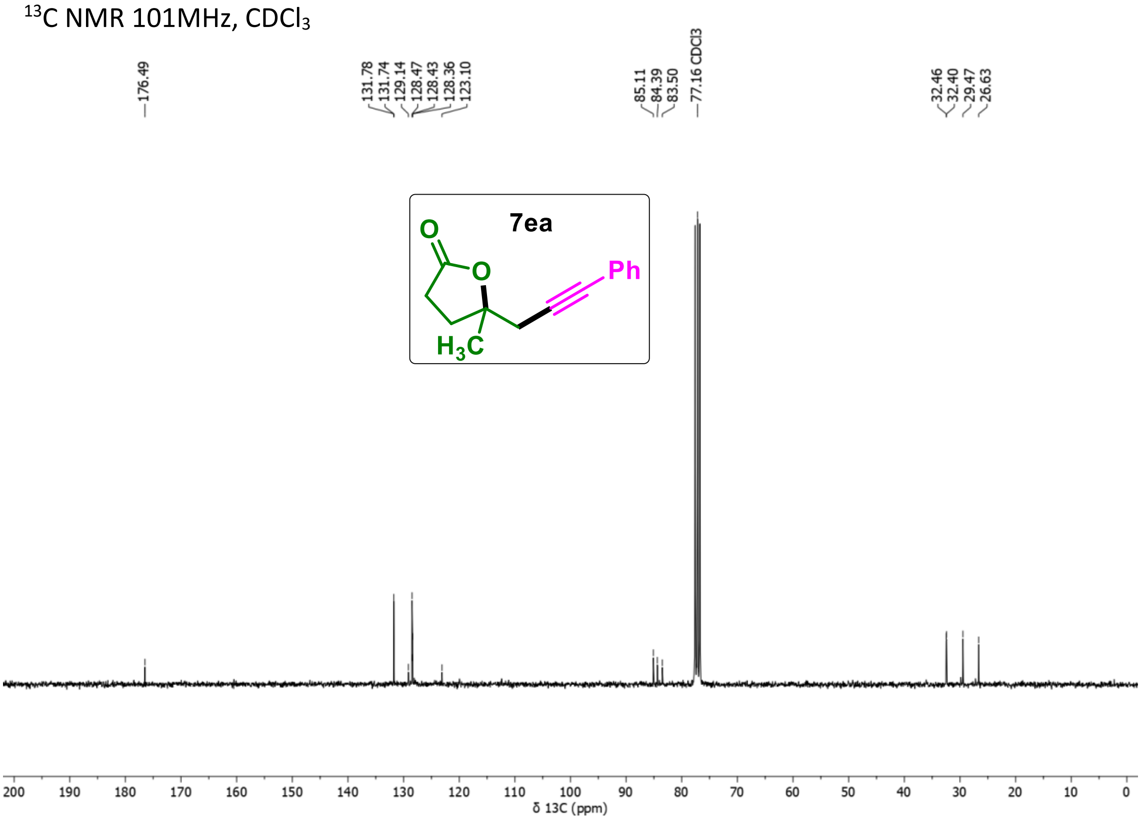

^{1}H NMR 300MHz, $CDCl_3$

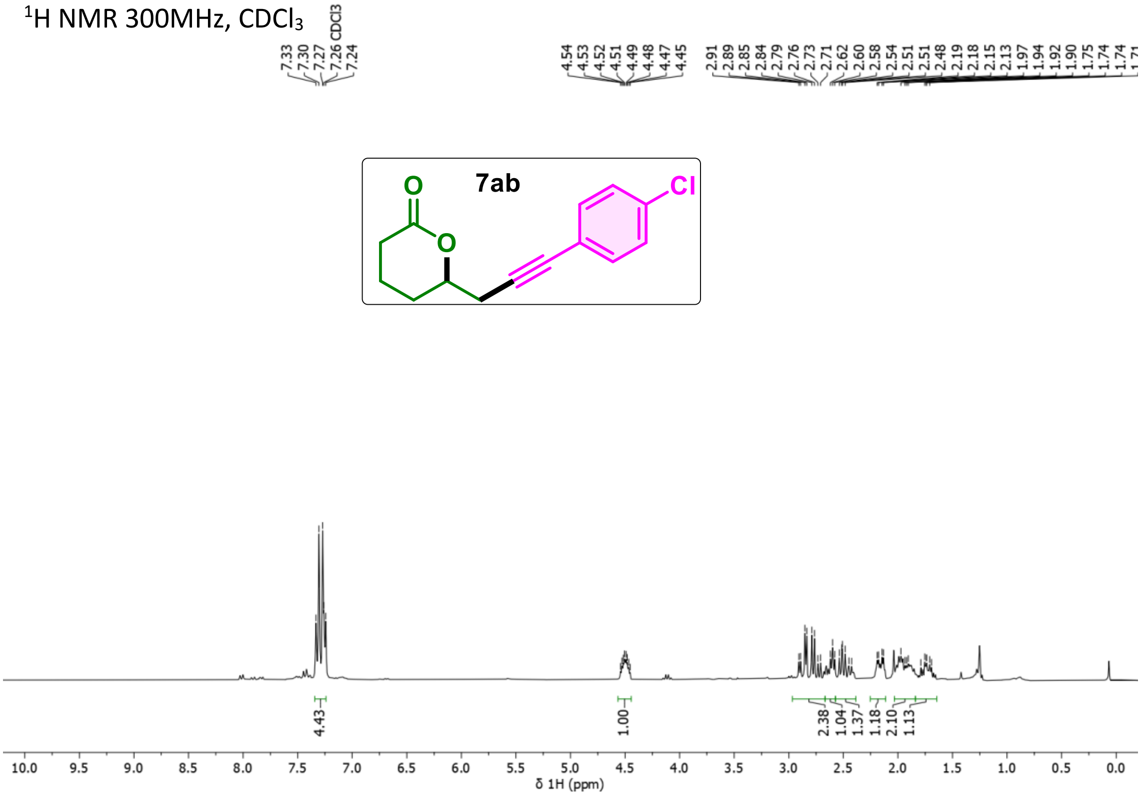

^{13}C NMR 101MHz, $CDCl_3$

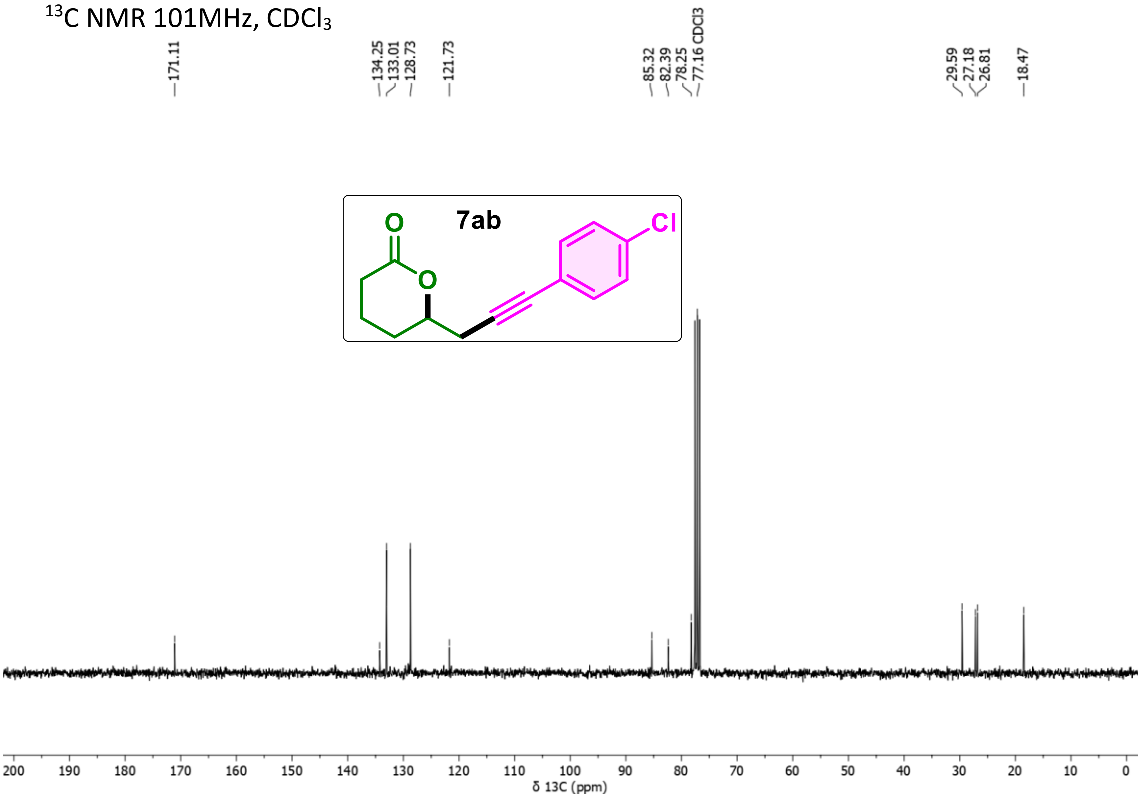

^{1}H NMR 300MHz, $CDCl_3$

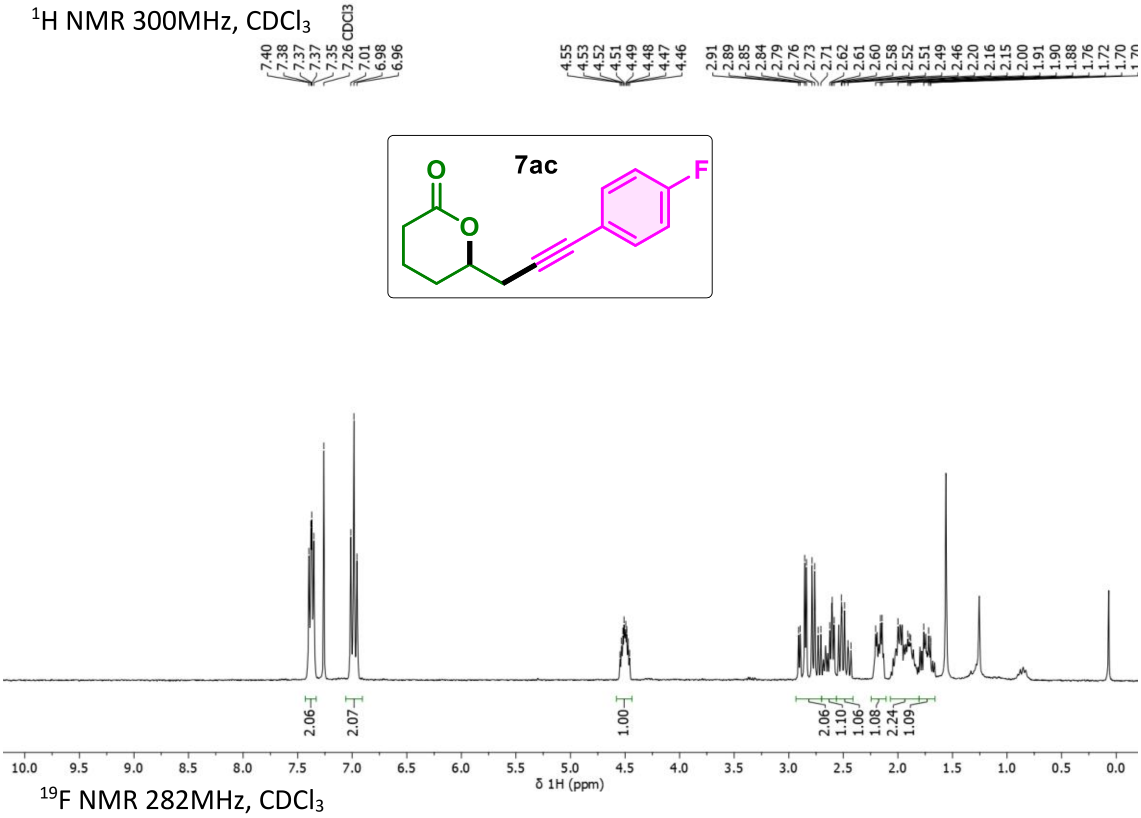

^{19}F NMR 282MHz, $CDCl_3$

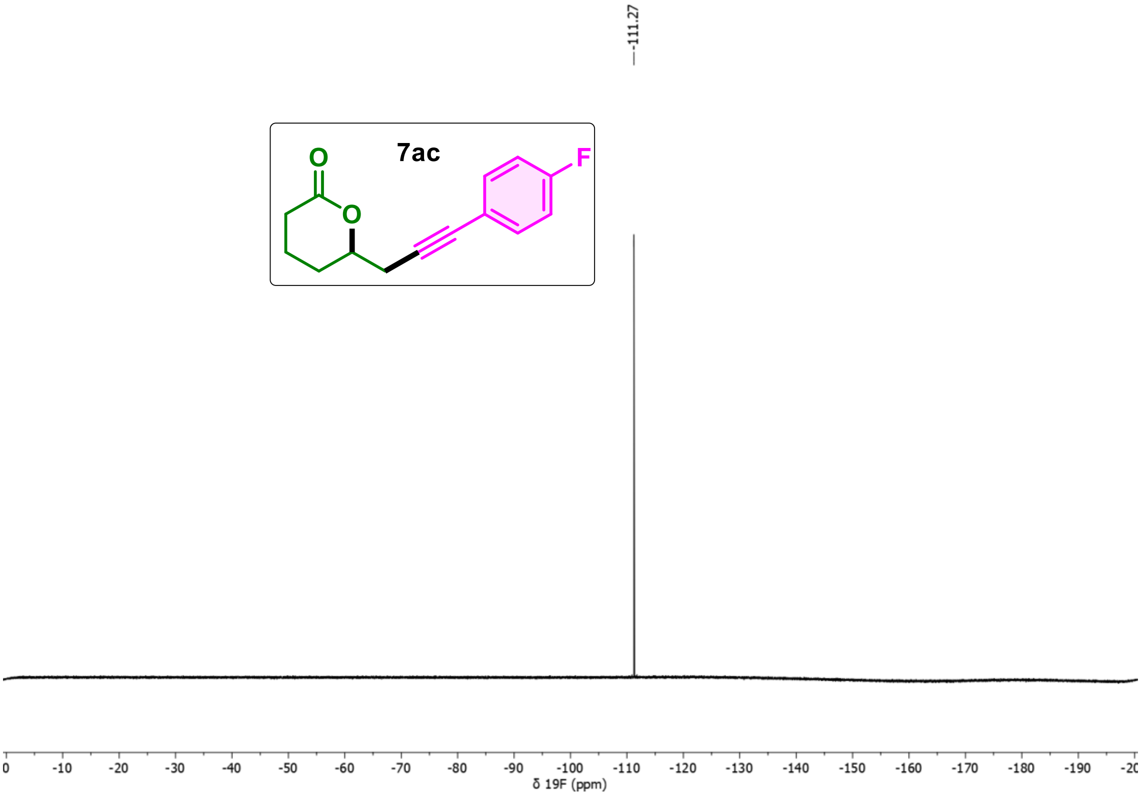

Table of contents

^{13}C NMR 101MHz, $CDCl_3$

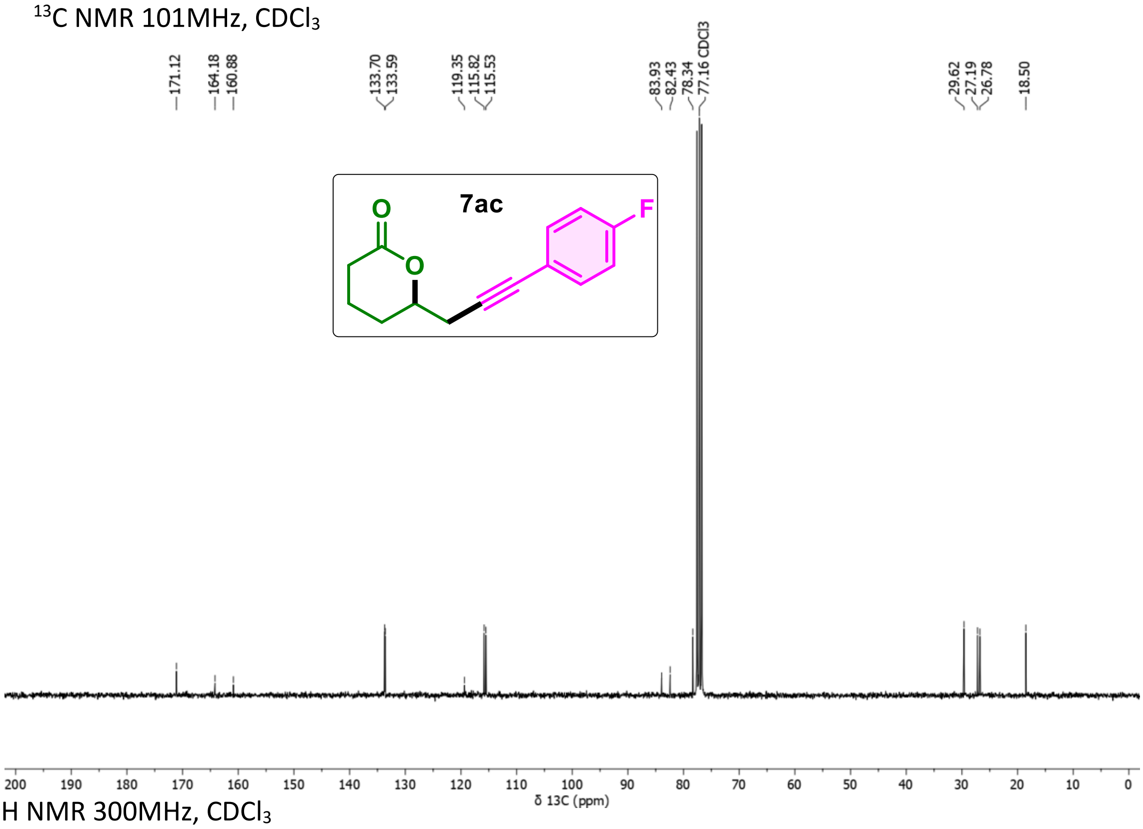

^{1}H NMR 300MHz, $CDCl_3$

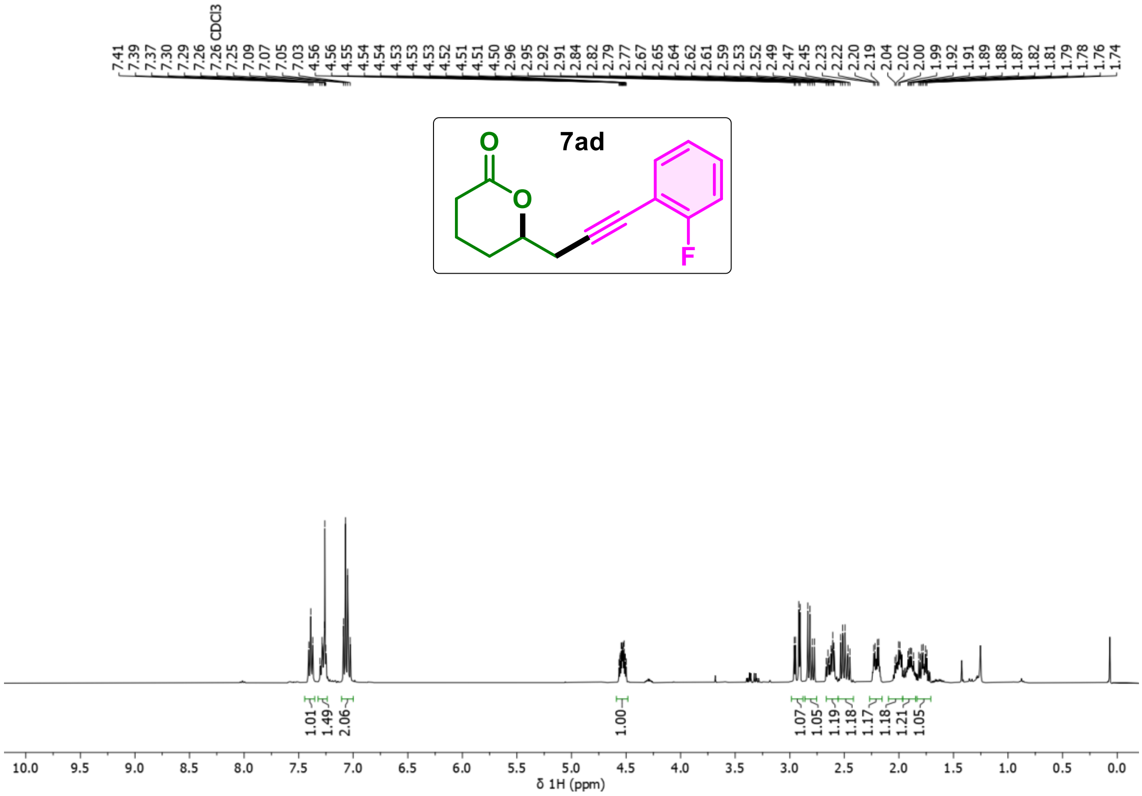

^{19}F NMR 282MHz, $CDCl_3$

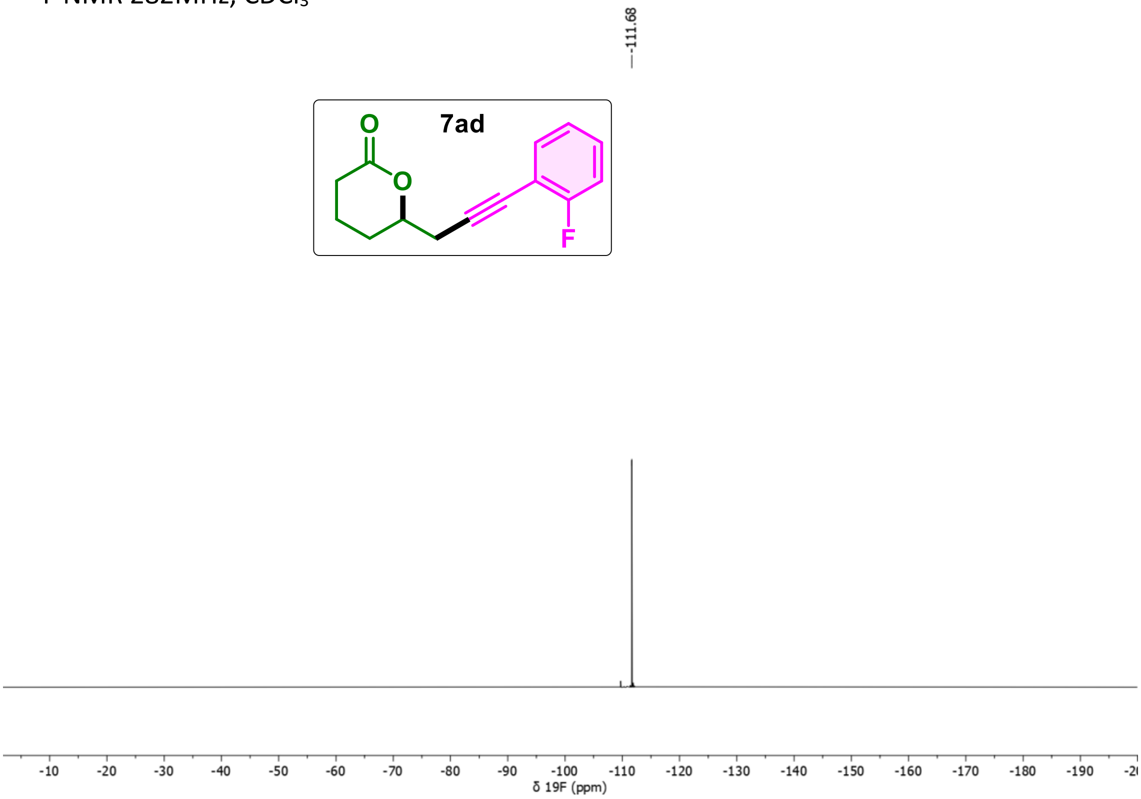

^{13}C NMR 101MHz, $CDCl_3$

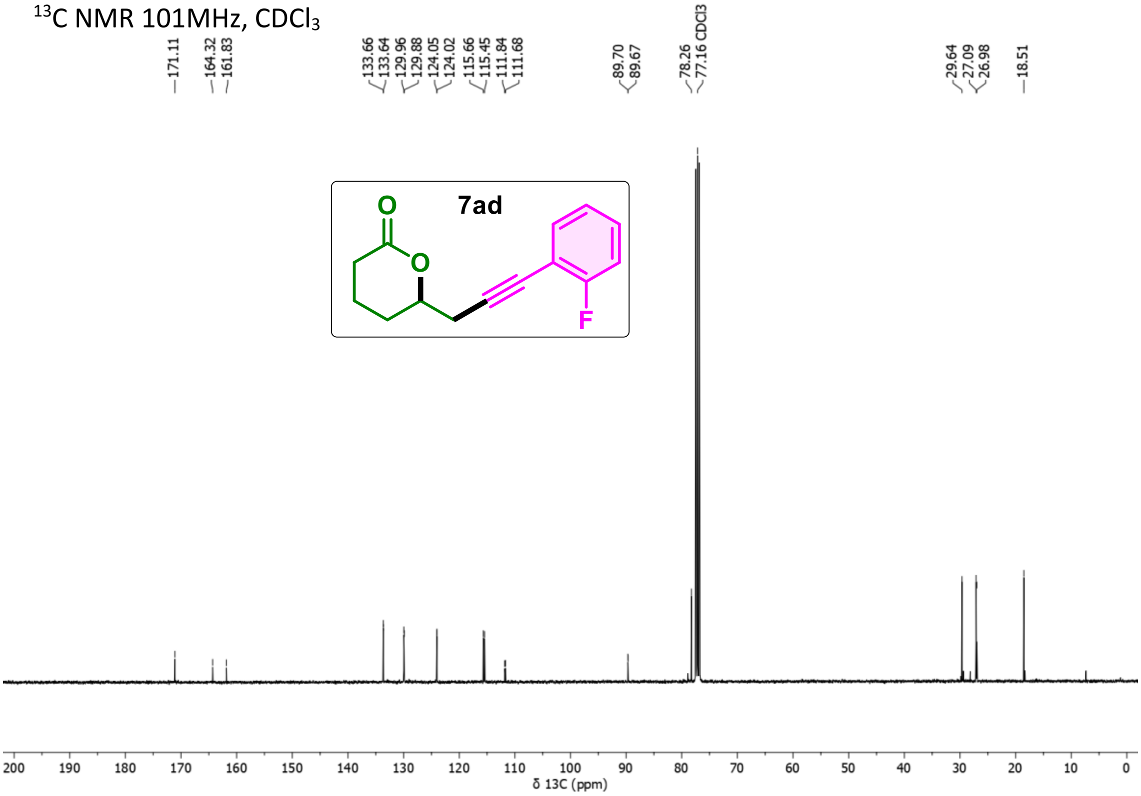

^{1}H NMR 300MHz, $CDCl_3$

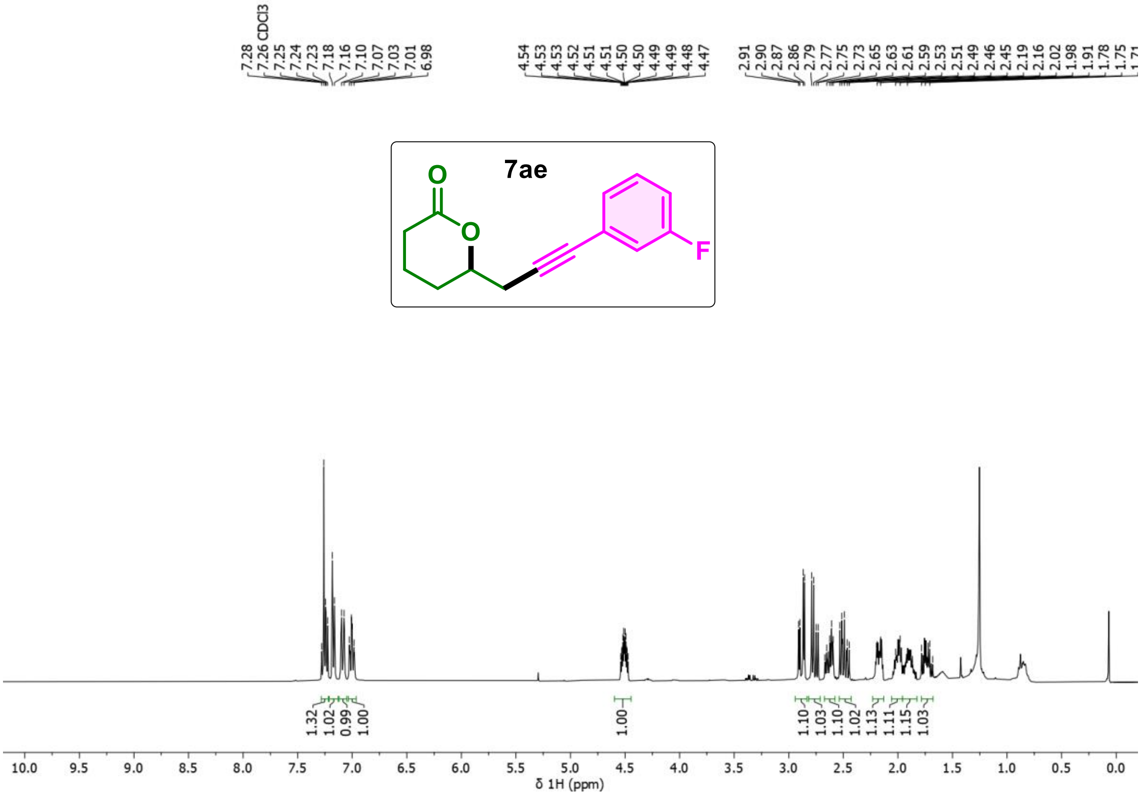

^{19}F NMR 282MHz, $CDCl_3$

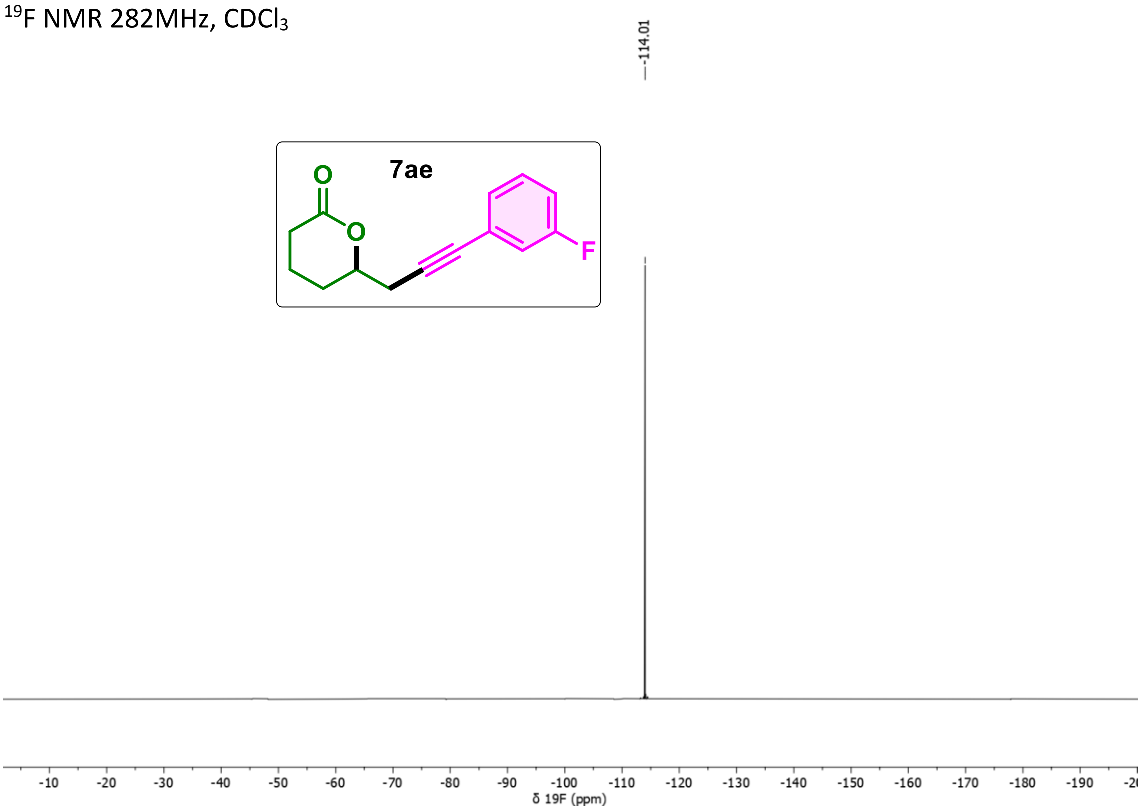

^{13}C NMR 101MHz, CDCl$_3$

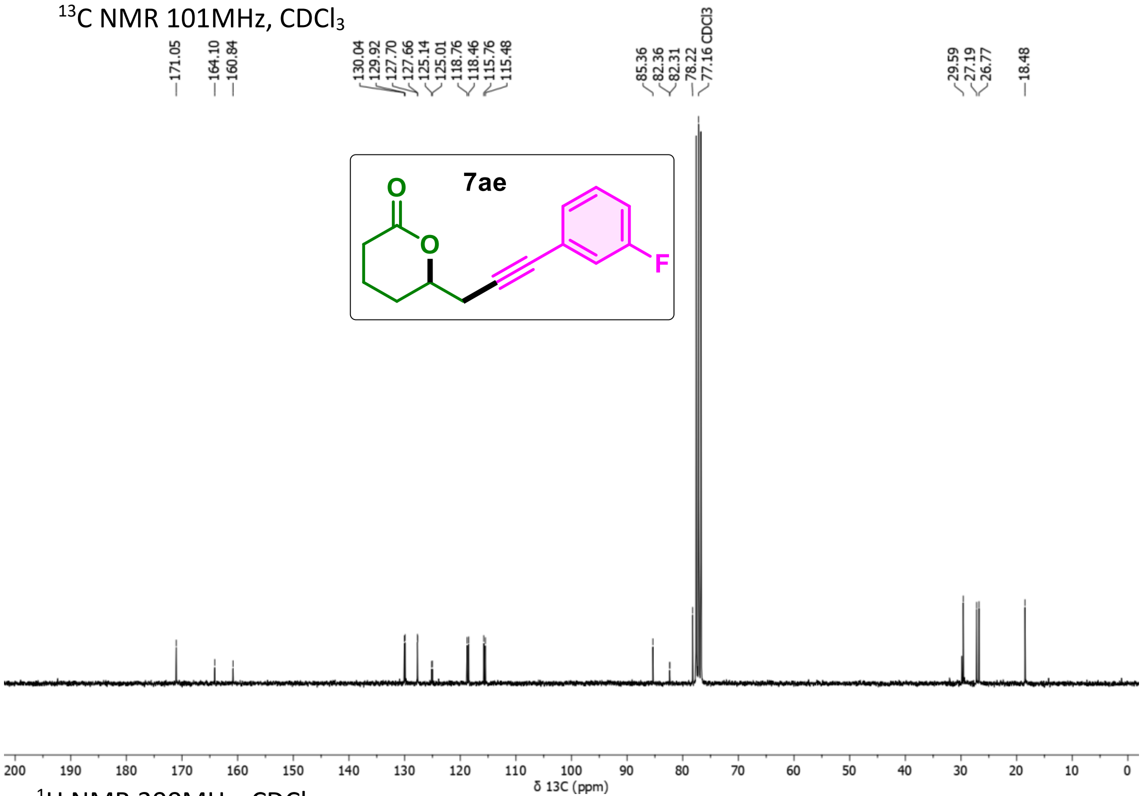

^{1}H NMR 300MHz, CDCl$_3$

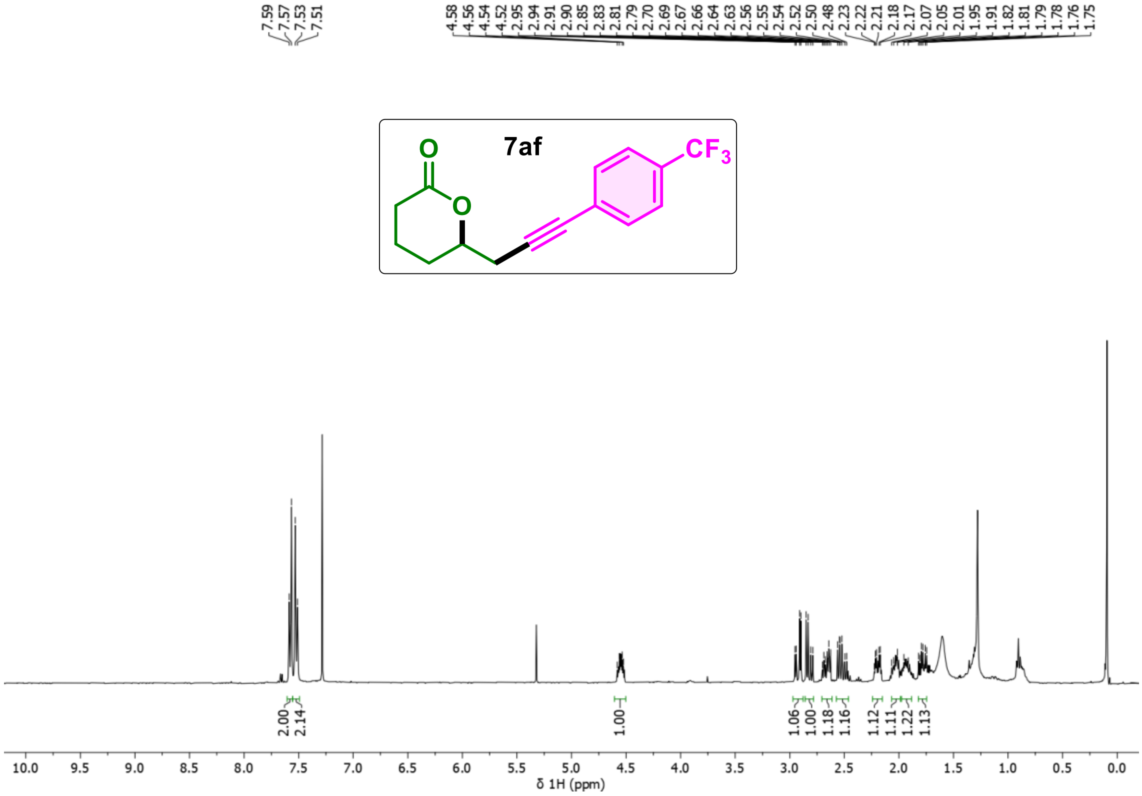

^{19}F NMR 282MHz, $CDCl_3$

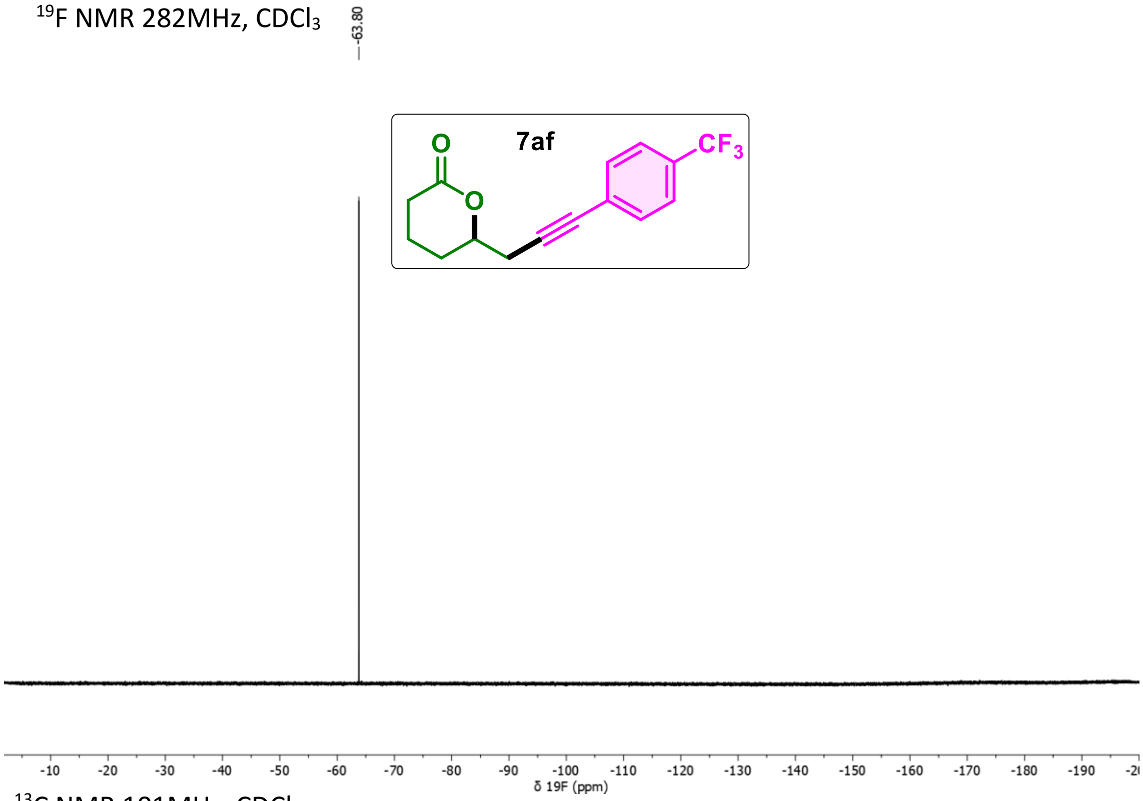

^{13}C NMR 101MHz, $CDCl_3$

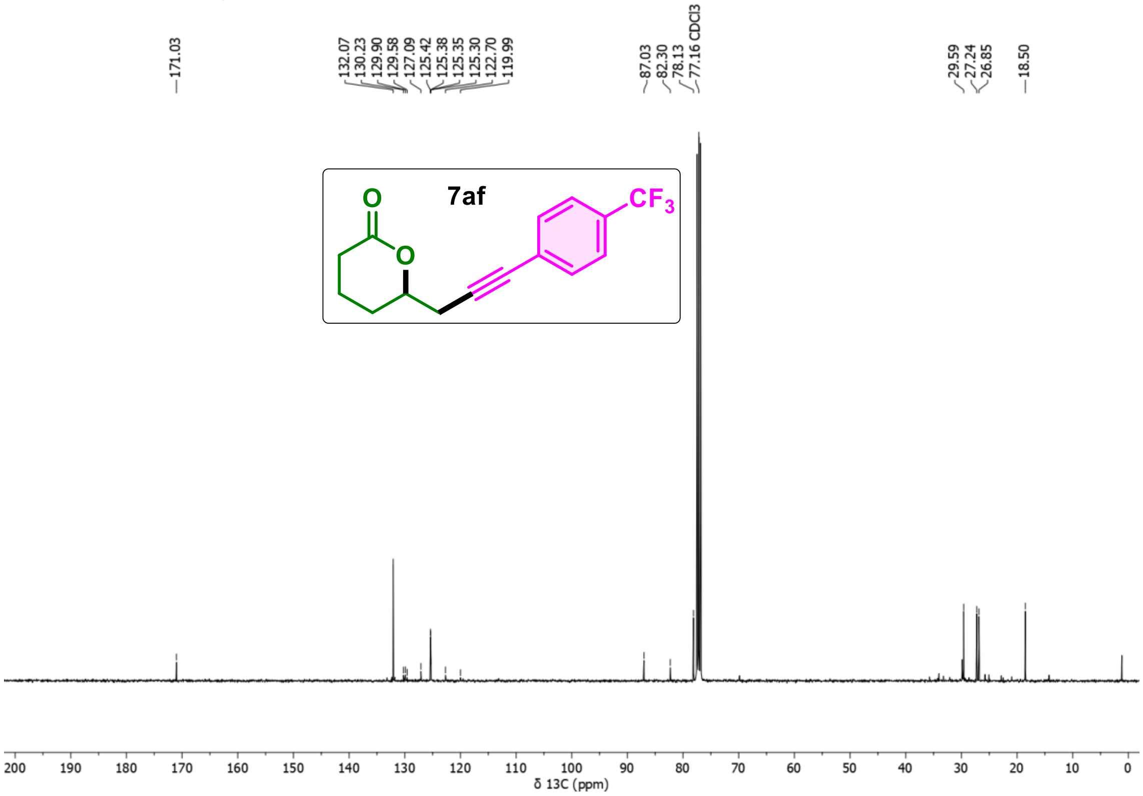

Table of contents

^{1}H NMR 300MHz, $CDCl_3$

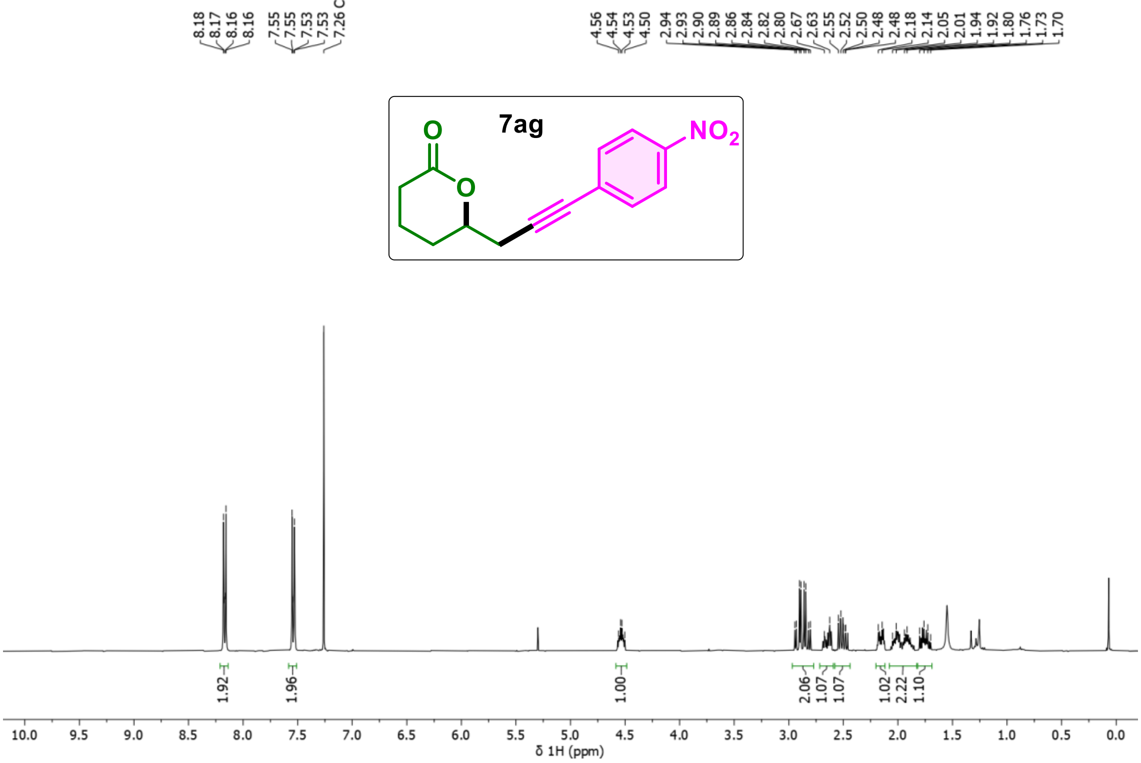

^{13}C NMR 101MHz, $CDCl_3$

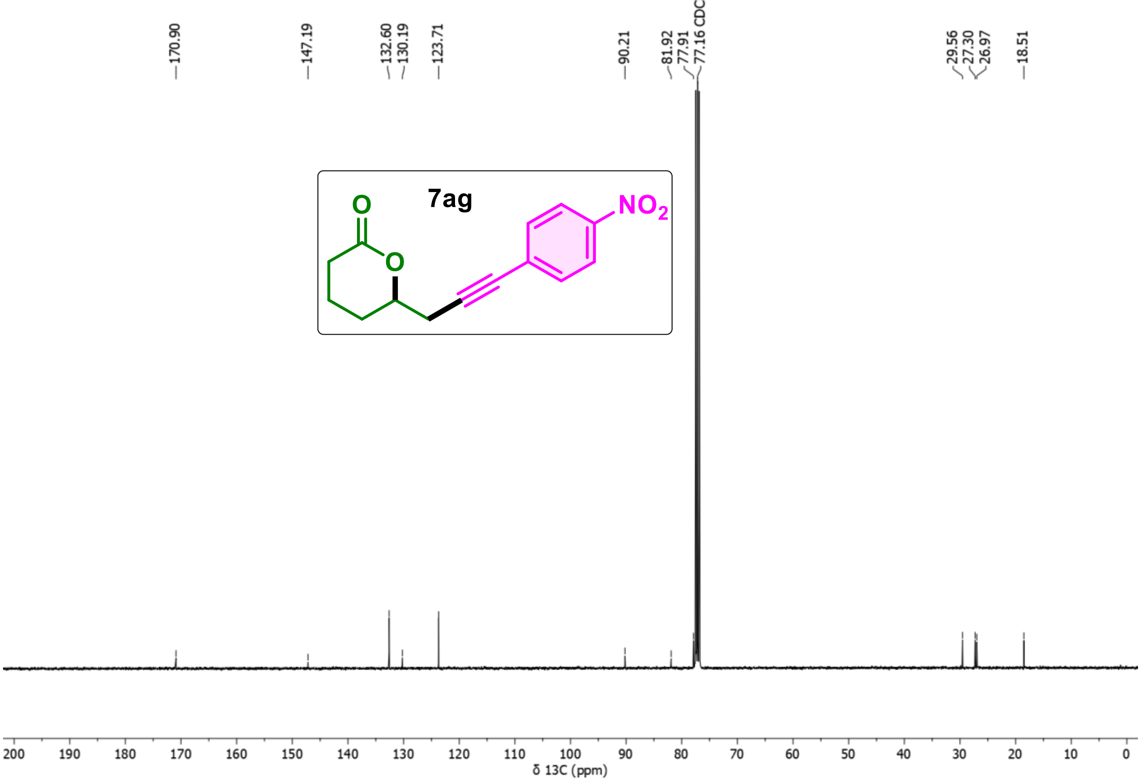

^{1}H NMR 300MHz, $CDCl_3$

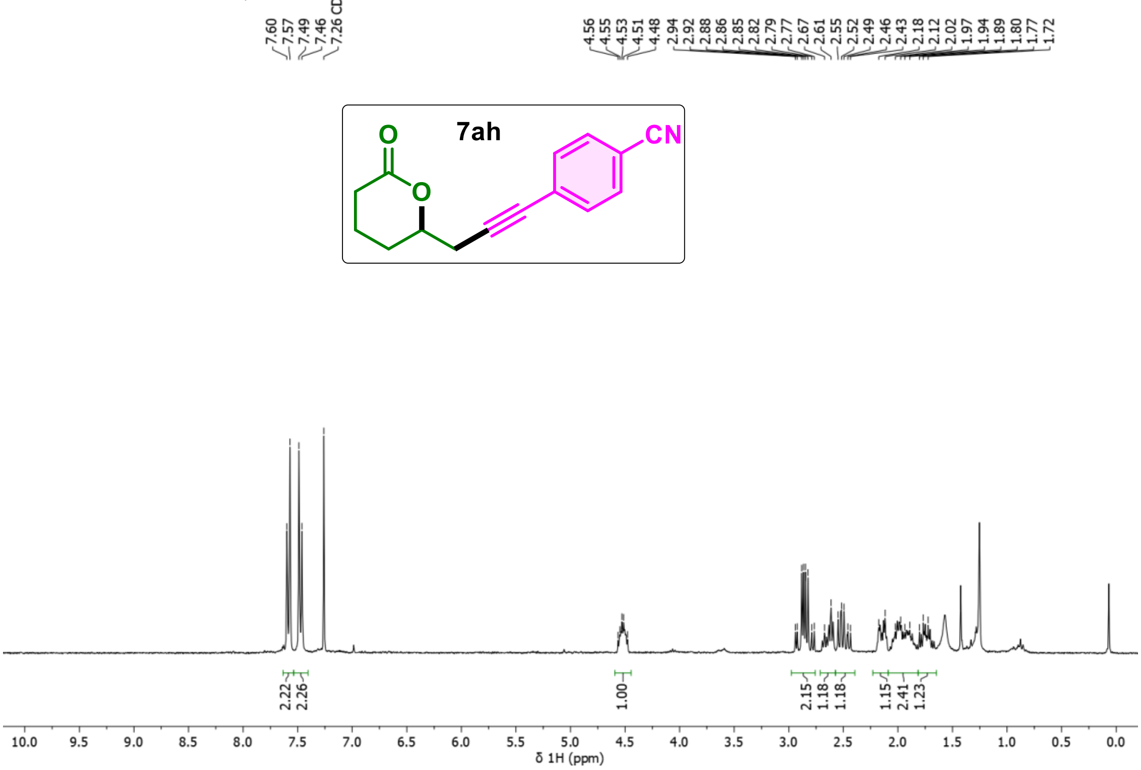

^{13}C NMR 101MHz, $CDCl_3$

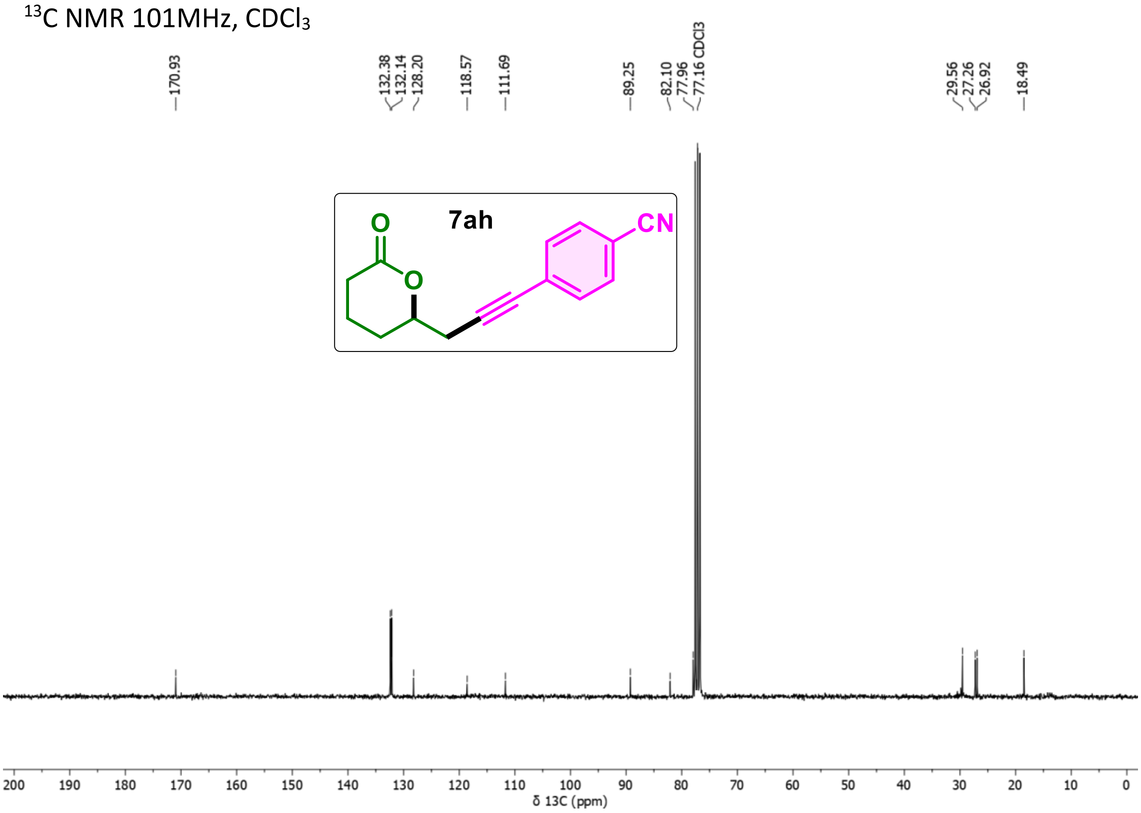

^{1}H NMR 300MHz, $CDCl_3$

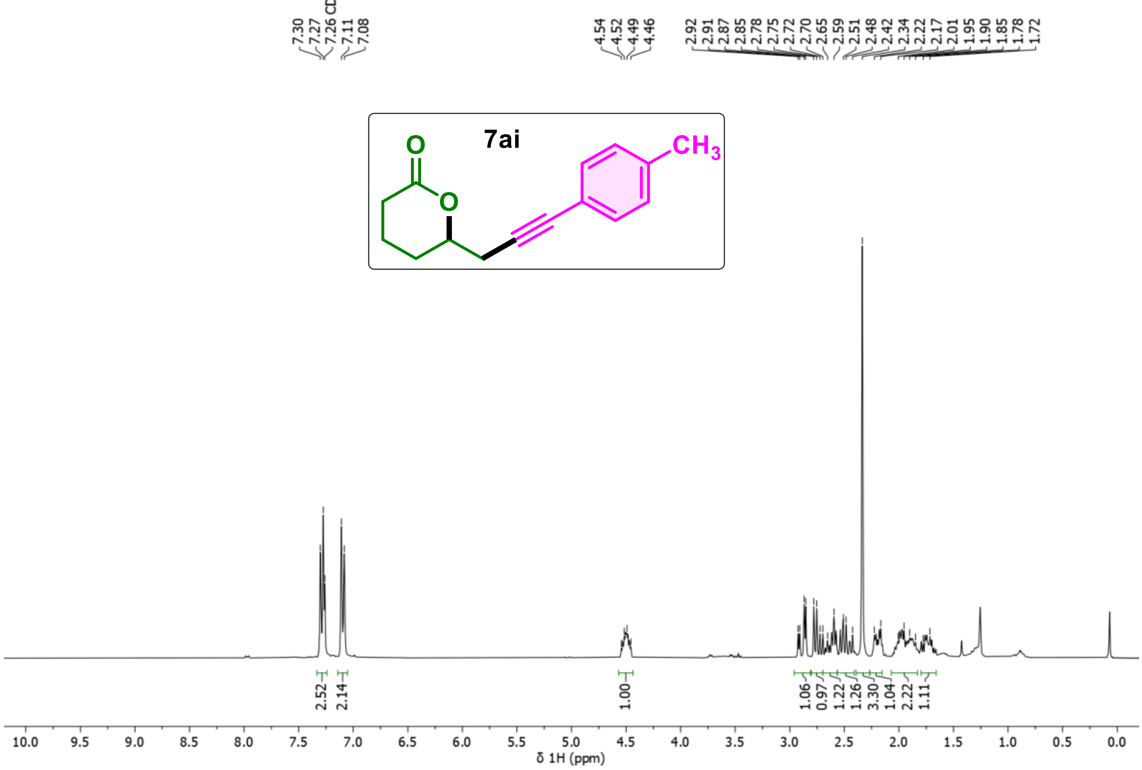

^{13}C NMR 101MHz, $CDCl_3$

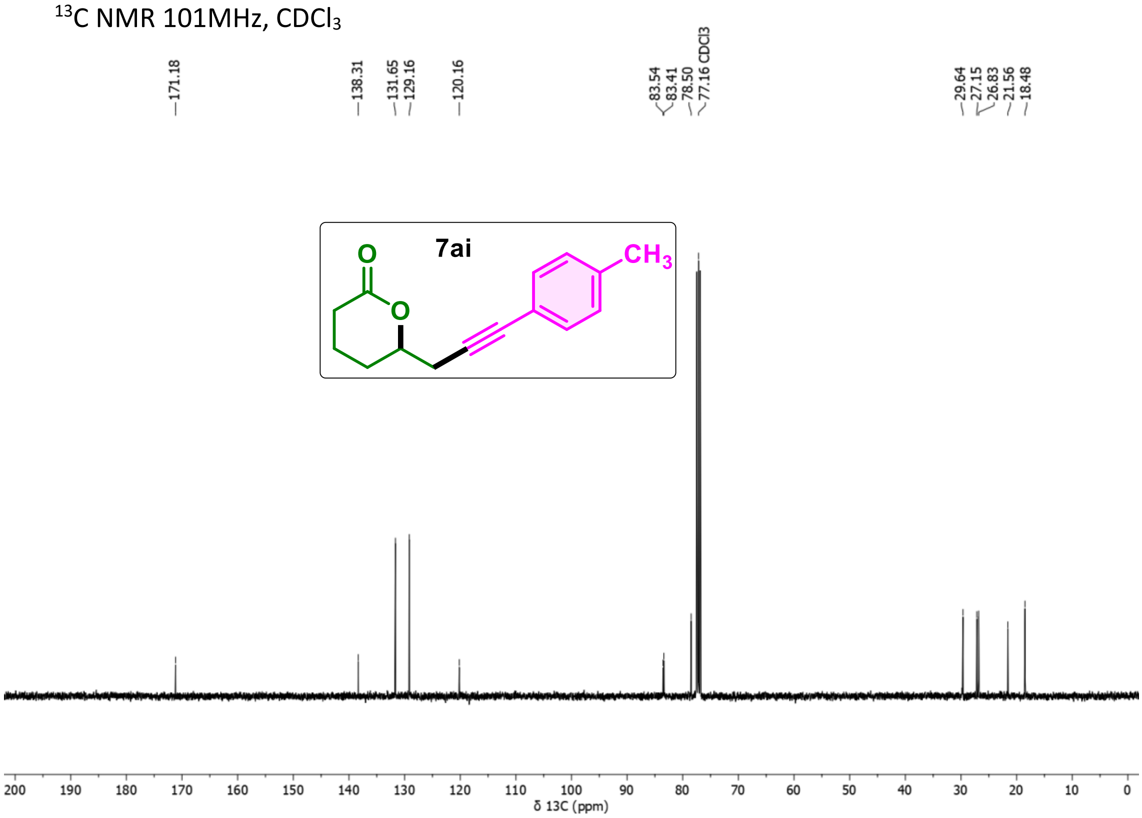

Table of contents

^{1}H NMR 300MHz, $CDCl_3$

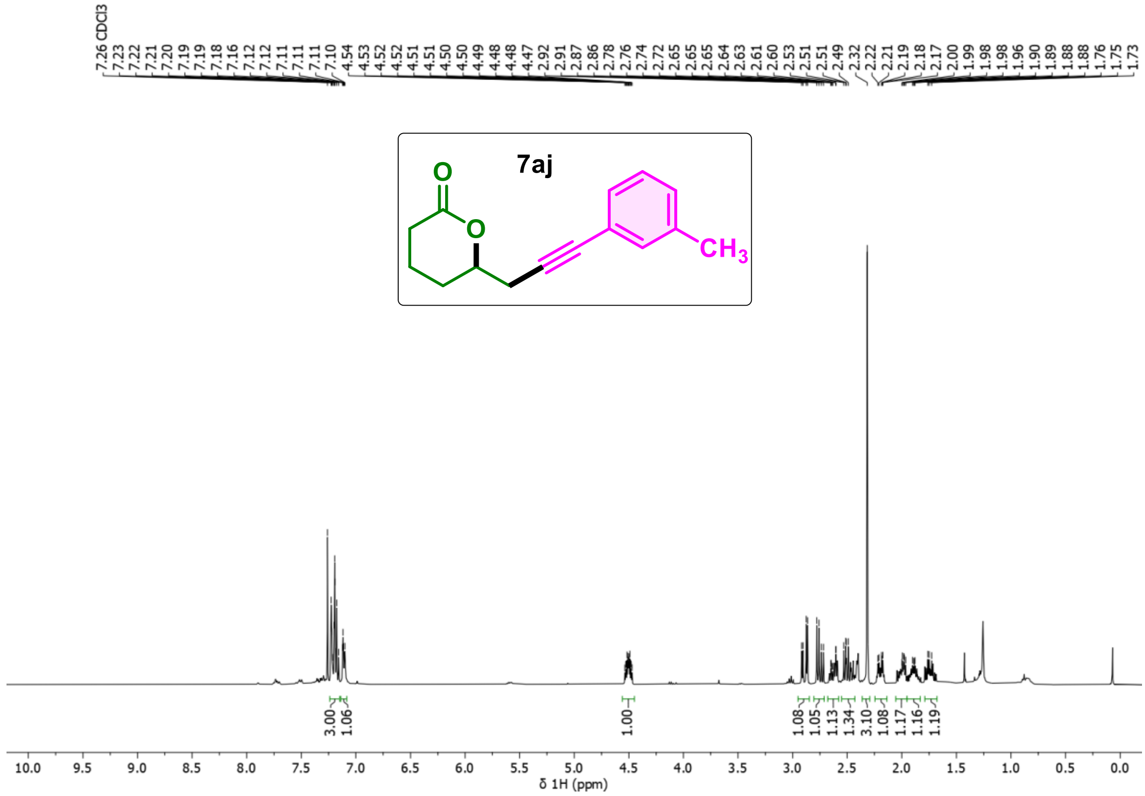

^{13}C NMR 101MHz, $CDCl_3$

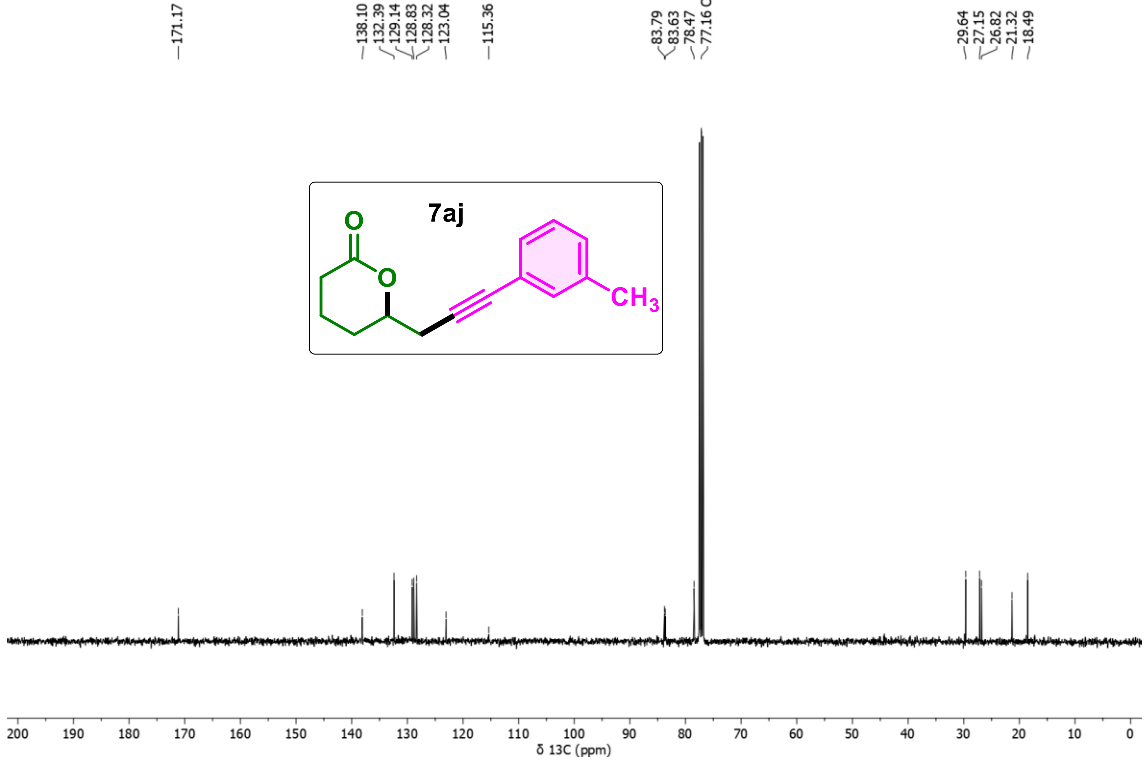

^{1}H NMR 300MHz, $CDCl_3$

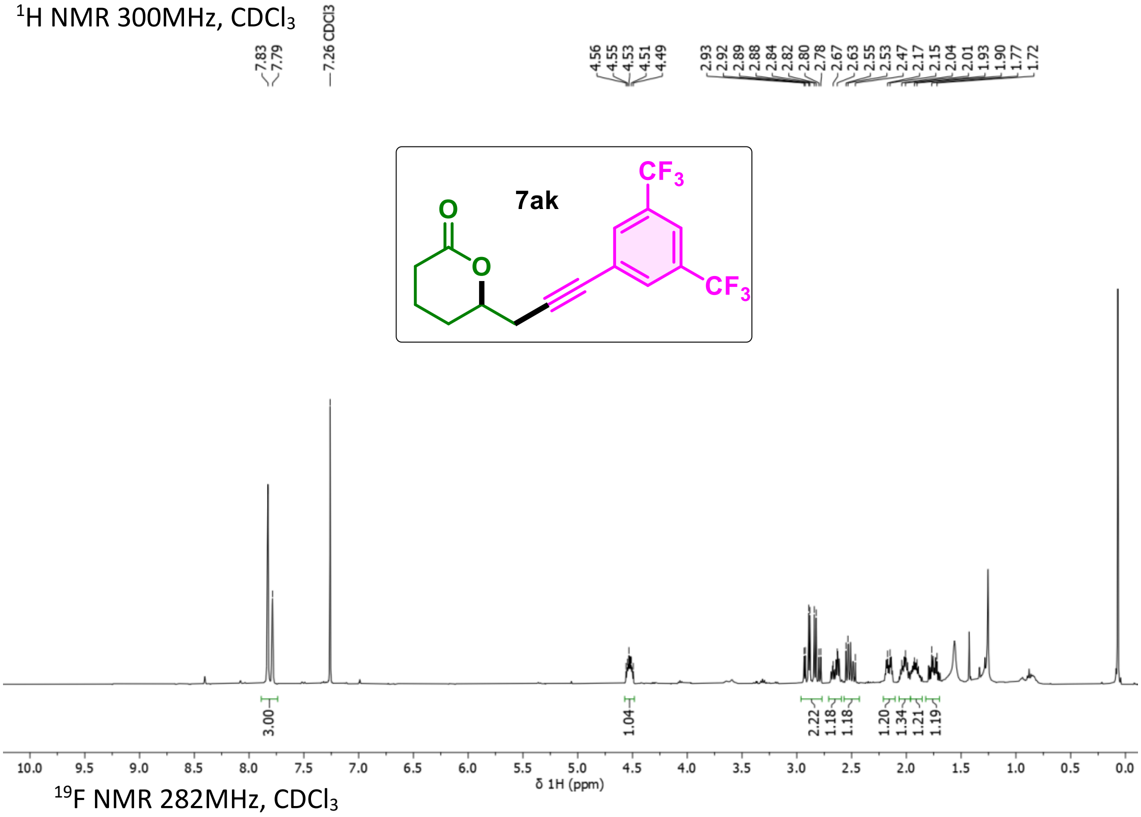

^{19}F NMR 282MHz, $CDCl_3$

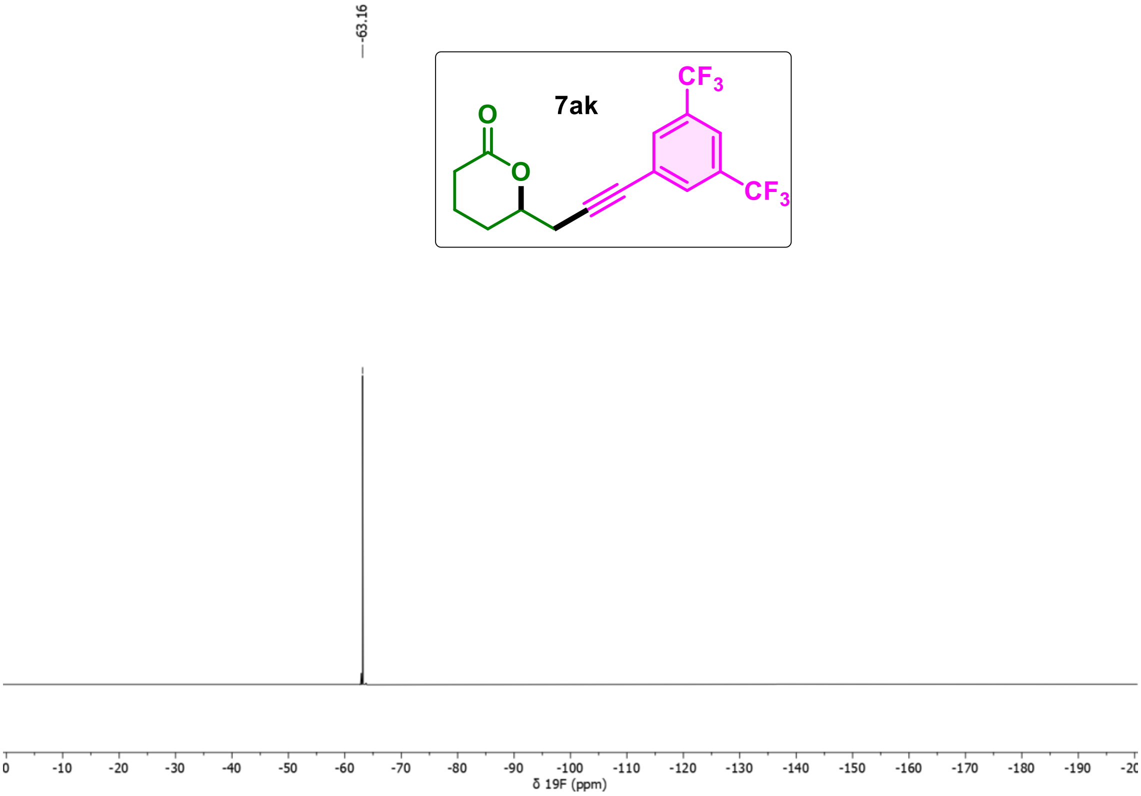

^{13}C NMR 101MHz, $CDCl_3$

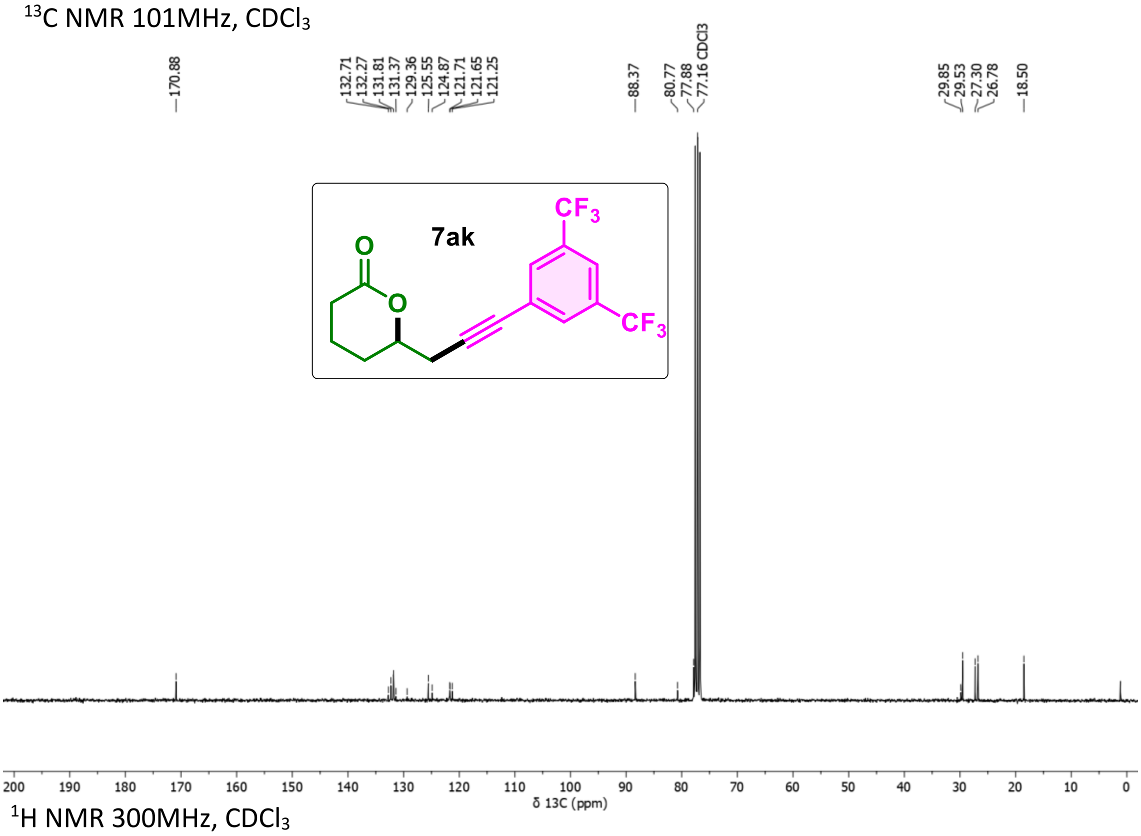

^1H NMR 300MHz, $CDCl_3$

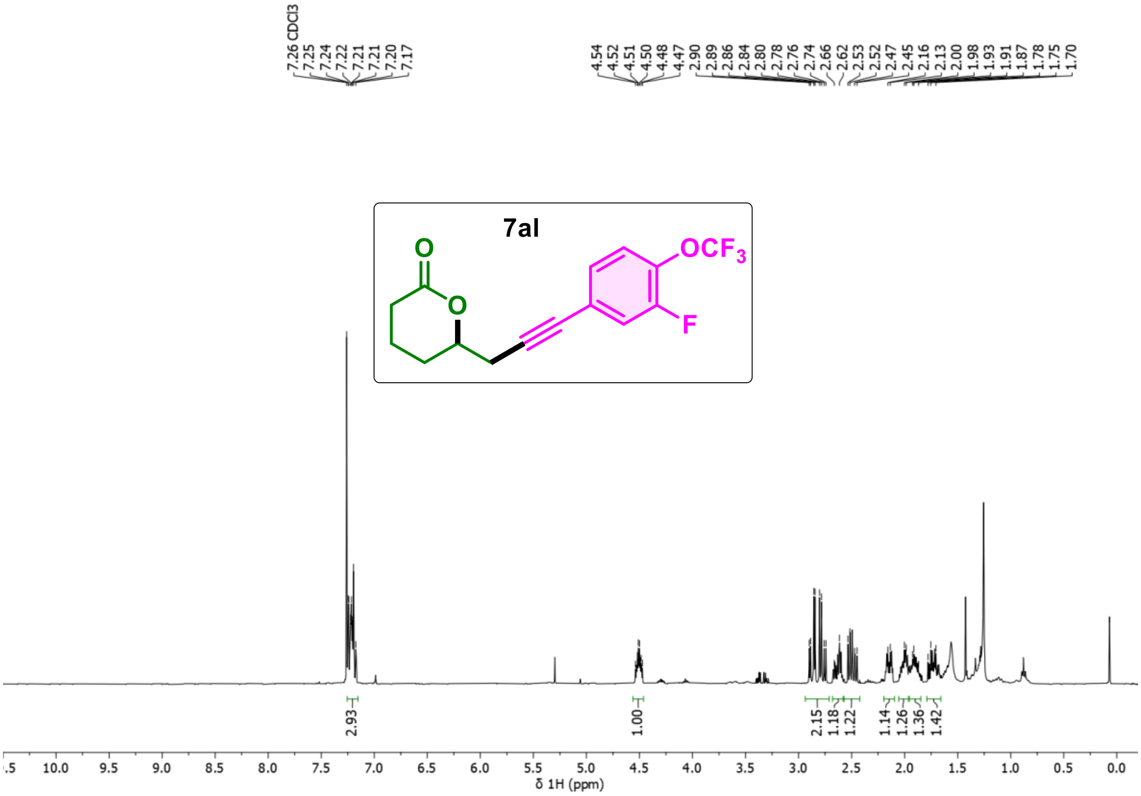

^{19}F NMR 282MHz, $CDCl_3$

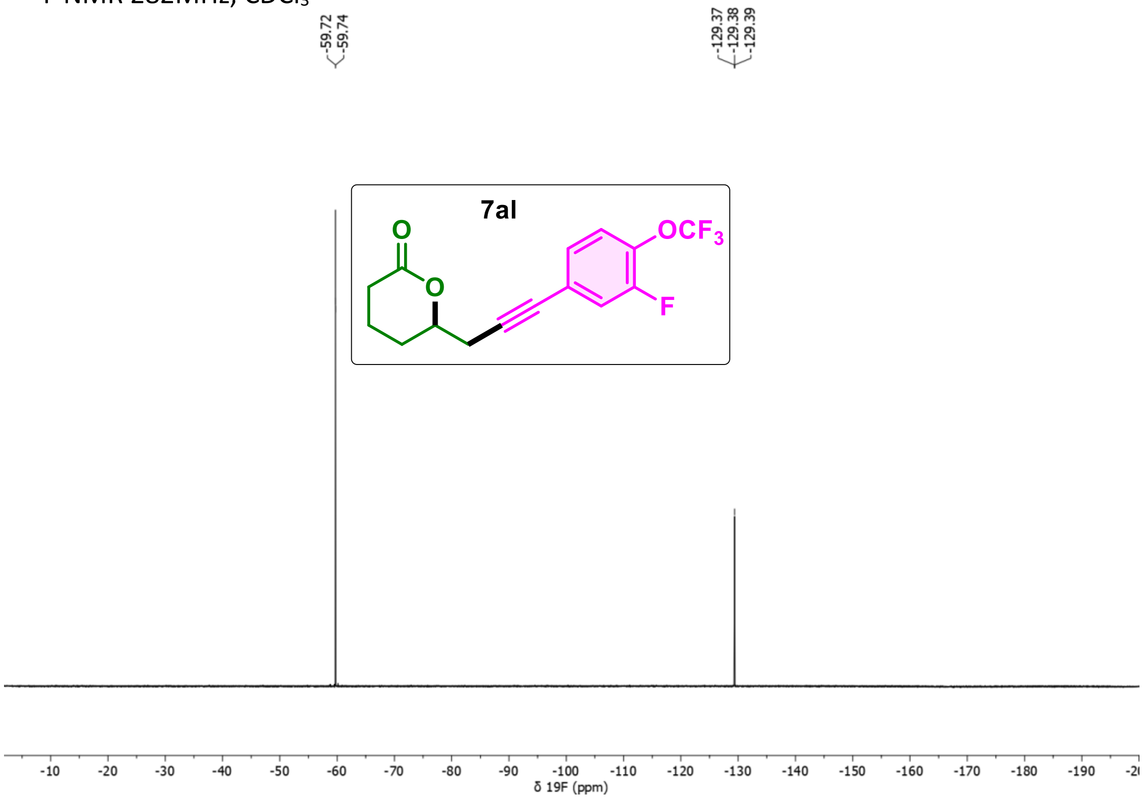

^{13}C NMR 101MHz, $CDCl_3$

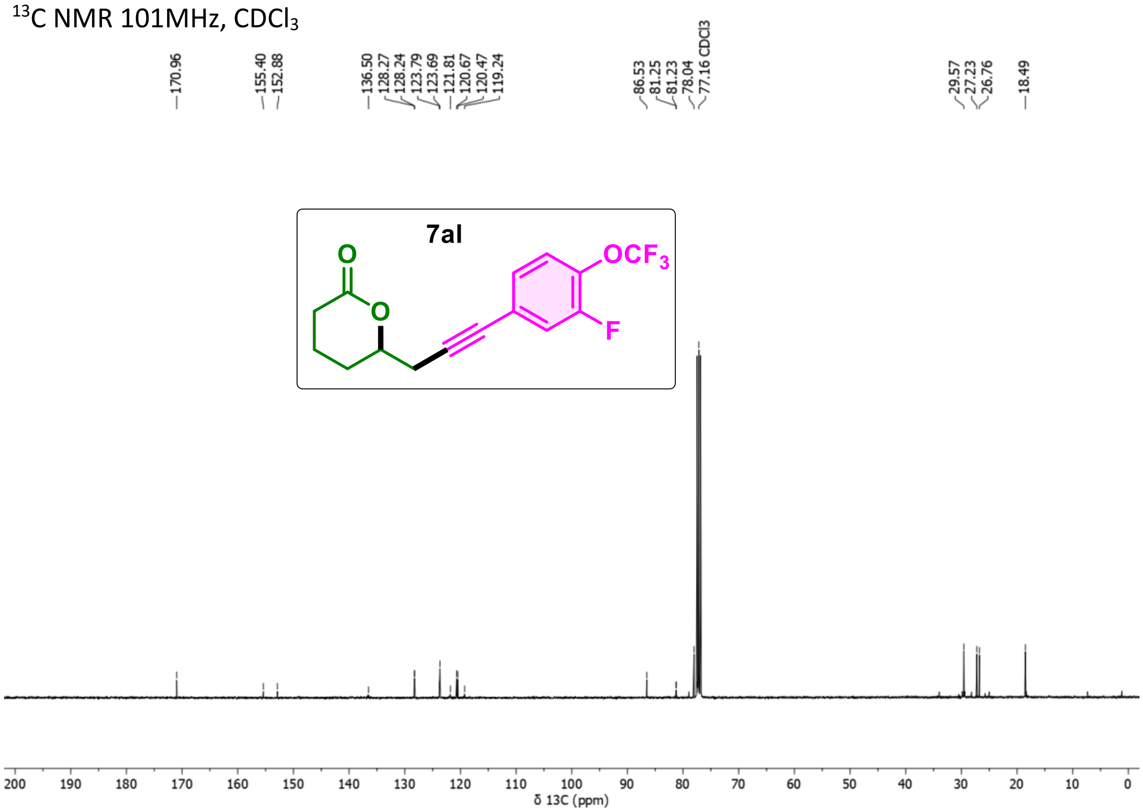

^{1}H NMR 300MHz, $CDCl_3$

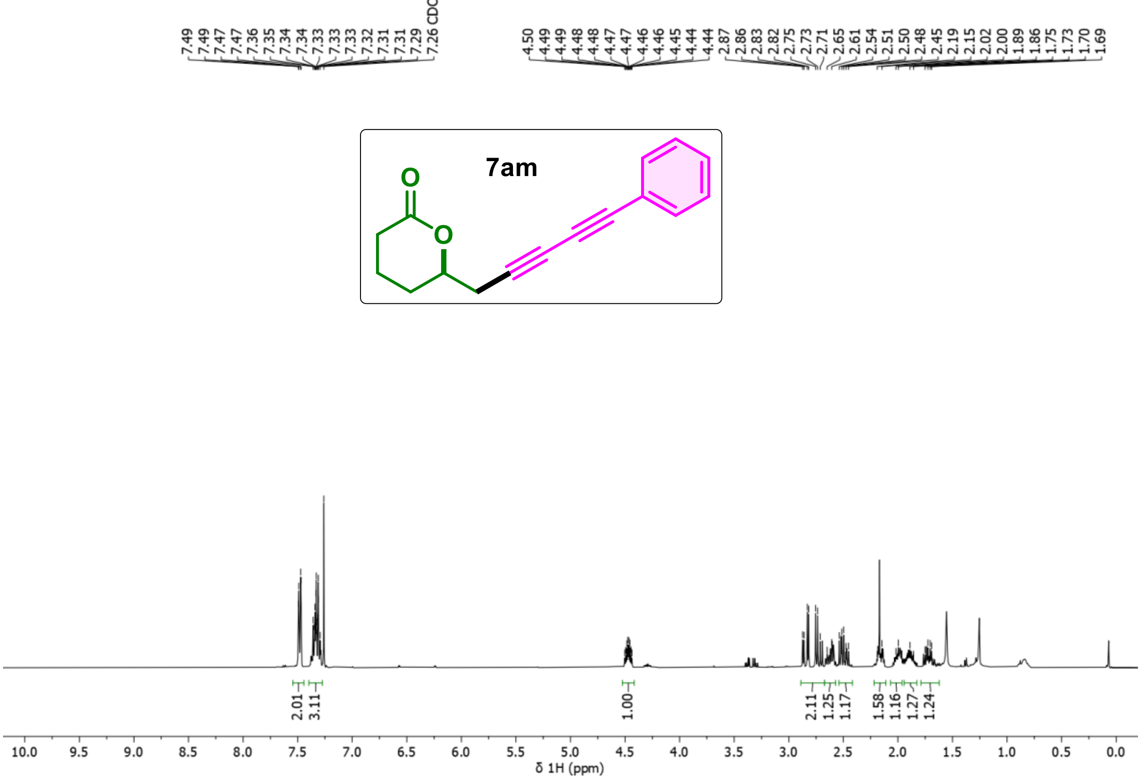

^{13}C NMR 101MHz, $CDCl_3$

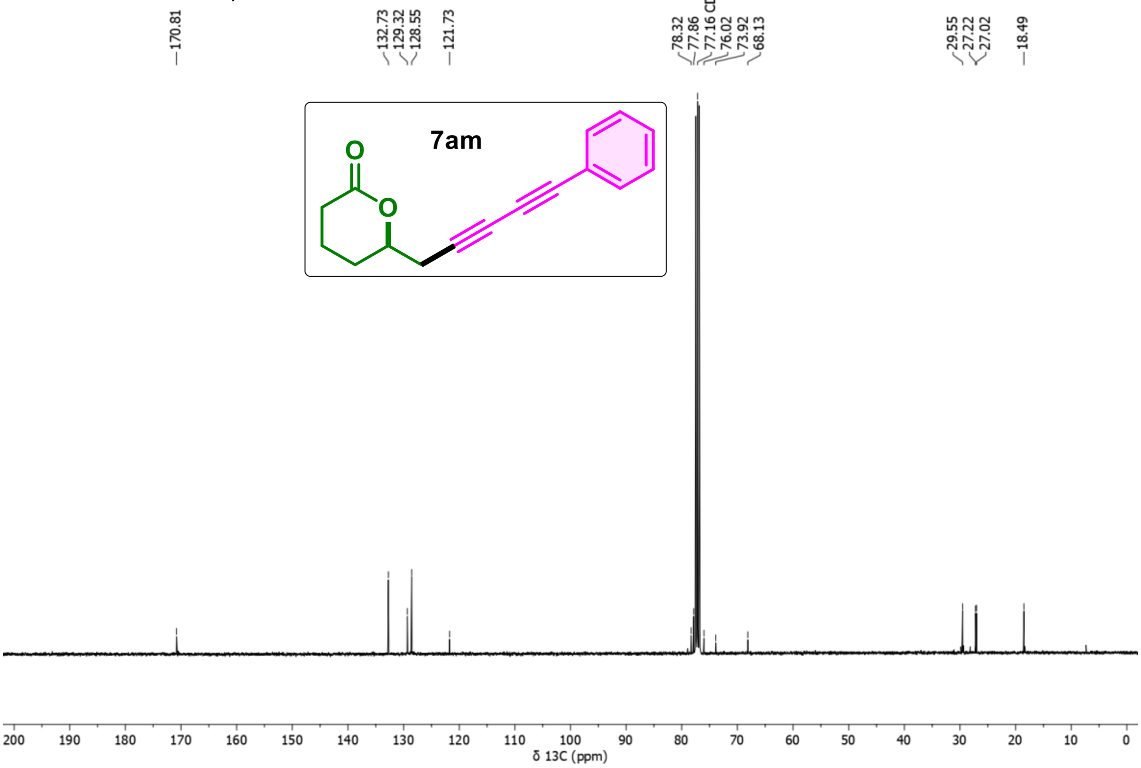

Table of contents

^{1}H NMR 300MHz, $CDCl_3$

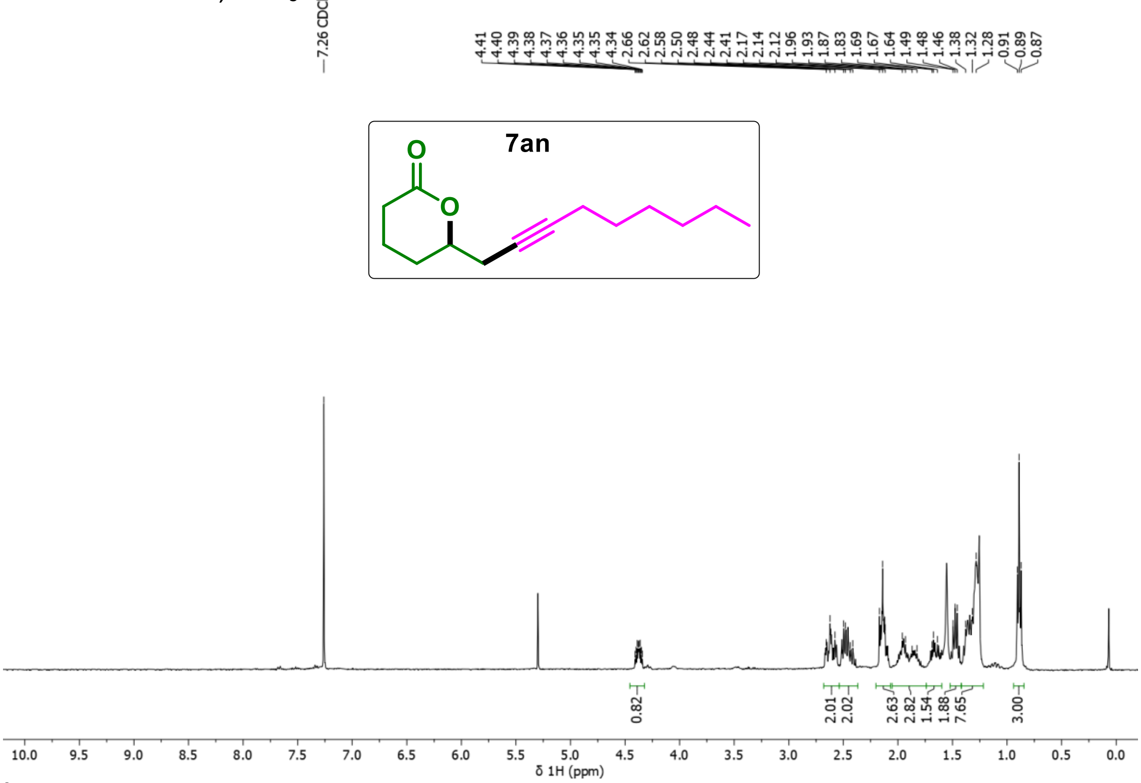

^{13}C NMR 101MHz, $CDCl_3$

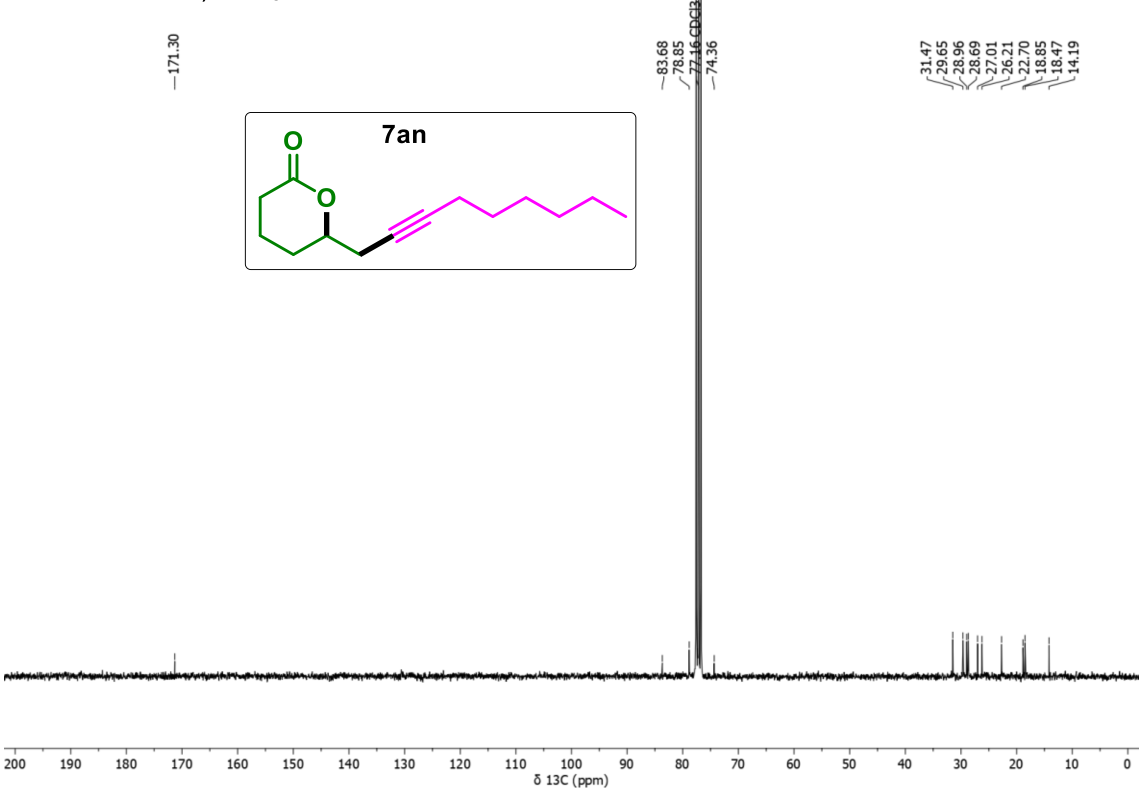

^{1}H NMR 300MHz, $CDCl_3$

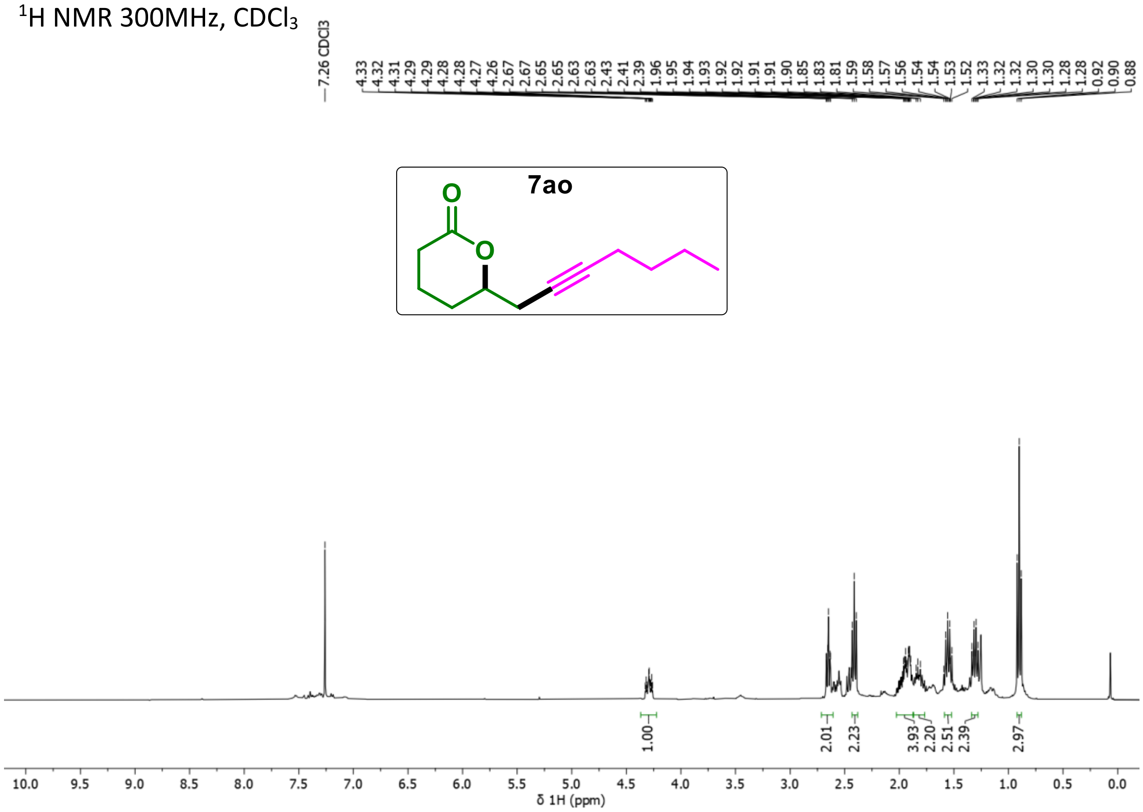

^{13}C NMR 101MHz, $CDCl_3$

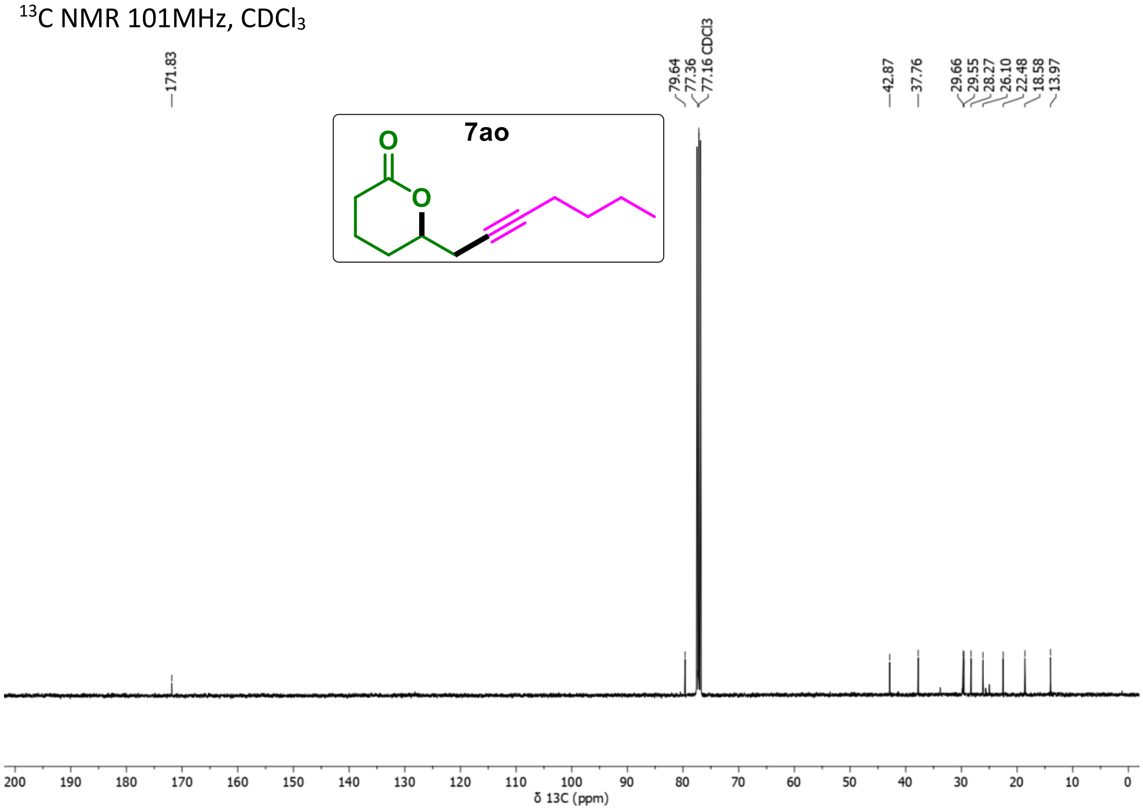

Supplement: Supplementary file 1 [file ol6c01067_si_001.pdf]
